# Global gene expression defines faded whorl specification of double flower domestication in *Camellia*

**DOI:** 10.1038/s41598-017-03575-2

**Published:** 2017-06-09

**Authors:** Xinlei Li, Jiyuan Li, Zhengqi Fan, Zhongchi Liu, Takayuki Tanaka, Hengfu Yin

**Affiliations:** 1 Research Institute of Subtropical Forestry, Chinese Academy of Forestry, Fuyang, Zhejiang, 311400 China; 2 Key Laboratory of Forest genetics and breeding, Chinese Academy of Forestry, Fuyang, Zhejiang, 311400 China; 3 Departments of Cell Biology and Molecular Genetics, University of Maryland, College Park, MD USA; 4 Department of Plant Science, School of Agriculture, Tokai University, Minami-aso-mura, Aso-gun, Kumamoto, 869-1404 Japan

## Abstract

Double flowers in cultivated camellias are divergent in floral patterns which present a rich resource for demonstrating molecular modifications influenced by the human demands. Despite the key principle of ABCE model in whorl specification, the underlying mechanism of fine-tuning double flower formation remains largely unclear. Here a comprehensive comparative transcriptomics interrogation of gene expression among floral organs of wild type and “formal double” and “anemone double” is presented. Through a combination of transcriptome, small RNA and “degradome” sequencing, we studied the regulatory gene expression network underlying the double flower formation. We obtained the differentially expressed genes between whorls in wild and cultivated *Camellia*. We showed that the formation of double flowers tends to demolish gene expression canalization of key functions; the faded whorl specification mechanism was fundamental under the diverse patterns of double flowers. Furthermore, we identified conserved miRNA-targets regulations in the control of double flowers, and we found that miR172-AP2, miR156-SPLs were critical regulatory nodes contributing to the diversity of double flower forms. This work highlights the hierarchical patterning of global gene expression in floral development, and supports the roles of “faded ABC model” mechanism and miRNA-targets regulations underlying the double flower domestication.

## Introduction

Human selection of plant traits through breeding is the foundation of modern day agriculture that supports human subsistence. The process of plant domestication is rooted in the genetic modification of genes that regulate plant development and growth. Therefore, plant domestication is a special class of evolution influenced by human behavior^1^.

The showy double flower in many ornaments is a typical example of “domestication syndrome” (traits that are different to their progenitors) and thought to be controlled by genetic selection of a small number of genes^2, 3^. It is recognized that several groups of floral homeotic genes, mainly the ABC(E) genes, played central roles in directing floral forms both in floral organ identify and number^4, 5^. The well-established ABCE model on flower development elegantly explains how a few key regulatory genes could control floral organ identity, which then informs (or directs) subsequent floral organ development characteristic of the specific floral organ^4, 5^. Under the guidance of the ABCE model, double flower domestication in many cases was found to be associated with the alterations of the C function gene^3^. For example, in the cultivated rose, the incensement of petal numbers was correlated with the reduced domain of C class gene expression^6^. Although the ABCE model was shown to be largely conserved in many other higher plants, refinements were proposed by individual studies in plant species with noncanonical floral structures. For example, the ‘inside-out’ whorl specification in *Lacandonia* was matched with recombination of B and C function genes^7^; the petaloid bracts in dove tree were likely associated with ectopic expression of petal and stamen genes^8^; the expression analysis of C function orthologous gene in daffodil indicated that the corona was a structural innovation between petal and stamen^9^. These studies largely supported the ABCE model by showing how modified expression domains of A, B, C, E genes led to morphological innovations^5^. Nevertheless, to form a functional organ with defined shape, the subsequent morphogenesis of a floral organ is far more complicated. As examined in *Arabidopsis* and *Antirrhinum*, the petal development required organized cell division and growth in different zones controlled by gene and plant hormones^10, 11^. Hence, it has been difficult to investigate molecular mechanisms of floral organogenesis.

In recent years, global transcriptomic analyses of floral organs in the basal and eudicot flowering plants provided another means to understand the evolution of floral forms. In parallel to the molecular genetics studies in model plants, the feasibility of obtaining gene expression profiles at the genome scale was revolutionary in the field of floral evolution^12, 13^. It appeared that the ABCE model was established based on the study of highly derivative plant species; the ‘shift of boundary’ (floral structure evolution involved changes of spatial expression of ABCE genes) or ‘faded whorl specification’ (gradient expression of regulatory genes was rudimentary for establishing floral genetic programs) was proposed based on comparisons with the basal angiosperm flowers^5^. Genomics characterizations from the broader phylogenetic sampling revealed that the faded whorl specification might represent a ‘default’ state, and the diversity of floral forms required the canalization of gene expression patterns such as, the whorl-specific expression of floral regulatory genes^12^.

It is now clear that multiple pathways were involved in the regulation of floral forms. The microRNA was an important part of post-transcriptional regulation of gene expression and played critical roles in plant floral development^14^. For example, miR172 targeted A function gene *AP2* and regulated petal development; expression of miR172-resistant *AP2* by the *AP3* promoter caused a special double flower phenotype in *Arabidopsis* ^15^. The miR169-NFYA regulation was required for restraining C class gene expression in *Antirrhinum* and *Petunia* ^16^. Moreover, miRNA-target regulatory modules were found to be required for gamete cell differentiation in stamen (miR156-SPLs)^17^, determination of floral organ boundaries and numbers (miR164-NACs)^18^, and outgrowth of stamens and petals (miR319-TCPs)^19^. With the increasing power of high throughput sequencing, the sequencing of small RNA and Parallel Analysis of RNA Ends (PARE, degradome) offer effective and high throughput ways in identifying miRNAs and their targets in plant species even in the absence of a reference genome^20, 21^. Indeed, in *Camellia azalea*, the small RNA and transcriptome sequencing were shown to be useful in identifying key regulatory genes including conserved and novel miRNAs, and evolution of miRNA genes was predictable in the context plant evolution^22, 23^. It is unclear if these miRNAs target genes are involved in the regulation of double flower development.

**Figure 1:**
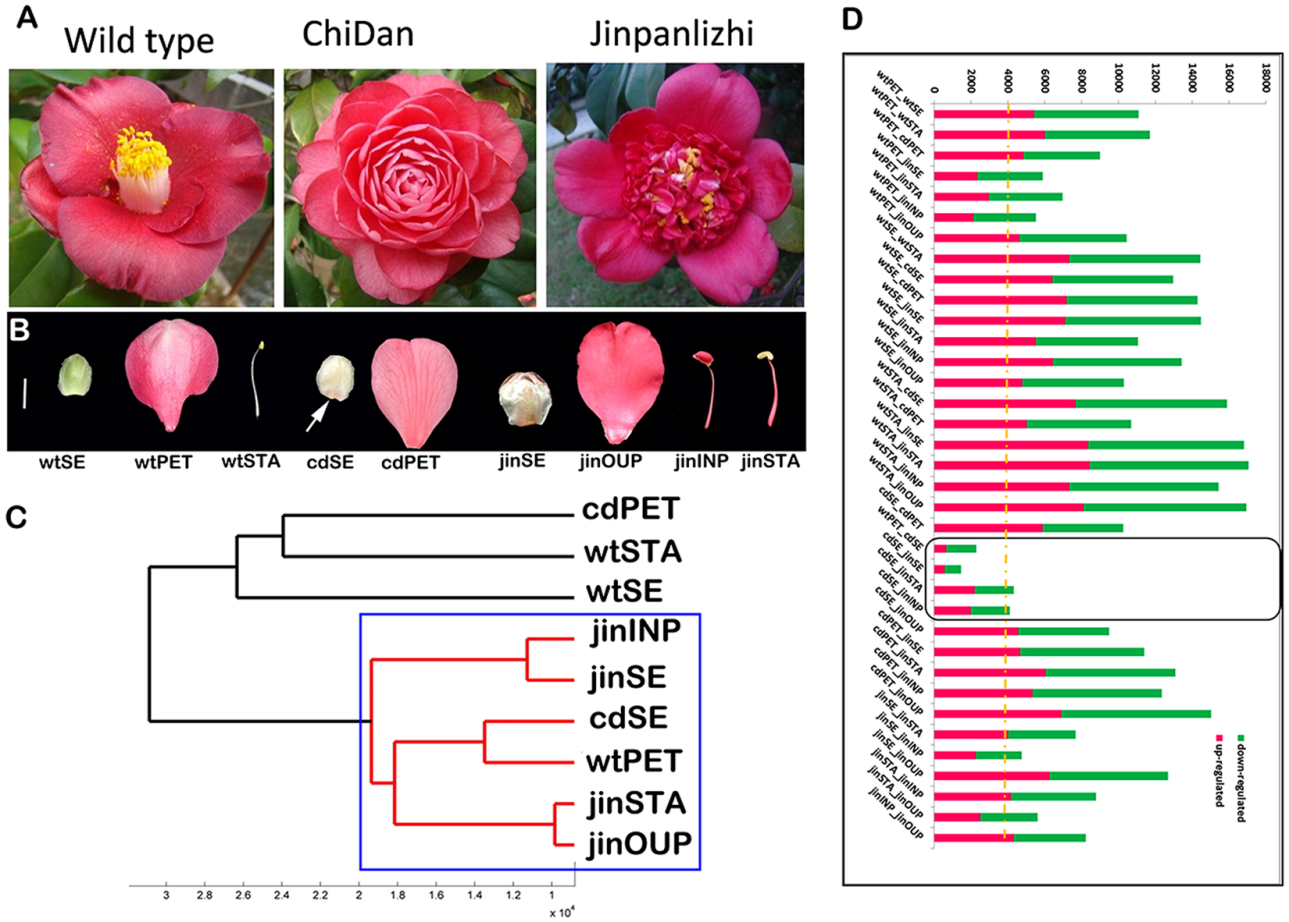
Comparative transcriptomics analysis in wild and doubled camellias. (**A**) Overviews of wild and double flower varieties. From left to right, wild, formal double “ChiDan”, anemone double “Jinpanlizhi”. (**B**) Floral tissues for the transcriptomics study. Arrow indicates the pigmentation in sepal of ChiDan. White bar, =1 cm. (**A**,**C**) dendrogram plot of clustering of samples by the mean expression levels. Distance less than 20,000 was highlighted by red lines (inside the blue rectangular). (**D**) The summary of numbers of DEGs among floral organs. Red, up-regulated genes; green, down-regulated genes. wtSE, wild type sepal; wtPET, wild type petal; wtSTA, wild type stamen; cdSE, ChiDan sepal; cdPET, ChiDan petal; jinSE, Jinpanlizhi sepal; jinOUP, Jinpanlizhi outer petal; jinINP, Jinpanlizhi inner petal; jinSTA, Jinpanlizhi stamen.

Cultivated *Camellia japonica* is a world-wide ornamental flower and has been domesticated over centuries. Its remarkable diversity of floral forms imparts a rich resource for understanding the genetic regulation of floral patterning and forms^24^. For instance, the double flowers in *Camellia* include 5 distinctive types: semi, formal, anemone, rose, and peony doubles, mainly distinguished by the number and arrangement of petals and stamens. The wild *Camellia* flower was defined as a single flower, with one row of overlapping petals (usually less than 8), and a columnar stamen cluster and one normal pistil in the center; the anemone form was characterized with several outer rows of large petals, with many petaloid stamens, and few degenerated stamens and pistils. The center of anemone double was clustered with many small petals, and the petaloid stamens and degenerated stamens and pistils were generally covered in the small petal cluster. The formal double completely lacked stamen and pistil organs with replacement of many rows of petals (Fig. 1A). The cherished varieties of ‘Wabisuke’ and ‘Higo’ camellias somehow represented mitigated and augmented stamen growth respectively^25^. Double flowering in camellia cultivars is also a phenomenon of interest especially for its proliferated and redundant floral structure. An unusual flowering phenomenon had been observed in an unnamed camellia cultivars characterized by a formal double flower of perfect symmetrical form^26^. Previous studies in *Camellia* characterized the expression of A-, and C- function genes and suggested that double flower domestication were involved in multiple pathways^27^. Interestingly, the expression of C function ortholog (*CjAG*) displayed opposite patterns in petals of formal and anemone doubles (expression of *CjAG* suppressed in formal double, but upregulated in anemone double)^27^. Despite the extensive knowledge on genetic regulation of floral development in the model plant species, it remains unclear how the floral patterns in cultivated *Camellia* were achieved.

In this work, we performed high-throughput transcriptome sequencing in wild, formal double and anemone double types of *Camellia* to capture the genome-wide gene expression patterns related to double flower formation. We identified differentially expressed genes among organ types in both wild and cultivated *Camellia* and analyzed their functional properties by comparing homological structures. Small RNA and degradome sequencing identified conserved miRNA-targets pairs and their potential contribution to different types of double flowers. We found that double flower domestication in camellia tends to deteriorate specified expression patterns of regulatory genes. Further, the development of inner floral organs, a characteristic that distinguishes between double flower types, appear to have promiscuous organ identity influenced by positional effects of gene expression in the zones of floral bud. Moreover, some key miRNA-targets, such as miR172-AP2, miR156-SPLs, were identified as important molecular signatures for the classification of double flower types. These findings may highlight the orchestration of genes controlling floral organ identity, miRNA-targets underlying the floral patterning and their contributions to the double flower domestication.

## Results and Discussion

### Transcriptome sequencing for gene discovery and gene expression profiling in wild and cultivated *C. japonica*

To generate a comprehensive genomic resource in *C. japonica* for gene discovery, RNAs from a mixed sample containing leaf, shoot, floral bud, sepal, petal, stamen and carpel were sequenced by Illumina Hiseq platform, which yielded over 45.7 million 2 × 125 pair-end reads. Raw reads were filtered and assembled using the Trinity software, and 104, 810 unigenes were obtained with N50 of 893 bp (Supplementary Table S1). The unigene sequences were assessed by alignments to various databases for annotation (details in Supplementary Table S2), and 37384 unigenes were annotated with at least one hit in all databases (Supplementary Table S2). Notably, 78.3% of annotated unigenes (29265) were over 299 bp indicating a competent transcriptome in *C. japonica* for gene identification. The assembly of 104,810 unigenes served as the reference for subsequent RNA-seq analysis.

Cultivated *Camellia* contains more than 5 types of double flowers with distinctive floral forms. To understand the global signatures of gene expression between different organ types, we constructed 27 libraries of 9 tissue types from wild (sepal, petal, stamen), formal double (sepal, petal) and anemone double flowers (sepal, outer petal, inner petal, petaloid stamen) with 3 biological replicates for next generation sequencing (Fig. 1A,B). Around 12.3 million reads (single end 50 bp in length) on average were generated for each library with a mean value of 92.8% for over Q30 bases (Supplementary Table S3). The above transcriptome assembly was served as a reference for gene expression quantification. The mapped reads were converted into RPKM for the expression level of unigene (see Material and Methods). To assess the expression data between replicates and tissues, the Pearson’s correlation coefficient between every pair was calculated (Supplementary Figure S1). The correlation values among replicates were mostly over 0.9 (Supplementary Figure S1), which indicated that the results were reliable. The samples ‘jinOUP-3′ and ‘jinSTA-3′ were poorly correlated (Pearson’s correlation <0.2) with other two biological replicates and therefore removed for further analysis. ‘jinSE-1′ and ‘cdSE-2′ displayed low correlation within biological replicates (Pearson’s correlation <0.73) and were also removed from analysis.

### Global analysis of gene expression between wild and cultivated *Camellias* identified DEGs among various floral organs

The primary objective of this study was to capture the global expression signatures between flower types by identifying significant differentially expressed genes (DEGs) between organs types and between different double flower types. To generate a top-down view of gene expression profiles, we performed clustering of global expression of mean values of replicates. The Euclidean distances between organ types were calculated and clustered to display the resemblance of organs at the level of gene expression. We showed that, in wild type *Camellia*, each of the floral organs, sepal, petal and stamen, exhibit distinct morphology and easily separable (Fig. 1B). While some of the sepals from formal double (ChiDan) exhibit some petal-like pigmentation spots (Fig. 1B). In contrast, large numbers of inner petal, and petaloid stamen arise and cluster together in the center of the anemone double flower (Fig. 1A) suggesting the continuous differentiation and proliferation of inner petal/stamen like organs. To determine the genes that were preferentially expressed in specific organs, we performed statistical analysis to identify significant DEGs between tissue types (with fold change > = 2, P-value <0.05). The numbers of up and down regulated unigenes were generally comparable between pairs of organs (Fig. 1D). It was not surprising that, between each pair of wild *Camellia* organs, there were over 10,000 DEGs considering distinctive floral organ identities (Fig. 1D). Nevertheless, the putative homologous organs displayed large numbers of DEGs between wild and different types of double flowers. For instance, wild and formal double sepal (wtSE_cdSE) had over 12,000 DEGs and wild and anemone double stamen (wtSTA_jinSTA) had the largest number of DEGs (Fig. 1D). Also, markedly less DEGs among cdSE_wtPET, cdSE_jinSE, cdSE_jinINP and jinSTA_cdSE were revealed (Fig. 1D) underlying complex regulatory networks might be responsible during the domestication of *Camellia* double flowers, and development of these organs might require some concurrent processes. Both formal doubled and anemone doubled *Camellia* cultivars displayed highly proliferated growth of petal or petal-like organs, while the differentiation of extra organs varied remarkably in terms of shape and cell types (Fig. 1A). These results suggested that the double flower domestication may have a role in shaping the global gene expression landscapes during the whorl specification and outgrowth.

### Common and specific sets of DEGs in structural homologous organs of wild and double flowers

**Figure 2:**
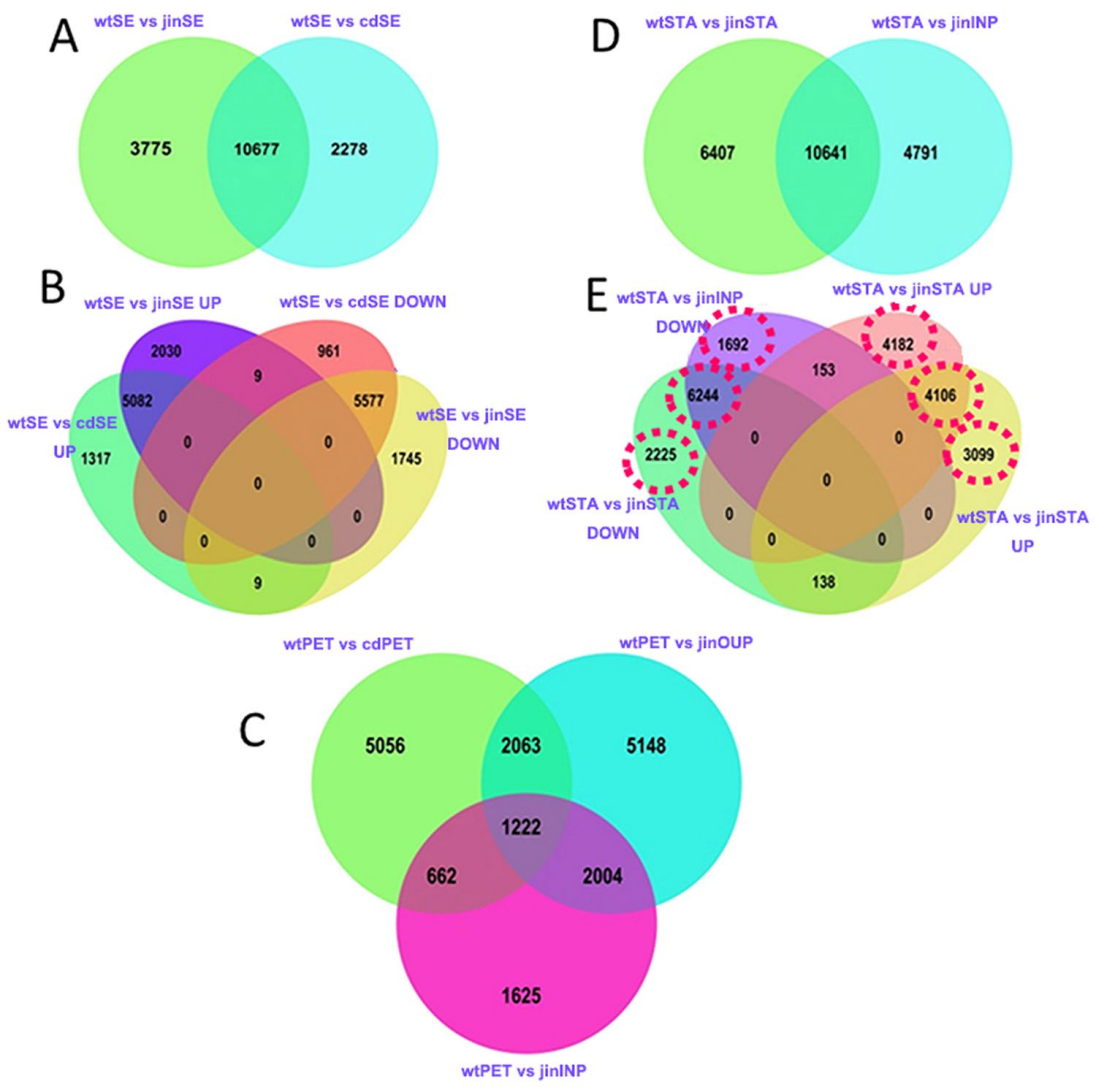
The venn diagram plots of distribution of DEGs. (**A**) Common and special DEGs between sepals of wild and double flower cultivars. (**B**) 4-way venn diagram of up and down regulated DEGs between sepals of wild and double flower cultivars. (**C**) Common and special DEGs between petals of wild and double flower cultivars. (**D**) Common and special DEGs between stamen like organs of wild and double flower cultivars. (**E**) 4-way venn diagram of up and down regulated DEGs between stamen and inner organs of wild and double flower cultivars. The sample labels were described in Fig. 1, and UP indicated up-regulated genes, DOWN indicated down-regulated genes in each comparsion.

To investigate further characteristics of DEGs, we examined the DEGs between homologous organs in wild and cultivated *Camellia*. In sepals, the DEGs between wild-formal double and wild-anemone double were considerably overlapped (more than 80% of total DEGs, Fig. 2A); majority of DEGs were found in both double flower varieties compared to wild *Camellia*, and almost all DEGs had the same expression pattern of up- or down- regulation (Fig. 2B). These data suggested that domestication of formal and ammonia double might result in the same modifications of sepal development despite the ChiDan sepals had some petal-like pigmentations. The petals in double flower cultivars were diverse in shape and cell types-formal double displayed a gradient change of petal size, and outer petal and inner petal in anemone double were distinctive in petal shape with the filament-like structures in inner petals^28^. The analysis of DEGs between petals showed a different and more complexed pattern than the analysis of sepals. By comparing DEGs from formal double and anemone double to wild, the large proportion of DEGs was specific to the double flower type, and small number of DEGs was shared (Fig. 2C). When comparing stamen-like organ and inner petal from anemone double to wild stamen, we found majority of DEGs had consistent expression patterns (both up- or down- regulated in double flower than wild *Camellia*) despite the morphological distinctions of inner organs, while considerably more DEGs were found to be not overlapped between inner petal and petaloid stamens (Fig. 2D,E). This result suggested the inner petal and stamen of anemone double flower might share some conserved gene expression patterns during development which were different from normal stamen development, but may more related to petal development. The comparisons of up- and down- regulated DEGs showed that about half of up- regulated genes of inner petal and petaloid stamen were not shared, and a relatively large number of down-regulated DEGs was shared (Fig. 2E). Similar to the scenario in sepal, most of genes had consistent regulation patterns, and few genes showed opposite up- or down repression trends in different types of double flowers (Fig. 2E). Significant overlap of DEGs between sepals and inner organs from anemone double versus wild stamen (Fig. 2A,D) implied derivative origins of those double flower organs during domestication.

### Enrichment of biological processes entails the domestication paths relevant to doubled floral types

**Figure 3:**
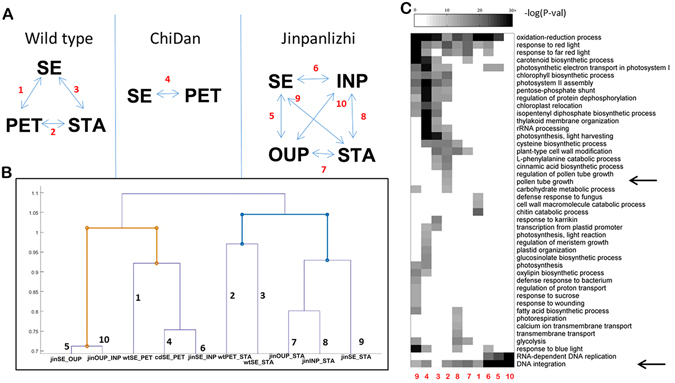
Functional characterizations of DEGs among homologous structural organs. (**A**) The overview of comparisons among organ types in wild and double flower cultivars. (**B**) The computed resemblances based on the presence and significance of all enriched GO terms. (**C**) the highly enriched (*KS* < 0.00001) GO terms and their distributions among comparisons. Arrows indicate GO terms related to pollen tube growth and DNA integration respectively. The numbers indicate the pairs for comparison described in (**A**) SE, sepal; PET, petal; STA, stamen; OUP, outer petal; INP, inner petal.

To gain insights from DEGs, the Gene Ontology (GO) enrichment analysis was performed to identify over-representative GO terms in different categories of DEGs. Significant enriched GO terms were analyzed between pairs of organ types (sepal, petal, stamen) in wild *Camellia*, and some organ-wise significant biological processes, such as photosynthesis, stress responses, hormone signaling, highlighted the activities related to cell growth and differentiation during floral organ development (Supplementary Dataset 1). Meanwhile, the significant enriched biological process could reveal the cumulative gene expression that was required for floral organ development. In order to capture a holistic view in floral development of double flowers, we performed GO enrichment analysis in 10 pairs of organ types (Supplementary Dataset 1; Fig. 3A) based on the whorl identities in wild and doubled cultivars. Significant biological processes were identified (*ks* < 0.05, FDR corrected) and combined as a matrix keeping only the significant GO terms for comparative analysis to test the resemblance of homolog between assayed pairs of organ types. The topology of sample pairs was calculated by the furthest neighbor distance and clustered to reveal the resemblance of organ pairs (Fig. 3B). We found 4 subgroups were formed, and the organ types in wild *Camellia* were classified into independent subgroups, coincided with the structural homology (Fig. 3B). The formal double simply consisted of sepal and petal, and the GO signature was closely correlated with sepal and petal pairs in wild and anemone double (Fig. 3B). In anemone double, a diverse pattern was revealed in which adjacent whorls appeared to be more closely related (Fig. 3B). Whereas the ‘identity’ of inner organs of anemone double seemed to be not strictly constrained, for example, significant processes in 7 (jinOUP-jinSTA) and 8 (jinINP-jinSTA) were clustered together (Fig. 3B). To understand how gene functions were related to the hierarchical relationships, we screened the most significant GO terms in all combinations through (*ks* < = 0.00001), and 44 highly enriched biological processes were identified (Fig. 3C). We found that, the sepal/petal in formal double displayed much more processes comparing to wild sepal/petal which were not found in wild and anemone doubled *Camellia* (Fig. 3C), and some processes were identified in wild petal/stamen and sepal/stamen. Considering the complete lack of stamen and pistil development in formal double, this result suggested gene expression were deployed across whorl specification. Nevertheless, a small number of GO terms were revealed between adjacent whorls in anemone double flower (Fig. 3C). In the anemone double, sepal/outer petal, outer petal/inner petal, inner petal/petaloid stamen displayed 7, 2, 12 GO terms respectively (Fig. 3C). These data uncovered a spread-out domain of gene expression which supported the faded whorl specification origin of higher plants, and domestication of double flowers tends to be subversive to the canalization of gene expression.

### ABCE genes in the formation of double flowers

**Figure 4:**
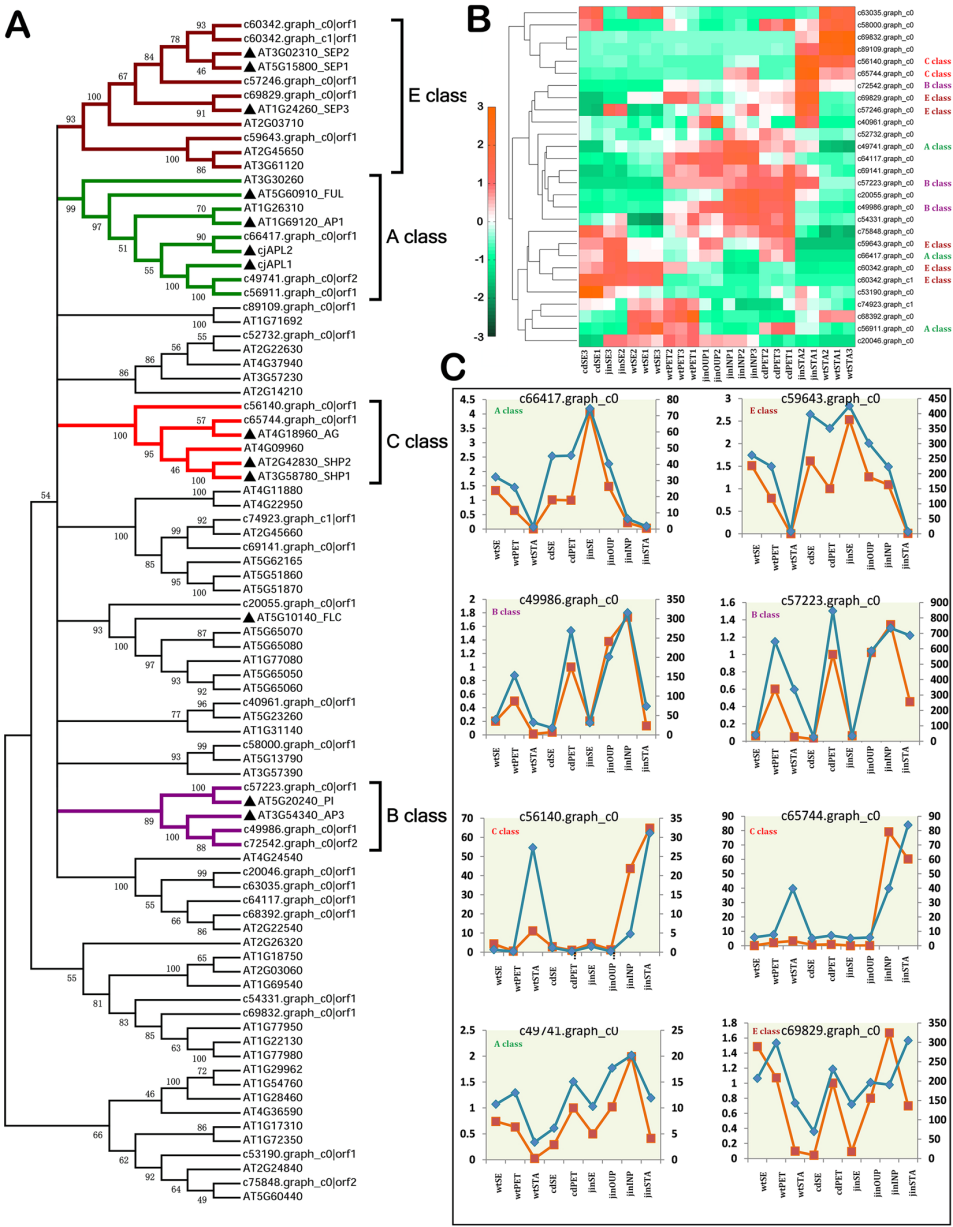
Identification and expression analyses of MADS-box genes. (**A**) The phylogenetic tree of MADS-box genes in Arabidopsis and *C. japonica*, and subgroups of functional characters were identified by annotation from Arabidopsis. Colored branch indicated a subgroup of MADS genes based on the ABCE genes in Arabidopsis. (**A**,**B**) heatmap plot of expression patterns of MADS-box genes from *C. japonica*. The genes that were classified in ABCE categories as shown in (**A**) were labeled. Expression levels of samples were normalized by z-score and plotted. (**C**) Comparisons of expression profiles of key MADS-box genes by RNAseq and qPCR analyses. In each panel, the blue line indicated values of qPCR experiments referring to the left y-axis, and the orange line indicated RPKM values of RNA-seq referring to the right y-axis.

We showed the domestication of different types of double flowers potentially deconstructed the constrains of gene expression required for the whorl development. To gain insights of transcriptional activities in double flower development, we focused on several gene families containing important floral regulators. The MADS-box transcription factors included some best characterized floral homeotic genes of ABCE model^29^. We performed sequence and phylogenetic analyses to identify homologous genes in *C. japonica* (Fig. 4A; See ‘Materials and methods’ for details). We found 28 homologs including A, B, C, E function genes and flowering timing regulators (Fig. 4A).

Previous studies showed A function genes were up-regulated in different double flower cultivars and C function gene (*CjAG*) had different expression patterns in formal and anemone double flowers^27, 28^. We found the transcriptomics results were in good agreement with previous work: A function genes (c66417. graph.c0, Fig. 4C) were up-regulated in double flower organs consistent with more petals and a lack of carpels and reduced stamens in these double flowers (Fig. 4B); AG-like (including *Plena*-like) genes were not detectable in formal double consistent with a lack of stamens or carpels in formal double. However C class gene was upregulated in inner organs of anemone double (Fig. 4B,C); this was surprising as many inner organs displayed petal-like structures^27^. We selected 31 genes and validated the expression profiles through real-time PCR experiment (Primers in Supplementary Table S5). The expression patterns of candidates were compared to the RNA-seq data. We found 21 of those profiles were significantly correlated in Q-PCR and RNA-seq, and 7 correlations were not significant (Supplementary Figure S2).

Expression of unigenes that were homologous to ABCE genes was characterized to understand the roles in double flower domestication. The A-class unigenes (c66417. graph_c0) displayed expected expression pattern in wild *Camellia*-highly expressed in sepal and petal, undetectable in stamen (Fig. 4C). In the inner petal of anemone double, unigene c59643. graph_c0 (a class E gene), not c66417. graph_c0 (a class A gene close to *CjAPL2*), was expressed abundantly (Fig. 4C). The inner petal displayed the characteristics of petal identity, but placed in the central zone (whorl 3) of floral bud^27^, and the expression pattern suggested that the E class gene might be essential to inner organ differentiation. The B- class genes had similar expression levels in petals of wild, formal double, and outer petals of anemone double, but its high expression in inner petal of anemone was also notable (Fig. 4C). The expression of ABCE homologs was predictable in wild *Camellia* suggesting the well-defined floral organ differentiation required conserved functions of those genes. However, in domesticated cultivars, the formal double tends to disrupt the deployment of homeotic regulators to achieve the petal conversion; the inner whorls of anemone double displayed obscure expression domains crossing the borders of floral organ types.

### Conserved miRNA-target regulations with functional diversifications in *Camellia* and double flowers

**Figure 5:**
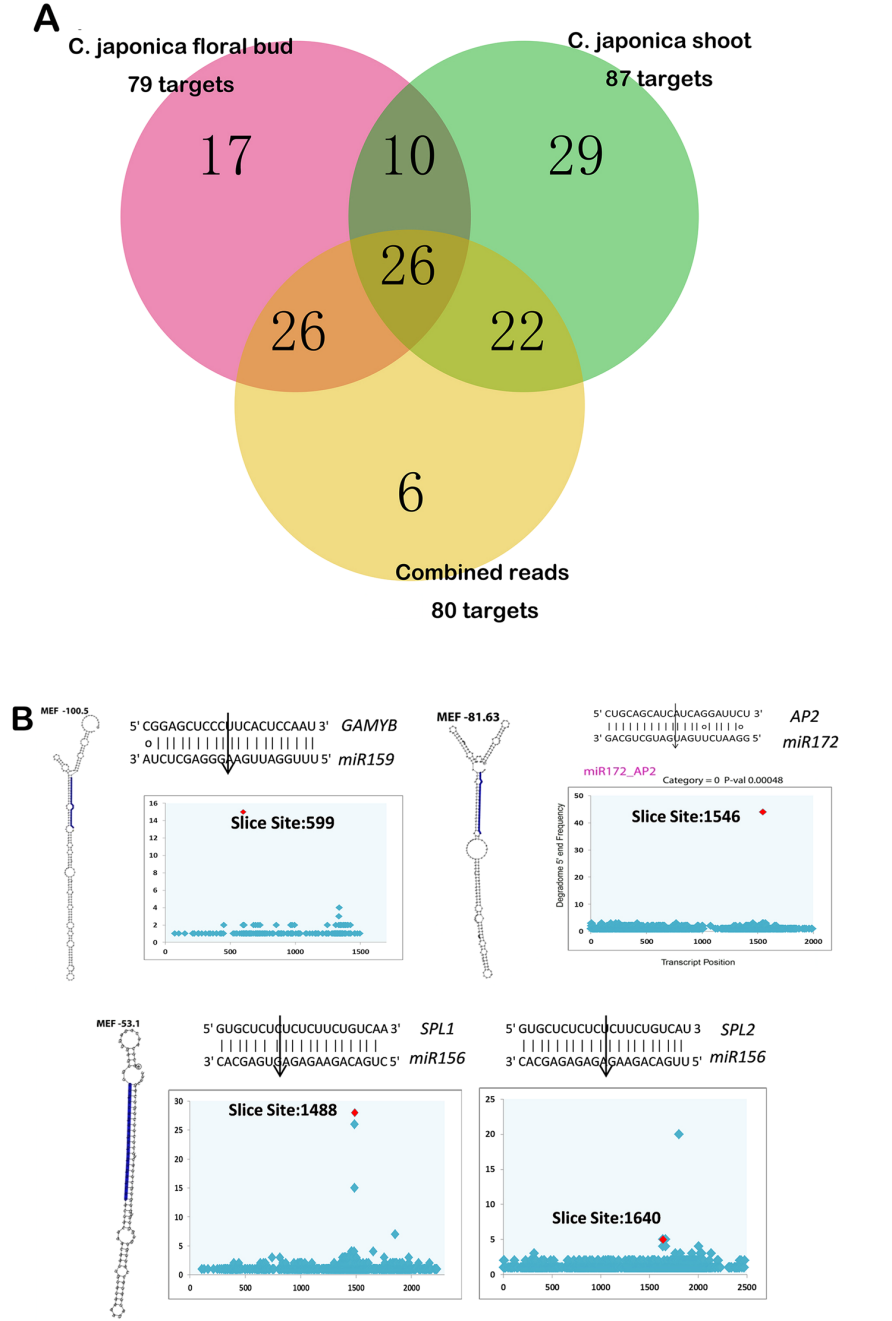
Analysis of miRNA-Targets from small RNA and degradome sequencing. (**A**) The venn plot of highly confident miRNA targets from degradome sequencing. The degradome libraries of shoot and floral bud were analyzed independently and also merged together to identify targets. (**B**), in each panel, left displayed the secondary structure of miRNA precursors and blue color highlighted the mature miRNA sequences. Right, the T-plot of key miRNA targeted slicing sites validated in the degradome sequencing. The slicing sites were indicated by arrows.

To investigate the molecular mechanism underlying the altered domain allocation, we asked whether the micro-RNA (miRNA) and target circuit may contribute to the double flower. To generate the sequence resource for miRNA and target identification, we performed small RNA sequencing in wild shoot and floral bud and compared the miRNA expression between wild type petal and inner petal and petaloid stamen of anemone double flower. 18.5~21.5 million short reads per library were obtained and short reads between 18–30 bp in length were subjected to bioinformatics pipeline for miRNA isolation using transcriptome as the reference (Supplementary Table S4). In total 127 miRNAs genes were identified with mature and precursor sequences (Supplementary Dataset 3). To further understand the roles of miRNAs, we also sequenced 2 ‘degradome’ libraries of wild type shoot buds (spring) and floral buds (fully differentiated before opening, average 1.5 cm in length) to identify the recognition targets of miRNAs. Over 80 million reads per the degradome library were generated for target identification (Supplementary Table S4). The slice sites of miRNAs were searched against the sequencing end of degradome libraries by CleaveLand software version 4.0^20, 30^, and a total of 771 potential targeted genes with annotation information were predicted (alignment score >5) (Supplementary Dataset 4). 87 and 79 highly accurate recognition sites (P-value < 0.05) were revealed in shoot and floral bud, and 80 targets were identified when combining sequencing datasets from 2 libraries for target identification (Fig. 5A, Supplementary Dataset 4).

Among these highly confident targets, we found many evolutionarily conserved miRNA-target pairs in *Camellia* (Supplementary Dataset 4). Some transcription factors including *Auxin Response Factors*, *MYB*, *Homeodomain-containing genes*, *AP2*, *SPL*, *TCP* which had important functions in plant development were identified (Supplementary Dataset 4); meanwhile some targets like galactinol synthase (c49174. graph_c0), anthocyanidin glucosyltransferase (c67697. graph_c0) were potentially newly evolved in the regulation of metabolism in *Camellia* (Supplementary Dataset 4). Hence, these results suggested that the present resource of miRNA and their targets were comprehensive for further functional evaluation in double flower development.

**Figure 6:**
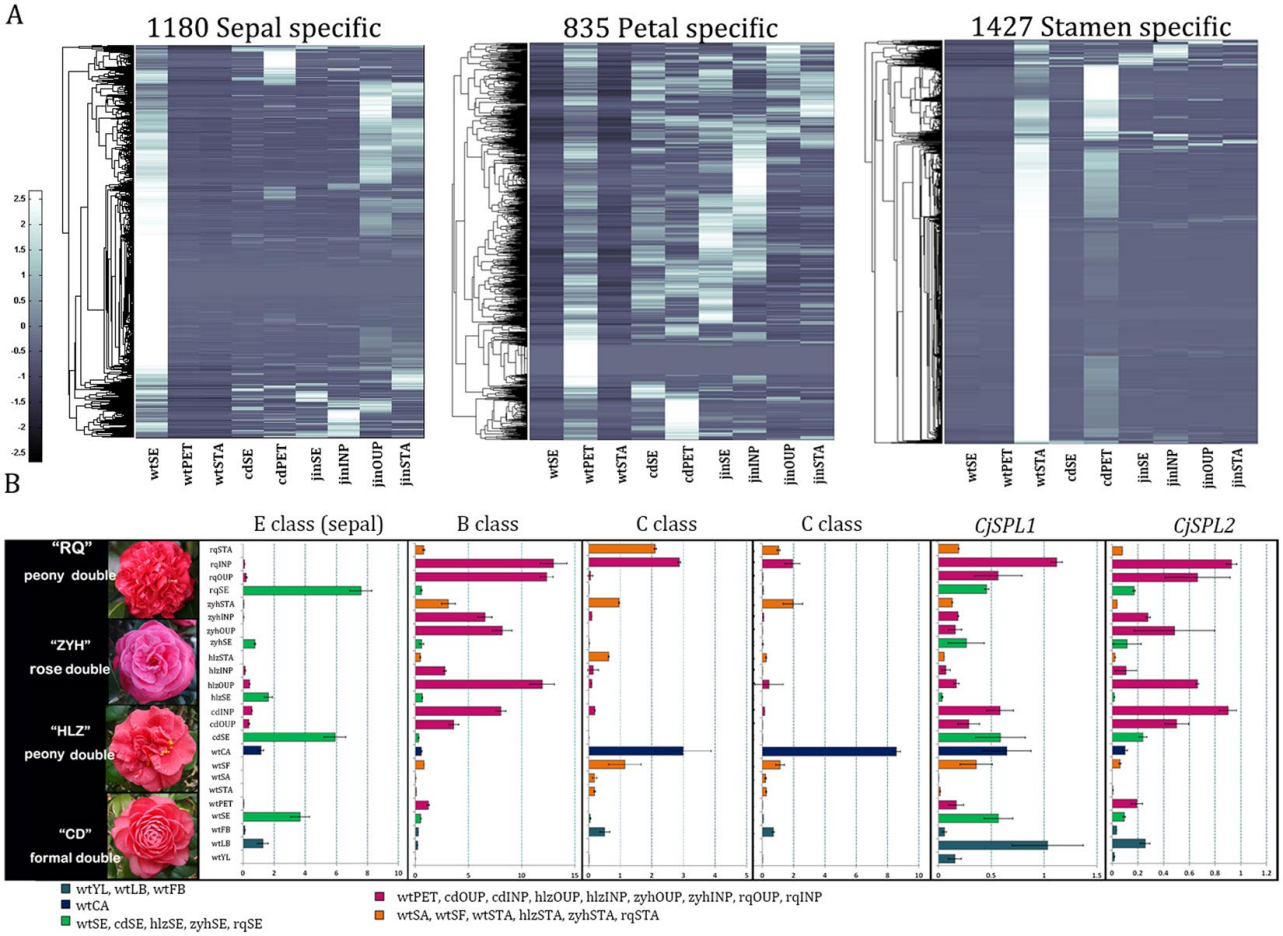
Whorl-specific genes and expression analysis of key regulators in various double flowers in *Camellia*. **(A**) Whorl specific genes displayed disrupted expression patterns in doubled flower culvars. The genes with preference in one organ of wild *Camellia* sepal, petal and stamen were identified through expression analysis. The mean expression of samples was obtained and normalized by the z score method. Normalized expression of clusters was plotted in the heatmap figure. (**B**) Expression analysis of floral regulators in different double flower cultivars. “RQ”, Rongqiu, peony double; “ZYH”, ZhuangYuanHong, rose double; “HLZ”, HongLuZhen, peony double with modified arrangements of inner petal and stamen whorls; “CD”, ChiDan, formal double. Organ types were grouped by the positional homology and indicated in different colors. wtYL, wild type young leaf; wtLB, wild type leaf bud; wtFB, wild type floral bud; wtSE, wild type sepal; wtPET, wild type petal; wtSTA, wild type stamen; wtSA, wild type anther; wtSF, wild type filaments; wtCA, wild type carpel; cdSE, ChiDan sepal; cdOUP, ChiDan petals in outer whorls; cdINP, ChiDan petals in the inner whorls; hlzSE, HongLuZhen sepal; hlzOUP, HongLuZhen outer petal; hlzINP, HongLuZhen inner petal; hlzSTA, HongLuZhen stamen; zyhSE, ZhuangYuanHong sepal; zyhOUP, ZhuangYuanHong outer petal; zyhINP, ZhuangYuanHong inner petal; zyhSTA, ZhuangYuanHong stamen; rqSE, RongQiu sepal; rqOUP, RongQiu outer petal; rqINP, RongQiu inner petal; rqSTA, RongQiu stamen.

It’s known that miR172-AP2 and miR169-NFYA regulations were critical for restricting the C-class function during floral patterning, and other regulations such as miR164-CUC, and miR156-SPL were involved in the determination of floral organ number and shape in parallel to ABC functions^14, 16, 31^. We showed that the recognition of predictive target sites was verified in degradome sequencing (Fig. 5B). In *Arabidopsis*, SPL was found to be required for anther sporogenic tissues differentiation and expressed abundantly in the anther tissues^17, 32, 33^. In *Camellia*, miR156 was expressed abundantly in stamen where *SPL1* and *SPL2* were expressed at a low level^22^. In this study, SPLs were expressed just in stamen filaments but not anther tissues (Fig. 6) suggesting the silencing by miR156. It indicated that suppression of SPLs by miR156 was a key step of inner petal and stamen-like organ formation. The expression level of SPLs in petal and stamen organs may present a distinctive molecular event for determination of double flower type.

### Collapsed expression of whorl-specific genes in different types of double flowers

We showed that distinctive gene expression is related to morphological alterations in double flowers, and post-transcriptional regulation mediated by miRNA played important roles in fine-tuning floral organ development. To further characterize the well-defined floral organs in wild *Camellia*, we identified some whorl-specific genes and analyzed the expression among the floral organs in double flowers. Using the RNA-seq abundance data of wild *Camellia*, we identified the whorl specific unigenes with more than ten-fold level in one organ than the rest expression level in other organs; we found 1180 unigenes were sepal-specific, 835 petal-specific, and 1427 stamen-specific; the whorl-specific expression tends to be diverted in double flower cultivars; hierarchical expression of whorl-specific genes revealed diverted patterns in domesticated cultivars despite highly preferential expression in specific organ of wild *Camellia* (Fig. 6A). Meanwhile, some abovementioned ABCE genes were found in the whorl specific categories, such as B and C class genes. Interestingly, one E class gene (c60342. graph_c1) was identified as a sepal-specific gene. To understand how the expression of whorl-specific genes and ABCE genes were altered in double flowers, we performed qPCR analysis in wild and 4 types of double flower cultivars including 2 peony double, 1 rose double and 1 formal double (Fig. 6B). We showed that the sepal-specific E class gene was indeed highly abundant in sepals, in wild as well as cultivated double flowers. However in peony and formal double, there is a slight induction of expression of this E gene in petals (Fig. 6A,B). The B class gene was up-regulated in all assayed double flower petals (Fig. 6B) confirming the previous study in which multiple B class copies had differential expression levels contributing to petal development^28^. The reduced expression of C class genes was expected in formal double^27^, however, low expression was detected in the inner whorls of petals–usually with deformed shapes in rose and formal doubles (Fig. 6B); in another peony double cultivar “RongQiu” (cultivar with more and enlarged inner petals in floral center), the expression of C class was evidently abundant which was similar to anemone double (Fig. 6B). Taken these results together we speculated that the domestication in *Camellia* has resulted in fundamental deconstruction of the gene expression deployment which consisted of not only ABCE function genes but also the overlapping spatial domains of present unknown factors. We showed the post-transcriptional regulation of SPL genes were specific to stamen development; specifically, the miR156 targeted silencing was required in the anther tissue formation. We found *CjSPL1/2* were expressed in whole stamens and filaments, but not detectable in anther (Fig. 6B). Detailed analysis in peony and rose double revealed that the expression levels of *CjSPL1/2* were significantly lower in inner stamenoid than inner petals (Fig. 6B). Since the reduction of SPLs expression was mediated by miR156, the expression levels SPLs could be a molecular character of types of double flowers.

## Conclusions

This work analyzed the genome-wide gene expression profiles in wild and cultivated double *Camellia*, and captured the dynamics of differential expressed genes with functional characteristics. We showed that the double flower domestication deconstructed the designated expression patterns of key components such as ABCE genes and other floral regulators, and revealed the diversity of double flower types relied on the faded whorl specification mechanisms. Finally, through small RNA and degradome sequencing, we identified the conserved miRNA-target regulations in *Camellia*; detailed expression comparisons indicated that key regulatory pairs, such as miR172-AP2 and miR156-SPLs, had divergent functions in *Camellia* floral development, and were central nodes of directing diverse inner organ growth in different double flowers.

## Methods

### Plant materials and growth conditions

*Camellia* plants were grown in the greenhouse of Research Institute of Subtropical Forestry (Fuyang, Zhejiang, China) under natural light. For RNA extraction, different floral organs from wild and cultivated camellias (floral bud around 1.5–2 cm, before opening) were collected and frozen immediately in liquid nitrogen and stored in −80 °C freezers before use. Three biological replicates of all samples were collected from at least three individuals during the flowering seasons of year 2014 and 2015.

### RNA extraction and quality assessment

Total RNAs were extracted using the PLANTpure kit (Cat. RN33, Aidlab, Beijing, China) and stored in freezer before use. For QPCR analysis, cDNA was synthesized by a PrimeScriptII 1st Strand cDNA Synthesis Kit (Cat. 6210 A, Takara, Dalian, China) supplied with DNase treatment columns to avoid DNA contamination. For next-generation sequencing experiments, the RNA quality and quantity was determined using a Nanodrop 1000 spectrophotometer (Thermo Fisher Scientific, Wilmington, DE) and a Bioanalyzer RNA nano chip (Agilent Technologies, Singapore). The quality and quantity of RNAs were assessed by OD 260/280 and 260/230 and RNA integration number (RIN) to meet the standards for library construction of transcriptome, small RNA and degradome sequencing as described^21, 34^.

### Transcriptome, small RNA and degradome sequencing and data processing

Approximately, 5 ug of total RNA for each tissue sample was used for the construction of libraries using mRNA-Seq Sample Prep kit (Illumina Inc., San Diego, CA) according to the manufacturer’s protocol. Equal quantities of libraries (approximately 5 ng per sample) with different indices were mixed and stored in −80 °C freezer before sequencing. Sequencing was performed in a v3 flowcell on an Illumina HiSeq 2500 sequencer by Biomarker Technologies (Beijing, China), generating 2 × 101 bp and 1 × 60 bp reads. About 120 million high quality RNA-Seq reads (with quality score >20 for each base) were pooled from Illumina sequencing of each of the 9 samples (three biological replicates of 3 stages) and were then assembled into contigs using Trinity^35^. All sequencing data were deposited in NCBI Short Read Archive under BioProject ID PRJNA331772. We quantified transcript levels in reads per kilobase of exon mode per million mapped reads (RPKM)^36^. Small RNA libraries were constructed as previously described and sequenced by a Hiseq 2500 machine generating 1 × 51 bp reads. The ‘degradome’ sequencing libraries were constructed as described and sequenced by a Hiseq 2500 as described before (Biomarker technologies, Beijing, China). All sequencing reads were trimmed to yield clean reads for downstream analysis, and detailed parameters for trimming were as previously described in the^37^. All sequencing data were deposited in NCBI Short Read Archive under BioProject ID PRJNA331772.

### Identification of miRNA and targets

For each small RNA sequencing library, the clean reads range from 18–30 bp were obtained and then filtered through RNA databases before miRNA identification^22^. The transcriptome assemble from *C. japonica* was used as reference for precursors identification. The unannotated reads were undergone the miRDP package pipeline to search for miRNAs^38^. The core miRDeep output figures were generated for each miRNA and accessed. The precursors and mature sequences were aligned in PNRD database for annotation of miRNAs^39^. To quantify the abundance of miRNA, the TPM value was defined as ‘counts of reads mapped to miRNA × 1,000 000’/‘reads mapped to the reference genome’^40^.

The sequencing reads from degradom library were obtained for target site identification. The CleaveLand4.0 pipeline was used for target scanning^30^. The reads were mapped to *C. japonica* transcriptome and the alignment scores and p-value were calculated according the signatures (abundances of potential slicing end based on reads distributions). The t-plots were generated to visualize the miRNA directed slicing to targets. The alignment score over 5 was used to select potential miRNA targets; and p-value less than 0.05 was used to identify highly confident targets. The 2 degradome libraries were run through the target identification process separately, and the merged data combing the 2 libraries went through for the target identification as well.

### DEG identification and clustering and GO enrichment analysis

The RPKM values were used to calculate the significance of gene expression changes. The differential expression analysis was carried out by using the Bioconductor package package EdgeR^41^. The DEGs were identified by the thresholds of log2 (RPKM) ratio > = 1, P-value < 0.05, false discovery rate <0.001. The ks statistic test (Kolmogorov-Smirnov test) was performed to identify the enrichment significance of GO term in DEG datasets^42^; p-value correction was performed using the Benjamini-Hochberg (BH) method. The GO terms with p-values less than 0.05 were considered significantly enriched. The enriched GO terms from each comparison were consolidated into a matrix, and the distance between comparisons was calculated by the furthest neighbor distance and clustered. The Hierarchical clustering of gene expression was performed by clustergram function in Matlab Bioinformatics toolbox with minor changes of appearance.

### Phylogenetic analysis

The MADS-box genes were searched by using *Arabidopsis* genes sequences. The Matlab script of reciprocal blast against two protein databases (RBH) was used to find orthologs of MADS-box sequences in *C. japonica* and *Arabidopsis*. The phylogenetic tree was built by MEGA5 using the Neighbor Joining Tree method with minor modifications^43^.

### Quantitative PCR analysis

To validate the gene expression of RNA-seq, Real time quantitative PCR was performed on a QuantStudio 7 Flex Real-Time PCR System (Applied Biosystems, USA). The regular ΔΔct method was used by comparing target gene and the internal reference gene (Glyceraldehyde 3-phosphate dehydrogenase, GAPDH)^44^. All amplifications were performed 3 times as technical replicates, and 3 biological replicates were used for each experiment. The SYBR Premix Ex Taq II kits (Cat. RR820a, Takara, Dalian, China) were used to perform the real-time PCR analyses. The relative expression values were calculated by using the default software of the QuantStudio 7 system. The primers used in the study were listed in Supplementary Table S5.

### Availability of data and materials

All relevant data are published within the paper and its supporting additional files.

## Electronic supplementary material

Supplementary Information
