# Supplementary material for: Global gene expression defines faded whorl specification of double flower domestication in Camellia
Source: Sci Rep. 2017 Jun 9;7:3197. doi: 10.1038/s41598-017-03575-2 (PMC5466612; doi:10.1038/s41598-017-03575-2)
Supplement: Supplementary file 1 — Supplementary Information [file 41598_2017_3575_MOESM1_ESM.doc]

**Global gene expression defines faded whorl specification of double flower domestication in *Camellia***

Xinlei Li ^1,2^, Jiyuan Li ^1,2^, Zhengqi Fan ^1,2^, Zhongchi Liu ^3^, Takayuki Tanaka ^4^, Hengfu Yin ^1,2,*^

^1^ Research Institute of Subtropical Forestry, Chinese Academy of Forestry, Fuyang, Zhejiang 311400, China

^2^ Key Laboratory of Forest genetics and breeding, Chinese Academy of Forestry, Fuyang, Zhejiang 311400, China

^3^ Departments of Cell Biology and Molecular Genetics, University of Maryland, College Park, MD, USA

^4^ Department of Plant Science, School of Agriculture, Tokai University, Minami-aso-mura, Aso-gun, Kumamoto 869-1404, Japan

^*^ Corresponding author Email: hfyin@caf.ac.cn

**Supplementary Information**

This file contains 2 supplementary figures, 5 supplementary tables and 4 supplementary datasets. Captions and legends are also included.

**Supplementary Tables**

**S1. The summary of assembly statistics in *C. japonica*.** The distribution of assembled unigenes were listed according to length. The total 104,810 unigenes were obtained with a N50 of 893bp.

| **All_Combination.Unigenes Length** | **Total Number** | **Percentage** |
| --- | --- | --- |
| **200-300** | 45103 | 43.03% |
| **300-500** | 27598 | 26.33% |
| **500-1000** | 16988 | 16.21% |
| **1000-2000** | 9763 | 9.31% |
| **2000+** | 5358 | 5.11% |
| **Total Number** | 104810 |  |
| **Total Length** | 62308438 |  |
| **N50 Length** | 893 |  |
| **Mean Length** | 594.5 |  |

**S2. The summary of annotation statistics of *C. japonica* transcriptome.** The assembled unigenes were searched in various databases for annotation. The databases were listed below. Nr, the NCBI non-redundant protein database; GO, Gene Ontology; KEGG, the Kyoto Encyclopedia of Genes and Genomes; KOG, EuKaryotic Orthologous Groups; COG, Cluster of Orthologous Groups of proteins database; Pfam, http://pfam.xfam.org/; Swissprot, www.uniprot.org. In total, 37384 unigenes were found and annotated in at least one database.

| **#Anno_Database** | **Annotated_Number** | **300<=length<1000** | **length>=1000** |
| --- | --- | --- | --- |
| **COG_Annotation** | 9270 | 2698 | 5490 |
| **GO_Annotation** | 20284 | 7936 | 8211 |
| **KEGG_Annotation** | 6603 | 2439 | 3099 |
| **KOG_Annotation** | 19862 | 7728 | 8289 |
| **Pfam_Annotation** | 21222 | 7395 | 11214 |
| **Swissprot_Annotation** | 24300 | 9737 | 10335 |
| **nr_Annotation** | 36992 | 15559 | 13527 |
| **All_Annotated** | 37384 | 15716 | 13549 |

**S3. Transcriptome sequencing statistics in different floral organs of wild and double flower *Camellias*.** In total, 9 floral tissue types from wild Camellia and formal double and anemone double cultivars were collected for illumina sequencing with 3 biological replicates for each tissue type. The sequencing statistics of 27 libraries were listed.

| **Samples** | **Sample description** | **Read Number** | **Base Number** | **GC Content** | **%≥Q30** |
| --- | --- | --- | --- | --- | --- |
| **wtPET-1** | Wild petal | 14,016,839 | 714,740,452 | 45.04% | 91.35% |
| **wtPET-2** | Wild petal | 12,935,950 | 659,626,734 | 44.88% | 91.37% |
| **cj Pet-3** | Wild petal | 12,292,550 | 626,809,016 | 44.50% | 91.07% |
| **cj se-1** | Wild sepal | 13,700,007 | 698,580,314 | 44.42% | 94.67% |
| **cj se-2** | Wild sepal | 13,065,215 | 666,210,157 | 44.71% | 94.66% |
| **cj se-3** | Wild sepal | 10,887,668 | 555,184,330 | 44.80% | 91.41% |
| **cj Sta-1** | Wild stamen | 9,966,892 | 508,231,173 | 44.92% | 91.50% |
| **cj Sta-2** | Wild stamen | 10,297,173 | 525,071,352 | 44.84% | 91.59% |
| **cj Sta-3** | Wild stamen | 10,413,473 | 531,004,029 | 44.97% | 91.56% |
| **Jin Outp-1** | Anemone Outer petal | 14,776,321 | 753,471,316 | 45.26% | 94.57% |
| **Jin Oupt-2** | Anemone Outer petal | 11,169,688 | 569,560,970 | 44.83% | 94.56% |
| **Jin Oupt-3** | Anemone Outer petal | 14,781,497 | 753,726,143 | 44.97% | 94.50% |
| **Jin sta-1** | Anemone petaloid stamen | 16,030,340 | 817,411,676 | 44.72% | 94.73% |
| **Jin sta-2** | Anemone petaloid stamen | 13,368,152 | 681,664,115 | 44.50% | 94.70% |
| **Jin sta-3** | Anemone petaloid stamen | 13,026,979 | 664,269,470 | 45.14% | 94.62% |
| **Jin Inp-1** | Anemone Inner petal | 9,722,658 | 495,785,988 | 45.11% | 91.28% |
| **Jin Inp-2** | Anemone Inner petal | 10,753,993 | 548,373,695 | 44.51% | 91.41% |
| **Jin Inp-3** | Anemone Inner petal | 10,535,882 | 537,251,195 | 44.21% | 91.73% |
| **Jin Se1** | Anemone sepal | 12,313,511 | 627,891,921 | 44.64% | 91.36% |
| **Jin se-2** | Anemone sepal | 11,754,373 | 599,380,888 | 44.34% | 91.54% |
| **Jin se-2** | Anemone sepal | 11,774,154 | 600,388,192 | 44.39% | 91.53% |
| **ChiD Pet-1** | Formal petal | 11,665,216 | 594,837,353 | 44.30% | 91.64% |
| **ChiD pet-2** | Formal petal | 12,208,531 | 622,542,836 | 44.68% | 91.58% |
| **ChiD pet-3** | Formal petal | 9,556,629 | 487,315,895 | 44.87% | 91.56% |
| **ChiD Se1** | Formal sepal | 13,821,501 | 704,771,344 | 44.63% | 94.72% |
| **ChiD se-2** | Formal sepal | 14,544,893 | 741,661,700 | 44.20% | 94.72% |
| **ChiD Se3** | Formal sepal | 13,346,485 | 680,517,729 | 44.57% | 94.28% |

**S4. The sequencing statistics of small RNA and degradome libraries. In total, 4 floral tissue types from wild Camellia and anemone double cultivars were collected small RNA sequencing. Two degradome libraries were constructed in wild Camellia using shoot and floral bud tissues, and each library yielded over 80million reads for target identification.**

| **Sample ID** | **Library type** | **Raw reads** | **Q30(%)** | **Clean reads**  **(18-30nt)** |
| --- | --- | --- | --- | --- |
| **Wild shoot** | **small RNA** | **21,523,346** | **94.03** | **15,362,009** |
| **Wild FB** | **small RNA** | **18,497,670** | **93.81** | **12,318,369** |
| **Jin inP** | **small RNA** | **20,800,346** | **94.04** | **15,005,235** |
| **Jin inS** | **small RNA** | **19,458,741** | **93.91** | **14,564,060** |
|  |  |  |  |  |
| **Sample ID** | **Library type** | **Raw reads** | **Clean reads** | **Q30(%)** |
| **Wild shoot** | **degradome** | **83,266,766** | **83,164,764** | **96.75** |
| **Wild FB** | **degradome** | **87,448,721** | **87,252,511** | **96.52** |

**S5. The sequences of primers used in this study.**

| **Primer ID** | **Sequence 5'-3'** | **bp** |
| --- | --- | --- |
| c1701c0—F | CACATGCATCAACAGAACATAGAGTT | 26 |
| c1701c0—R | TTCGAAGAAGAAAGGCTGGATT | 22 |
| c19879c0—F | GATCAAACGGATCGAAAACACA | 22 |
| c19879c0—R | GCGGCGCTTGCAGAAG | 16 |
| c20046c0—F | TCTGTTTCAACCACACATTCTCTTC | 25 |
| c20046c0—R | AATCACCGCCCGTAAGACAA | 20 |
| c4106c0—F | GGAGTCAATGAACCGTGTCATG | 22 |
| c4106c0—R | CTGCATATCAAATGTGGACAAATG | 24 |
| c43387c0—F | AACAAGAAGCTGCCAAATTGC | 21 |
| c43387c0—R | GCATGTGCCTGTTTGAATTCTG | 22 |
| c43639c0—F | CGCTGATGATATCCATTTTCCA | 22 |
| c43639c0—R | TTGCATCAGCAAGAGATGAACA | 22 |
| c44492c0—F | CAAGAAAAGGAAGAAGCACTCTGA | 24 |
| c44492c0—R | CGACAGGACAAGGGTGGAA | 19 |
| c49741c0—F | GGAAACAAGCAGAGGAGAAACAC | 23 |
| c49741c0—R | ATGCTTGCACACCTCTCGAA | 20 |
| c49986c0—F | CCGTTTCGTCGCTTGGAGTA | 20 |
| c49986c0—R | CAGATCAAGCGGATCGAGAAC | 21 |
| c53235c0—F | GCACCCTTTTATGTCACTCGAAT | 23 |
| c53235c0—R | CCATCATCAAAGGAAAATGCATT | 23 |
| c54198c0—F | CTTGAACGCTACCGGACTCATA | 22 |
| c54198c0—R | CAGGTTGATGATCGTTGCTGTT | 22 |
| c54331c0—F | CCGCCACGTGCCAAA | 15 |
| c54331c0—R | CCCACCTAAGCCTTCATTTCC | 21 |
| c56140c0—F | ACTGTTGTTGGCGTACTCGTAAAG | 24 |
| c56140c0—R | CGAAGTCGCCCTTATCGTCTT | 21 |
| c57223c0—F | GGAAAGATGCTGGAGGAGGAT | 21 |
| c57223c0—R | TCTCCATCTCTTGCTGATGCA | 21 |
| c58000c0—F | CCAGCACTGGAAAACTCGAAA | 21 |
| c58000c0—R | TGCCGAAGTTGCGGTTATTAT | 21 |
| c59643c0—F | GCCGCGGCAAGCTCTATA | 18 |
| c59643c0—R | CCTCTCCAGGGTTTTGGTCAT | 21 |
| c60342c1—F | TGCTCAAGCTCCTTTGTGGTT | 21 |
| c60342c1—R | CAATCTCAGAGAAACCTGCTTGGT | 24 |
| c62465c0—F | CCTCTTAGAAACCATGAACCCTAGAT | 26 |
| c62465c0—R | GCCGCGCCATTCTTGTC | 17 |
| c63035c0—F | CCAACAATGGCAGCACAGTT | 20 |
| c63035c0—R | TTGAGGGAAGTATCAGAGCTGTCA | 24 |
| c64117c0—F | TGAGCTAGCATAATCGAAGAGCTTT | 25 |
| c64117c0—R | CGATGCTGATGTTGCTCTCATC | 22 |
| c65744c0—F | CCGTTTCGACGCTTGCA | 17 |
| c65744c0—R | TCAAGCGGATCGAAAACACTAC | 22 |
| c66417c0—F | GCAACTTCAGCATCACACAAAAC | 23 |
| c66417c0—R | AAGAGGAGAGGTGGGCTTCTG | 21 |
| c66854c0—F | CCAGGACCGAAGTAACCAGAGT | 22 |
| c66854c0—R | GAGATGTGGAGTGCTCGACTGA | 22 |
| c68392c0—F | CCAAGAGACGAAAAGGGCTTT | 21 |
| c68392c0—R | ACGTCAGCATCGCAGAGAACT | 21 |
| c68724c0—F | AGGTCGTAAGGCGGTTGGT | 19 |
| c68724c0—R | GGGCGGGTCAAGTTGAAGAT | 20 |
| c69141c0—F | TCCGTCTCAACATCCGAAAAT | 21 |
| c69141c0—R | TGCCAATTCCGCTGCTAGTA | 20 |
| c69829c0—F | GCTGCCTTTCCAGTGACTCAA | 21 |
| c69829c0—R | CCTCCTGGGTGAGGATCTTG | 20 |
| c69832c0—F | CACCTCCGGGCAAGTTGA | 18 |
| c69832c0—R | GCTGCGGAGCAACATCATC | 19 |
| c75848c0—F | AACACCTCTTCCACCCCTTTC | 21 |
| c75848c0—R | AGATGGGTGGACGTACTGGTTT | 22 |
| c77784c0—F | TGCTCCGGCGTAGGATTC | 18 |
| c77784c0—R | GGAGTTCAATCCACACCAAGTTG | 23 |
| c89109c0—F | GCCCTTGCATGGTTCCTTTA | 20 |
| c89109c0—R | GTTCTCATTTTCTCTGCCAATGG | 23 |

**Supplementary Figures**

**Supplementary Figure S1. Sample correlations between samples of RNA-seq.** Red indicates high correlations; green indicates low correlations. Cj, wild Camellia; ChiD, ChiDan; Jin, Jinpanlizhi; Se, Sepal, Pet, Petal, sta, Stamen; OutP, outer petal; InP, inner petal. Each highlighted rectangular included one sample that was not highly correlated with other two replicates.

**
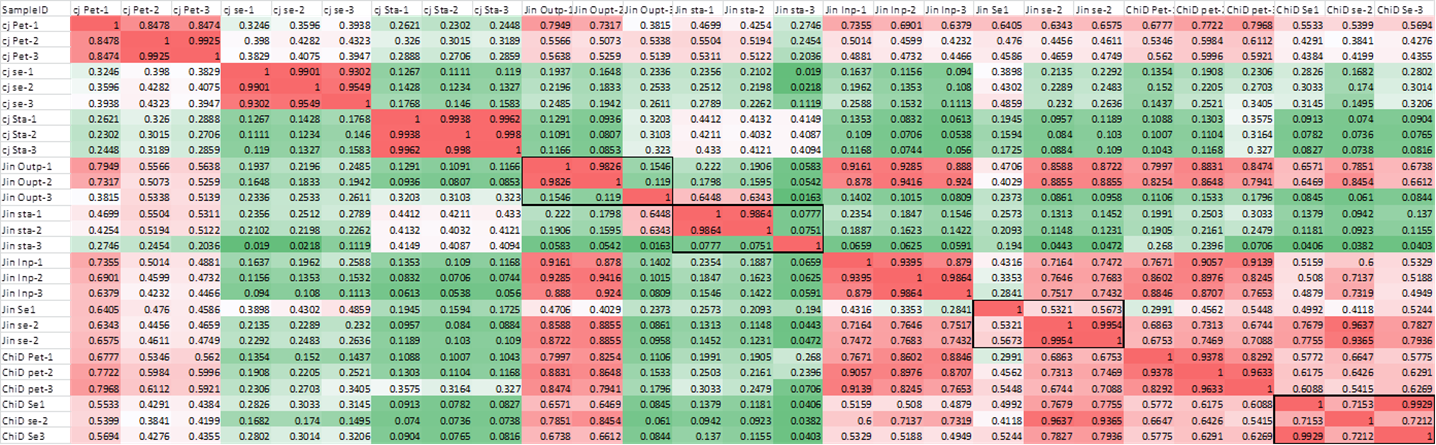
**

**Supplementary Figure S2. Correlation analysis of qPCR and RNA-seq of gene expression patterns.** The yellow line indicates significant threshold of positive correlations. The original gene names were on the bottom.

**
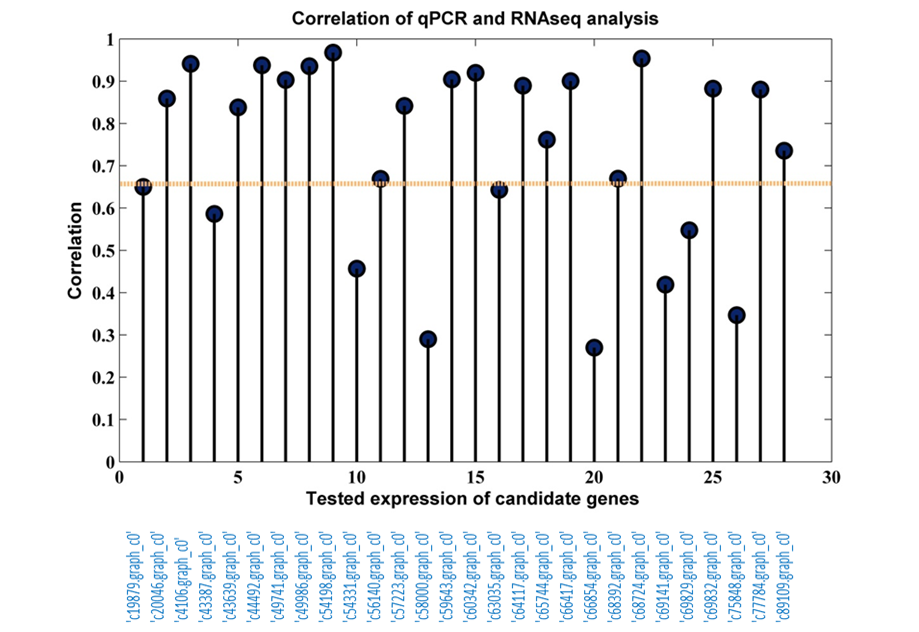
**

**Supplementary Dataset 1.** Enriched GO terms of each comparison of differentially expressed genes.

| **GO description(wtSE-wtP)** | **GO term** | **P value** |
| --- | --- | --- |
| oxidation-reduction process | GO:0055114 | 1.8E-15 |
| chitin catabolic process | GO:0006032 | 3.7E-10 |
| DNA integration | GO:0015074 | 3.3E-07 |
| response to red light | GO:0010114 | 2.8E-06 |
| cell wall macromolecule catabolic process | GO:0016998 | 0.000003 |
| defense response to fungus | GO:0050832 | 5.8E-06 |
| cysteine biosynthetic process | GO:0019344 | 0.000026 |
| carbohydrate metabolic process | GO:0005975 | 0.000057 |
| photosynthetic electron transport in photosystem I | GO:0009773 | 0.000063 |
| defense response to bacterium | GO:0042742 | 0.00007 |
| glutamate metabolic process | GO:0006536 | 0.000072 |
| negative regulation of catalytic activity | GO:0043086 | 0.00013 |
| RNA-dependent DNA replication | GO:0006278 | 0.00016 |
| single-organism transport | GO:0044765 | 0.0003 |
| chlorophyll biosynthetic process | GO:0015995 | 0.00032 |
| lignin biosynthetic process | GO:0009809 | 0.00033 |
| activation of MAPKK activity | GO:0000186 | 0.0005 |
| carboxylic acid metabolic process | GO:0019752 | 0.0007 |
| regulation of protein dephosphorylation | GO:0035304 | 0.00079 |
| oxylipin biosynthetic process | GO:0031408 | 0.00085 |
| nitrate assimilation | GO:0042128 | 0.00123 |
| response to hormone | GO:0009725 | 0.00158 |
| response to arsenic-containing substance | GO:0046685 | 0.00159 |
| response to far red light | GO:0010218 | 0.00161 |
| photosynthesis, light harvesting | GO:0009765 | 0.00162 |
| pentose-phosphate shunt | GO:0006098 | 0.00165 |
| photosystem II assembly | GO:0010207 | 0.00171 |
| reductive pentose-phosphate cycle | GO:0019253 | 0.00225 |
| glycerol metabolic process | GO:0006071 | 0.00252 |
| response to karrikin | GO:0080167 | 0.0029 |
| organonitrogen compound biosynthetic process | GO:1901566 | 0.00305 |
| cellular ion homeostasis | GO:0006873 | 0.00311 |
| anthocyanin-containing compound biosynthetic process | GO:0009718 | 0.00358 |
| response to blue light | GO:0009637 | 0.00384 |
| anion homeostasis | GO:0055081 | 0.00384 |
| intracellular protein transport | GO:0006886 | 0.00413 |
| transmembrane receptor protein tyrosine kinase signaling pathway | GO:0007169 | 0.00416 |
| pectin catabolic process | GO:0045490 | 0.00461 |
| response to water deprivation | GO:0009414 | 0.00519 |
| detection of biotic stimulus | GO:0009595 | 0.00561 |
| pigment metabolic process | GO:0042440 | 0.00577 |
| plant-type cell wall biogenesis | GO:0009832 | 0.00584 |
| phosphorylation | GO:0016310 | 0.00585 |
| hydrogen peroxide transmembrane transport | GO:0080170 | 0.00601 |
| peptidyl-histidine phosphorylation | GO:0018106 | 0.00612 |
| defense response by callose deposition | GO:0052542 | 0.00618 |
| salicylic acid biosynthetic process | GO:0009697 | 0.0066 |
| potassium ion transport | GO:0006813 | 0.00675 |
| cytoplasmic transport | GO:0016482 | 0.00697 |
| signal transduction by phosphorylation | GO:0023014 | 0.00733 |
| galactose transport | GO:0015757 | 0.00738 |
| carbohydrate derivative biosynthetic process | GO:1901137 | 0.00746 |
| systemic acquired resistance, salicylic acid mediated signaling pathway | GO:0009862 | 0.00807 |
| regulation of plant-type hypersensitive response | GO:0010363 | 0.00837 |
| response to nematode | GO:0009624 | 0.00841 |
| cellular response to hydrogen peroxide | GO:0070301 | 0.00959 |
| L-phenylalanine catabolic process | GO:0006559 | 0.00985 |
| translation | GO:0006412 | 0.01007 |
| jasmonic acid mediated signaling pathway | GO:0009867 | 0.01042 |
| lipid metabolic process | GO:0006629 | 0.01057 |
| carotenoid biosynthetic process | GO:0016117 | 0.01057 |
| amino acid transmembrane transport | GO:0003333 | 0.0106 |
| regulation of flavonoid biosynthetic process | GO:0009962 | 0.0108 |
| glucosinolate metabolic process | GO:0019760 | 0.01107 |
| cellular glucan metabolic process | GO:0006073 | 0.01149 |
| response to drug | GO:0042493 | 0.01164 |
| flavonoid biosynthetic process | GO:0009813 | 0.01167 |
| response to abscisic acid | GO:0009737 | 0.01239 |
| CVT pathway | GO:0032258 | 0.01362 |
| transition metal ion transport | GO:0000041 | 0.01392 |
| water transport | GO:0006833 | 0.01413 |
| response to cytokinin | GO:0009735 | 0.01429 |
| hydrogen peroxide catabolic process | GO:0042744 | 0.01431 |
| carboxylic acid transport | GO:0046942 | 0.01465 |
| drug transmembrane transport | GO:0006855 | 0.01467 |
| cellular lipid metabolic process | GO:0044255 | 0.01536 |
| cellular protein metabolic process | GO:0044267 | 0.01557 |
| cellular cation homeostasis | GO:0030003 | 0.01567 |
| lipid biosynthetic process | GO:0008610 | 0.01595 |
| inflorescence development | GO:0010229 | 0.01639 |
| cinnamic acid biosynthetic process | GO:0009800 | 0.01672 |
| carboxylic acid biosynthetic process | GO:0046394 | 0.01691 |
| rRNA processing | GO:0006364 | 0.01781 |
| urea transmembrane transport | GO:0071918 | 0.01801 |
| oligosaccharide biosynthetic process | GO:0009312 | 0.01808 |
| ribosome biogenesis | GO:0042254 | 0.01848 |
| cellular response to iron ion starvation | GO:0010106 | 0.0192 |
| small molecule biosynthetic process | GO:0044283 | 0.01934 |
| positive regulation of flavonoid biosynthetic process | GO:0009963 | 0.02079 |
| syncytium formation | GO:0006949 | 0.02129 |
| vitamin B6 biosynthetic process | GO:0042819 | 0.02187 |
| neutral amino acid transport | GO:0015804 | 0.02218 |
| protein phosphorylation | GO:0006468 | 0.02221 |
| glucosinolate biosynthetic process | GO:0019761 | 0.0228 |
| chlorophyll metabolic process | GO:0015994 | 0.02296 |
| potassium ion transmembrane transport | GO:0071805 | 0.02397 |
| calcium ion homeostasis | GO:0055074 | 0.02438 |
| protein metabolic process | GO:0019538 | 0.02498 |
| response to cadmium ion | GO:0046686 | 0.02667 |
| response to abiotic stimulus | GO:0009628 | 0.027 |
| response to jasmonic acid | GO:0009753 | 0.02707 |
| phospholipid biosynthetic process | GO:0008654 | 0.02728 |
| developmental growth | GO:0048589 | 0.02729 |
| unsaturated fatty acid biosynthetic process | GO:0006636 | 0.02777 |
| ionotropic glutamate receptor signaling pathway | GO:0035235 | 0.02826 |
| glutamate biosynthetic process | GO:0006537 | 0.02852 |
| ion transport | GO:0006811 | 0.0289 |
| polyamine catabolic process | GO:0006598 | 0.02939 |
| asparaginyl-tRNA aminoacylation | GO:0006421 | 0.02942 |
| protein targeting | GO:0006605 | 0.03006 |
| oligosaccharide metabolic process | GO:0009311 | 0.0303 |
| cell wall macromolecule metabolic process | GO:0044036 | 0.03057 |
| xylem development | GO:0010089 | 0.03065 |
| cellular amino acid metabolic process | GO:0006520 | 0.03153 |
| sulfur compound metabolic process | GO:0006790 | 0.03241 |
| isoprenoid biosynthetic process | GO:0008299 | 0.03243 |
| cellular response to abscisic acid stimulus | GO:0071215 | 0.03279 |
| positive regulation of abscisic acid-activated signaling pathway | GO:0009789 | 0.03371 |
| proline transport | GO:0015824 | 0.03435 |
| cell wall organization | GO:0071555 | 0.03579 |
| coenzyme A metabolic process | GO:0015936 | 0.03607 |
| flavonol biosynthetic process | GO:0051555 | 0.03626 |
| monocarboxylic acid metabolic process | GO:0032787 | 0.03668 |
| metal ion transport | GO:0030001 | 0.03755 |
| response to wounding | GO:0009611 | 0.03763 |
| regulation of stomatal movement | GO:0010119 | 0.03814 |
| response to biotic stimulus | GO:0009607 | 0.03995 |
| response to nitrate | GO:0010167 | 0.0403 |
| stomatal movement | GO:0010118 | 0.04057 |
| transmembrane transport | GO:0055085 | 0.04062 |
| tryptophan metabolic process | GO:0006568 | 0.04082 |
| hyperosmotic salinity response | GO:0042538 | 0.04113 |
| plant-type hypersensitive response | GO:0009626 | 0.04208 |
| pigment biosynthetic process | GO:0046148 | 0.04293 |
| MAPK cascade | GO:0000165 | 0.04398 |
| macromolecule catabolic process | GO:0009057 | 0.04412 |
| ncRNA metabolic process | GO:0034660 | 0.04414 |
| response to oxidative stress | GO:0006979 | 0.04466 |
| response to metal ion | GO:0010038 | 0.04553 |
| endoplasmic reticulum unfolded protein response | GO:0030968 | 0.04639 |
| iron ion transport | GO:0006826 | 0.0467 |
| negative regulation of programmed cell death | GO:0043069 | 0.04715 |
| abscisic acid-activated signaling pathway | GO:0009738 | 0.0485 |
| organic substance transport | GO:0071702 | 0.0485 |
| negative regulation of iron ion transport | GO:0034757 | 0.04856 |
| cellular response to cold | GO:0070417 | 0.04856 |
| ER-nucleus signaling pathway | GO:0006984 | 0.04899 |
| cation transport | GO:0006812 | 0.04933 |
| GO description (wtSE-wtST) | GO term | P value |
| oxidation-reduction process | GO:0055114 | 1.2E-12 |
| photosynthesis, light harvesting | GO:0009765 | 1.3E-12 |
| photosystem II assembly | GO:0010207 | 9.9E-10 |
| plant-type cell wall modification | GO:0009827 | 2.3E-09 |
| isopentenyl diphosphate biosynthetic process, methylerythritol 4-phosphate pathway | GO:0019288 | 3.6E-09 |
| cysteine biosynthetic process | GO:0019344 | 0.000000018 |
| response to karrikin | GO:0080167 | 0.00000002 |
| chloroplast relocation | GO:0009902 | 0.000000022 |
| chlorophyll biosynthetic process | GO:0015995 | 0.00000023 |
| response to red light | GO:0010114 | 0.00000033 |
| rRNA processing | GO:0006364 | 0.00000037 |
| pentose-phosphate shunt | GO:0006098 | 0.00000039 |
| regulation of protein dephosphorylation | GO:0035304 | 0.00000044 |
| transcription from plastid promoter | GO:0042793 | 0.00000052 |
| L-phenylalanine catabolic process | GO:0006559 | 0.00000095 |
| thylakoid membrane organization | GO:0010027 | 0.0000013 |
| carotenoid biosynthetic process | GO:0016117 | 0.0000015 |
| cinnamic acid biosynthetic process | GO:0009800 | 0.0000029 |
| photosynthetic electron transport in photosystem I | GO:0009773 | 0.0000047 |
| chitin catabolic process | GO:0006032 | 0.000012 |
| L-serine biosynthetic process | GO:0006564 | 0.000014 |
| response to far red light | GO:0010218 | 0.000016 |
| plastid organization | GO:0009657 | 0.000023 |
| pollen tube growth | GO:0009860 | 0.000052 |
| photosynthesis | GO:0015979 | 0.000054 |
| cellular cation homeostasis | GO:0030003 | 0.000058 |
| positive regulation of flavonoid biosynthetic process | GO:0009963 | 0.00016 |
| organonitrogen compound catabolic process | GO:1901565 | 0.00016 |
| carbohydrate metabolic process | GO:0005975 | 0.00021 |
| photosynthesis, light reaction | GO:0019684 | 0.00024 |
| cell wall organization | GO:0071555 | 0.00032 |
| flavonol biosynthetic process | GO:0051555 | 0.00038 |
| cation transport | GO:0006812 | 0.00039 |
| glucosinolate biosynthetic process | GO:0019761 | 0.00045 |
| regulation of pollen tube growth | GO:0080092 | 0.00049 |
| sodium ion transmembrane transport | GO:0035725 | 0.00061 |
| response to UV-B | GO:0010224 | 0.00068 |
| salicylic acid biosynthetic process | GO:0009697 | 0.0009 |
| stomatal complex morphogenesis | GO:0010103 | 0.00098 |
| urea transmembrane transport | GO:0071918 | 0.0011 |
| ATP biosynthetic process | GO:0006754 | 0.00113 |
| water transport | GO:0006833 | 0.00122 |
| chlorophyll catabolic process | GO:0015996 | 0.00123 |
| lipid metabolic process | GO:0006629 | 0.00127 |
| electron transport chain | GO:0022900 | 0.00139 |
| potassium ion transmembrane transport | GO:0071805 | 0.0014 |
| glutamate metabolic process | GO:0006536 | 0.00142 |
| DNA integration | GO:0015074 | 0.00142 |
| extracellular polysaccharide biosynthetic process | GO:0045226 | 0.00143 |
| nonphotochemical quenching | GO:0010196 | 0.00166 |
| cellular response to phosphate starvation | GO:0016036 | 0.00183 |
| protein-chromophore linkage | GO:0018298 | 0.0019 |
| intracellular transport | GO:0046907 | 0.00193 |
| fatty acid biosynthetic process | GO:0006633 | 0.00203 |
| response to blue light | GO:0009637 | 0.00218 |
| hydrogen peroxide catabolic process | GO:0042744 | 0.00258 |
| maltose metabolic process | GO:0000023 | 0.00263 |
| response to sucrose | GO:0009744 | 0.00288 |
| positive regulation of catalytic activity | GO:0043085 | 0.00295 |
| response to organonitrogen compound | GO:0010243 | 0.00316 |
| protein import into chloroplast thylakoid membrane | GO:0045038 | 0.00327 |
| anion transport | GO:0006820 | 0.00328 |
| one-carbon metabolic process | GO:0006730 | 0.00339 |
| UDP-rhamnose biosynthetic process | GO:0010253 | 0.00344 |
| drug transmembrane transport | GO:0006855 | 0.00378 |
| signal transduction by phosphorylation | GO:0023014 | 0.00393 |
| callose deposition in phloem sieve plate | GO:0080165 | 0.00395 |
| ion transport | GO:0006811 | 0.00405 |
| protein transport | GO:0015031 | 0.00433 |
| response to cadmium ion | GO:0046686 | 0.00468 |
| lipid biosynthetic process | GO:0008610 | 0.0048 |
| response to hormone | GO:0009725 | 0.00487 |
| divalent metal ion transport | GO:0070838 | 0.00512 |
| (1->3)-beta-D-glucan biosynthetic process | GO:0006075 | 0.00542 |
| response to jasmonic acid | GO:0009753 | 0.00564 |
| regulation of photosynthesis | GO:0010109 | 0.00573 |
| anthocyanin-containing compound metabolic process | GO:0046283 | 0.00574 |
| trehalose biosynthetic process | GO:0005992 | 0.00576 |
| small molecule metabolic process | GO:0044281 | 0.00702 |
| de-etiolation | GO:0009704 | 0.00717 |
| cell wall macromolecule catabolic process | GO:0016998 | 0.00734 |
| carbon fixation | GO:0015977 | 0.00765 |
| malate metabolic process | GO:0006108 | 0.00781 |
| protein tetramerization | GO:0051262 | 0.00803 |
| detection of biotic stimulus | GO:0009595 | 0.00842 |
| response to salt stress | GO:0009651 | 0.00858 |
| glycolysis | GO:0006096 | 0.00861 |
| transmembrane transport | GO:0055085 | 0.00868 |
| vesicle-mediated transport | GO:0016192 | 0.00877 |
| alcohol metabolic process | GO:0006066 | 0.00896 |
| steroid metabolic process | GO:0008202 | 0.00923 |
| oligopeptide transport | GO:0006857 | 0.00928 |
| organonitrogen compound metabolic process | GO:1901564 | 0.00946 |
| cellular macromolecule localization | GO:0070727 | 0.00975 |
| pectin catabolic process | GO:0045490 | 0.00997 |
| carbohydrate transport | GO:0008643 | 0.01005 |
| intracellular protein transport | GO:0006886 | 0.01028 |
| defense response to bacterium | GO:0042742 | 0.01041 |
| glutamate biosynthetic process | GO:0006537 | 0.01057 |
| phosphate ion transport | GO:0006817 | 0.01106 |
| GTP catabolic process | GO:0006184 | 0.01119 |
| calcium ion homeostasis | GO:0055074 | 0.01134 |
| carboxylic acid metabolic process | GO:0019752 | 0.01148 |
| developmental growth involved in morphogenesis | GO:0060560 | 0.01332 |
| MAPK cascade | GO:0000165 | 0.01349 |
| cytoplasmic transport | GO:0016482 | 0.01361 |
| cellular protein localization | GO:0034613 | 0.0137 |
| response to fungus | GO:0009620 | 0.01392 |
| endosperm development | GO:0009960 | 0.01431 |
| stomatal movement | GO:0010118 | 0.01481 |
| response to chitin | GO:0010200 | 0.01492 |
| steroid biosynthetic process | GO:0006694 | 0.01516 |
| jasmonic acid metabolic process | GO:0009694 | 0.01519 |
| polyamine catabolic process | GO:0006598 | 0.01539 |
| actin filament-based movement | GO:0030048 | 0.01566 |
| zinc ion transport | GO:0006829 | 0.01577 |
| activation of MAPKK activity | GO:0000186 | 0.01583 |
| regulation of hormone levels | GO:0010817 | 0.01621 |
| response to external stimulus | GO:0009605 | 0.01642 |
| cell tip growth | GO:0009932 | 0.0166 |
| phenylpropanoid biosynthetic process | GO:0009699 | 0.01673 |
| unsaturated fatty acid biosynthetic process | GO:0006636 | 0.01685 |
| proton transport | GO:0015992 | 0.0173 |
| response to biotic stimulus | GO:0009607 | 0.01735 |
| response to lipid | GO:0033993 | 0.01771 |
| asparagine biosynthetic process | GO:0006529 | 0.01831 |
| zinc ion transmembrane transport | GO:0071577 | 0.01834 |
| anthocyanin-containing compound biosynthetic process | GO:0009718 | 0.01846 |
| cellular amino acid catabolic process | GO:0009063 | 0.01876 |
| serine family amino acid metabolic process | GO:0009069 | 0.01885 |
| response to nematode | GO:0009624 | 0.01887 |
| acidic amino acid transport | GO:0015800 | 0.01958 |
| inorganic anion transport | GO:0015698 | 0.01972 |
| plant-type spore development | GO:0048236 | 0.01973 |
| photosynthetic electron transport in photosystem II | GO:0009772 | 0.01978 |
| regulation of transport | GO:0051049 | 0.01982 |
| ammonia assimilation cycle | GO:0019676 | 0.02024 |
| plant-type cell wall organization | GO:0009664 | 0.02033 |
| regulation of multi-organism process | GO:0043900 | 0.02116 |
| regulation of photosynthesis, light reaction | GO:0042548 | 0.02117 |
| response to wounding | GO:0009611 | 0.02123 |
| response to alcohol | GO:0097305 | 0.02141 |
| protein homotetramerization | GO:0051289 | 0.02148 |
| response to other organism | GO:0051707 | 0.02159 |
| spermidine biosynthetic process | GO:0008295 | 0.02178 |
| hyperosmotic response | GO:0006972 | 0.02222 |
| polyamine biosynthetic process | GO:0006596 | 0.02295 |
| nucleotide-sugar metabolic process | GO:0009225 | 0.0239 |
| purine ribonucleotide catabolic process | GO:0009154 | 0.02494 |
| purine ribonucleoside triphosphate catabolic process | GO:0009207 | 0.02494 |
| regulation of pH | GO:0006885 | 0.025 |
| oxylipin metabolic process | GO:0031407 | 0.02504 |
| starch biosynthetic process | GO:0019252 | 0.02517 |
| response to abscisic acid | GO:0009737 | 0.02545 |
| RNA-dependent DNA replication | GO:0006278 | 0.02592 |
| regulation of protein phosphorylation | GO:0001932 | 0.02631 |
| purine ribonucleoside catabolic process | GO:0046130 | 0.02654 |
| phosphatidylglycerol biosynthetic process | GO:0006655 | 0.02664 |
| cell wall pectin biosynthetic process | GO:0052325 | 0.02759 |
| cellular amino acid metabolic process | GO:0006520 | 0.02775 |
| PSII associated light-harvesting complex II catabolic process | GO:0010304 | 0.02797 |
| nucleotide catabolic process | GO:0009166 | 0.02823 |
| ionotropic glutamate receptor signaling pathway | GO:0035235 | 0.02847 |
| regulation of ion transport | GO:0043269 | 0.02862 |
| galactose transport | GO:0015757 | 0.02891 |
| cellular calcium ion homeostasis | GO:0006874 | 0.02952 |
| negative regulation of iron ion transport | GO:0034757 | 0.02972 |
| cellular response to cold | GO:0070417 | 0.02972 |
| leaf morphogenesis | GO:0009965 | 0.03001 |
| reductive pentose-phosphate cycle | GO:0019253 | 0.03015 |
| gibberellic acid mediated signaling pathway | GO:0009740 | 0.03022 |
| cell wall thickening | GO:0052386 | 0.03028 |
| positive regulation of protein kinase activity | GO:0045860 | 0.03029 |
| hyperosmotic salinity response | GO:0042538 | 0.03064 |
| defense response to fungus | GO:0050832 | 0.03149 |
| flavonoid biosynthetic process | GO:0009813 | 0.03151 |
| cellular glucan metabolic process | GO:0006073 | 0.03179 |
| purine nucleoside catabolic process | GO:0006152 | 0.03188 |
| response to bacterium | GO:0009617 | 0.03247 |
| regulation of stomatal movement | GO:0010119 | 0.03403 |
| chlorophyll metabolic process | GO:0015994 | 0.03442 |
| pollen germination | GO:0009846 | 0.03457 |
| sucrose transport | GO:0015770 | 0.03545 |
| regulation of protein kinase activity | GO:0045859 | 0.03601 |
| positive regulation of abscisic acid-activated signaling pathway | GO:0009789 | 0.03653 |
| cellular lipid metabolic process | GO:0044255 | 0.03735 |
| amine metabolic process | GO:0009308 | 0.03752 |
| positive regulation of transcription, DNA-templated | GO:0045893 | 0.03788 |
| negative regulation of catalytic activity | GO:0043086 | 0.03795 |
| defense response, incompatible interaction | GO:0009814 | 0.03853 |
| asparaginyl-tRNA aminoacylation | GO:0006421 | 0.03872 |
| regulation of translational fidelity | GO:0006450 | 0.03898 |
| response to oxygen-containing compound | GO:1901700 | 0.03902 |
| organonitrogen compound biosynthetic process | GO:1901566 | 0.03903 |
| glycine metabolic process | GO:0006544 | 0.03918 |
| microsporogenesis | GO:0009556 | 0.03948 |
| alcohol biosynthetic process | GO:0046165 | 0.04034 |
| response to salicylic acid | GO:0009751 | 0.0404 |
| indole-containing compound metabolic process | GO:0042430 | 0.04051 |
| translation | GO:0006412 | 0.04056 |
| positive regulation of phosphorylation | GO:0042327 | 0.04127 |
| oxylipin biosynthetic process | GO:0031408 | 0.04188 |
| root epidermal cell differentiation | GO:0010053 | 0.04192 |
| nitrate assimilation | GO:0042128 | 0.04202 |
| positive regulation of gene expression | GO:0010628 | 0.04243 |
| plant-type hypersensitive response | GO:0009626 | 0.04263 |
| polysaccharide metabolic process | GO:0005976 | 0.04575 |
| proline biosynthetic process | GO:0006561 | 0.04591 |
| glutamine metabolic process | GO:0006541 | 0.0462 |
| syncytium formation | GO:0006949 | 0.04708 |
| cellular response to iron ion starvation | GO:0010106 | 0.04786 |
| coenzyme A metabolic process | GO:0015936 | 0.04829 |
| unidimensional cell growth | GO:0009826 | 0.04866 |
| phosphatidylinositol dephosphorylation | GO:0046856 | 0.04878 |
| photosynthetic electron transport chain | GO:0009767 | 0.04984 |
| GO description (CDSE_CDP) | GO term | P value |
| oxidation-reduction process | GO:0055114 | 1.9E-17 |
| thylakoid membrane organization | GO:0010027 | 1.3E-15 |
| photosystem II assembly | GO:0010207 | 2.3E-15 |
| photosynthesis, light harvesting | GO:0009765 | 1.5E-14 |
| isopentenyl diphosphate biosynthetic process, methylerythritol 4-phosphate pathway | GO:0019288 | 1.5E-14 |
| rRNA processing | GO:0006364 | 1E-13 |
| pentose-phosphate shunt | GO:0006098 | 2.7E-13 |
| regulation of protein dephosphorylation | GO:0035304 | 2.9E-13 |
| photosynthetic electron transport in photosystem I | GO:0009773 | 5E-13 |
| carotenoid biosynthetic process | GO:0016117 | 3E-12 |
| chloroplast relocation | GO:0009902 | 6.6E-11 |
| chlorophyll biosynthetic process | GO:0015995 | 1.6E-09 |
| response to red light | GO:0010114 | 8.6E-09 |
| cysteine biosynthetic process | GO:0019344 | 2E-08 |
| photosynthesis | GO:0015979 | 4.9E-08 |
| DNA integration | GO:0015074 | 7.1E-08 |
| glucosinolate biosynthetic process | GO:0019761 | 9.7E-08 |
| plastid organization | GO:0009657 | 1.4E-07 |
| response to far red light | GO:0010218 | 5.1E-07 |
| regulation of meristem growth | GO:0010075 | 1.1E-06 |
| response to blue light | GO:0009637 | 1.9E-06 |
| photosynthesis, light reaction | GO:0019684 | 2.5E-06 |
| oxylipin biosynthetic process | GO:0031408 | 5.5E-06 |
| transcription from plastid promoter | GO:0042793 | 9.8E-06 |
| response to water deprivation | GO:0009414 | 0.00002 |
| starch biosynthetic process | GO:0019252 | 0.00003 |
| maltose metabolic process | GO:0000023 | 0.000048 |
| chlorophyll catabolic process | GO:0015996 | 0.000066 |
| regulation of proton transport | GO:0010155 | 0.000079 |
| salicylic acid biosynthetic process | GO:0009697 | 0.000088 |
| defense response to bacterium | GO:0042742 | 0.000088 |
| nonphotochemical quenching | GO:0010196 | 0.00012 |
| systemic acquired resistance, salicylic acid mediated signaling pathway | GO:0009862 | 0.00013 |
| protein phosphorylation | GO:0006468 | 0.00014 |
| DNA unwinding involved in DNA replication | GO:0006268 | 0.00016 |
| regulation of hydrogen peroxide metabolic process | GO:0010310 | 0.00017 |
| negative regulation of catalytic activity | GO:0043086 | 0.00019 |
| lignin biosynthetic process | GO:0009809 | 0.00023 |
| RNA-dependent DNA replication | GO:0006278 | 0.00034 |
| iron-sulfur cluster assembly | GO:0016226 | 0.00043 |
| unsaturated fatty acid biosynthetic process | GO:0006636 | 0.00045 |
| protein import into chloroplast thylakoid membrane | GO:0045038 | 0.0006 |
| flavonoid biosynthetic process | GO:0009813 | 0.00061 |
| transmembrane receptor protein tyrosine kinase signaling pathway | GO:0007169 | 0.00064 |
| tryptophan catabolic process | GO:0006569 | 0.00087 |
| regulation of cell size | GO:0008361 | 0.0009 |
| cuticle development | GO:0042335 | 0.00094 |
| jasmonic acid mediated signaling pathway | GO:0009867 | 0.00104 |
| indole-containing compound metabolic process | GO:0042430 | 0.00105 |
| oligopeptide transport | GO:0006857 | 0.00108 |
| microtubule-based process | GO:0007017 | 0.00115 |
| organonitrogen compound biosynthetic process | GO:1901566 | 0.00115 |
| cellular cation homeostasis | GO:0030003 | 0.00128 |
| hydrogen peroxide catabolic process | GO:0042744 | 0.00135 |
| auxin polar transport | GO:0009926 | 0.00157 |
| response to nematode | GO:0009624 | 0.00161 |
| reductive pentose-phosphate cycle | GO:0019253 | 0.00171 |
| xylem development | GO:0010089 | 0.00199 |
| anthocyanin-containing compound metabolic process | GO:0046283 | 0.002 |
| stomatal complex morphogenesis | GO:0010103 | 0.00208 |
| myo-inositol hexakisphosphate biosynthetic process | GO:0010264 | 0.00247 |
| intracellular protein transport | GO:0006886 | 0.00249 |
| cellular protein metabolic process | GO:0044267 | 0.00271 |
| indoleacetic acid biosynthetic process | GO:0009684 | 0.00321 |
| response to nitrate | GO:0010167 | 0.00347 |
| aromatic amino acid family metabolic process | GO:0009072 | 0.00362 |
| response to absence of light | GO:0009646 | 0.00391 |
| asymmetric cell division | GO:0008356 | 0.00411 |
| detection of biotic stimulus | GO:0009595 | 0.00488 |
| negative regulation of defense response | GO:0031348 | 0.00537 |
| amino acid transmembrane transport | GO:0003333 | 0.00562 |
| carbohydrate metabolic process | GO:0005975 | 0.00566 |
| defense response to fungus | GO:0050832 | 0.00568 |
| inflorescence development | GO:0010229 | 0.0059 |
| translation | GO:0006412 | 0.00625 |
| activation of MAPKK activity | GO:0000186 | 0.00648 |
| regulation of flavonoid biosynthetic process | GO:0009962 | 0.00664 |
| nitrate transport | GO:0015706 | 0.00677 |
| L-phenylalanine catabolic process | GO:0006559 | 0.00685 |
| pigment biosynthetic process | GO:0046148 | 0.00702 |
| cytoplasmic transport | GO:0016482 | 0.00746 |
| acidic amino acid transport | GO:0015800 | 0.00774 |
| carboxylic acid biosynthetic process | GO:0046394 | 0.00776 |
| positive regulation of gene expression | GO:0010628 | 0.00792 |
| regulation of catalytic activity | GO:0050790 | 0.00792 |
| host programmed cell death induced by symbiont | GO:0034050 | 0.0081 |
| hyperosmotic salinity response | GO:0042538 | 0.00912 |
| plant-type hypersensitive response | GO:0009626 | 0.01027 |
| response to desiccation | GO:0009269 | 0.0105 |
| positive regulation of catalytic activity | GO:0043085 | 0.01074 |
| amine metabolic process | GO:0009308 | 0.01081 |
| very long-chain fatty acid metabolic process | GO:0000038 | 0.01105 |
| small molecule biosynthetic process | GO:0044283 | 0.01108 |
| cinnamic acid biosynthetic process | GO:0009800 | 0.01111 |
| positive regulation of transcription, DNA-templated | GO:0045893 | 0.01144 |
| regulation of immune response | GO:0050776 | 0.01155 |
| wax metabolic process | GO:0010166 | 0.01157 |
| protein import into chloroplast stroma | GO:0045037 | 0.01243 |
| cytokinin-activated signaling pathway | GO:0009736 | 0.01289 |
| syncytium formation | GO:0006949 | 0.0134 |
| response to external stimulus | GO:0009605 | 0.01351 |
| regulation of innate immune response | GO:0045088 | 0.01386 |
| ionotropic glutamate receptor signaling pathway | GO:0035235 | 0.01421 |
| glycine catabolic process | GO:0006546 | 0.01451 |
| cell wall organization | GO:0071555 | 0.01492 |
| floral whorl development | GO:0048438 | 0.0163 |
| PSII associated light-harvesting complex II catabolic process | GO:0010304 | 0.01633 |
| monosaccharide transport | GO:0015749 | 0.01633 |
| tryptophan metabolic process | GO:0006568 | 0.01741 |
| cell proliferation | GO:0008283 | 0.01774 |
| response to carbohydrate | GO:0009743 | 0.01835 |
| response to abiotic stimulus | GO:0009628 | 0.01836 |
| photosynthetic electron transport in photosystem II | GO:0009772 | 0.01968 |
| UDP-rhamnose biosynthetic process | GO:0010253 | 0.01989 |
| protein targeting | GO:0006605 | 0.02113 |
| response to hypoxia | GO:0001666 | 0.02134 |
| photosystem II repair | GO:0010206 | 0.02186 |
| secondary metabolic process | GO:0019748 | 0.02212 |
| response to molecule of fungal origin | GO:0002238 | 0.02252 |
| flavonol biosynthetic process | GO:0051555 | 0.02264 |
| phyllome development | GO:0048827 | 0.02267 |
| response to heat | GO:0009408 | 0.02338 |
| cellular lipid metabolic process | GO:0044255 | 0.02345 |
| wax biosynthetic process | GO:0010025 | 0.02497 |
| response to metal ion | GO:0010038 | 0.02524 |
| cytokinesis by cell plate formation | GO:0000911 | 0.02531 |
| CVT pathway | GO:0032258 | 0.02552 |
| photoprotection | GO:0010117 | 0.02559 |
| phenylpropanoid biosynthetic process | GO:0009699 | 0.02575 |
| cell wall thickening | GO:0052386 | 0.02587 |
| response to endogenous stimulus | GO:0009719 | 0.0259 |
| regulation of cell death | GO:0010941 | 0.02602 |
| response to biotic stimulus | GO:0009607 | 0.02629 |
| response to oxidative stress | GO:0006979 | 0.0263 |
| glutamine biosynthetic process | GO:0006542 | 0.02661 |
| cytokinesis | GO:0000910 | 0.02674 |
| galactose transport | GO:0015757 | 0.02695 |
| pattern specification process | GO:0007389 | 0.02696 |
| response to drug | GO:0042493 | 0.02706 |
| leaf morphogenesis | GO:0009965 | 0.02711 |
| protein catabolic process | GO:0030163 | 0.02744 |
| regulation of photosynthesis, light reaction | GO:0042548 | 0.02772 |
| abscisic acid-activated signaling pathway | GO:0009738 | 0.02812 |
| lipid biosynthetic process | GO:0008610 | 0.02849 |
| regulation of programmed cell death | GO:0043067 | 0.02865 |
| phosphatidylglycerol biosynthetic process | GO:0006655 | 0.0288 |
| chloroplast organization | GO:0009658 | 0.02921 |
| cell wall organization or biogenesis | GO:0071554 | 0.0296 |
| anthocyanin-containing compound biosynthetic process | GO:0009718 | 0.03018 |
| cellular amino acid biosynthetic process | GO:0008652 | 0.03069 |
| response to hormone | GO:0009725 | 0.03081 |
| cellular protein modification process | GO:0006464 | 0.03105 |
| phenylpropanoid metabolic process | GO:0009698 | 0.03213 |
| MAPK cascade | GO:0000165 | 0.03272 |
| cell wall macromolecule metabolic process | GO:0044036 | 0.03368 |
| response to ethylene | GO:0009723 | 0.03375 |
| cellular macromolecule catabolic process | GO:0044265 | 0.03405 |
| lipid oxidation | GO:0034440 | 0.03409 |
| cellular response to abscisic acid stimulus | GO:0071215 | 0.03416 |
| isoprenoid biosynthetic process | GO:0008299 | 0.03465 |
| response to chitin | GO:0010200 | 0.0347 |
| response to stimulus | GO:0050896 | 0.03486 |
| coenzyme A metabolic process | GO:0015936 | 0.03512 |
| regulation of photosynthesis | GO:0010109 | 0.03516 |
| fatty acid catabolic process | GO:0009062 | 0.03557 |
| regulation of plant-type hypersensitive response | GO:0010363 | 0.03628 |
| response to organonitrogen compound | GO:0010243 | 0.03644 |
| response to sucrose | GO:0009744 | 0.03731 |
| macromolecule modification | GO:0043412 | 0.03801 |
| small molecule metabolic process | GO:0044281 | 0.03911 |
| de-etiolation | GO:0009704 | 0.03979 |
| protein-chromophore linkage | GO:0018298 | 0.04023 |
| phosphate ion transport | GO:0006817 | 0.04109 |
| response to organic substance | GO:0010033 | 0.04148 |
| entrainment of circadian clock | GO:0009649 | 0.04237 |
| negative regulation of peptidase activity | GO:0010466 | 0.04525 |
| response to cadmium ion | GO:0046686 | 0.0456 |
| fatty acid beta-oxidation | GO:0006635 | 0.04575 |
| adaxial/abaxial axis specification | GO:0009943 | 0.04645 |
| carbohydrate transport | GO:0008643 | 0.04752 |
| response to chemical | GO:0042221 | 0.04758 |
| carbohydrate derivative biosynthetic process | GO:1901137 | 0.04823 |
| polarity specification of adaxial/abaxial axis | GO:0009944 | 0.04885 |
| callose deposition in cell wall | GO:0052543 | 0.04994 |
| GO description (JinOP_JinSE) | GO term | P value |
| DNA integration | GO:0015074 | 1.6E-19 |
| oxidation-reduction process | GO:0055114 | 1.2E-11 |
| RNA-dependent DNA replication | GO:0006278 | 2.8E-11 |
| photosynthetic electron transport in photosystem I | GO:0009773 | 1.5E-06 |
| L-phenylalanine catabolic process | GO:0006559 | 0.00002 |
| response to red light | GO:0010114 | 0.000022 |
| defense response to bacterium | GO:0042742 | 0.000027 |
| cinnamic acid biosynthetic process | GO:0009800 | 0.000084 |
| MAPK cascade | GO:0000165 | 0.00022 |
| carbohydrate metabolic process | GO:0005975 | 0.00024 |
| response to blue light | GO:0009637 | 0.00027 |
| regulation of cell size | GO:0008361 | 0.00029 |
| organonitrogen compound biosynthetic process | GO:1901566 | 0.00046 |
| salicylic acid biosynthetic process | GO:0009697 | 0.0005 |
| intracellular protein transport | GO:0006886 | 0.00141 |
| lipid biosynthetic process | GO:0008610 | 0.00168 |
| cellular lipid metabolic process | GO:0044255 | 0.00175 |
| systemic acquired resistance, salicylic acid mediated signaling pathway | GO:0009862 | 0.00188 |
| response to far red light | GO:0010218 | 0.00194 |
| negative regulation of catalytic activity | GO:0043086 | 0.00198 |
| cysteine biosynthetic process | GO:0019344 | 0.00206 |
| lignin biosynthetic process | GO:0009809 | 0.00221 |
| auxin polar transport | GO:0009926 | 0.00223 |
| asymmetric cell division | GO:0008356 | 0.00224 |
| defense response to fungus | GO:0050832 | 0.00239 |
| chlorophyll metabolic process | GO:0015994 | 0.00316 |
| photosystem II assembly | GO:0010207 | 0.0032 |
| detection of biotic stimulus | GO:0009595 | 0.00328 |
| xylem development | GO:0010089 | 0.0035 |
| anthocyanin-containing compound biosynthetic process | GO:0009718 | 0.00372 |
| negative regulation of defense response | GO:0031348 | 0.00377 |
| translation | GO:0006412 | 0.00418 |
| response to nematode | GO:0009624 | 0.00446 |
| amino acid transmembrane transport | GO:0003333 | 0.00453 |
| cytoplasmic transport | GO:0016482 | 0.00491 |
| pigment metabolic process | GO:0042440 | 0.00531 |
| flavonoid biosynthetic process | GO:0009813 | 0.00532 |
| flavonol biosynthetic process | GO:0051555 | 0.00542 |
| response to UV | GO:0009411 | 0.00575 |
| carboxylic acid biosynthetic process | GO:0046394 | 0.00582 |
| reductive pentose-phosphate cycle | GO:0019253 | 0.00608 |
| jasmonic acid mediated signaling pathway | GO:0009867 | 0.00642 |
| respiratory burst | GO:0045730 | 0.00672 |
| small molecule metabolic process | GO:0044281 | 0.00795 |
| regulation of protein dephosphorylation | GO:0035304 | 0.00843 |
| host programmed cell death induced by symbiont | GO:0034050 | 0.00849 |
| response to endogenous stimulus | GO:0009719 | 0.00891 |
| chemical homeostasis | GO:0048878 | 0.00928 |
| regulation of flavonoid biosynthetic process | GO:0009962 | 0.00955 |
| oxylipin biosynthetic process | GO:0031408 | 0.00958 |
| response to abiotic stimulus | GO:0009628 | 0.00991 |
| UDP-rhamnose biosynthetic process | GO:0010253 | 0.00999 |
| regulation of reactive oxygen species metabolic process | GO:2000377 | 0.0102 |
| fatty acid metabolic process | GO:0006631 | 0.01031 |
| ATP catabolic process | GO:0006200 | 0.01045 |
| ribosome biogenesis | GO:0042254 | 0.01063 |
| photosynthesis, light harvesting | GO:0009765 | 0.01066 |
| plant-type hypersensitive response | GO:0009626 | 0.01169 |
| response to ethylene | GO:0009723 | 0.01188 |
| regulation of hydrogen peroxide metabolic process | GO:0010310 | 0.01194 |
| response to hormone | GO:0009725 | 0.01206 |
| regulation of multi-organism process | GO:0043900 | 0.01223 |
| monocarboxylic acid metabolic process | GO:0032787 | 0.01292 |
| pigment biosynthetic process | GO:0046148 | 0.01326 |
| anthocyanin accumulation in tissues in response to UV light | GO:0043481 | 0.01347 |
| response to wounding | GO:0009611 | 0.01351 |
| pentose-phosphate shunt | GO:0006098 | 0.01367 |
| indole-containing compound metabolic process | GO:0042430 | 0.01548 |
| phospholipid biosynthetic process | GO:0008654 | 0.01558 |
| regulation of immune response | GO:0050776 | 0.01576 |
| amine metabolic process | GO:0009308 | 0.01579 |
| carbohydrate derivative biosynthetic process | GO:1901137 | 0.01592 |
| response to organonitrogen compound | GO:0010243 | 0.0161 |
| protein targeting | GO:0006605 | 0.01614 |
| coenzyme A metabolic process | GO:0015936 | 0.017 |
| phenylpropanoid metabolic process | GO:0009698 | 0.01709 |
| glucosinolate metabolic process | GO:0019760 | 0.01712 |
| cuticle development | GO:0042335 | 0.01733 |
| pigmentation | GO:0043473 | 0.01804 |
| respiratory burst involved in defense response | GO:0002679 | 0.01817 |
| regulation of innate immune response | GO:0045088 | 0.01918 |
| isoprenoid biosynthetic process | GO:0008299 | 0.0194 |
| catabolic process | GO:0009056 | 0.01963 |
| response to chitin | GO:0010200 | 0.01975 |
| cell wall macromolecule catabolic process | GO:0016998 | 0.02098 |
| protein catabolic process | GO:0030163 | 0.02117 |
| response to metal ion | GO:0010038 | 0.02141 |
| NADP metabolic process | GO:0006739 | 0.02186 |
| activation of MAPKK activity | GO:0000186 | 0.02225 |
| response to oxidative stress | GO:0006979 | 0.02231 |
| abscisic acid-activated signaling pathway | GO:0009738 | 0.02305 |
| tryptophan metabolic process | GO:0006568 | 0.02397 |
| response to inorganic substance | GO:0010035 | 0.02421 |
| response to light intensity | GO:0009642 | 0.02433 |
| carboxylic acid metabolic process | GO:0019752 | 0.02456 |
| rRNA processing | GO:0006364 | 0.02506 |
| response to heat | GO:0009408 | 0.0252 |
| regulation of protein kinase activity | GO:0045859 | 0.02574 |
| ER-nucleus signaling pathway | GO:0006984 | 0.02639 |
| sulfur compound metabolic process | GO:0006790 | 0.0267 |
| response to high light intensity | GO:0009644 | 0.02718 |
| extracellular polysaccharide biosynthetic process | GO:0045226 | 0.02727 |
| cellular macromolecule catabolic process | GO:0044265 | 0.02728 |
| chlorophyll biosynthetic process | GO:0015995 | 0.02809 |
| response to gibberellin | GO:0009739 | 0.02833 |
| protein localization | GO:0008104 | 0.02896 |
| glucose catabolic process | GO:0006007 | 0.02944 |
| chitin catabolic process | GO:0006032 | 0.02982 |
| protein transport | GO:0015031 | 0.03001 |
| cell wall thickening | GO:0052386 | 0.03053 |
| zinc ion transmembrane transport | GO:0071577 | 0.03063 |
| gibberellic acid mediated signaling pathway | GO:0009740 | 0.03066 |
| carotenoid biosynthetic process | GO:0016117 | 0.03086 |
| tetrapyrrole biosynthetic process | GO:0033014 | 0.03101 |
| oxygen transport | GO:0015671 | 0.0314 |
| folic acid transport | GO:0015884 | 0.03307 |
| regulation of plant-type hypersensitive response | GO:0010363 | 0.03309 |
| glucosinolate biosynthetic process | GO:0019761 | 0.03374 |
| response to water deprivation | GO:0009414 | 0.03399 |
| glutamate biosynthetic process | GO:0006537 | 0.03408 |
| regulation of abscisic acid-activated signaling pathway | GO:0009787 | 0.03408 |
| hyperosmotic salinity response | GO:0042538 | 0.03463 |
| response to carbohydrate | GO:0009743 | 0.03511 |
| positive regulation of protein kinase activity | GO:0045860 | 0.03617 |
| endoplasmic reticulum unfolded protein response | GO:0030968 | 0.03678 |
| ncRNA metabolic process | GO:0034660 | 0.03822 |
| cellular ion homeostasis | GO:0006873 | 0.03854 |
| regulation of protein phosphorylation | GO:0001932 | 0.03879 |
| porphyrin-containing compound biosynthetic process | GO:0006779 | 0.03896 |
| alcohol metabolic process | GO:0006066 | 0.03946 |
| positive regulation of phosphorylation | GO:0042327 | 0.03974 |
| response to temperature stimulus | GO:0009266 | 0.04047 |
| cellular cation homeostasis | GO:0030003 | 0.04051 |
| macromolecule localization | GO:0033036 | 0.04066 |
| positive regulation of abscisic acid-activated signaling pathway | GO:0009789 | 0.04136 |
| regulation of cell death | GO:0010941 | 0.0414 |
| response to oxygen-containing compound | GO:1901700 | 0.04157 |
| response to hydrogen peroxide | GO:0042542 | 0.04158 |
| phospholipid metabolic process | GO:0006644 | 0.04243 |
| fatty acid biosynthetic process | GO:0006633 | 0.04393 |
| cellular amino acid metabolic process | GO:0006520 | 0.04426 |
| cellular homeostasis | GO:0019725 | 0.04428 |
| RNA processing | GO:0006396 | 0.0447 |
| glucose metabolic process | GO:0006006 | 0.04513 |
| cellular amino acid biosynthetic process | GO:0008652 | 0.04518 |
| proteolysis involved in cellular protein catabolic process | GO:0051603 | 0.04621 |
| vesicle-mediated transport | GO:0016192 | 0.04649 |
| defense response signaling pathway, resistance gene-independent | GO:0010204 | 0.04653 |
| zinc ion transport | GO:0006829 | 0.04715 |
| tryptophan catabolic process | GO:0006569 | 0.04848 |
| oligosaccharide transport | GO:0015772 | 0.04881 |
| mitochondrial transport | GO:0006839 | 0.04902 |
| response to chemical | GO:0042221 | 0.04928 |
| cell wall macromolecule metabolic process | GO:0044036 | 0.04952 |
| GO description (JinSE_JinIP) | GO term | P value |
| oxidation-reduction process | GO:0055114 | 2.6E-20 |
| DNA integration | GO:0015074 | 2.9E-16 |
| RNA-dependent DNA replication | GO:0006278 | 2E-09 |
| response to red light | GO:0010114 | 1.9E-07 |
| photosynthetic electron transport in photosystem I | GO:0009773 | 8.8E-07 |
| response to far red light | GO:0010218 | 3.9E-06 |
| response to blue light | GO:0009637 | 8.7E-06 |
| L-phenylalanine catabolic process | GO:0006559 | 0.000068 |
| negative regulation of catalytic activity | GO:0043086 | 0.000092 |
| defense response to bacterium | GO:0042742 | 0.000093 |
| photosystem II assembly | GO:0010207 | 0.00013 |
| cinnamic acid biosynthetic process | GO:0009800 | 0.00025 |
| photosynthesis, light harvesting | GO:0009765 | 0.0003 |
| lignin catabolic process | GO:0046274 | 0.00044 |
| lignin biosynthetic process | GO:0009809 | 0.00047 |
| regulation of protein dephosphorylation | GO:0035304 | 0.00054 |
| photosynthesis | GO:0015979 | 0.00067 |
| transmembrane receptor protein tyrosine kinase signaling pathway | GO:0007169 | 0.00075 |
| glucosinolate biosynthetic process | GO:0019761 | 0.00083 |
| oxylipin biosynthetic process | GO:0031408 | 0.00106 |
| organonitrogen compound biosynthetic process | GO:1901566 | 0.00119 |
| response to nitrate | GO:0010167 | 0.00175 |
| carotenoid biosynthetic process | GO:0016117 | 0.00227 |
| response to nematode | GO:0009624 | 0.00243 |
| intracellular protein transport | GO:0006886 | 0.0025 |
| response to water deprivation | GO:0009414 | 0.00278 |
| pentose-phosphate shunt | GO:0006098 | 0.00287 |
| lipid biosynthetic process | GO:0008610 | 0.00339 |
| phosphate ion transport | GO:0006817 | 0.00379 |
| response to hormone | GO:0009725 | 0.00407 |
| cellular lipid metabolic process | GO:0044255 | 0.00409 |
| carbohydrate metabolic process | GO:0005975 | 0.0041 |
| cysteine biosynthetic process | GO:0019344 | 0.00421 |
| regulation of cell size | GO:0008361 | 0.00429 |
| flavonoid biosynthetic process | GO:0009813 | 0.00437 |
| reductive pentose-phosphate cycle | GO:0019253 | 0.00464 |
| cell wall thickening | GO:0052386 | 0.00488 |
| regulation of hormone levels | GO:0010817 | 0.00502 |
| chlorophyll biosynthetic process | GO:0015995 | 0.00508 |
| nitrate assimilation | GO:0042128 | 0.00525 |
| salicylic acid biosynthetic process | GO:0009697 | 0.00559 |
| CVT pathway | GO:0032258 | 0.00611 |
| amino acid transmembrane transport | GO:0003333 | 0.00643 |
| xylem development | GO:0010089 | 0.00648 |
| tryptophan metabolic process | GO:0006568 | 0.00649 |
| translation | GO:0006412 | 0.0067 |
| carboxylic acid biosynthetic process | GO:0046394 | 0.00694 |
| systemic acquired resistance, salicylic acid mediated signaling pathway | GO:0009862 | 0.00717 |
| anthocyanin accumulation in tissues in response to UV light | GO:0043481 | 0.00821 |
| response to wounding | GO:0009611 | 0.00822 |
| cytoplasmic transport | GO:0016482 | 0.00828 |
| glutamate biosynthetic process | GO:0006537 | 0.00994 |
| chemical homeostasis | GO:0048878 | 0.01006 |
| regulation of meristem growth | GO:0010075 | 0.0101 |
| single-organism transport | GO:0044765 | 0.0101 |
| response to oxidative stress | GO:0006979 | 0.01029 |
| ATP catabolic process | GO:0006200 | 0.01098 |
| response to fungus | GO:0009620 | 0.01141 |
| chloroplast relocation | GO:0009902 | 0.01185 |
| defense response by callose deposition | GO:0052542 | 0.01212 |
| callose deposition in cell wall | GO:0052543 | 0.01236 |
| pigment metabolic process | GO:0042440 | 0.01254 |
| flavonol biosynthetic process | GO:0051555 | 0.01299 |
| ribosome biogenesis | GO:0042254 | 0.01344 |
| response to molecule of fungal origin | GO:0002238 | 0.01382 |
| phospholipid biosynthetic process | GO:0008654 | 0.01421 |
| small molecule metabolic process | GO:0044281 | 0.01466 |
| response to abiotic stimulus | GO:0009628 | 0.01473 |
| response to high light intensity | GO:0009644 | 0.01693 |
| fatty acid metabolic process | GO:0006631 | 0.01702 |
| rRNA processing | GO:0006364 | 0.01778 |
| response to heat | GO:0009408 | 0.01792 |
| MAPK cascade | GO:0000165 | 0.01916 |
| response to jasmonic acid | GO:0009753 | 0.01946 |
| signal transduction by phosphorylation | GO:0023014 | 0.01983 |
| oligopeptide transport | GO:0006857 | 0.0205 |
| response to metal ion | GO:0010038 | 0.02098 |
| short-chain fatty acid metabolic process | GO:0046459 | 0.02123 |
| response to desiccation | GO:0009269 | 0.02133 |
| carboxylic acid metabolic process | GO:0019752 | 0.02162 |
| hydrogen peroxide catabolic process | GO:0042744 | 0.02185 |
| negative regulation of defense response | GO:0031348 | 0.02238 |
| protein phosphorylation | GO:0006468 | 0.0224 |
| regulation of hydrogen peroxide metabolic process | GO:0010310 | 0.02252 |
| regulation of reactive oxygen species metabolic process | GO:2000377 | 0.0227 |
| response to inorganic substance | GO:0010035 | 0.02327 |
| protein targeting | GO:0006605 | 0.02331 |
| defense response to fungus | GO:0050832 | 0.02374 |
| cuticle development | GO:0042335 | 0.02542 |
| catabolic process | GO:0009056 | 0.02695 |
| detection of biotic stimulus | GO:0009595 | 0.02705 |
| coenzyme A metabolic process | GO:0015936 | 0.02822 |
| regulation of developmental growth | GO:0048638 | 0.02902 |
| protein catabolic process | GO:0030163 | 0.02924 |
| auxin polar transport | GO:0009926 | 0.02971 |
| chitin catabolic process | GO:0006032 | 0.03032 |
| cellular response to hydrogen peroxide | GO:0070301 | 0.03191 |
| cellular amino acid metabolic process | GO:0006520 | 0.03207 |
| RNA processing | GO:0006396 | 0.03242 |
| glycerol ether metabolic process | GO:0006662 | 0.03275 |
| regulation of meristem development | GO:0048509 | 0.03372 |
| phospholipid metabolic process | GO:0006644 | 0.03416 |
| monocarboxylic acid metabolic process | GO:0032787 | 0.03483 |
| cellular amino acid biosynthetic process | GO:0008652 | 0.03504 |
| ncRNA metabolic process | GO:0034660 | 0.03512 |
| ammonia assimilation cycle | GO:0019676 | 0.03603 |
| tryptophan catabolic process | GO:0006569 | 0.0366 |
| cellular macromolecule catabolic process | GO:0044265 | 0.0366 |
| nitrate transport | GO:0015706 | 0.03667 |
| para-aminobenzoic acid metabolic process | GO:0046482 | 0.0389 |
| isoprenoid biosynthetic process | GO:0008299 | 0.0395 |
| fatty acid catabolic process | GO:0009062 | 0.04145 |
| adaxial/abaxial axis specification | GO:0009943 | 0.04165 |
| lipid oxidation | GO:0034440 | 0.04257 |
| folic acid transport | GO:0015884 | 0.04313 |
| response to light intensity | GO:0009642 | 0.04329 |
| cellular response to auxin stimulus | GO:0071365 | 0.04385 |
| (1->3)-beta-D-glucan biosynthetic process | GO:0006075 | 0.04421 |
| asymmetric cell division | GO:0008356 | 0.04532 |
| regulation of auxin mediated signaling pathway | GO:0010928 | 0.0458 |
| cellular cation homeostasis | GO:0030003 | 0.04588 |
| response to organonitrogen compound | GO:0010243 | 0.04607 |
| starch biosynthetic process | GO:0019252 | 0.04671 |
| polarity specification of adaxial/abaxial axis | GO:0009944 | 0.04693 |
| defense response by callose deposition in cell wall | GO:0052544 | 0.04699 |
| alcohol metabolic process | GO:0006066 | 0.04735 |
| response to carbohydrate | GO:0009743 | 0.04735 |
| cellular ion homeostasis | GO:0006873 | 0.04768 |
| response to chitin | GO:0010200 | 0.04793 |
| response to cadmium ion | GO:0046686 | 0.04833 |
| abscisic acid-activated signaling pathway | GO:0009738 | 0.0491 |
| negative regulation of peptidase activity | GO:0010466 | 0.04942 |
| carbohydrate derivative biosynthetic process | GO:1901137 | 0.04942 |
| zinc ion transmembrane transport | GO:0071577 | 0.04953 |
| GO description(JinOP_JinST) | GO term | P value |
| oxidation-reduction process | GO:0055114 | 3.8E-10 |
| response to far red light | GO:0010218 | 5.5E-10 |
| response to red light | GO:0010114 | 6E-10 |
| DNA integration | GO:0015074 | 2.2E-09 |
| glycolysis | GO:0006096 | 9E-07 |
| carotenoid biosynthetic process | GO:0016117 | 0.000009 |
| plant-type cell wall modification | GO:0009827 | 9.8E-06 |
| response to blue light | GO:0009637 | 0.000022 |
| oxylipin biosynthetic process | GO:0031408 | 0.000046 |
| response to karrikin | GO:0080167 | 0.000049 |
| fatty acid biosynthetic process | GO:0006633 | 0.000068 |
| (1->3)-beta-D-glucan biosynthetic process | GO:0006075 | 0.000071 |
| cysteine biosynthetic process | GO:0019344 | 0.000082 |
| nitrate assimilation | GO:0042128 | 0.0001 |
| calcium ion transmembrane transport | GO:0070588 | 0.00011 |
| RNA-dependent DNA replication | GO:0006278 | 0.00013 |
| response to jasmonic acid | GO:0009753 | 0.00014 |
| water transport | GO:0006833 | 0.00015 |
| regulation of proton transport | GO:0010155 | 0.00015 |
| carbohydrate metabolic process | GO:0005975 | 0.00016 |
| phototropism | GO:0009638 | 0.00023 |
| cell wall organization | GO:0071555 | 0.00024 |
| photosynthetic electron transport in photosystem I | GO:0009773 | 0.00028 |
| pollen tube development | GO:0048868 | 0.00031 |
| amine transport | GO:0015837 | 0.00044 |
| cell tip growth | GO:0009932 | 0.00045 |
| response to salt stress | GO:0009651 | 0.00047 |
| response to sucrose | GO:0009744 | 0.00051 |
| glucosinolate biosynthetic process | GO:0019761 | 0.0006 |
| ion transport | GO:0006811 | 0.00064 |
| chlorophyll biosynthetic process | GO:0015995 | 0.00064 |
| response to high light intensity | GO:0009644 | 0.00065 |
| protein-chromophore linkage | GO:0018298 | 0.00077 |
| chloroplast relocation | GO:0009902 | 0.00078 |
| defense response to bacterium | GO:0042742 | 0.00092 |
| transmembrane transport | GO:0055085 | 0.00093 |
| response to endoplasmic reticulum stress | GO:0034976 | 0.00101 |
| regulation of pollen tube growth | GO:0080092 | 0.0011 |
| photosynthesis, light harvesting | GO:0009765 | 0.00111 |
| photosystem II assembly | GO:0010207 | 0.00113 |
| metal ion transport | GO:0030001 | 0.00123 |
| cellular calcium ion homeostasis | GO:0006874 | 0.00124 |
| drug transmembrane transport | GO:0006855 | 0.00168 |
| single-organism transport | GO:0044765 | 0.00188 |
| reductive pentose-phosphate cycle | GO:0019253 | 0.00196 |
| ATP biosynthetic process | GO:0006754 | 0.00199 |
| carboxylic acid metabolic process | GO:0019752 | 0.00224 |
| glutamate biosynthetic process | GO:0006537 | 0.0023 |
| pentose-phosphate shunt | GO:0006098 | 0.00249 |
| response to nematode | GO:0009624 | 0.00252 |
| response to light intensity | GO:0009642 | 0.00258 |
| unsaturated fatty acid biosynthetic process | GO:0006636 | 0.00262 |
| systemic acquired resistance | GO:0009627 | 0.00265 |
| auxin metabolic process | GO:0009850 | 0.00328 |
| photosynthesis, light reaction | GO:0019684 | 0.00328 |
| organonitrogen compound biosynthetic process | GO:1901566 | 0.0035 |
| syncytium formation | GO:0006949 | 0.00382 |
| nonphotochemical quenching | GO:0010196 | 0.00383 |
| blue light signaling pathway | GO:0009785 | 0.00397 |
| regulation of hydrogen peroxide metabolic process | GO:0010310 | 0.00404 |
| electron transport chain | GO:0022900 | 0.00422 |
| response to auxin | GO:0009733 | 0.00431 |
| flavonol biosynthetic process | GO:0051555 | 0.00434 |
| carotene biosynthetic process | GO:0016120 | 0.00447 |
| carboxylic acid transport | GO:0046942 | 0.00447 |
| urea transmembrane transport | GO:0071918 | 0.00447 |
| PSII associated light-harvesting complex II catabolic process | GO:0010304 | 0.00458 |
| response to oxygen-containing compound | GO:1901700 | 0.00468 |
| anthocyanin accumulation in tissues in response to UV light | GO:0043481 | 0.00487 |
| photorespiration | GO:0009853 | 0.00525 |
| response to heat | GO:0009408 | 0.00529 |
| chemical homeostasis | GO:0048878 | 0.00533 |
| systemic acquired resistance, salicylic acid mediated signaling pathway | GO:0009862 | 0.00546 |
| stomatal complex morphogenesis | GO:0010103 | 0.00561 |
| hormone-mediated signaling pathway | GO:0009755 | 0.00566 |
| trehalose metabolic process | GO:0005991 | 0.00578 |
| positive gravitropism | GO:0009958 | 0.00618 |
| response to cadmium ion | GO:0046686 | 0.00655 |
| microsporogenesis | GO:0009556 | 0.00688 |
| tryptophan catabolic process | GO:0006569 | 0.00689 |
| ionotropic glutamate receptor signaling pathway | GO:0035235 | 0.0073 |
| response to chitin | GO:0010200 | 0.00735 |
| ER-nucleus signaling pathway | GO:0006984 | 0.00748 |
| sucrose metabolic process | GO:0005985 | 0.00783 |
| ammonia assimilation cycle | GO:0019676 | 0.00793 |
| one-carbon metabolic process | GO:0006730 | 0.00823 |
| regulation of protein dephosphorylation | GO:0035304 | 0.00834 |
| intracellular protein transport | GO:0006886 | 0.00836 |
| ammonium transmembrane transport | GO:0072488 | 0.00839 |
| aerobic respiration | GO:0009060 | 0.00845 |
| plastid organization | GO:0009657 | 0.00901 |
| glycerol ether metabolic process | GO:0006662 | 0.00918 |
| jasmonic acid metabolic process | GO:0009694 | 0.00987 |
| fruit ripening | GO:0009835 | 0.01022 |
| negative regulation of catalytic activity | GO:0043086 | 0.01032 |
| spermidine biosynthetic process | GO:0008295 | 0.01041 |
| anion homeostasis | GO:0055081 | 0.01068 |
| callose deposition in phloem sieve plate | GO:0080165 | 0.01079 |
| amino acid transport | GO:0006865 | 0.01097 |
| extracellular polysaccharide biosynthetic process | GO:0045226 | 0.01102 |
| response to wounding | GO:0009611 | 0.01186 |
| inorganic anion transport | GO:0015698 | 0.01207 |
| cell wall modification | GO:0042545 | 0.01215 |
| endoplasmic reticulum unfolded protein response | GO:0030968 | 0.01222 |
| trehalose biosynthetic process | GO:0005992 | 0.0124 |
| dicarboxylic acid transport | GO:0006835 | 0.0125 |
| isopentenyl diphosphate biosynthetic process, methylerythritol 4-phosphate pathway | GO:0019288 | 0.01292 |
| cytoplasmic transport | GO:0016482 | 0.01299 |
| pollen tube growth | GO:0009860 | 0.01373 |
| response to gibberellin | GO:0009739 | 0.014 |
| cold acclimation | GO:0009631 | 0.01443 |
| response to hexose | GO:0009746 | 0.01453 |
| regulation of plant-type hypersensitive response | GO:0010363 | 0.01523 |
| polyamine biosynthetic process | GO:0006596 | 0.01524 |
| polysaccharide metabolic process | GO:0005976 | 0.01539 |
| hydrogen peroxide catabolic process | GO:0042744 | 0.01555 |
| tricarboxylic acid cycle | GO:0006099 | 0.01569 |
| pectin catabolic process | GO:0045490 | 0.01679 |
| lipid metabolic process | GO:0006629 | 0.01774 |
| amino acid transmembrane transport | GO:0003333 | 0.01786 |
| photosynthesis | GO:0015979 | 0.01804 |
| phosphate ion transport | GO:0006817 | 0.01886 |
| arginine biosynthetic process | GO:0006526 | 0.01915 |
| indoleacetic acid biosynthetic process | GO:0009684 | 0.01937 |
| jasmonic acid mediated signaling pathway | GO:0009867 | 0.01976 |
| divalent metal ion transport | GO:0070838 | 0.01993 |
| response to fructose | GO:0009750 | 0.02048 |
| translation | GO:0006412 | 0.02099 |
| vesicle-mediated transport | GO:0016192 | 0.02181 |
| cation transport | GO:0006812 | 0.02199 |
| lipid biosynthetic process | GO:0008610 | 0.02219 |
| regulation of lipid metabolic process | GO:0019216 | 0.02287 |
| cellular lipid metabolic process | GO:0044255 | 0.02316 |
| salicylic acid biosynthetic process | GO:0009697 | 0.02385 |
| actin filament depolymerization | GO:0030042 | 0.02402 |
| cellular macromolecule catabolic process | GO:0044265 | 0.02413 |
| auxin biosynthetic process | GO:0009851 | 0.02418 |
| carbohydrate transport | GO:0008643 | 0.02437 |
| carbohydrate derivative biosynthetic process | GO:1901137 | 0.02472 |
| cellular homeostasis | GO:0019725 | 0.02483 |
| small molecule biosynthetic process | GO:0044283 | 0.02483 |
| response to water | GO:0009415 | 0.02489 |
| phospholipid metabolic process | GO:0006644 | 0.02498 |
| detection of stimulus | GO:0051606 | 0.02599 |
| cellular response to hydrogen peroxide | GO:0070301 | 0.02652 |
| ribosome biogenesis | GO:0042254 | 0.02669 |
| stomatal movement | GO:0010118 | 0.02681 |
| xylem development | GO:0010089 | 0.02702 |
| pentose-phosphate shunt, oxidative branch | GO:0009051 | 0.0289 |
| detection of biotic stimulus | GO:0009595 | 0.02938 |
| salicylic acid metabolic process | GO:0009696 | 0.02938 |
| cell wall thickening | GO:0052386 | 0.03013 |
| phospholipid biosynthetic process | GO:0008654 | 0.0305 |
| protein catabolic process | GO:0030163 | 0.03069 |
| carboxylic acid biosynthetic process | GO:0046394 | 0.03086 |
| regulation of flavonoid biosynthetic process | GO:0009962 | 0.03122 |
| carbon fixation | GO:0015977 | 0.03138 |
| spermine biosynthetic process | GO:0006597 | 0.03145 |
| response to fungus | GO:0009620 | 0.03224 |
| protein tetramerization | GO:0051262 | 0.0323 |
| response to absence of light | GO:0009646 | 0.0328 |
| photosynthetic electron transport chain | GO:0009767 | 0.03297 |
| primary root development | GO:0080022 | 0.03301 |
| cellular ion homeostasis | GO:0006873 | 0.03323 |
| cellular amino acid metabolic process | GO:0006520 | 0.0333 |
| cellular cation homeostasis | GO:0030003 | 0.03429 |
| regulation of programmed cell death | GO:0043067 | 0.03534 |
| proteolysis involved in cellular protein catabolic process | GO:0051603 | 0.03549 |
| flower morphogenesis | GO:0048439 | 0.03642 |
| response to water deprivation | GO:0009414 | 0.03743 |
| response to abiotic stimulus | GO:0009628 | 0.03821 |
| glutamine biosynthetic process | GO:0006542 | 0.03823 |
| nucleotide-sugar metabolic process | GO:0009225 | 0.03936 |
| UDP-rhamnose biosynthetic process | GO:0010253 | 0.04014 |
| hyperosmotic response | GO:0006972 | 0.04066 |
| photosynthetic electron transport in photosystem II | GO:0009772 | 0.04096 |
| response to light stimulus | GO:0009416 | 0.04159 |
| regulation of cyclin-dependent protein serine/threonine kinase activity | GO:0000079 | 0.04233 |
| sucrose transport | GO:0015770 | 0.04266 |
| protein targeting | GO:0006605 | 0.04316 |
| glycine catabolic process | GO:0006546 | 0.04337 |
| photoinhibition | GO:0010205 | 0.04378 |
| callose deposition in cell wall | GO:0052543 | 0.04401 |
| monocarboxylic acid biosynthetic process | GO:0072330 | 0.04407 |
| regulation of innate immune response | GO:0045088 | 0.04472 |
| acidic amino acid transport | GO:0015800 | 0.04517 |
| macromolecule catabolic process | GO:0009057 | 0.046 |
| starch biosynthetic process | GO:0019252 | 0.04643 |
| rRNA processing | GO:0006364 | 0.04655 |
| purine nucleobase biosynthetic process | GO:0009113 | 0.04662 |
| jasmonic acid biosynthetic process | GO:0009695 | 0.04704 |
| phosphorelay signal transduction system | GO:0000160 | 0.04781 |
| signal transduction by phosphorylation | GO:0023014 | 0.04868 |
| potassium ion transport | GO:0006813 | 0.04874 |
| multidimensional cell growth | GO:0009825 | 0.04895 |
| cellular amino acid biosynthetic process | GO:0008652 | 0.04918 |
| starch catabolic process | GO:0005983 | 0.04966 |
| GO description (JinIP_JinST) | GO term | P value |
| oxidation-reduction process | GO:0055114 | 4E-11 |
| plant-type cell wall modification | GO:0009827 | 3.8E-09 |
| DNA integration | GO:0015074 | 1.4E-08 |
| glycolysis | GO:0006096 | 4.1E-08 |
| response to red light | GO:0010114 | 2.1E-07 |
| response to far red light | GO:0010218 | 4.8E-07 |
| transmembrane transport | GO:0055085 | 5.3E-07 |
| cysteine biosynthetic process | GO:0019344 | 1.5E-06 |
| fatty acid biosynthetic process | GO:0006633 | 3.9E-06 |
| response to blue light | GO:0009637 | 4.2E-06 |
| calcium ion transmembrane transport | GO:0070588 | 0.000005 |
| photorespiration | GO:0009853 | 6.9E-06 |
| electron transport chain | GO:0022900 | 0.000011 |
| water transport | GO:0006833 | 0.000019 |
| oxylipin biosynthetic process | GO:0031408 | 0.000035 |
| response to cadmium ion | GO:0046686 | 0.000037 |
| single-organism transport | GO:0044765 | 0.000047 |
| proton transport | GO:0015992 | 0.00012 |
| ATP biosynthetic process | GO:0006754 | 0.00013 |
| cell wall organization | GO:0071555 | 0.00021 |
| chloroplast relocation | GO:0009902 | 0.00024 |
| pollen tube growth | GO:0009860 | 0.00027 |
| response to desiccation | GO:0009269 | 0.0003 |
| sucrose metabolic process | GO:0005985 | 0.00034 |
| pentose-phosphate shunt | GO:0006098 | 0.00034 |
| flavonol biosynthetic process | GO:0051555 | 0.00039 |
| photosynthesis | GO:0015979 | 0.00051 |
| photosynthesis, light reaction | GO:0019684 | 0.00057 |
| amine transport | GO:0015837 | 0.00058 |
| glycine catabolic process | GO:0006546 | 0.00059 |
| metal ion transport | GO:0030001 | 0.00059 |
| monovalent inorganic cation transport | GO:0015672 | 0.0006 |
| response to salt stress | GO:0009651 | 0.00069 |
| chlorophyll biosynthetic process | GO:0015995 | 0.00075 |
| regulation of protein dephosphorylation | GO:0035304 | 0.00083 |
| carbohydrate metabolic process | GO:0005975 | 0.00094 |
| RNA-dependent DNA replication | GO:0006278 | 0.001 |
| positive gravitropism | GO:0009958 | 0.001 |
| carbohydrate transport | GO:0008643 | 0.00101 |
| endoplasmic reticulum unfolded protein response | GO:0030968 | 0.00107 |
| oligopeptide transport | GO:0006857 | 0.00112 |
| cell tip growth | GO:0009932 | 0.0012 |
| response to jasmonic acid | GO:0009753 | 0.00125 |
| PSII associated light-harvesting complex II catabolic process | GO:0010304 | 0.00129 |
| anion homeostasis | GO:0055081 | 0.00129 |
| regulation of plant-type hypersensitive response | GO:0010363 | 0.00134 |
| extracellular polysaccharide biosynthetic process | GO:0045226 | 0.00136 |
| cation transport | GO:0006812 | 0.0014 |
| regulation of pollen tube growth | GO:0080092 | 0.00142 |
| unsaturated fatty acid biosynthetic process | GO:0006636 | 0.00169 |
| response to high light intensity | GO:0009644 | 0.00171 |
| pectin catabolic process | GO:0045490 | 0.00171 |
| response to sucrose | GO:0009744 | 0.00179 |
| auxin efflux | GO:0010315 | 0.00192 |
| defense response to bacterium | GO:0042742 | 0.0022 |
| organonitrogen compound biosynthetic process | GO:1901566 | 0.00227 |
| anion transport | GO:0006820 | 0.00235 |
| proteasome core complex assembly | GO:0080129 | 0.00241 |
| response to nematode | GO:0009624 | 0.00266 |
| one-carbon metabolic process | GO:0006730 | 0.00301 |
| photosystem II assembly | GO:0010207 | 0.00306 |
| response to karrikin | GO:0080167 | 0.00307 |
| hyperosmotic salinity response | GO:0042538 | 0.00314 |
| response to fructose | GO:0009750 | 0.00322 |
| phenylpropanoid biosynthetic process | GO:0009699 | 0.00333 |
| aerobic respiration | GO:0009060 | 0.0035 |
| syncytium formation | GO:0006949 | 0.00356 |
| regulation of proton transport | GO:0010155 | 0.00357 |
| fructose 6-phosphate metabolic process | GO:0006002 | 0.00407 |
| root hair elongation | GO:0048767 | 0.00447 |
| photosynthetic electron transport in photosystem I | GO:0009773 | 0.00468 |
| phosphate ion transport | GO:0006817 | 0.00502 |
| photosynthesis, light harvesting | GO:0009765 | 0.00526 |
| amino acid transmembrane transport | GO:0003333 | 0.00528 |
| (1->3)-beta-D-glucan biosynthetic process | GO:0006075 | 0.0053 |
| nonphotochemical quenching | GO:0010196 | 0.0053 |
| UDP-rhamnose biosynthetic process | GO:0010253 | 0.00534 |
| systemic acquired resistance | GO:0009627 | 0.00548 |
| cellular calcium ion homeostasis | GO:0006874 | 0.00578 |
| carotene biosynthetic process | GO:0016120 | 0.00618 |
| proline biosynthetic process | GO:0006561 | 0.00627 |
| response to absence of light | GO:0009646 | 0.00629 |
| regulation of hydrogen peroxide metabolic process | GO:0010310 | 0.00686 |
| glucosinolate biosynthetic process | GO:0019761 | 0.0072 |
| systemic acquired resistance, salicylic acid mediated signaling pathway | GO:0009862 | 0.00722 |
| fruit ripening | GO:0009835 | 0.00751 |
| photoinhibition | GO:0010205 | 0.00764 |
| cell wall modification involved in multidimensional cell growth | GO:0042547 | 0.00777 |
| detection of biotic stimulus | GO:0009595 | 0.00813 |
| intracellular protein transport | GO:0006886 | 0.00846 |
| anthocyanin accumulation in tissues in response to UV light | GO:0043481 | 0.0085 |
| carotenoid biosynthetic process | GO:0016117 | 0.00864 |
| response to drug | GO:0042493 | 0.00903 |
| response to oxygen-containing compound | GO:1901700 | 0.00912 |
| carboxylic acid biosynthetic process | GO:0046394 | 0.00917 |
| pigment metabolic process | GO:0042440 | 0.00954 |
| protein targeting to membrane | GO:0006612 | 0.00964 |
| response to hormone | GO:0009725 | 0.00978 |
| jasmonic acid metabolic process | GO:0009694 | 0.00997 |
| drug transmembrane transport | GO:0006855 | 0.01006 |
| anthocyanin-containing compound biosynthetic process | GO:0009718 | 0.0102 |
| acidic amino acid transport | GO:0015800 | 0.01051 |
| acetyl-CoA biosynthetic process | GO:0006085 | 0.01079 |
| plant-type cell wall organization | GO:0009664 | 0.01082 |
| response to organonitrogen compound | GO:0010243 | 0.01157 |
| ammonium transmembrane transport | GO:0072488 | 0.01181 |
| spermidine biosynthetic process | GO:0008295 | 0.01182 |
| regulation of cell size | GO:0008361 | 0.01184 |
| tryptophan metabolic process | GO:0006568 | 0.01204 |
| response to misfolded protein | GO:0051788 | 0.01254 |
| ionotropic glutamate receptor signaling pathway | GO:0035235 | 0.0128 |
| sucrose biosynthetic process | GO:0005986 | 0.01315 |
| tryptophan catabolic process | GO:0006569 | 0.0136 |
| nitrogen compound transport | GO:0071705 | 0.01373 |
| potassium ion transport | GO:0006813 | 0.01381 |
| ammonium transport | GO:0015696 | 0.01408 |
| urea transmembrane transport | GO:0071918 | 0.01421 |
| cold acclimation | GO:0009631 | 0.01491 |
| response to endoplasmic reticulum stress | GO:0034976 | 0.01512 |
| vitamin E biosynthetic process | GO:0010189 | 0.01514 |
| nitrate assimilation | GO:0042128 | 0.01526 |
| response to heat | GO:0009408 | 0.01592 |
| lipoate metabolic process | GO:0009106 | 0.01598 |
| response to chitin | GO:0010200 | 0.01625 |
| negative regulation of cell death | GO:0060548 | 0.01631 |
| lignin biosynthetic process | GO:0009809 | 0.01666 |
| starch catabolic process | GO:0005983 | 0.01674 |
| cytoplasmic transport | GO:0016482 | 0.01699 |
| monocarboxylic acid biosynthetic process | GO:0072330 | 0.01747 |
| polyamine biosynthetic process | GO:0006596 | 0.01753 |
| response to gibberellin | GO:0009739 | 0.01839 |
| auxin metabolic process | GO:0009850 | 0.01852 |
| arginine biosynthetic process | GO:0006526 | 0.01905 |
| inorganic anion transport | GO:0015698 | 0.01936 |
| translation | GO:0006412 | 0.02063 |
| ribosome biogenesis | GO:0042254 | 0.02075 |
| ATP synthesis coupled electron transport | GO:0042773 | 0.02153 |
| sulfur compound metabolic process | GO:0006790 | 0.02156 |
| carboxylic acid transport | GO:0046942 | 0.02163 |
| ATP synthesis coupled proton transport | GO:0015986 | 0.02182 |
| carbon fixation | GO:0015977 | 0.0224 |
| isopentenyl diphosphate biosynthetic process, methylerythritol 4-phosphate pathway | GO:0019288 | 0.0224 |
| phosphate ion transmembrane transport | GO:0035435 | 0.02243 |
| salicylic acid biosynthetic process | GO:0009697 | 0.02258 |
| potassium ion transmembrane transport | GO:0071805 | 0.0229 |
| negative regulation of programmed cell death | GO:0043069 | 0.02442 |
| regulation of lipid metabolic process | GO:0019216 | 0.02468 |
| sucrose transport | GO:0015770 | 0.02522 |
| monocarboxylic acid metabolic process | GO:0032787 | 0.02559 |
| starch biosynthetic process | GO:0019252 | 0.02642 |
| regulation of hormone levels | GO:0010817 | 0.02653 |
| tricarboxylic acid cycle | GO:0006099 | 0.02703 |
| pollen germination | GO:0009846 | 0.02756 |
| vesicle-mediated transport | GO:0016192 | 0.02803 |
| salicylic acid metabolic process | GO:0009696 | 0.02926 |
| lipid metabolic process | GO:0006629 | 0.02949 |
| jasmonic acid mediated signaling pathway | GO:0009867 | 0.02983 |
| response to lipid | GO:0033993 | 0.03108 |
| plant-type cell wall modification involved in multidimensional cell growth | GO:0009831 | 0.03132 |
| response to wounding | GO:0009611 | 0.03153 |
| organic anion transport | GO:0015711 | 0.03249 |
| glutamate biosynthetic process | GO:0006537 | 0.0325 |
| sulfur compound biosynthetic process | GO:0044272 | 0.03259 |
| small molecule metabolic process | GO:0044281 | 0.03322 |
| cellular macromolecule catabolic process | GO:0044265 | 0.03374 |
| indoleacetic acid biosynthetic process | GO:0009684 | 0.03375 |
| maltose metabolic process | GO:0000023 | 0.03378 |
| tripeptide transport | GO:0042939 | 0.03494 |
| oxidative phosphorylation | GO:0006119 | 0.03498 |
| indole-containing compound metabolic process | GO:0042430 | 0.03557 |
| cellular lipid metabolic process | GO:0044255 | 0.03567 |
| cellular amino acid biosynthetic process | GO:0008652 | 0.03573 |
| pigment biosynthetic process | GO:0046148 | 0.03603 |
| oligosaccharide transport | GO:0015772 | 0.03693 |
| regulation of stomatal movement | GO:0010119 | 0.03771 |
| pentose-phosphate shunt, oxidative branch | GO:0009051 | 0.03845 |
| auxin polar transport | GO:0009926 | 0.0392 |
| xylem development | GO:0010089 | 0.03954 |
| spermine biosynthetic process | GO:0006597 | 0.04018 |
| gibberellic acid mediated signaling pathway | GO:0009740 | 0.04164 |
| stomatal movement | GO:0010118 | 0.04164 |
| protein catabolic process | GO:0030163 | 0.04191 |
| flavonoid biosynthetic process | GO:0009813 | 0.04257 |
| carbohydrate derivative biosynthetic process | GO:1901137 | 0.04313 |
| manganese ion homeostasis | GO:0055071 | 0.04325 |
| cellular aldehyde metabolic process | GO:0006081 | 0.0435 |
| blue light signaling pathway | GO:0009785 | 0.0435 |
| regulation of multi-organism process | GO:0043900 | 0.04373 |
| jasmonic acid biosynthetic process | GO:0009695 | 0.04385 |
| cellular homeostasis | GO:0019725 | 0.04431 |
| nitrate transport | GO:0015706 | 0.04492 |
| macromolecule catabolic process | GO:0009057 | 0.04517 |
| trehalose metabolic process | GO:0005991 | 0.04577 |
| response to abscisic acid | GO:0009737 | 0.04587 |
| lipid biosynthetic process | GO:0008610 | 0.04683 |
| response to temperature stimulus | GO:0009266 | 0.04737 |
| L-phenylalanine catabolic process | GO:0006559 | 0.04792 |
| phospholipid biosynthetic process | GO:0008654 | 0.04807 |
| response to auxin | GO:0009733 | 0.04875 |
| pollen exine formation | GO:0010584 | 0.049 |
| organic cation transport | GO:0015695 | 0.04952 |
| ion transport | GO:0006811 | 0.04967 |
| GO description (JinSE_JinST) | GO term | P value |
| oxidation-reduction process | GO:0055114 | 3.5E-24 |
| response to far red light | GO:0010218 | 5.7E-17 |
| response to red light | GO:0010114 | 5.1E-14 |
| carotenoid biosynthetic process | GO:0016117 | 5.9E-14 |
| response to blue light | GO:0009637 | 3.6E-13 |
| photosystem II assembly | GO:0010207 | 1E-09 |
| photosynthetic electron transport in photosystem I | GO:0009773 | 2.8E-09 |
| oxylipin biosynthetic process | GO:0031408 | 2.8E-08 |
| chlorophyll biosynthetic process | GO:0015995 | 3.2E-08 |
| pentose-phosphate shunt | GO:0006098 | 4.4E-08 |
| fatty acid biosynthetic process | GO:0006633 | 5.4E-08 |
| chloroplast relocation | GO:0009902 | 2.7E-07 |
| photosynthesis | GO:0015979 | 3.8E-07 |
| defense response to bacterium | GO:0042742 | 5.7E-07 |
| isopentenyl diphosphate biosynthetic process, methylerythritol 4-phosphate pathway | GO:0019288 | 7.5E-07 |
| glycolysis | GO:0006096 | 7.7E-07 |
| regulation of proton transport | GO:0010155 | 2.1E-06 |
| response to sucrose | GO:0009744 | 2.2E-06 |
| response to wounding | GO:0009611 | 4.2E-06 |
| DNA integration | GO:0015074 | 6.7E-06 |
| carbohydrate metabolic process | GO:0005975 | 7.4E-06 |
| regulation of protein dephosphorylation | GO:0035304 | 9.9E-06 |
| (1->3)-beta-D-glucan biosynthetic process | GO:0006075 | 0.000017 |
| plant-type cell wall modification | GO:0009827 | 0.000018 |
| flavonol biosynthetic process | GO:0051555 | 0.000019 |
| water transport | GO:0006833 | 0.000023 |
| photosynthesis, light harvesting | GO:0009765 | 0.000024 |
| extracellular polysaccharide biosynthetic process | GO:0045226 | 0.000032 |
| response to salt stress | GO:0009651 | 0.000049 |
| stomatal movement | GO:0010118 | 0.000052 |
| unsaturated fatty acid biosynthetic process | GO:0006636 | 0.000064 |
| glucosinolate biosynthetic process | GO:0019761 | 0.000076 |
| cell tip growth | GO:0009932 | 0.000082 |
| UDP-rhamnose biosynthetic process | GO:0010253 | 0.000084 |
| glutamate biosynthetic process | GO:0006537 | 0.000088 |
| rRNA processing | GO:0006364 | 0.000099 |
| response to karrikin | GO:0080167 | 0.00011 |
| pectin catabolic process | GO:0045490 | 0.00018 |
| phenylpropanoid biosynthetic process | GO:0009699 | 0.00019 |
| cysteine biosynthetic process | GO:0019344 | 0.00021 |
| protein-chromophore linkage | GO:0018298 | 0.00022 |
| oligopeptide transport | GO:0006857 | 0.00023 |
| hydrogen peroxide catabolic process | GO:0042744 | 0.00026 |
| transmembrane transport | GO:0055085 | 0.00026 |
| ATP biosynthetic process | GO:0006754 | 0.00028 |
| microsporogenesis | GO:0009556 | 0.00028 |
| nitrate assimilation | GO:0042128 | 0.00037 |
| anthocyanin accumulation in tissues in response to UV light | GO:0043481 | 0.00043 |
| single-organism transport | GO:0044765 | 0.00044 |
| drug transmembrane transport | GO:0006855 | 0.00046 |
| callose deposition in cell wall | GO:0052543 | 0.00047 |
| amine transport | GO:0015837 | 0.00059 |
| reductive pentose-phosphate cycle | GO:0019253 | 0.00059 |
| signal transduction by phosphorylation | GO:0023014 | 0.00059 |
| calcium ion transmembrane transport | GO:0070588 | 0.00059 |
| syncytium formation | GO:0006949 | 0.00061 |
| cuticle development | GO:0042335 | 0.00073 |
| thylakoid membrane organization | GO:0010027 | 0.00085 |
| systemic acquired resistance, salicylic acid mediated signaling pathway | GO:0009862 | 0.00091 |
| pollen tube development | GO:0048868 | 0.00092 |
| response to jasmonic acid | GO:0009753 | 0.00096 |
| PSII associated light-harvesting complex II catabolic process | GO:0010304 | 0.00107 |
| electron transport chain | GO:0022900 | 0.00108 |
| response to cadmium ion | GO:0046686 | 0.0011 |
| iron ion transport | GO:0006826 | 0.00112 |
| response to fructose | GO:0009750 | 0.0015 |
| regulation of hydrogen peroxide metabolic process | GO:0010310 | 0.00164 |
| jasmonic acid metabolic process | GO:0009694 | 0.00198 |
| salicylic acid biosynthetic process | GO:0009697 | 0.00206 |
| cellular response to iron ion starvation | GO:0010106 | 0.00209 |
| lignin catabolic process | GO:0046274 | 0.00212 |
| urea transmembrane transport | GO:0071918 | 0.00223 |
| root hair elongation | GO:0048767 | 0.0023 |
| jasmonic acid mediated signaling pathway | GO:0009867 | 0.00254 |
| response to light intensity | GO:0009642 | 0.00258 |
| regulation of pollen tube growth | GO:0080092 | 0.00266 |
| flavonoid biosynthetic process | GO:0009813 | 0.0028 |
| stomatal complex morphogenesis | GO:0010103 | 0.00289 |
| plastid organization | GO:0009657 | 0.00306 |
| response to water deprivation | GO:0009414 | 0.00316 |
| regulation of cell size | GO:0008361 | 0.00321 |
| nitrate transport | GO:0015706 | 0.00331 |
| response to abscisic acid | GO:0009737 | 0.00333 |
| auxin efflux | GO:0010315 | 0.00344 |
| RNA-dependent DNA replication | GO:0006278 | 0.00353 |
| cell wall organization | GO:0071555 | 0.00357 |
| response to nematode | GO:0009624 | 0.00361 |
| photosynthesis, light reaction | GO:0019684 | 0.00364 |
| nonphotochemical quenching | GO:0010196 | 0.00371 |
| regulation of plant-type hypersensitive response | GO:0010363 | 0.00399 |
| cellular calcium ion homeostasis | GO:0006874 | 0.00405 |
| starch biosynthetic process | GO:0019252 | 0.00413 |
| detection of biotic stimulus | GO:0009595 | 0.00428 |
| regulation of lipid metabolic process | GO:0019216 | 0.00436 |
| amino acid transmembrane transport | GO:0003333 | 0.00443 |
| carotene biosynthetic process | GO:0016120 | 0.00475 |
| photorespiration | GO:0009853 | 0.00476 |
| lipid metabolic process | GO:0006629 | 0.00489 |
| sucrose metabolic process | GO:0005985 | 0.00509 |
| small molecule metabolic process | GO:0044281 | 0.00598 |
| ammonia assimilation cycle | GO:0019676 | 0.00626 |
| proline biosynthetic process | GO:0006561 | 0.00642 |
| folic acid transport | GO:0015884 | 0.00679 |
| negative regulation of defense response | GO:0031348 | 0.00735 |
| divalent metal ion transport | GO:0070838 | 0.00739 |
| photoinhibition | GO:0010205 | 0.00766 |
| intracellular protein transport | GO:0006886 | 0.00771 |
| response to endoplasmic reticulum stress | GO:0034976 | 0.00796 |
| photosynthetic electron transport in photosystem II | GO:0009772 | 0.00819 |
| acetyl-CoA biosynthetic process | GO:0006085 | 0.00863 |
| carbohydrate transport | GO:0008643 | 0.00882 |
| aerobic respiration | GO:0009060 | 0.00912 |
| coenzyme A metabolic process | GO:0015936 | 0.00962 |
| monovalent inorganic cation transport | GO:0015672 | 0.00985 |
| regulation of multi-organism process | GO:0043900 | 0.01015 |
| chemical homeostasis | GO:0048878 | 0.01052 |
| tricarboxylic acid cycle | GO:0006099 | 0.0106 |
| embryonic morphogenesis | GO:0048598 | 0.01098 |
| pollen tube growth | GO:0009860 | 0.01131 |
| transcription from plastid promoter | GO:0042793 | 0.01132 |
| response to biotic stimulus | GO:0009607 | 0.01175 |
| protein import into chloroplast thylakoid membrane | GO:0045038 | 0.01201 |
| one-carbon metabolic process | GO:0006730 | 0.01209 |
| pollen germination | GO:0009846 | 0.01227 |
| hyperosmotic response | GO:0006972 | 0.01245 |
| positive regulation of catalytic activity | GO:0043085 | 0.01263 |
| maltose metabolic process | GO:0000023 | 0.01366 |
| MAPK cascade | GO:0000165 | 0.01366 |
| regulation of stomatal movement | GO:0010119 | 0.01374 |
| cytoplasmic transport | GO:0016482 | 0.01382 |
| response to high light intensity | GO:0009644 | 0.01385 |
| response to auxin | GO:0009733 | 0.01386 |
| cellular lipid metabolic process | GO:0044255 | 0.01403 |
| response to absence of light | GO:0009646 | 0.01423 |
| phosphorelay signal transduction system | GO:0000160 | 0.01432 |
| organonitrogen compound biosynthetic process | GO:1901566 | 0.0144 |
| positive gravitropism | GO:0009958 | 0.01472 |
| negative regulation of catalytic activity | GO:0043086 | 0.01537 |
| cellular response to light stimulus | GO:0071482 | 0.01555 |
| fruit ripening | GO:0009835 | 0.01617 |
| cellular response to phosphate starvation | GO:0016036 | 0.01629 |
| response to heat | GO:0009408 | 0.01638 |
| blue light signaling pathway | GO:0009785 | 0.01661 |
| tripeptide transport | GO:0042939 | 0.01662 |
| anthocyanin-containing compound biosynthetic process | GO:0009718 | 0.01663 |
| anion homeostasis | GO:0055081 | 0.0167 |
| lipid biosynthetic process | GO:0008610 | 0.01689 |
| trehalose metabolic process | GO:0005991 | 0.01694 |
| phosphate ion transport | GO:0006817 | 0.01749 |
| callose deposition in phloem sieve plate | GO:0080165 | 0.01772 |
| inorganic anion transport | GO:0015698 | 0.01786 |
| phosphatidylinositol dephosphorylation | GO:0046856 | 0.01945 |
| hyperosmotic salinity response | GO:0042538 | 0.01955 |
| jasmonic acid biosynthetic process | GO:0009695 | 0.01963 |
| ion transport | GO:0006811 | 0.02115 |
| arginine biosynthetic process | GO:0006526 | 0.02165 |
| sucrose transport | GO:0015770 | 0.02176 |
| L-phenylalanine catabolic process | GO:0006559 | 0.02207 |
| carbon fixation | GO:0015977 | 0.02207 |
| glycine catabolic process | GO:0006546 | 0.02244 |
| protein targeting to membrane | GO:0006612 | 0.02315 |
| catabolic process | GO:0009056 | 0.02346 |
| vesicle-mediated transport | GO:0016192 | 0.02348 |
| galactose transport | GO:0015757 | 0.02359 |
| coumarin biosynthetic process | GO:0009805 | 0.02361 |
| defense response to fungus | GO:0050832 | 0.02384 |
| very long-chain fatty acid metabolic process | GO:0000038 | 0.02461 |
| monocarboxylic acid metabolic process | GO:0032787 | 0.0251 |
| translation | GO:0006412 | 0.02579 |
| pigment biosynthetic process | GO:0046148 | 0.02589 |
| oligosaccharide metabolic process | GO:0009311 | 0.0267 |
| glutamine biosynthetic process | GO:0006542 | 0.02719 |
| multidimensional cell growth | GO:0009825 | 0.02767 |
| glycine metabolic process | GO:0006544 | 0.02779 |
| regulation of catalytic activity | GO:0050790 | 0.02799 |
| response to fungus | GO:0009620 | 0.02821 |
| pigment metabolic process | GO:0042440 | 0.02886 |
| response to gibberellin | GO:0009739 | 0.02904 |
| xylan catabolic process | GO:0045493 | 0.03027 |
| myo-inositol hexakisphosphate biosynthetic process | GO:0010264 | 0.03029 |
| response to nitrate | GO:0010167 | 0.03078 |
| carboxylic acid metabolic process | GO:0019752 | 0.03092 |
| calcium ion transport | GO:0006816 | 0.03127 |
| polyamine biosynthetic process | GO:0006596 | 0.03145 |
| acidic amino acid transport | GO:0015800 | 0.03152 |
| oligosaccharide transport | GO:0015772 | 0.03196 |
| phototropism | GO:0009638 | 0.0321 |
| ionotropic glutamate receptor signaling pathway | GO:0035235 | 0.03222 |
| trehalose biosynthetic process | GO:0005992 | 0.03269 |
| defense response by callose deposition | GO:0052542 | 0.03429 |
| response to arsenic-containing substance | GO:0046685 | 0.03443 |
| cinnamic acid biosynthetic process | GO:0009800 | 0.03487 |
| ATP synthesis coupled proton transport | GO:0015986 | 0.03589 |
| protein targeting | GO:0006605 | 0.03597 |
| plant-type cell wall biogenesis | GO:0009832 | 0.0374 |
| defense response by callose deposition in cell wall | GO:0052544 | 0.03773 |
| serine family amino acid metabolic process | GO:0009069 | 0.04002 |
| plant-type cell wall modification involved in multidimensional cell growth | GO:0009831 | 0.04013 |
| zinc ion transport | GO:0006829 | 0.04028 |
| phenylpropanoid metabolic process | GO:0009698 | 0.0404 |
| phosphatidylglycerol biosynthetic process | GO:0006655 | 0.04106 |
| cellular catabolic process | GO:0044248 | 0.04226 |
| purine nucleobase biosynthetic process | GO:0009113 | 0.04293 |
| lipoate metabolic process | GO:0009106 | 0.04336 |
| cell wall modification | GO:0042545 | 0.04338 |
| positive regulation of flavonoid biosynthetic process | GO:0009963 | 0.04445 |
| single-organism cellular process | GO:0044763 | 0.0452 |
| response to temperature stimulus | GO:0009266 | 0.04531 |
| manganese ion homeostasis | GO:0055071 | 0.04631 |
| regulation of cellular ketone metabolic process | GO:0010565 | 0.04685 |
| shade avoidance | GO:0009641 | 0.04745 |
| sulfur compound metabolic process | GO:0006790 | 0.0478 |
| protein autophosphorylation | GO:0046777 | 0.04903 |
| GO description (JinIP_JinOP) | GO term | P value |
| DNA integration | GO:0015074 | 1E-30 |
| RNA-dependent DNA replication | GO:0006278 | 4.5E-18 |
| organonitrogen compound biosynthetic process | GO:1901566 | 0.00026 |
| defense response to bacterium | GO:0042742 | 0.0004 |
| cellular lipid metabolic process | GO:0044255 | 0.00104 |
| intracellular protein transport | GO:0006886 | 0.00123 |
| lignin biosynthetic process | GO:0009809 | 0.00144 |
| lipid biosynthetic process | GO:0008610 | 0.00171 |
| response to heat | GO:0009408 | 0.00174 |
| response to abiotic stimulus | GO:0009628 | 0.00231 |
| carboxylic acid biosynthetic process | GO:0046394 | 0.00341 |
| cytoplasmic transport | GO:0016482 | 0.00347 |
| photosynthesis, light harvesting | GO:0009765 | 0.00377 |
| translation | GO:0006412 | 0.00384 |
| L-phenylalanine catabolic process | GO:0006559 | 0.00501 |
| ribosome biogenesis | GO:0042254 | 0.00576 |
| cofactor metabolic process | GO:0051186 | 0.00583 |
| cysteine biosynthetic process | GO:0019344 | 0.0064 |
| negative regulation of catalytic activity | GO:0043086 | 0.00656 |
| carbohydrate derivative biosynthetic process | GO:1901137 | 0.00692 |
| photosystem II assembly | GO:0010207 | 0.00725 |
| small molecule metabolic process | GO:0044281 | 0.00849 |
| cinnamic acid biosynthetic process | GO:0009800 | 0.00985 |
| xylem development | GO:0010089 | 0.0099 |
| oligopeptide transport | GO:0006857 | 0.0101 |
| response to light intensity | GO:0009642 | 0.01057 |
| monocarboxylic acid metabolic process | GO:0032787 | 0.01059 |
| carboxylic acid transport | GO:0046942 | 0.01124 |
| response to light stimulus | GO:0009416 | 0.01146 |
| phospholipid biosynthetic process | GO:0008654 | 0.01186 |
| organic substance transport | GO:0071702 | 0.01192 |
| response to nematode | GO:0009624 | 0.01249 |
| inorganic anion transport | GO:0015698 | 0.01256 |
| response to high light intensity | GO:0009644 | 0.01274 |
| pigment metabolic process | GO:0042440 | 0.01382 |
| pigment biosynthetic process | GO:0046148 | 0.01399 |
| rRNA processing | GO:0006364 | 0.01518 |
| chemical homeostasis | GO:0048878 | 0.01541 |
| protein targeting | GO:0006605 | 0.01615 |
| protein catabolic process | GO:0030163 | 0.0163 |
| carboxylic acid metabolic process | GO:0019752 | 0.01686 |
| photosynthetic electron transport chain | GO:0009767 | 0.01708 |
| zinc ion transmembrane transport | GO:0071577 | 0.01712 |
| isoprenoid biosynthetic process | GO:0008299 | 0.01778 |
| response to endogenous stimulus | GO:0009719 | 0.01817 |
| regulation of abscisic acid-activated signaling pathway | GO:0009787 | 0.01817 |
| positive regulation of abscisic acid-activated signaling pathway | GO:0009789 | 0.01831 |
| plant-type cell wall cellulose metabolic process | GO:0052541 | 0.01968 |
| NADP metabolic process | GO:0006739 | 0.02049 |
| tetrapyrrole biosynthetic process | GO:0033014 | 0.02094 |
| cellular macromolecule catabolic process | GO:0044265 | 0.02126 |
| sulfur compound metabolic process | GO:0006790 | 0.02181 |
| protein transport | GO:0015031 | 0.022 |
| indole-containing compound metabolic process | GO:0042430 | 0.0248 |
| glucose metabolic process | GO:0006006 | 0.02491 |
| RNA processing | GO:0006396 | 0.02533 |
| ncRNA metabolic process | GO:0034660 | 0.02575 |
| amino acid transmembrane transport | GO:0003333 | 0.02611 |
| cofactor biosynthetic process | GO:0051188 | 0.02629 |
| ionotropic glutamate receptor signaling pathway | GO:0035235 | 0.02652 |
| porphyrin-containing compound biosynthetic process | GO:0006779 | 0.02686 |
| fatty acid metabolic process | GO:0006631 | 0.02712 |
| pentose-phosphate shunt | GO:0006098 | 0.0272 |
| cellular amino acid biosynthetic process | GO:0008652 | 0.0276 |
| electron transport chain | GO:0022900 | 0.0276 |
| single-organism carbohydrate metabolic process | GO:0044723 | 0.02775 |
| cell wall modification | GO:0042545 | 0.02835 |
| mitochondrial transport | GO:0006839 | 0.02939 |
| catabolic process | GO:0009056 | 0.02948 |
| response to hormone | GO:0009725 | 0.03009 |
| photosynthetic electron transport in photosystem I | GO:0009773 | 0.0306 |
| response to metal ion | GO:0010038 | 0.03147 |
| cellular amino acid metabolic process | GO:0006520 | 0.03178 |
| phosphate ion transport | GO:0006817 | 0.0323 |
| response to inorganic substance | GO:0010035 | 0.03244 |
| response to radiation | GO:0009314 | 0.03348 |
| chromatin assembly or disassembly | GO:0006333 | 0.03376 |
| coenzyme metabolic process | GO:0006732 | 0.03408 |
| positive regulation of translational elongation | GO:0045901 | 0.03542 |
| vesicle-mediated transport | GO:0016192 | 0.03634 |
| regulation of cyclin-dependent protein serine/threonine kinase activity | GO:0000079 | 0.03651 |
| biological regulation | GO:0065007 | 0.03676 |
| oxidation-reduction process | GO:0055114 | 0.03701 |
| proteolysis involved in cellular protein catabolic process | GO:0051603 | 0.03737 |
| zinc ion transport | GO:0006829 | 0.03846 |
| lignin catabolic process | GO:0046274 | 0.03977 |
| phospholipid metabolic process | GO:0006644 | 0.03998 |
| response to cadmium ion | GO:0046686 | 0.04098 |
| amine metabolic process | GO:0009308 | 0.04148 |
| hexose metabolic process | GO:0019318 | 0.04159 |
| response to hydrogen peroxide | GO:0042542 | 0.04199 |
| single-organism transport | GO:0044765 | 0.04397 |
| photosynthetic electron transport in photosystem II | GO:0009772 | 0.04517 |
| protein localization | GO:0008104 | 0.04585 |
| ammonium transmembrane transport | GO:0072488 | 0.04683 |
| sulfur compound biosynthetic process | GO:0044272 | 0.04886 |
| glucose catabolic process | GO:0006007 | 0.04927 |
| amine transport | GO:0015837 | 0.04941 |
| organic anion transport | GO:0015711 | 0.04944 |

**Supplementary Dataset 2.** The mature and precursor sequences of miRNA identified in *C. japonica*.

**Mature sequences**

>conservative_c79576.graph_c0_44229

ggaaucuugaugaugcugcag

>conservative_c103602.graph_c0_4555

ugacgacgagagagagcacgc

>conservative_c66496.graph_c0_224200

ugagaugguuguguccgauuc

>conservative_c73855.graph_c0_358837

uggugaguucgggugagaua

>conservative_c115709.graph_c0_17842

ugguuugugcgaguuggaug

>conservative_c79710.graph_c0_445788

agacauuuuguucgacgguaguau

>conservative_c30045.graph_c0_54121

ugcacugccucuucccuggcu

>conservative_c71130.graph_c0_299430

accuggcuugugcgcguagcucuu

>conservative_c84089.graph_c0_454590

acuaaugcacugaccacucacaag

>conservative_c71127.graph_c0_299380

auuucauuuucgggguacuuu

>conservative_c64455.graph_c0_200162

uuuuguggucuucgcugugagaug

>conservative_c104868.graph_c0_5876

uguguugacgugaaacuaugaggu

>conservative_c75216.graph_c0_395182

uccucgguacuacccuauuug

>conservative_c66896.graph_c1_229677

aggagaguggccaaccuguugacuu

>conservative_c62103.graph_c0_176850

uugacagaagauagagagcac

>conservative_c51688.graph_c0_113067

agcugccgacucauucaucca

>conservative_c89015.graph_c0_463162

aaagcuguguggaaagccuugggu

>conservative_c76339.graph_c0_433671

acaaguagugguggagaagacaug

>conservative_c99069.graph_c0_477773

uuagugcuggguugugaaaggagc

>conservative_c20625.graph_c0_34424

gguugacggggcucguagccgg

>conservative_c64623.graph_c0_202034

ugaagcugccagcaugaucua

>conservative_c75679.graph_c0_408830

gcgugaucggauccaucuugaccc

>conservative_c69966.graph_c0_277690

acuuuccggccugucuucggc

>conservative_c54963.graph_c0_127636

uuuccgacuccgcccaugccau

>conservative_c65848.graph_c0_216092

aaggugugugaaacuaugugaaau

>conservative_c76638.graph_c0_437515

ggauucccggucuagcugauaaug

>conservative_c41939.graph_c0_80003

cugaaguguuugggggaacuc

>conservative_c79110.graph_c0_444311

aauuguggcgauuaaaagggguug

>conservative_c54625.graph_c0_126060

uuggguccugggggugugcucu

>conservative_c111357.graph_c0_13500

agaguuggacugagcaggcu

>conservative_c71130.graph_c0_299443

accuggcuugugcgcguagcucuu

>conservative_c45380.graph_c0_90682

auuggguuucagugggaauu

>conservative_c60471.graph_c0_162683

uuccacagcuuucuugaacuu

>conservative_c76053.graph_c0_421970

uccacauuggacgaaggcuacu

>conservative_c70324.graph_c0_284023

acggaccgacuucaccuuauaagc

>conservative_c65129.graph_c0_207724

agagcucuggcuggaaaucacccu

>conservative_c36330.graph_c0_67678

auuucuugguugcuuuacucc

>conservative_c30149.graph_c0_54380

uccggauucggagcgcccuuu

>conservative_c71243.graph_c2_301799

uggauguagcaaagagaagcu

>conservative_c74676.graph_c0_379582

auugcagguuguggacucuugc

>conservative_c82330.graph_c0_451301

uuguucucuguuuguuuuggc

>conservative_c74654.graph_c1_378954

acacugguguagggugaggacuagg

>conservative_c45777.graph_c0_91882

uugugucauggcugggaggucacu

>conservative_c75806.graph_c0_412828

uccacaauuucaucuuccgg

>conservative_c62782.graph_c0_182690

agaggacgucuuucgaguuagaga

>conservative_c56585.graph_c0_136412

ucgauaaaccucugcauccag

>conservative_c42414.graph_c1_81385

aauaugauuugaucaagugacuau

>conservative_c42772.graph_c0_82491

uggugcuggucuugugcuuggaug

>conservative_c75679.graph_c0_408834

cgauguggugccuucaggaucggcc

>conservative_c67091.graph_c0_232748

aaaaugaucuggaccauucauguc

>conservative_c82856.graph_c0_452321

aagcgggucuggcaucuucggauu

>conservative_c70775.graph_c0_292633

ggagagcucugggcuugggg

>conservative_c34181.graph_c0_63255

uuccacagcuuucuugaacug

>conservative_c57393.graph_c0_143059

aggugugcuggugcgggggc

>conservative_c51783.graph_c0_113379

uucccaaucccucccauuccua

>conservative_c42661.graph_c0_82141

acauauucucuccggcucagcgau

>conservative_c53753.graph_c0_121434

agggguuguggcauccuggcaaga

>conservative_c57698.graph_c0_146291

gcucagugggguugucaguccaca

>conservative_c46204.graph_c0_93133

aucucgaguagagccugcgggugu

>conservative_c56420.graph_c0_135602

auugauguaugugugggacucacu

>conservative_c33546.graph_c0_61987

uuuccuauuccucccauaccua

>conservative_c75719.graph_c1_410211

agacgacgccgucggaagccgguc

>conservative_c53201.graph_c0_119183

uaggagugcuguggauacc

>conservative_c59788.graph_c1_157442

aagucaucuaucucaggcagcagc

>conservative_c65959.graph_c0_217383

uugacagaagagagagagcac

>conservative_c18465.graph_c0_27461

uugaagaacuucggagaaauucgu

>conservative_c69580.graph_c0_270810

uccaaugugagaggauugugu

>conservative_c71561.graph_c0_307742

gaugaggaauuucugauu

>conservative_c63840.graph_c0_193439

agaucucccuugcccuguuccugg

>conservative_c4698.graph_c0_95395

aguguuggaggcgucggcaagaga

>conservative_c81013.graph_c0_448629

agaauuggagggugcuaaaca

>conservative_c72017.graph_c0_317454

accuguguugcaguccgcucgagc

>conservative_c105224.graph_c0_6335

cugaaguguuugggggaacuc

>conservative_c73603.graph_c0_353097

auuugcuguccgguugcuucacgc

>unconservative_c77270.graph_c0_439557

uaaaugcgaucccuugggaau

>unconservative_c38089.graph_c0_71335

uggguuggguuggguugg

>unconservative_c106335.graph_c0_7699

agacuguguuuggaucaaagauuu

>unconservative_c75995.graph_c1_419560

agcguggcuuucuccgcucgagcg

>unconservative_c65489.graph_c0_211886

cgccgcugcuggugucacugg

>unconservative_c60125.graph_c0_159844

cgagguuuggaucgaucugaug

>unconservative_c82109.graph_c0_450906

ugggggucuuguauagucucgagg

>unconservative_c86010.graph_c0_458018

ccgaacucagaaagcugccccu

>unconservative_c48244.graph_c0_99447

auggcacgacggugggucucuucc

>unconservative_c46156.graph_c0_92980

auuugauuuuggcccuugaugugu

>unconservative_c73847.graph_c0_358656

ugggucugugaguuccuggaagucu

>unconservative_c57463.graph_c0_143860

cggugcucuugaauuugauuuu

>unconservative_c20003.graph_c0_31748

uguuacgggccguggauacau

>unconservative_c63894.graph_c0_194052

aaugugugacucuguaguuguugg

>unconservative_c59299.graph_c0_154594

agggauuuggaaagggaaagg

>unconservative_c2583.graph_c0_45322

aauaguccguucggcuuagggugu

>unconservative_c80007.graph_c0_446469

acugcuauagcggcuguaacgggu

>unconservative_c13826.graph_c0_22263

augcgcuggagcagagaaaguugg

>unconservative_c54409.graph_c0_125000

acgacucgucccucguaguauggc

>unconservative_c56241.graph_c0_134623

auccgaaggagugaguaguccauc

>unconservative_c69021.graph_c0_261413

auaagucugauccccgaacuucgg

>unconservative_c73164.graph_c0_343143

ucaaaauugccaaaacgacgucgu

>unconservative_c87882.graph_c0_461178

guaagcuacugaugggcuccaagg

>unconservative_c67492.graph_c0_238175

gauacaucgugugauugggagugu

>unconservative_c69885.graph_c0_276264

accggauccucugucucacuuugc

>unconservative_c56318.graph_c0_135070

agaaagguaauuugggacaagagg

>unconservative_c74857.graph_c0_384696

auuuccgggcauggguuuaug

>unconservative_c41140.graph_c0_77518

uuuccaaguccacccauuccua

>unconservative_c3147.graph_c0_57501

aacucggcucggcucggcu

>unconservative_c69995.graph_c0_278326

cauuucccuuuccaaaucccu

>unconservative_c78304.graph_c0_442247

uaggaucagcagcuuaccaga

>unconservative_c76305.graph_c0_432161

cgggcacgacucgaagauggug

>unconservative_c67492.graph_c0_238174

gauacaucgugugauugggagugu

>unconservative_c71295.graph_c0_302848

aaaauuugggcugggccagaccgg

>unconservative_c67846.graph_c0_242971

aaucugcauccugagguuuagauc

>unconservative_c73377.graph_c2_347857

aaugacgucguuuuggcaaauucg

>unconservative_c63866.graph_c0_193746

acacaauccgaucgcagaaccaga

>unconservative_c57323.graph_c0_142112

gaugcaucaaggaaguugugggug

>unconservative_c38492.graph_c0_72239

gauuaucaaaacgggacucguggu

>unconservative_c70738.graph_c0_291885

aaauagaaaagguaggugaaaugu

>unconservative_c67492.graph_c0_238176

gauacaucgugugauugggagugu

>unconservative_c423.graph_c0_81014

uucccuaauugaugucggacaauu

>unconservative_c118299.graph_c0_20070

agcacagucuguguguguggcugg

>unconservative_c58852.graph_c0_152194

uuauccaaucugaucccugc

>unconservative_c73377.graph_c2_347876

aacgacgucguuuuggcaaauucg

>unconservative_c42080.graph_c0_80376

aaaggugugugaaacuaggugaaa

>unconservative_c70953.graph_c0_296208

uuuccuaaaccacccauuccuc

>unconservative_c71061.graph_c0_298215

auccuucggugcgcacucuguucu

>unconservative_c56155.graph_c0_134173

accccuacugauguauugaccauu

>unconservative_c65997.graph_c0_217806

aggcuguuuccgugacucgaaccc

>unconservative_c31153.graph_c0_56796

uguauaauauguugggccugaggc

>unconservative_c51783.graph_c0_113381

uuuccaagaccacccaugccga

>unconservative_c76307.graph_c3_432267

agauuaaaccauuaagcauagagc

**Precursors**

>conservative_c79576.graph_c0_44229

uuucauuuuugcauggacugaucaugaagauggagaugaugagcaaaaaucucaaggcaccagucuuuuuuagcuauuggagcaucaucaagauucacagaaccuuuuuagagcuucauuggagugguugguugaugguggguauagugaugguggccuuuuguggucccuucucuucgcuuccuucacuccaguggcucuuuugauaugggaaucuugaugaugcugcagcg

>conservative_c103602.graph_c0_4555

agcuuugacgcacaauuguuugacgacgagagagagcacgcucgucagcauucauguaggggacaugauuguauacaagcgugcugucuaucgucgucaugauuugcuugu

>conservative_c66496.graph_c0_224200

augagauagaauuggacacaauccucucacaugagaugaguccuacuaauuguguagguuccaccuaauaugagaugguuguguccgauucuguacaauuauuaggccuua

>conservative_c73855.graph_c0_358837

cucuucgucuucgccuucauuggugaguucgggugagauaaagaaugaggauauugaagcugguucuguugcuuguugucgaauuugccuugaaugugauggcgaggaag

>conservative_c115709.graph_c0_17842

gguggugcuugaguugugauugguuugugcgaguuggaugggucgcuaccugugcagccaacuccacuggugcauuccucuuguuccuuagguaauugccauuagguugu

>conservative_c79710.graph_c0_445788

auaaagacauuuuguucgacgguaguauguugaacaaaagcacguuguucgacuguaauauguugaauaaagacauuuuguucgacgguaguauguugaacaaaagcacuuugu

>conservative_c30045.graph_c0_54121

gacggggacgagacagagcaugagaugggauuuuaauugacacauagaaguuguguuguuauucuucucuugcacugccucuucccuggcucuccacucucuuucucu

>conservative_c71130.graph_c0_299430

cgguaguucucccucggaaccuggcuugugcgcguagcucuugcuuggcgcugcuucuucugcgccaugcaagagcuacgcgcacaagccagguuccgagggagaacuacc

>conservative_c84089.graph_c0_454590

ucguuaugggggauagauauacuaaugcacugaccacucacaaguucggguacuugugaguggucagugcauuuucuguaucuuuuaacuuuucucguuuccaauauaacuuac

>conservative_c71127.graph_c0_299380

caagugcgaucaugcauagaaaaguaccucgaaaaugaaauguguggauaagauuuauucugguugauacauuucauuuucgggguacuuuuuugaggcauuuuuugaagc

>conservative_c64455.graph_c0_200162

cccaauggaaaacacgagagaucuucauuaacgaugaucacucaaggauagagaaugaugugguuauccguuuuguggucuucgcugugagauggagaaagaaacauugggaca

>conservative_c104868.graph_c0_5876

cgggcauguguugacgugaaacuaugagguggagaugcuaucuaaaauauagcacuaccgcuucaaauguuacacaucgucaauauuaauuugaaucgua

>conservative_c75216.graph_c0_395182

cuauggguuucuucuuuauguccucgguacuacccuauuugcgaacagggauaauaccauaggacuuuaucuacugagcaacaugguggagcucgagcgggugagggagga

>conservative_c66896.graph_c1_229677

cagcgugauucagaggcuagaggagaguggccaaccuguugacuucaauaccgugcuugacagguuaaaugggcauucuucugcuggggguucucagagaggauggucuggguaa

>conservative_c62103.graph_c0_176850

agaagguagcggugauguuguugacagaagauagagagcacagaugaugaaaugcacagagagucugcaucucacuccuuugugcucucuauacuucugucaucauccu

>conservative_c51688.graph_c0_113067

gagguuguacgauuuaauuagcugccgacucauucauccaaauacugaguugaucgauguaaauacucaguaaaugagugaaugaugcgggagacaaauugaaucuua

>conservative_c89015.graph_c0_463162

acaacaucguugaugcuucgaaagcuguguggaaagccuuggguaucccucaagauaacugggguggcuuggauacagcuuucgaagcauca

>conservative_c76339.graph_c0_433671

cuucuccaggcauugggguuacaaguagugguggagaagacauguauuccuucaagcuuuccaaagcucggucgcauucgucgucccacucaaacuuggccuuuagggcaugga

>conservative_c99069.graph_c0_477773

gucauaugguugcuuuuacagauccacacacuaacguuaauguguauggccaauguuaguuagugcuggguugugaaaggagccacuggcuguggaaauggua

>conservative_c20625.graph_c0_34424

uagaagcccaaacaaggguucagggugggggguugacggggcucguagccggaguccaccagcaccagcacgugaacguuccccuguucgaaguuuuca

>conservative_c64623.graph_c0_202034

uaucgugcaccacuuucaguugaagcugccagcaugaucuaaacauaccuccuuuguugaggacagauuagaucgugugguagcuucaccuguuguuggugucacgaaa

>conservative_c75679.graph_c0_408830

aacuguacacaucagcuuuuggcgugaucggauccaucuugacccauucgggugccauguacccacgggugccccggauccgugacaugcuaaccaugucuuccuuuuuccuua

>conservative_c69966.graph_c0_277690

cugggacgacaccgucgccgggccucggccggaaagcggccucggaaggcuccguaaacagccuucguucacuuuccggccugucuucggcaaggaaucggacgguggaag

>conservative_c54963.graph_c0_127636

cucucggaaucuugcgguaugggcgaggcgguaagagagaguucucucgaugaucgauugauugauacucuuuccgacuccgcccaugccauuggauuccgauugcuccucc

>conservative_c65848.graph_c0_216092

auuuggaaaggaaaagggaaaaggugugugaaacuaugugaaauguaggcauguuuuuuuuagauuucaccuaccuuuuccauuuuucuuuccaaaucacucuucaaaucccuc

>conservative_c76638.graph_c0_437515

acccgaaagagcgccugcuuggauucccggucuagcugauaaugcaaugggaucacagcuuccaggaccaccacuguuagcucccguuggucagaugggccagggcagucagca

>conservative_c41939.graph_c0_80003

cccuggaguucccucuugcacuucagugaggucucuuggagccucuuuaguuauugguaacuuguaaucacugaaguguuugggggaacucugguugccauuugacacuug

>conservative_c79110.graph_c0_444311

aacuccuuuugcaucuccaccauugauguauguauaggaccauucauguaugugugggguucacucuaucaauuguggcgauuaaaagggguugugggucuccaaaaaauaa

>conservative_c54625.graph_c0_126060

ggccugguggcaaggccuuggguccugggggugugcucugucaaagguucaggaauugaaaccucuuaggugcuaauuauucuuggggaccaucgaacuaggggaaaa

>conservative_c111357.graph_c0_13500

uuuuggguugaaaauauuggccuagcccagcccuaugggcuaaaacaaccuucuuaaacucuagaaaaauagaguuggacugagcaggcuaggcgggucaaugggccgaugucc

>conservative_c71130.graph_c0_299443

cgguaguucucccucggaaccuggcuugugcgcguagcucuugcauggcgcagaagaagcagcgccaagcaagagcuacgcgcacaagccagguuccgagggagaacuacc

>conservative_c45380.graph_c0_90682

uccaugggggcaaacccaucauuggguuucagugggaauuauggcauuaauagcguaaacccgagcuugauggguaguuaugguucccaagcugcuuugcaggguaguua

>conservative_c60471.graph_c0_162683

gcuaaguccugucauguuuuuccacagcuuucuugaacuucaugauguagagauuuggagagagagacaaagagaacaauaccugaaguucaaggaagcugugggaagac

>conservative_c76053.graph_c0_421970

ggauuaagaggaaaccgaaguccacauuggacgaaggcuacuauagggaaagacaucuugucgccaucaggcauuauagagucuuccuccccagcuagucugagauggaccu

>conservative_c70324.graph_c0_284023

ucuucccccguuguugcgggcccaguccgcugggggguuauguuggggcaaucccacaucgccuugggggacggaccgacuucaccuuauaagcguacug

>conservative_c65129.graph_c0_207724

gagagagagagagagaaagagagcucuggcuggaaaucacccucgccgggaaucgccuugccggaaaucgccucuguauuucucucacaagugaacagugcuuagacaaggcu

>conservative_c36330.graph_c0_67678

uuuggugaaauauaguaaagauuucuugguugcuuuacuccuugucuauuauaauauaauauauugugauaagcauggaguaaaucaaccaagaaauucuacucau

>conservative_c30149.graph_c0_54380

accuuagggccgaguaaguauccggauucggagcgcccuuuggggcaugaucccuuagggccgauuaccccuuagggccgaauaacgucuccggauuccgagugcccuucg

>conservative_c71243.graph_c2_301799

aauucuuaugcuucuuuuugcugccuccacuaaauuuuguuucccuuuggugaagggaaagaauauuuaguggauguagcaaagagaagcuuggaaagggcagugccccuu

>conservative_c74676.graph_c0_379582

caggugugguugauggacaaauugcagguuguggacucuugcccagcauacucagcucgcgguuacuucuucaggcaacgcuuagucgaugcccuugacgaggguugguggu

>conservative_c82330.graph_c0_451301

uucaagaaaaauucccuucuuuguucucuguuuguuuuggcagaaaaguauuuuccuuuuccaacagagaaaaacuguuuuuuugccaaaacaaauggaagcucaaaaagu

>conservative_c74654.graph_c1_378954

ccucgcuucgucugcuuugagcccuagcccugcgccucgccucgccucgcgccuaggcgcgcuuuucacaacacugguguagggugaggacuagggcuaugaaagaaguauuuuu

>conservative_c45777.graph_c0_91882

cauucgagggcacaccaucccuugaccuccagcugucaaucaguguccggccaauucggccaaacacugauugugucauggcugggaggucacuugccaccgacuacaccgagc

>conservative_c75806.graph_c0_412828

aucucagaagugauguggcauccacaauuucaucuuccggcaagcauaaacuugaucgagaagaugaugaaguugauggacccacaucaugagaaaauucacauuuaucu

>conservative_c62782.graph_c0_182690

uaucacccuccaacaccucuucgaucucgauucaaaaagcaaccuuuccggcaacaauguuaccggaaagaggacgucuuucgaguuagagaaugaagagguucuagcucuc

>conservative_c56585.graph_c0_136412

uggacgcagcgguucaucgaucuguucccugaacugguuguguucuccaaauuuacagaacaggaaucggucgauaaaccucugcauccagcguucaccacaccucucucu

>conservative_c42414.graph_c1_81385

cgauagucacuugauuaaaucacauuuguaucaacaauuuauuuuaaaaaucauauauaccguugacacaaauaugauuugaucaagugacuauc

>conservative_c42772.graph_c0_82491

cccaucaagugcauauauugcaucucacccagccaauuccuucagauuguguugccuggcaauauaaguuuggugcuggucuugugcuuggaugcgacuuguaccuaggcgugc

>conservative_c75679.graph_c0_408834

ucggauggugaaaacagcgauguggugccuucaggaucggccagagacaaggccgucgauggggaagguggcuaagaugcuggaagggacuguugagaucacugaaccuaaaaaa

>conservative_c67091.graph_c0_232748

acacaacccucacaacuccuaaaaugaucuggaccauucaugucacuaucaauggucaauccaucagugggggucgugagauuccuguugugccuuugggcauagaugacuguu

>conservative_c82856.graph_c0_452321

agccagaccagcccacaaaagggaggggagagggucuggauccgaggugcaacaugcuaccugguuuuggaagcgggucuggcaucuucggauucccaauaagaucaaggucuu

>conservative_c70775.graph_c0_292633

cccucgccgucacuucucucggagagcucugggcuuggggccgcaacaucgaaucccagcucggccgcggccuucacucuccuagagaaucuuggaaugagcccaagaga

>conservative_c34181.graph_c0_63255

aaugacccucuuuguauuuuuccacagcuuucuugaacugcauuagugaaaucuuaauuuuuuuuaugauucaauuguugcgguucagugaagcuguggaaagauauaga

>conservative_c57393.graph_c0_143059

ucuccccgcaccgauucaucuucucuuccuguucacagauccaaacgccugagcaggaagagaaucaggaaggugugcuggugcgggggcagagagggagaggacugaaa

>conservative_c51783.graph_c0_113379

gagaaaagggagguuaggcuucacaaagcuguaggauggagagguuuugggaagaaaauacauaauuuuuuucccaaucccucccauuccuaugacuuuaugugcuuucuc

>conservative_c42661.graph_c0_82141

gccgaguacaaggagacuacauauucucuccggcucagcgauagucgucucaaccuuuucaaagugcaagagggugaugaaauugucugauaaaaugagaagauguagcaccaa

>conservative_c53753.graph_c0_121434

cucucaugagcaaaaacccaagggguuguggcauccuggcaagaucuuggggcauaagccugagaucccagguuccacucucuccggggcuaaccauccgggcaauagcauaua

>conservative_c57698.graph_c0_146291

cuggugaggcaagcacaagugcucagugggguugucaguccacagcugguugguuguggaaaguacaacccuuuuuggucuuauggcguguuuggauuauugauuucguuuugc

>conservative_c46204.graph_c0_93133

agaucucgaguagagccugcggguguggcccuaaaccuaagacuuggagggagcacacucccaggcuccaaguugcacccacuaagccaaccccuugggauuauuugaggaugu

>conservative_c56420.graph_c0_135602

uuuggauuuagauuucuaccauugauguaugugugggacucacucauguauacguggagcccacaucaucgauggugaagaucuaaaagggguuacgggggucuucaaaaaaca

>conservative_c33546.graph_c0_61987

ccaacgggagauuuggguugggagugguaggaaaagccucugagaaaauggaaaauuuggaggaauggcuuuuccuauuccucccauaccuaggguuuccgaucgcuccuc

>conservative_c75719.graph_c1_410211

ucgucagcggcgaaggcggagacgacgccgucggaagccggucugacgguggcgacaacggaagcgucgauuccgcugagucgacucggcagcaucgccgauuugagcugaga

>conservative_c53201.graph_c0_119183

uuccuccaacugcugccacugucagcuccucucuccugcucgguaucugcagucuccccgauacaauguuuaggagugcuguggauaccgcguuggcagauucugagug

>conservative_c59788.graph_c1_157442

cagucuggcaugaaugcuucaagucaucuaucucaggcagcagcuacuguugauuucuaucuuaacaucugucaugcugcuaaggcugcggacgagaagcuaguuagggaccag

>conservative_c65959.graph_c0_217383

gauggguuucgagaaaaauguugacagaagagagagagcacaacccgccaucagcuaaagaaaaucuuuauguguugugggagugugcucuuucuucuucugucaucaucu

>conservative_c18465.graph_c0_27461

ugaugaauuuuaagggaaacuauacuuuucucugaaguucuuaauaaucaucuaucuauguauguaauuguugaagaacuucggagaaauucguguaauuguugagauuuauuu

>conservative_c69580.graph_c0_270810

gaaaaagucaagaaauacaggaaucgaucacggaaauggacacaacacucucacacgagguggauuccacuccaaugugagaggauuguguucgguuuugugauugguuuc

>conservative_c71561.graph_c0_307742

guguuggaauuccggcgguggugaugaggaauuucugauuuaauuuauggaggugguuaccucucauggaauacgacgccggaauuccgaugucccaagccgcugucaccgacgc

>conservative_c63840.graph_c0_193439

ccgacagagcggaugcuuccagagcuucuccgauaguuaucgcaucacgguugauagccggugcugccggagaucucccuugcccuguuccuggcuguguguauugucccacua

>conservative_c4698.graph_c0_95395

ucaaugaucaauacauuaguggaggucauaggguguccucuuuugugggggucucaaacauuuucaccgaaguguuggaggcgucggcaagagagcgaaag

>conservative_c81013.graph_c0_448629

aagaaucguaugugagaauuggagggugcuaaacaaauuguugcuaaaaccauuugcacacauuguuuaguuuucuccaauaucucauguccaacuucucaacug

>conservative_c72017.graph_c0_317454

ucaagcucccaguauuuuugaccuguguugcaguccgcucgagcggggaaaccugagcucgagcggaggccuuguuucacugaaauuucuggaauuguuuucuaaguguauuuu

>conservative_c105224.graph_c0_6335

ugugcccuagaguuuccccacaacacuucauuggggacuuucuuuuuucuuuauuuuuaaacuuuccuuacugaaguguuugggggaacuccugggaucauuugauauuuc

>conservative_c73603.graph_c0_353097

ccgugggcauauuuuggucaacugguguugcauguguuguggagcugcggagucaguggaucauuugcuuauuugcuguccgguugcuucacgccuuuggaugucuauagucgc

>unconservative_c77270.graph_c0_439557

gcagguggagaacuccaaagggaucgcauugauccaacguucgauugaucgcaugguuuuuuugguuggauaaaugcgaucccuugggaauuuuccaucuauagcucu

>unconservative_c38089.graph_c0_71335

cgcagucaccuuuuggcuucguuugggaagauggguuggguuggguuggguuuggguucaauuggguacgauucggagguucucaugaguuggaggggacgagaguua

>unconservative_c106335.graph_c0_7699

cuguaauucuugauguuuucagacuguguuuggaucaaagauuugaguucacaucuuucaaaucuuugauccuuaauaaacacagucucaguguguugagcuccacaaauccc

>unconservative_c75995.graph_c1_419560

gaaaacagaggucucgcucgagcguggcuuucuccgcucgagcgagcuuacuaaaacagagggucgcucgagcguagacuuccucgcucgagcagucuguaaaaaugcaaaaag

>unconservative_c65489.graph_c0_211886

cugcucacucgcaaagccaccgccgcugcuggugucacugggucugcgucgagucgaucauugucgagcucgacuagauccaauggcggcagcggugguggcggugcaacg

>unconservative_c60125.graph_c0_159844

ugaagucagaaacgaaguagacgaagaggaagaagacgaucgcgaucaagaucgcgaucgaggauuagaucgagguuuggaucgaucugauggugagagagacgaagaagau

>unconservative_c82109.graph_c0_450906

uacaccuagaccuagauuucugggggucuuguauagucucgaggaauguggaucuagauguguuuucuuuuuuauauuucuuggauuaucauucuguauaaauuaggagguggu

>unconservative_c86010.graph_c0_458018

aacuagccacuuaugagcacacgaccgagcuaaaacuucaggggccaagccagcuuucuguuuucccaagccgaacucagaaagcugccccuugacguucagccggaauauc

>unconservative_c48244.graph_c0_99447

agaagagggaagagagaaagaaagaaagaagaggcgauggcggcgugacaaaagcgauggugcuacggugauggcacgacggugggucucuuccucucucuucucucaacagau

>unconservative_c46156.graph_c0_92980

uugagauuucaugacccccaauuugauuuuggcccuugauguguauguagggucuacucauguauauguaggccccauauuaugagugguugagaugagagugaggauuguugg

>unconservative_c73847.graph_c0_358656

uuugauuucugagauguugauugggucugugaguuccuggaagucuagucggaagagcacuugaucaccgcuauuucugauuggaacucuagcaacaucaugggacaaucauua

>unconservative_c57463.graph_c0_143860

aaaggcgguuuagcccgcuacggugcucuugaauuugauuuugcaguugccccauuucggcagcaauugcagaaagccuguuugcagcauccauagccauacgugcuaguac

>unconservative_c20003.graph_c0_31748

acccuaaaaaggcgaaccucuuggaucaucacuccaucaagcacauucucgacgaguccgucacugagauuguuacgggccguggauacauugaagaugugaggaugagua

>unconservative_c63894.graph_c0_194052

gcuccccaguauugccucaacaacccaagagucacauauuuauauggaauucauugacagguuucacacaaaugugugacucuguaguuguuggggcaauaccuuaaaaacacg

>unconservative_c59299.graph_c0_154594

gauuuggggagauuuggaaaagggauuuggaaagggaaaggaaauaugaauaaaauauaaguuuuuuuuuuuuuucuccauuucccucccauuuuccuuucucuuuccaaa

>unconservative_c2583.graph_c0_45322

gguagucccuucggacuaaaaggccuugggcaagcggauuaacagucugagcaaggagaugguauuagaaaauaguccguucggcuuaggguguuaccagccggucaaccggcc

>unconservative_c80007.graph_c0_446469

uacacauguaaauuugucacacugcuauagcggcuguaacggguggcaauaccuaucacagcgguuguaacggguagcaguaacuaucacagccauuauguaacaaccguuacg

>unconservative_c13826.graph_c0_22263

uucguuucagcucuuuauucucugccuucaaugcgcaaaucucugcucacgcaucuuuguacaugcgcuggagcagagaaaguugggcauuuaacgaauguacc

>unconservative_c54409.graph_c0_125000

acacguucaauguauaggcacgaacuaucucaaguaguacgagucguucgcucuaaauuaggguagcaacacgacucgucccucguaguauggcuggucucauaccuugaacau

>unconservative_c56241.graph_c0_134623

auuauuagugaacauagguuguguuugauugggggauuaagggggggacuauucacuauucggauuguuuauccgaaggagugaguaguccaucauuuguuuuuuuuuuaguca

>unconservative_c69021.graph_c0_261413

auauaggcguguacgaagggucggggagucgaggcaaugauuaaagggggaguaauuaaacuuccgcgugauaagucugauccccgaacuucgguucagagacuuugaaaauug

>unconservative_c73164.graph_c0_343143

uuuguggcucaaauggcucaaaauugccaaaacgacgucguuuugggcguaaaacgacgcaaaacgacgucguuuuaacaguuuugagccauuugagccacacuuuuuaug

>unconservative_c87882.graph_c0_461178

uguauuuuguuuguacugccguaagcuacugaugggcuccaagguugugggccauggaugagcaagacaucaaacaaucuuggagcccuucaacaguuuacgguaguauuaaaa

>unconservative_c67492.graph_c0_238175

aucuucaaguauuuugaugagauacaucgugugauugggaguguauugaaaauuuugcuaauccucuguccccaugcgcucacagucgcaugauugugucucaaucuucaagua

>unconservative_c69885.graph_c0_276264

ccaaugauaaaaaggauguuuggaauagggagacaugagagugggacagagaauccauagccaauggacaaccggauccucugucucacuuugcauaaccucugucccaaguuu

>unconservative_c56318.graph_c0_135070

uuuuuggauaucuccucuacucuugucucaagguuccuuucuaucauauugaccuauuauauuguacgguagaaagguaauuugggacaagaggagagaggaucgggacaaaag

>unconservative_c74857.graph_c0_384696

augggugggauagggaagacaacccuugcucggaaagucuacaaccacccaguuguugcugagaauuuugauuuccgggcauggguuuaugucucucaaggcuacagagug

>unconservative_c41140.graph_c0_77518

ugccgaaaaucuuuggggauugguggcuuggaaagcuucguucuuuucucuuuugaaugaauuuaauggcuuuccaaguccacccauuccuauuguuuucggucuucucucu

>unconservative_c3147.graph_c0_57501

cgaaccgagccaagcuugaguuugauucauuuucgagcucgauaaacaaaggagaugagcuugagcucugcaaaacucggcucggcucggcucaacuuguuggcagcccuaau

>unconservative_c69995.graph_c0_278326

guagggauuugaaaaagaaaaggaaagggaagacaagaaggaaugaagaauauaagcuaauuguuuucuccauuucccuuuccaaaucccuauccaaauuugggauccaaa

>unconservative_c78304.graph_c0_442247

aaagagagaaugugggcaagaacuaaaucugguaaguugcugauccuaucuucugagggaauggcgaagauaggaucagcagcuuaccagauucaguucuaaccagaa

>unconservative_c76305.graph_c0_432161

aagaggggaugucugcagcacgggcacgacucgaagauggugugccaguaggccucagugguggagcucguacucccgucaagggggacaucagauauaugcggcgauaggu

>unconservative_c67492.graph_c0_238174

uuugcuaauccucuguccccaugcgcucacagucgcaugauugugucucaaucuucaaguauuuugaugagauacaucgugugauugggaguguauugggauauugggacuacg

>unconservative_c71295.graph_c0_302848

uuuguuuaauaaugauuggugguguaaauuuggcccgguccguucauguugggccguguaaguucuagucaaaauuugggcugggccagaccgggugacaggccaaaaaauuaa

>unconservative_c67846.graph_c0_242971

accaagucaagaucagguaaucugcauccugagguuuagaucaauauauaugucguauguaucuagcucucaggguacagauuaccucuuccuuacuggcaacgugguagag

>unconservative_c73377.graph_c2_347857

uuguagcucaaauggcucgaauuugccaaaacgacgucguuuuguuauaaaacgaccguuuuguacccaaaaugacgucguuuuggcaaauucgagucauuugauccaaagaaa

>unconservative_c63866.graph_c0_193746

aguagaaaaauucuacgugcacacaauccgaucgcagaaccagacacaaccauaacaacgagaaaguauugugcaguugugugaucggauucuaugcacguagaagaacugau

>unconservative_c57323.graph_c0_142112

auuuucugaccuucauugaugaugcaucaaggaaguuguggguguauuuccugaagacaaaggaugagguauuucagcacuuucaggaguuccaugcaaugguggaaagagaga

>unconservative_c38492.graph_c0_72239

acacauuaaacgauucugauuaucaaaacgggacucguggugggccucuacgggccgaacaucaccguccuguguaaacaacaucguaggcaaguuuuucuuauaucucuu

>unconservative_c70738.graph_c0_291885

gauuuggaaaagaaaauagaaaagguaggugaaaugugaauauaauauaucuacauuuuaucuaguuucguacacuauuuucuuuuuuucuuuccaaaucccuuuucaaau

>unconservative_c67492.graph_c0_238176

uuugcuaauccucuguccccaugcgcucacagucgcaugauugugucucaaucuucaaguauuuugaugagauacaucgugugauugggaguguauugaaaauuuugcuaaucc

>unconservative_c423.graph_c0_81014

ggcauggggaggaauauuucuucccuaauugaugucggacaauuugccaagguaagugccucgccaauuguuaaguugucccacauugguuaggggagaaaaacuuacuccccc

>unconservative_c118299.graph_c0_20070

aaucaaauuaauaauuauuugauggaaaauaaugauaugauguaucacuagcacuggcagugcacagcagcacagucuguguguguggcuggccuuauuuauuuauuuuuuuaa

>unconservative_c58852.graph_c0_152194

cucgcauuaaaacaugcgagauuuggguguuccgccccaagccugguuacgacaaauugggcacucacauuuuauccaaucugaucccugcgcuugaaggaauuaccaaa

>unconservative_c73377.graph_c2_347876

cuuuggaucaaaugacucgaauuugccaaaacgacgucauuuuggguacaaaacggucguuuuauaacaaaacgacgucguuuuggcaaauucgagccauuugagcuacaaaaa

>unconservative_c42080.graph_c0_80376

gauuuggaaaggaaaaugggaaaggugugugaaacuaggugaaagguauauuuuauucacuuuucaccuucuuuuuuaauuuuucuuuccaaauucuuucccaaauucuc

>unconservative_c70953.graph_c0_296208

uaacaagaaauugugggaaugggucgauugggaaggaauuccuuugguggguauugccaaacgaaauccuuuccuaaaccacccauuccucugauuucuugauguuuuucu

>unconservative_c71061.graph_c0_298215

uucagacuccucuucacuaucagauggacaacgaaggggacgucuaggagcauucugaacagguugaccucgggcaaauccuucggugcgcacucuguucugguuaacaggu

>unconservative_c56155.graph_c0_134173

ccacaaaagaggauucuacgaccccuacugauguauugaccauugaugugguugaauuuuuuuuucuaucucugacaaugacauaaaugguccagaucauuuuggggguugugu

>unconservative_c65997.graph_c0_217806

gcacgacgcucccgcuuaagcggggucuggggaaggguugauauaugcagccuuaccgccgcaaacggagaggcuguuuccgugacucgaacccgugaccacuaggucacaaag

>unconservative_c31153.graph_c0_56796

uguuggcccugagaccauucuguauaauauguugggccugaggccguuucguaucauaugugggcccgaggccguucuguauuacauguaggccugaggccguucuaaaauaug

>unconservative_c51783.graph_c0_113381

aagggaggcagaucugcacgaaguuauuggcauggugaucuugggaaagaaagaguuguguuuuagaucuuuuccaagaccacccaugccgaugauuucuugcagaucc

>unconservative_c76307.graph_c3_432267

auacuuuugguaaugaaucucuuaaguauaaaacguuuuaugccguugguaaugaaucuccaaagagauuaaaccauuaagcauagagcguuuuauacuuuugauaauaa

**Supplementary Dataset 3.** The validated miRNA targets with alignment score over 5 and annotation information of targets.

Validated miRNA-targets in degradome sequencing:

| **miRNAs** | **Validated Targets** | **Aligned range** | **Slice site** |
| --- | --- | --- | --- |
| **CJPconservative_c105647.graph_c0_611** | c105647.graph_c0 | 110-129 Slice Site | 120 |
| **CJPconservative_c105647.graph_c0_611** | c29785.graph_c0 | 588-607 Slice Site | 598 |
| **CJPconservative_c105647.graph_c0_611** | c69172.graph_c1 | 1933-1951 Slice Site | 1943 |
| **CJPconservative_c105647.graph_c0_611** | c76058.graph_c0 | 3113-3132 Slice Site | 3123 |
| **CJPconservative_c23362.graph_c0_3894** | c66032.graph_c1 | 5689-5710 Slice Site | 5701 |
| **CJPconservative_c23362.graph_c0_3894** | c71579.graph_c0 | 1373-1392 Slice Site | 1383 |
| **CJPconservative_c30045.graph_c0_5397** | c30082.graph_c0 | 121-141 Slice Site | 132 |
| **CJPconservative_c30045.graph_c0_5397** | c52955.graph_c0 | 131-153 Slice Site | 144 |
| **CJPconservative_c30045.graph_c0_5397** | c71575.graph_c0 | 522-541 Slice Site | 532 |
| **CJPconservative_c30045.graph_c0_5397** | c74215.graph_c0 | 1456-1476 Slice Site | 1467 |
| **CJPconservative_c30045.graph_c0_5397** | c75240.graph_c0 | 1131-1151 Slice Site | 1142 |
| **CJPconservative_c32855.graph_c0_6076** | c56142.graph_c0 | 563-583 Slice Site | 575 |
| **CJPconservative_c32855.graph_c0_6076** | c73928.graph_c0 | 1165-1185 Slice Site | 1176 |
| **CJPconservative_c34181.graph_c0_6365** | c60557.graph_c0 | 553-572 Slice Site | 562 |
| **CJPconservative_c34181.graph_c0_6365** | c61656.graph_c0 | 487-508 Slice Site | 498 |
| **CJPconservative_c34181.graph_c0_6365** | c67047.graph_c0 | 610-629 Slice Site | 621 |
| **CJPconservative_c34181.graph_c0_6365** | c67408.graph_c0 | 182-202 Slice Site | 193 |
| **CJPconservative_c34181.graph_c0_6365** | c69226.graph_c0 | 973-994 Slice Site | 984 |
| **CJPconservative_c34181.graph_c0_6365** | c69760.graph_c1 | 1161-1181 Slice Site | 1172 |
| **CJPconservative_c34181.graph_c0_6365** | c70118.graph_c0 | 1945-1964 Slice Site | 1955 |
| **CJPconservative_c34181.graph_c0_6365** | c75132.graph_c0 | 2206-2227 Slice Site | 2217 |
| **CJPconservative_c34181.graph_c0_6365** | c75779.graph_c2 | 672-693 Slice Site | 683 |
| **CJPconservative_c34181.graph_c0_6365** | c76129.graph_c0 | 666-684 Slice Site | 675 |
| **CJPconservative_c34181.graph_c0_6365** | c78379.graph_c0 | 175-195 Slice Site | 186 |
| **CJPconservative_c34181.graph_c0_6365** | c80882.graph_c0 | 278-298 Slice Site | 289 |
| **CJPconservative_c35784.graph_c0_6703** | c64707.graph_c0 | 527-550 Slice Site | 541 |
| **CJPconservative_c36330.graph_c0_6908** | c57769.graph_c0 | 810-831 Slice Site | 822 |
| **CJPconservative_c36330.graph_c0_6908** | c64598.graph_c0 | 973-992 Slice Site | 983 |
| **CJPconservative_c36330.graph_c0_6908** | c69798.graph_c0 | 1355-1375 Slice Site | 1366 |
| **CJPconservative_c36330.graph_c0_6908** | c70056.graph_c0 | 296-316 Slice Site | 307 |
| **CJPconservative_c36330.graph_c0_6908** | c72724.graph_c0 | 1878-1897 Slice Site | 1888 |
| **CJPconservative_c36330.graph_c0_6908** | c75782.graph_c1 | 1023-1043 Slice Site | 1034 |
| **CJPconservative_c36330.graph_c0_6908** | c76364.graph_c0 | 3165-3187 Slice Site | 3178 |
| **CJPconservative_c46859.graph_c0_9784** | c19937.graph_c0 | 147-168 Slice Site | 159 |
| **CJPconservative_c46859.graph_c0_9784** | c35959.graph_c0 | 68-88 Slice Site | 79 |
| **CJPconservative_c46859.graph_c0_9784** | c46859.graph_c0 | 230-250 Slice Site | 241 |
| **CJPconservative_c46859.graph_c0_9784** | c59307.graph_c0 | 177-197 Slice Site | 188 |
| **CJPconservative_c46859.graph_c0_9784** | c63325.graph_c0 | 153-173 Slice Site | 165 |
| **CJPconservative_c46859.graph_c0_9784** | c70152.graph_c0 | 302-323 Slice Site | 314 |
| **CJPconservative_c46859.graph_c0_9784** | c71093.graph_c0 | 891-911 Slice Site | 902 |
| **CJPconservative_c46859.graph_c0_9784** | c71254.graph_c0 | 1054-1075 Slice Site | 1065 |
| **CJPconservative_c46859.graph_c0_9784** | c74175.graph_c2 | 762-782 Slice Site | 773 |
| **CJPconservative_c46859.graph_c0_9784** | c75798.graph_c0 | 566-587 Slice Site | 578 |
| **CJPconservative_c51783.graph_c0_11743** | c22570.graph_c0 | 65-85 Slice Site | 76 |
| **CJPconservative_c51783.graph_c0_11743** | c55059.graph_c0 | 656-678 Slice Site | 669 |
| **CJPconservative_c51783.graph_c0_11743** | c59123.graph_c0 | 907-925 Slice Site | 916 |
| **CJPconservative_c51783.graph_c0_11743** | c65994.graph_c0 | 1591-1612 Slice Site | 1603 |
| **CJPconservative_c51783.graph_c0_11743** | c66291.graph_c0 | 135-155 Slice Site | 145 |
| **CJPconservative_c51783.graph_c0_11743** | c73714.graph_c0 | 722-742 Slice Site | 733 |
| **CJPconservative_c51783.graph_c0_11743** | c75061.graph_c0 | 1632-1651 Slice Site | 1643 |
| **CJPconservative_c55164.graph_c0_13241** | c73502.graph_c0 | 1942-1956 Slice Site | 1947 |
| **CJPconservative_c58697.graph_c0_15251** | c31774.graph_c0 | 44-62 Slice Site | 53 |
| **CJPconservative_c58697.graph_c0_15251** | c67579.graph_c0 | 969-987 Slice Site | 978 |
| **CJPconservative_c58697.graph_c0_15251** | c70409.graph_c0 | 425-443 Slice Site | 434 |
| **CJPconservative_c58697.graph_c0_15251** | c75247.graph_c0 | 80-98 Slice Site | 89 |
| **CJPconservative_c59299.graph_c0_15578** | c22768.graph_c0 | 193-216 Slice Site | 207 |
| **CJPconservative_c59299.graph_c0_15578** | c70489.graph_c0 | 932-957 Slice Site | 948 |
| **CJPconservative_c59299.graph_c0_15578** | c73503.graph_c0 | 2140-2163 Slice Site | 2154 |
| **CJPconservative_c59299.graph_c0_15578** | c73796.graph_c0 | 1101-1122 Slice Site | 1113 |
| **CJPconservative_c59299.graph_c0_15578** | c75522.graph_c2 | 1032-1055 Slice Site | 1046 |
| **CJPconservative_c59299.graph_c0_15578** | c75548.graph_c1 | 31-55 Slice Site | 45 |
| **CJPconservative_c61050.graph_c0_16614** | c61050.graph_c0 | 48-67 Slice Site | 58 |
| **CJPconservative_c65959.graph_c0_20343** | c19964.graph_c0 | 1671-1692 Slice Site | 1683 |
| **CJPconservative_c65959.graph_c0_20343** | c42431.graph_c1 | 286-307 Slice Site | 297 |
| **CJPconservative_c65959.graph_c0_20343** | c62280.graph_c0 | 77-96 Slice Site | 88 |
| **CJPconservative_c65959.graph_c0_20343** | c69827.graph_c0 | 1656-1676 Slice Site | 1667 |
| **CJPconservative_c65959.graph_c0_20343** | c73704.graph_c0 | 35-55 Slice Site | 46 |
| **CJPconservative_c66622.graph_c1_20928** | c74789.graph_c0 | 246-266 Slice Site | 257 |
| **CJPconservative_c68751.graph_c0_23175** | c29785.graph_c0 | 588-608 Slice Site | 599 |
| **CJPconservative_c68751.graph_c0_23175** | c39287.graph_c0 | 291-311 Slice Site | 302 |
| **CJPconservative_c68751.graph_c0_23175** | c47159.graph_c0 | 269-289 Slice Site | 280 |
| **CJPconservative_c68751.graph_c0_23175** | c60342.graph_c3 | 391-410 Slice Site | 401 |
| **CJPconservative_c68751.graph_c0_23175** | c63597.graph_c0 | 826-846 Slice Site | 837 |
| **CJPconservative_c68751.graph_c0_23175** | c73632.graph_c2 | 1155-1175 Slice Site | 1166 |
| **CJPconservative_c68751.graph_c0_23175** | c74150.graph_c0 | 2190-2210 Slice Site | 2201 |
| **CJPconservative_c68751.graph_c0_23175** | c74577.graph_c1 | 1974-1993 Slice Site | 1985 |
| **CJPconservative_c69076.graph_c1_23496** | c69433.graph_c0 | 705-728 Slice Site | 719 |
| **CJPconservative_c69076.graph_c1_23496** | c74732.graph_c1 | 513-536 Slice Site | 527 |
| **CJPconservative_c71073.graph_c0_25835** | c71908.graph_c0 | 248-268 Slice Site | 259 |
| **CJPconservative_c74438.graph_c0_30822** | c42232.graph_c0 | 55-72 Slice Site | 63 |
| **CJPconservative_c74438.graph_c0_30822** | c57304.graph_c0 | 1824-1841 Slice Site | 1832 |
| **CJPconservative_c74438.graph_c0_30822** | c60424.graph_c0 | 416-436 Slice Site | 425 |
| **CJPconservative_c74438.graph_c0_30822** | c63759.graph_c0 | 588-605 Slice Site | 596 |
| **CJPconservative_c74438.graph_c0_30822** | c66561.graph_c0 | 1739-1756 Slice Site | 1747 |
| **CJPconservative_c74438.graph_c0_30822** | c66658.graph_c0 | 303-320 Slice Site | 311 |
| **CJPconservative_c74438.graph_c0_30822** | c67894.graph_c0 | 3595-3613 Slice Site | 3603 |
| **CJPconservative_c74438.graph_c0_30822** | c68352.graph_c0 | 776-793 Slice Site | 784 |
| **CJPconservative_c74438.graph_c0_30822** | c69278.graph_c0 | 749-766 Slice Site | 757 |
| **CJPconservative_c74438.graph_c0_30822** | c69679.graph_c2 | 538-555 Slice Site | 546 |
| **CJPconservative_c74438.graph_c0_30822** | c71850.graph_c0 | 629-646 Slice Site | 637 |
| **CJPconservative_c74438.graph_c0_30822** | c71937.graph_c0 | 1653-1670 Slice Site | 1661 |
| **CJPconservative_c74438.graph_c0_30822** | c73092.graph_c0 | 940-956 Slice Site | 947 |
| **CJPconservative_c74438.graph_c0_30822** | c75261.graph_c0 | 3764-3783 Slice Site | 3773 |
| **CJPconservative_c74438.graph_c0_30822** | c75485.graph_c0 | 1603-1620 Slice Site | 1611 |
| **CJPconservative_c74438.graph_c0_30822** | c75543.graph_c3 | 1135-1153 Slice Site | 1143 |
| **CJPconservative_c74438.graph_c0_30822** | c76314.graph_c0 | 849-866 Slice Site | 857 |
| **CJPconservative_c74438.graph_c0_30822** | c77194.graph_c0 | 36-53 Slice Site | 44 |
| **CJPconservative_c74438.graph_c0_30822** | c78425.graph_c0 | 305-322 Slice Site | 313 |
| ***CJPconservative_c76997.graph_c0_36486*** | ***c29365.graph_c0*** | 475-495 Slice Site | 486 |
| ***CJPconservative_c76997.graph_c0_36486*** | ***c58482.graph_c0*** | 287-307 Slice Site | 298 |
| ***CJPconservative_c76997.graph_c0_36486*** | ***c71241.graph_c0*** | 914-933 Slice Site | 925 |
| **CJPunconservative_c41140.graph_c0_7974** | c30106.graph_c0 | 319-340 Slice Site | 331 |
| **CJPunconservative_c41140.graph_c0_7974** | c41140.graph_c0 | 580-601 Slice Site | 592 |
| **CJPunconservative_c41140.graph_c0_7974** | c54366.graph_c0 | 302-323 Slice Site | 314 |
| **CJPunconservative_c41140.graph_c0_7974** | c56631.graph_c0 | 239-260 Slice Site | 251 |
| **CJPunconservative_c41140.graph_c0_7974** | c57315.graph_c0 | 174-192 Slice Site | 183 |
| **CJPunconservative_c41140.graph_c0_7974** | c64223.graph_c0 | 963-985 Slice Site | 976 |
| **CJPunconservative_c41140.graph_c0_7974** | c67144.graph_c1 | 1289-1310 Slice Site | 1301 |
| **CJPunconservative_c41140.graph_c0_7974** | c74758.graph_c0 | 1611-1631 Slice Site | 1622 |
| **CJPunconservative_c41140.graph_c0_7974** | c74771.graph_c0 | 963-983 Slice Site | 974 |
| **CJPunconservative_c41140.graph_c0_7974** | c75905.graph_c0 | 603-625 Slice Site | 616 |
| **CJPunconservative_c70953.graph_c0_25662** | c25572.graph_c0 | 1161-1182 Slice Site | 1173 |
| **CJPunconservative_c70953.graph_c0_25662** | c27184.graph_c0 | 156-176 Slice Site | 167 |
| **CJPunconservative_c71775.graph_c1_26775** | c32623.graph_c0 | 202-225 Slice Site | 216 |
| **CJPunconservative_c71775.graph_c1_26775** | c65848.graph_c0 | 314-337 Slice Site | 328 |
| **CJPunconservative_c71775.graph_c1_26775** | c73161.graph_c0 | 2316-2339 Slice Site | 2330 |
| **CJPunconservative_c73011.graph_c0_28646** | c28899.graph_c0 | 268-284 Slice Site | 276 |
| **CJPunconservative_c73011.graph_c0_28646** | c45188.graph_c0 | 211-228 Slice Site | 218 |
| **CJPunconservative_c73011.graph_c0_28646** | c50172.graph_c0 | 108-123 Slice Site | 115 |
| **CJPunconservative_c73011.graph_c0_28646** | c56077.graph_c0 | 469-486 Slice Site | 477 |
| **CJPunconservative_c73011.graph_c0_28646** | c56701.graph_c0 | 356-372 Slice Site | 364 |
| **CJPunconservative_c73011.graph_c0_28646** | c59665.graph_c0 | 1051-1068 Slice Site | 1059 |
| **CJPunconservative_c73011.graph_c0_28646** | c61907.graph_c0 | 649-665 Slice Site | 657 |
| **CJPunconservative_c73011.graph_c0_28646** | c64063.graph_c0 | 190-205 Slice Site | 198 |
| **CJPunconservative_c73011.graph_c0_28646** | c64238.graph_c0 | 54-71 Slice Site | 62 |
| **CJPunconservative_c73011.graph_c0_28646** | c65013.graph_c0 | 946-963 Slice Site | 954 |
| **CJPunconservative_c73011.graph_c0_28646** | c66032.graph_c1 | 3538-3556 Slice Site | 3547 |
| **CJPunconservative_c73011.graph_c0_28646** | c66985.graph_c0 | 238-255 Slice Site | 246 |
| **CJPunconservative_c73011.graph_c0_28646** | c68791.graph_c0 | 1069-1085 Slice Site | 1077 |
| **CJPunconservative_c73011.graph_c0_28646** | c69066.graph_c0 | 916-933 Slice Site | 924 |
| **CJPunconservative_c73011.graph_c0_28646** | c69113.graph_c0 | 1016-1033 Slice Site | 1024 |
| **CJPunconservative_c73011.graph_c0_28646** | c69664.graph_c0 | 315-331 Slice Site | 323 |
| **CJPunconservative_c73011.graph_c0_28646** | c70652.graph_c0 | 2894-2910 Slice Site | 2901 |
| **CJPunconservative_c73011.graph_c0_28646** | c71317.graph_c0 | 1142-1157 Slice Site | 1150 |
| **CJPunconservative_c73011.graph_c0_28646** | c71819.graph_c0 | 1876-1894 Slice Site | 1884 |
| **CJPunconservative_c73011.graph_c0_28646** | c71919.graph_c0 | 2977-2994 Slice Site | 2985 |
| **CJPunconservative_c73011.graph_c0_28646** | c72458.graph_c0 | 1008-1025 Slice Site | 1017 |
| **CJPunconservative_c73011.graph_c0_28646** | c72700.graph_c0 | 1641-1658 Slice Site | 1648 |
| **CJPunconservative_c73011.graph_c0_28646** | c72874.graph_c3 | 564-580 Slice Site | 572 |
| **CJPunconservative_c73011.graph_c0_28646** | c73251.graph_c0 | 1006-1024 Slice Site | 1015 |
| **CJPunconservative_c73011.graph_c0_28646** | c73796.graph_c0 | 1032-1049 Slice Site | 1040 |
| **CJPunconservative_c73011.graph_c0_28646** | c74969.graph_c2 | 1278-1295 Slice Site | 1286 |
| **CJPunconservative_c73011.graph_c0_28646** | c75161.graph_c0 | 1778-1795 Slice Site | 1786 |
| **CPconservative_c45380.graph_c0_90682** | c74692.graph_c1 | 1335-1355 Slice Site | 1346 |
| **CPconservative_c51688.graph_c0_113067** | c62600.graph_c0 | 975-993 Slice Site | 985 |
| **CPconservative_c54625.graph_c0_126060** | c64202.graph_c0 | 1096-1116 Slice Site | 1107 |
| **CPconservative_c57393.graph_c0_143059** | c70437.graph_c0 | 102-120 Slice Site | 112 |
| **CPconservative_c66496.graph_c0_224200** | c40974.graph_c0 | 97-116 Slice Site | 107 |
| **CPconservative_c66496.graph_c0_224200** | c45536.graph_c0 | 54-74 Slice Site | 65 |
| **CPconservative_c66496.graph_c0_224200** | c52830.graph_c0 | 218-238 Slice Site | 229 |
| **CPconservative_c66496.graph_c0_224200** | c66496.graph_c0 | 607-627 Slice Site | 618 |
| **CPconservative_c69580.graph_c0_270810** | c59137.graph_c1 | 20-39 Slice Site | 30 |
| **CPconservative_c69580.graph_c0_270810** | c70720.graph_c0 | 202-222 Slice Site | 213 |
| **CPconservative_c69966.graph_c0_277690** | c61514.graph_c0 | 435-454 Slice Site | 445 |
| **CPconservative_c69966.graph_c0_277690** | c66629.graph_c0 | 574-593 Slice Site | 584 |
| **CPconservative_c69966.graph_c0_277690** | c69062.graph_c0 | 1432-1451 Slice Site | 1442 |
| **CPconservative_c69966.graph_c0_277690** | c70721.graph_c0 | 708-727 Slice Site | 718 |
| **CPconservative_c69966.graph_c0_277690** | c74630.graph_c0 | 4104-4124 Slice Site | 4115 |
| **CPconservative_c69966.graph_c0_277690** | c75232.graph_c0 | 1917-1937 Slice Site | 1928 |
| **CPconservative_c73855.graph_c0_358837** | c44069.graph_c1 | 106-125 Slice Site | 116 |
| **CPconservative_c73855.graph_c0_358837** | c45987.graph_c0 | 41-59 Slice Site | 50 |
| **CPconservative_c73855.graph_c0_358837** | c69859.graph_c0 | 1074-1093 Slice Site | 1084 |
| **CPconservative_c73855.graph_c0_358837** | c72022.graph_c0 | 836-855 Slice Site | 846 |
| **CPconservative_c73855.graph_c0_358837** | c75704.graph_c0 | 449-467 Slice Site | 458 |
| **CPconservative_c82330.graph_c0_451301** | c59317.graph_c0 | 1519-1537 Slice Site | 1528 |
| **CPconservative_c82330.graph_c0_451301** | c59643.graph_c0 | 40-60 Slice Site | 51 |
| **CPconservative_c82330.graph_c0_451301** | c63177.graph_c0 | 1788-1808 Slice Site | 1799 |
| **CPconservative_c82330.graph_c0_451301** | c68536.graph_c0 | 1902-1922 Slice Site | 1913 |
| **CPconservative_c82330.graph_c0_451301** | c71781.graph_c0 | 3184-3205 Slice Site | 3196 |
| **CPconservative_c82330.graph_c0_451301** | c71875.graph_c0 | 1936-1956 Slice Site | 1947 |
| **CPconservative_c82330.graph_c0_451301** | c74367.graph_c0 | 2599-2620 Slice Site | 2609 |
| **CPconservative_c82330.graph_c0_451301** | c74639.graph_c0 | 3277-3298 Slice Site | 3289 |
| **CPunconservative_c20003.graph_c0_31748** | c66353.graph_c0 | 1136-1156 Slice Site | 1147 |
| **CPunconservative_c31153.graph_c0_56796** | c69847.graph_c0 | 320-333 Slice Site | 324 |
| **CPunconservative_c38089.graph_c0_71335** | c100034.graph_c0 | 192-210 Slice Site | 201 |
| **CPunconservative_c38089.graph_c0_71335** | c102107.graph_c0 | 129-145 Slice Site | 137 |
| **CPunconservative_c38089.graph_c0_71335** | c19263.graph_c0 | 144-161 Slice Site | 152 |
| **CPunconservative_c38089.graph_c0_71335** | c19971.graph_c0 | 402-419 Slice Site | 410 |
| **CPunconservative_c38089.graph_c0_71335** | c27331.graph_c0 | 881-898 Slice Site | 889 |
| **CPunconservative_c38089.graph_c0_71335** | c29904.graph_c0 | 19-36 Slice Site | 27 |
| **CPunconservative_c38089.graph_c0_71335** | c30346.graph_c0 | 1-17 Slice Site | 8 |
| **CPunconservative_c38089.graph_c0_71335** | c31476.graph_c0 | 166-183 Slice Site | 174 |
| **CPunconservative_c38089.graph_c0_71335** | c36235.graph_c0 | 125-143 Slice Site | 134 |
| **CPunconservative_c38089.graph_c0_71335** | c41720.graph_c0 | 1314-1331 Slice Site | 1322 |
| **CPunconservative_c38089.graph_c0_71335** | c42513.graph_c0 | 372-388 Slice Site | 379 |
| **CPunconservative_c38089.graph_c0_71335** | c42940.graph_c0 | 321-337 Slice Site | 328 |
| **CPunconservative_c38089.graph_c0_71335** | c44122.graph_c0 | 247-264 Slice Site | 255 |
| **CPunconservative_c38089.graph_c0_71335** | c46411.graph_c0 | 2708-2724 Slice Site | 2715 |
| **CPunconservative_c38089.graph_c0_71335** | c47354.graph_c0 | 439-455 Slice Site | 447 |
| **CPunconservative_c38089.graph_c0_71335** | c48356.graph_c0 | 505-524 Slice Site | 514 |
| **CPunconservative_c38089.graph_c0_71335** | c52124.graph_c0 | 84-100 Slice Site | 91 |
| **CPunconservative_c38089.graph_c0_71335** | c52924.graph_c0 | 7-26 Slice Site | 17 |
| **CPunconservative_c38089.graph_c0_71335** | c53634.graph_c1 | 278-295 Slice Site | 286 |
| **CPunconservative_c38089.graph_c0_71335** | c53634.graph_c1 | 303-320 Slice Site | 311 |
| **CPunconservative_c38089.graph_c0_71335** | c54888.graph_c0 | 96-111 Slice Site | 102 |
| **CPunconservative_c38089.graph_c0_71335** | c55002.graph_c0 | 164-184 Slice Site | 173 |
| **CPunconservative_c38089.graph_c0_71335** | c55452.graph_c0 | 788-805 Slice Site | 796 |
| **CPunconservative_c38089.graph_c0_71335** | c55873.graph_c0 | 222-239 Slice Site | 230 |
| **CPunconservative_c38089.graph_c0_71335** | c56701.graph_c0 | 161-179 Slice Site | 170 |
| **CPunconservative_c38089.graph_c0_71335** | c57107.graph_c0 | 327-346 Slice Site | 337 |
| **CPunconservative_c38089.graph_c0_71335** | c57663.graph_c0 | 364-381 Slice Site | 372 |
| **CPunconservative_c38089.graph_c0_71335** | c57967.graph_c0 | 1103-1121 Slice Site | 1112 |
| **CPunconservative_c38089.graph_c0_71335** | c58315.graph_c0 | 162-179 Slice Site | 170 |
| **CPunconservative_c38089.graph_c0_71335** | c59586.graph_c0 | 613-631 Slice Site | 622 |
| **CPunconservative_c38089.graph_c0_71335** | c61194.graph_c0 | 518-534 Slice Site | 526 |
| **CPunconservative_c38089.graph_c0_71335** | c61241.graph_c0 | 367-383 Slice Site | 374 |
| **CPunconservative_c38089.graph_c0_71335** | c61484.graph_c0 | 362-379 Slice Site | 370 |
| **CPunconservative_c38089.graph_c0_71335** | c61493.graph_c0 | 130-147 Slice Site | 138 |
| **CPunconservative_c38089.graph_c0_71335** | c61663.graph_c0 | 492-512 Slice Site | 503 |
| **CPunconservative_c38089.graph_c0_71335** | c63573.graph_c0 | 151-169 Slice Site | 159 |
| **CPunconservative_c38089.graph_c0_71335** | c63818.graph_c0 | 297-314 Slice Site | 305 |
| **CPunconservative_c38089.graph_c0_71335** | c63922.graph_c0 | 2062-2078 Slice Site | 2069 |
| **CPunconservative_c38089.graph_c0_71335** | c64044.graph_c0 | 1781-1798 Slice Site | 1789 |
| **CPunconservative_c38089.graph_c0_71335** | c64326.graph_c0 | 276-294 Slice Site | 283 |
| **CPunconservative_c38089.graph_c0_71335** | c65099.graph_c2 | 378-396 Slice Site | 386 |
| **CPunconservative_c38089.graph_c0_71335** | c65316.graph_c0 | 389-406 Slice Site | 397 |
| **CPunconservative_c38089.graph_c0_71335** | c65575.graph_c0 | 73-90 Slice Site | 81 |
| **CPunconservative_c38089.graph_c0_71335** | c66138.graph_c0 | 218-236 Slice Site | 227 |
| **CPunconservative_c38089.graph_c0_71335** | c66241.graph_c1 | 115-133 Slice Site | 123 |
| **CPunconservative_c38089.graph_c0_71335** | c66616.graph_c0 | 2463-2479 Slice Site | 2471 |
| **CPunconservative_c38089.graph_c0_71335** | c66653.graph_c0 | 56-72 Slice Site | 64 |
| **CPunconservative_c38089.graph_c0_71335** | c67310.graph_c0 | 368-385 Slice Site | 376 |
| **CPunconservative_c38089.graph_c0_71335** | c67575.graph_c0 | 307-324 Slice Site | 315 |
| **CPunconservative_c38089.graph_c0_71335** | c68032.graph_c0 | 235-252 Slice Site | 243 |
| **CPunconservative_c38089.graph_c0_71335** | c68048.graph_c0 | 110-128 Slice Site | 118 |
| **CPunconservative_c38089.graph_c0_71335** | c68478.graph_c0 | 333-349 Slice Site | 341 |
| **CPunconservative_c38089.graph_c0_71335** | c68750.graph_c0 | 285-302 Slice Site | 293 |
| **CPunconservative_c38089.graph_c0_71335** | c69022.graph_c0 | 274-292 Slice Site | 282 |
| **CPunconservative_c38089.graph_c0_71335** | c70042.graph_c0 | 31-51 Slice Site | 41 |
| **CPunconservative_c38089.graph_c0_71335** | c70456.graph_c0 | 2961-2977 Slice Site | 2969 |
| **CPunconservative_c38089.graph_c0_71335** | c70896.graph_c1 | 109-123 Slice Site | 116 |
| **CPunconservative_c38089.graph_c0_71335** | c71112.graph_c0 | 847-863 Slice Site | 854 |
| **CPunconservative_c38089.graph_c0_71335** | c71276.graph_c0 | 2011-2028 Slice Site | 2019 |
| **CPunconservative_c38089.graph_c0_71335** | c71574.graph_c0 | 83-101 Slice Site | 92 |
| **CPunconservative_c38089.graph_c0_71335** | c72280.graph_c0 | 857-875 Slice Site | 865 |
| **CPunconservative_c38089.graph_c0_71335** | c72458.graph_c0 | 743-760 Slice Site | 751 |
| **CPunconservative_c38089.graph_c0_71335** | c72574.graph_c0 | 2498-2517 Slice Site | 2507 |
| **CPunconservative_c38089.graph_c0_71335** | c72793.graph_c0 | 164-181 Slice Site | 172 |
| **CPunconservative_c38089.graph_c0_71335** | c73777.graph_c0 | 1469-1485 Slice Site | 1476 |
| **CPunconservative_c38089.graph_c0_71335** | c73845.graph_c0 | 421-438 Slice Site | 429 |
| **CPunconservative_c38089.graph_c0_71335** | c73987.graph_c0 | 180-199 Slice Site | 190 |
| **CPunconservative_c38089.graph_c0_71335** | c74525.graph_c3 | 328-345 Slice Site | 336 |
| **CPunconservative_c38089.graph_c0_71335** | c74647.graph_c1 | 546-563 Slice Site | 554 |
| **CPunconservative_c38089.graph_c0_71335** | c74731.graph_c0 | 1758-1775 Slice Site | 1766 |
| **CPunconservative_c38089.graph_c0_71335** | c74795.graph_c0 | 3398-3415 Slice Site | 3406 |
| **CPunconservative_c38089.graph_c0_71335** | c74858.graph_c0 | 2122-2139 Slice Site | 2130 |
| **CPunconservative_c38089.graph_c0_71335** | c74858.graph_c0 | 2127-2144 Slice Site | 2135 |
| **CPunconservative_c38089.graph_c0_71335** | c75282.graph_c1 | 4744-4761 Slice Site | 4752 |
| **CPunconservative_c38089.graph_c0_71335** | c75568.graph_c1 | 177-197 Slice Site | 186 |
| **CPunconservative_c38089.graph_c0_71335** | c75689.graph_c0 | 3398-3413 Slice Site | 3406 |
| **CPunconservative_c38089.graph_c0_71335** | c75927.graph_c1 | 670-687 Slice Site | 678 |
| **CPunconservative_c38089.graph_c0_71335** | c76175.graph_c2 | 3784-3800 Slice Site | 3792 |
| **CPunconservative_c38089.graph_c0_71335** | c76681.graph_c0 | 22-38 Slice Site | 30 |
| **CPunconservative_c38089.graph_c0_71335** | c80833.graph_c0 | 212-230 Slice Site | 221 |
| **CPunconservative_c42080.graph_c0_80376** | c73056.graph_c0 | 2263-2286 Slice Site | 2277 |
| **CPunconservative_c65997.graph_c0_217806** | c75913.graph_c0 | 1767-1790 Slice Site | 1781 |
| **CPunconservative_c69995.graph_c0_278326** | c20671.graph_c0 | 807-828 Slice Site | 819 |
| **CPunconservative_c69995.graph_c0_278326** | c24900.graph_c0 | 375-395 Slice Site | 386 |
| **CPunconservative_c69995.graph_c0_278326** | c57553.graph_c0 | 898-917 Slice Site | 908 |
| **CPunconservative_c69995.graph_c0_278326** | c69664.graph_c0 | 3221-3241 Slice Site | 3232 |
| **CPunconservative_c69995.graph_c0_278326** | c72249.graph_c0 | 13-31 Slice Site | 22 |
| **CPunconservative_c69995.graph_c0_278326** | c72912.graph_c0 | 2093-2113 Slice Site | 2104 |
| **CPunconservative_c69995.graph_c0_278326** | c73078.graph_c0 | 1716-1737 Slice Site | 1728 |
| **CPunconservative_c69995.graph_c0_278326** | c73241.graph_c1 | 2-22 Slice Site | 13 |
| **CPunconservative_c69995.graph_c0_278326** | c75548.graph_c1 | 31-51 Slice Site | 42 |
| **CPunconservative_c69995.graph_c0_278326** | c76846.graph_c0 | 274-294 Slice Site | 286 |
| **CPunconservative_c73164.graph_c0_343143** | c73377.graph_c2 | 146-169 Slice Site | 160 |
| **CPunconservative_c73377.graph_c2_347857** | c42602.graph_c0 | 163-186 Slice Site | 177 |
| **CPunconservative_c74857.graph_c0_384696** | c66623.graph_c0 | 567-587 Slice Site | 578 |
| **CPunconservative_c78304.graph_c0_442247** | c66163.graph_c0 | 515-535 Slice Site | 526 |
| **CPunconservative_c78304.graph_c0_442247** | c70156.graph_c0 | 1020-1038 Slice Site | 1030 |
| **CPunconservative_c78304.graph_c0_442247** | c70770.graph_c0 | 386-407 Slice Site | 398 |
| **CZconservative_c12409.graph_c0_1611** | c67920.graph_c0 | 1246-1269 Slice Site | 1260 |
| **CZconservative_c27272.graph_c0_13642** | c36597.graph_c0 | 921-940 Slice Site | 931 |
| **CZconservative_c27272.graph_c0_13642** | c44031.graph_c0 | 400-418 Slice Site | 409 |
| **CZconservative_c27272.graph_c0_13642** | c52529.graph_c0 | 601-619 Slice Site | 610 |
| **CZconservative_c27272.graph_c0_13642** | c55977.graph_c0 | 932-950 Slice Site | 941 |
| **CZconservative_c27272.graph_c0_13642** | c58537.graph_c0 | 224-242 Slice Site | 233 |
| **CZconservative_c27272.graph_c0_13642** | c60725.graph_c0 | 237-255 Slice Site | 246 |
| **CZconservative_c27272.graph_c0_13642** | c61416.graph_c1 | 121-140 Slice Site | 131 |
| **CZconservative_c27272.graph_c0_13642** | c62685.graph_c0 | 228-245 Slice Site | 237 |
| **CZconservative_c27272.graph_c0_13642** | c65040.graph_c0 | 279-296 Slice Site | 287 |
| **CZconservative_c27272.graph_c0_13642** | c66163.graph_c0 | 36-53 Slice Site | 44 |
| **CZconservative_c27272.graph_c0_13642** | c67616.graph_c1 | 776-793 Slice Site | 785 |
| **CZconservative_c27272.graph_c0_13642** | c68339.graph_c0 | 22-40 Slice Site | 30 |
| **CZconservative_c27272.graph_c0_13642** | c69308.graph_c0 | 725-743 Slice Site | 734 |
| **CZconservative_c27272.graph_c0_13642** | c70005.graph_c0 | 2478-2495 Slice Site | 2486 |
| **CZconservative_c27272.graph_c0_13642** | c70861.graph_c0 | 641-658 Slice Site | 650 |
| **CZconservative_c27272.graph_c0_13642** | c71165.graph_c0 | 132-150 Slice Site | 141 |
| **CZconservative_c27272.graph_c0_13642** | c71590.graph_c0 | 5316-5336 Slice Site | 5327 |
| **CZconservative_c27272.graph_c0_13642** | c71911.graph_c1 | 807-825 Slice Site | 816 |
| **CZconservative_c27272.graph_c0_13642** | c72526.graph_c0 | 130-146 Slice Site | 138 |
| **CZconservative_c27272.graph_c0_13642** | c72908.graph_c0 | 163-181 Slice Site | 172 |
| **CZconservative_c27272.graph_c0_13642** | c72944.graph_c0 | 49-67 Slice Site | 58 |
| **CZconservative_c27272.graph_c0_13642** | c73216.graph_c0 | 3446-3463 Slice Site | 3454 |
| **CZconservative_c27272.graph_c0_13642** | c73726.graph_c0 | 3214-3232 Slice Site | 3223 |
| **CZconservative_c27272.graph_c0_13642** | c74406.graph_c0 | 1036-1054 Slice Site | 1045 |
| **CZconservative_c27272.graph_c0_13642** | c74594.graph_c0 | 479-497 Slice Site | 488 |
| **CZconservative_c30846.graph_c0_33849** | c45987.graph_c0 | 95-114 Slice Site | 105 |
| **CZconservative_c30846.graph_c0_33849** | c66163.graph_c0 | 60-80 Slice Site | 71 |
| **CZconservative_c30846.graph_c0_33849** | c70435.graph_c0 | 1394-1413 Slice Site | 1405 |
| **CZconservative_c30846.graph_c0_33849** | c74434.graph_c2 | 316-335 Slice Site | 327 |
| **CZconservative_c31373.graph_c0_37688** | c104541.graph_c0 | 42-61 Slice Site | 52 |
| **CZconservative_c31373.graph_c0_37688** | c114418.graph_c0 | 40-59 Slice Site | 49 |
| **CZconservative_c31373.graph_c0_37688** | c13928.graph_c0 | 2-21 Slice Site | 12 |
| **CZconservative_c31373.graph_c0_37688** | c14664.graph_c0 | 168-187 Slice Site | 178 |
| **CZconservative_c31373.graph_c0_37688** | c19321.graph_c0 | 8-27 Slice Site | 18 |
| **CZconservative_c31373.graph_c0_37688** | c19839.graph_c0 | 87-106 Slice Site | 97 |
| **CZconservative_c31373.graph_c0_37688** | c19855.graph_c1 | 53-72 Slice Site | 63 |
| **CZconservative_c31373.graph_c0_37688** | c19919.graph_c0 | 2-21 Slice Site | 12 |
| **CZconservative_c31373.graph_c0_37688** | c19923.graph_c0 | 1-20 Slice Site | 11 |
| **CZconservative_c31373.graph_c0_37688** | c19935.graph_c0 | 1-20 Slice Site | 11 |
| **CZconservative_c31373.graph_c0_37688** | c19947.graph_c0 | 3-22 Slice Site | 13 |
| **CZconservative_c31373.graph_c0_37688** | c19950.graph_c0 | 2-21 Slice Site | 12 |
| **CZconservative_c31373.graph_c0_37688** | c19972.graph_c0 | 45-66 Slice Site | 57 |
| **CZconservative_c31373.graph_c0_37688** | c20004.graph_c0 | 477-497 Slice Site | 488 |
| **CZconservative_c31373.graph_c0_37688** | c21282.graph_c0 | 2-21 Slice Site | 12 |
| **CZconservative_c31373.graph_c0_37688** | c24210.graph_c0 | 104-123 Slice Site | 114 |
| **CZconservative_c31373.graph_c0_37688** | c2673.graph_c0 | 120-140 Slice Site | 131 |
| **CZconservative_c31373.graph_c0_37688** | c28889.graph_c0 | 2-21 Slice Site | 12 |
| **CZconservative_c31373.graph_c0_37688** | c29904.graph_c0 | 161-180 Slice Site | 171 |
| **CZconservative_c31373.graph_c0_37688** | c31689.graph_c0 | 1-20 Slice Site | 11 |
| **CZconservative_c31373.graph_c0_37688** | c35959.graph_c0 | 2-21 Slice Site | 12 |
| **CZconservative_c31373.graph_c0_37688** | c37692.graph_c0 | 10-29 Slice Site | 20 |
| **CZconservative_c31373.graph_c0_37688** | c37736.graph_c0 | 479-498 Slice Site | 489 |
| **CZconservative_c31373.graph_c0_37688** | c40469.graph_c0 | 574-594 Slice Site | 584 |
| **CZconservative_c31373.graph_c0_37688** | c41960.graph_c0 | 24-43 Slice Site | 34 |
| **CZconservative_c31373.graph_c0_37688** | c42478.graph_c0 | 2-21 Slice Site | 12 |
| **CZconservative_c31373.graph_c0_37688** | c43719.graph_c0 | 210-229 Slice Site | 220 |
| **CZconservative_c31373.graph_c0_37688** | c44302.graph_c0 | 41-60 Slice Site | 51 |
| **CZconservative_c31373.graph_c0_37688** | c44633.graph_c0 | 1-20 Slice Site | 11 |
| **CZconservative_c31373.graph_c0_37688** | c44797.graph_c0 | 2-21 Slice Site | 12 |
| **CZconservative_c31373.graph_c0_37688** | c44830.graph_c0 | 2-21 Slice Site | 12 |
| **CZconservative_c31373.graph_c0_37688** | c45628.graph_c0 | 20-39 Slice Site | 30 |
| **CZconservative_c31373.graph_c0_37688** | c45735.graph_c0 | 100-119 Slice Site | 110 |
| **CZconservative_c31373.graph_c0_37688** | c45824.graph_c0 | 1-20 Slice Site | 11 |
| **CZconservative_c31373.graph_c0_37688** | c45966.graph_c0 | 49-69 Slice Site | 60 |
| **CZconservative_c31373.graph_c0_37688** | c46411.graph_c0 | 3214-3233 Slice Site | 3224 |
| **CZconservative_c31373.graph_c0_37688** | c47354.graph_c0 | 1058-1078 Slice Site | 1069 |
| **CZconservative_c31373.graph_c0_37688** | c48115.graph_c0 | 1-20 Slice Site | 11 |
| **CZconservative_c31373.graph_c0_37688** | c50051.graph_c0 | 93-113 Slice Site | 103 |
| **CZconservative_c31373.graph_c0_37688** | c50051.graph_c0 | 75-94 Slice Site | 85 |
| **CZconservative_c31373.graph_c0_37688** | c50223.graph_c0 | 1-20 Slice Site | 11 |
| **CZconservative_c31373.graph_c0_37688** | c51589.graph_c0 | 1-20 Slice Site | 11 |
| **CZconservative_c31373.graph_c0_37688** | c53255.graph_c0 | 931-950 Slice Site | 941 |
| **CZconservative_c31373.graph_c0_37688** | c53515.graph_c1 | 115-134 Slice Site | 125 |
| **CZconservative_c31373.graph_c0_37688** | c54000.graph_c0 | 1-20 Slice Site | 11 |
| **CZconservative_c31373.graph_c0_37688** | c54245.graph_c0 | 2-21 Slice Site | 12 |
| **CZconservative_c31373.graph_c0_37688** | c54310.graph_c0 | 1-20 Slice Site | 11 |
| **CZconservative_c31373.graph_c0_37688** | c54366.graph_c0 | 31-53 Slice Site | 44 |
| **CZconservative_c31373.graph_c0_37688** | c54398.graph_c0 | 65-84 Slice Site | 75 |
| **CZconservative_c31373.graph_c0_37688** | c54570.graph_c0 | 1-20 Slice Site | 11 |
| **CZconservative_c31373.graph_c0_37688** | c54594.graph_c0 | 3-22 Slice Site | 13 |
| **CZconservative_c31373.graph_c0_37688** | c55059.graph_c0 | 260-279 Slice Site | 270 |
| **CZconservative_c31373.graph_c0_37688** | c55590.graph_c0 | 74-93 Slice Site | 84 |
| **CZconservative_c31373.graph_c0_37688** | c56056.graph_c0 | 11-29 Slice Site | 20 |
| **CZconservative_c31373.graph_c0_37688** | c56125.graph_c0 | 261-281 Slice Site | 272 |
| **CZconservative_c31373.graph_c0_37688** | c56909.graph_c0 | 2-21 Slice Site | 12 |
| **CZconservative_c31373.graph_c0_37688** | c57130.graph_c0 | 596-617 Slice Site | 608 |
| **CZconservative_c31373.graph_c0_37688** | c57152.graph_c0 | 1846-1866 Slice Site | 1857 |
| **CZconservative_c31373.graph_c0_37688** | c57159.graph_c1 | 3-22 Slice Site | 13 |
| **CZconservative_c31373.graph_c0_37688** | c57171.graph_c0 | 15-34 Slice Site | 25 |
| **CZconservative_c31373.graph_c0_37688** | c57222.graph_c0 | 119-138 Slice Site | 129 |
| **CZconservative_c31373.graph_c0_37688** | c57223.graph_c0 | 2-21 Slice Site | 12 |
| **CZconservative_c31373.graph_c0_37688** | c57557.graph_c0 | 21-40 Slice Site | 31 |
| **CZconservative_c31373.graph_c0_37688** | c60343.graph_c0 | 1-20 Slice Site | 11 |
| **CZconservative_c31373.graph_c0_37688** | c60372.graph_c0 | 112-130 Slice Site | 121 |
| **CZconservative_c31373.graph_c0_37688** | c60415.graph_c0 | 2-21 Slice Site | 12 |
| **CZconservative_c31373.graph_c0_37688** | c60422.graph_c1 | 1-20 Slice Site | 11 |
| **CZconservative_c31373.graph_c0_37688** | c60522.graph_c0 | 375-395 Slice Site | 386 |
| **CZconservative_c31373.graph_c0_37688** | c61043.graph_c0 | 115-135 Slice Site | 125 |
| **CZconservative_c31373.graph_c0_37688** | c61055.graph_c0 | 1-20 Slice Site | 11 |
| **CZconservative_c31373.graph_c0_37688** | c61194.graph_c0 | 183-202 Slice Site | 193 |
| **CZconservative_c31373.graph_c0_37688** | c61241.graph_c0 | 637-657 Slice Site | 647 |
| **CZconservative_c31373.graph_c0_37688** | c61970.graph_c0 | 764-783 Slice Site | 774 |
| **CZconservative_c31373.graph_c0_37688** | c61975.graph_c0 | 337-356 Slice Site | 347 |
| **CZconservative_c31373.graph_c0_37688** | c62390.graph_c0 | 380-399 Slice Site | 390 |
| **CZconservative_c31373.graph_c0_37688** | c62586.graph_c0 | 2-21 Slice Site | 12 |
| **CZconservative_c31373.graph_c0_37688** | c62633.graph_c0 | 2-21 Slice Site | 12 |
| **CZconservative_c31373.graph_c0_37688** | c62860.graph_c0 | 1-20 Slice Site | 11 |
| **CZconservative_c31373.graph_c0_37688** | c62918.graph_c0 | 2-21 Slice Site | 12 |
| **CZconservative_c31373.graph_c0_37688** | c62954.graph_c0 | 1-20 Slice Site | 11 |
| **CZconservative_c31373.graph_c0_37688** | c62954.graph_c0 | 3-22 Slice Site | 13 |
| **CZconservative_c31373.graph_c0_37688** | c63434.graph_c0 | 1-20 Slice Site | 11 |
| **CZconservative_c31373.graph_c0_37688** | c63497.graph_c0 | 41-60 Slice Site | 51 |
| **CZconservative_c31373.graph_c0_37688** | c63577.graph_c0 | 639-658 Slice Site | 649 |
| **CZconservative_c31373.graph_c0_37688** | c64122.graph_c0 | 1-20 Slice Site | 11 |
| **CZconservative_c31373.graph_c0_37688** | c64141.graph_c0 | 466-485 Slice Site | 476 |
| **CZconservative_c31373.graph_c0_37688** | c64193.graph_c0 | 1-20 Slice Site | 11 |
| **CZconservative_c31373.graph_c0_37688** | c64211.graph_c0 | 1-20 Slice Site | 11 |
| **CZconservative_c31373.graph_c0_37688** | c64334.graph_c0 | 176-195 Slice Site | 186 |
| **CZconservative_c31373.graph_c0_37688** | c64620.graph_c1 | 351-370 Slice Site | 361 |
| **CZconservative_c31373.graph_c0_37688** | c65209.graph_c0 | 395-414 Slice Site | 405 |
| **CZconservative_c31373.graph_c0_37688** | c65361.graph_c0 | 162-180 Slice Site | 171 |
| **CZconservative_c31373.graph_c0_37688** | c65484.graph_c0 | 1-20 Slice Site | 11 |
| **CZconservative_c31373.graph_c0_37688** | c65503.graph_c0 | 382-402 Slice Site | 392 |
| **CZconservative_c31373.graph_c0_37688** | c65542.graph_c0 | 2-21 Slice Site | 12 |
| **CZconservative_c31373.graph_c0_37688** | c65550.graph_c1 | 1331-1352 Slice Site | 1343 |
| **CZconservative_c31373.graph_c0_37688** | c65620.graph_c0 | 1-20 Slice Site | 11 |
| **CZconservative_c31373.graph_c0_37688** | c65721.graph_c0 | 1-20 Slice Site | 11 |
| **CZconservative_c31373.graph_c0_37688** | c65994.graph_c0 | 35-54 Slice Site | 45 |
| **CZconservative_c31373.graph_c0_37688** | c66008.graph_c0 | 77-95 Slice Site | 86 |
| **CZconservative_c31373.graph_c0_37688** | c66048.graph_c1 | 2-21 Slice Site | 12 |
| **CZconservative_c31373.graph_c0_37688** | c66061.graph_c0 | 76-95 Slice Site | 86 |
| **CZconservative_c31373.graph_c0_37688** | c66155.graph_c0 | 1-20 Slice Site | 11 |
| **CZconservative_c31373.graph_c0_37688** | c66237.graph_c0 | 1179-1198 Slice Site | 1189 |
| **CZconservative_c31373.graph_c0_37688** | c66351.graph_c0 | 1109-1130 Slice Site | 1121 |
| **CZconservative_c31373.graph_c0_37688** | c66365.graph_c0 | 3-22 Slice Site | 13 |
| **CZconservative_c31373.graph_c0_37688** | c66768.graph_c0 | 40-58 Slice Site | 49 |
| **CZconservative_c31373.graph_c0_37688** | c66814.graph_c0 | 2-21 Slice Site | 12 |
| **CZconservative_c31373.graph_c0_37688** | c66898.graph_c0 | 2-21 Slice Site | 12 |
| **CZconservative_c31373.graph_c0_37688** | c66985.graph_c0 | 48-67 Slice Site | 58 |
| **CZconservative_c31373.graph_c0_37688** | c67024.graph_c0 | 11-30 Slice Site | 21 |
| **CZconservative_c31373.graph_c0_37688** | c67693.graph_c0 | 1-20 Slice Site | 11 |
| **CZconservative_c31373.graph_c0_37688** | c67758.graph_c0 | 1-20 Slice Site | 11 |
| **CZconservative_c31373.graph_c0_37688** | c68033.graph_c0 | 3-22 Slice Site | 13 |
| **CZconservative_c31373.graph_c0_37688** | c68064.graph_c0 | 363-382 Slice Site | 373 |
| **CZconservative_c31373.graph_c0_37688** | c68298.graph_c0 | 1-20 Slice Site | 11 |
| **CZconservative_c31373.graph_c0_37688** | c68355.graph_c0 | 3-22 Slice Site | 13 |
| **CZconservative_c31373.graph_c0_37688** | c68420.graph_c0 | 94-112 Slice Site | 103 |
| **CZconservative_c31373.graph_c0_37688** | c68480.graph_c1 | 91-109 Slice Site | 100 |
| **CZconservative_c31373.graph_c0_37688** | c68481.graph_c2 | 95-115 Slice Site | 106 |
| **CZconservative_c31373.graph_c0_37688** | c68775.graph_c0 | 2-21 Slice Site | 12 |
| **CZconservative_c31373.graph_c0_37688** | c68887.graph_c0 | 40-62 Slice Site | 53 |
| **CZconservative_c31373.graph_c0_37688** | c69074.graph_c0 | 1931-1950 Slice Site | 1941 |
| **CZconservative_c31373.graph_c0_37688** | c69188.graph_c0 | 995-1013 Slice Site | 1004 |
| **CZconservative_c31373.graph_c0_37688** | c69257.graph_c0 | 288-309 Slice Site | 300 |
| **CZconservative_c31373.graph_c0_37688** | c69357.graph_c0 | 2-21 Slice Site | 12 |
| **CZconservative_c31373.graph_c0_37688** | c69397.graph_c0 | 1868-1887 Slice Site | 1878 |
| **CZconservative_c31373.graph_c0_37688** | c69433.graph_c0 | 1-20 Slice Site | 11 |
| **CZconservative_c31373.graph_c0_37688** | c69439.graph_c0 | 2-21 Slice Site | 12 |
| **CZconservative_c31373.graph_c0_37688** | c69586.graph_c0 | 120-139 Slice Site | 130 |
| **CZconservative_c31373.graph_c0_37688** | c69600.graph_c0 | 162-182 Slice Site | 172 |
| **CZconservative_c31373.graph_c0_37688** | c69609.graph_c0 | 1-20 Slice Site | 11 |
| **CZconservative_c31373.graph_c0_37688** | c69609.graph_c0 | 3-22 Slice Site | 13 |
| **CZconservative_c31373.graph_c0_37688** | c69772.graph_c0 | 1-20 Slice Site | 11 |
| **CZconservative_c31373.graph_c0_37688** | c69782.graph_c0 | 2-21 Slice Site | 12 |
| **CZconservative_c31373.graph_c0_37688** | c69918.graph_c0 | 146-165 Slice Site | 156 |
| **CZconservative_c31373.graph_c0_37688** | c70013.graph_c0 | 72-91 Slice Site | 82 |
| **CZconservative_c31373.graph_c0_37688** | c70126.graph_c0 | 89-109 Slice Site | 100 |
| **CZconservative_c31373.graph_c0_37688** | c70150.graph_c0 | 2-21 Slice Site | 12 |
| **CZconservative_c31373.graph_c0_37688** | c70264.graph_c0 | 78-97 Slice Site | 88 |
| **CZconservative_c31373.graph_c0_37688** | c70947.graph_c1 | 274-290 Slice Site | 282 |
| **CZconservative_c31373.graph_c0_37688** | c71082.graph_c1 | 1-20 Slice Site | 11 |
| **CZconservative_c31373.graph_c0_37688** | c71290.graph_c0 | 126-148 Slice Site | 139 |
| **CZconservative_c31373.graph_c0_37688** | c71298.graph_c0 | 421-440 Slice Site | 431 |
| **CZconservative_c31373.graph_c0_37688** | c71328.graph_c1 | 1-20 Slice Site | 11 |
| **CZconservative_c31373.graph_c0_37688** | c71396.graph_c0 | 7-25 Slice Site | 16 |
| **CZconservative_c31373.graph_c0_37688** | c71472.graph_c0 | 1-20 Slice Site | 11 |
| **CZconservative_c31373.graph_c0_37688** | c71482.graph_c0 | 30-50 Slice Site | 41 |
| **CZconservative_c31373.graph_c0_37688** | c71558.graph_c0 | 5-23 Slice Site | 14 |
| **CZconservative_c31373.graph_c0_37688** | c71778.graph_c1 | 458-477 Slice Site | 468 |
| **CZconservative_c31373.graph_c0_37688** | c71853.graph_c2 | 2-21 Slice Site | 12 |
| **CZconservative_c31373.graph_c0_37688** | c72001.graph_c0 | 216-234 Slice Site | 225 |
| **CZconservative_c31373.graph_c0_37688** | c72095.graph_c1 | 40-60 Slice Site | 51 |
| **CZconservative_c31373.graph_c0_37688** | c72111.graph_c0 | 138-157 Slice Site | 148 |
| **CZconservative_c31373.graph_c0_37688** | c72194.graph_c0 | 44-63 Slice Site | 54 |
| **CZconservative_c31373.graph_c0_37688** | c72309.graph_c0 | 68-88 Slice Site | 79 |
| **CZconservative_c31373.graph_c0_37688** | c72314.graph_c0 | 3-22 Slice Site | 13 |
| **CZconservative_c31373.graph_c0_37688** | c72381.graph_c0 | 1632-1651 Slice Site | 1642 |
| **CZconservative_c31373.graph_c0_37688** | c72381.graph_c0 | 1634-1653 Slice Site | 1644 |
| **CZconservative_c31373.graph_c0_37688** | c72537.graph_c0 | 3548-3566 Slice Site | 3557 |
| **CZconservative_c31373.graph_c0_37688** | c72570.graph_c0 | 1-20 Slice Site | 11 |
| **CZconservative_c31373.graph_c0_37688** | c72693.graph_c0 | 1550-1568 Slice Site | 1559 |
| **CZconservative_c31373.graph_c0_37688** | c72718.graph_c0 | 606-625 Slice Site | 616 |
| **CZconservative_c31373.graph_c0_37688** | c72735.graph_c0 | 1-20 Slice Site | 11 |
| **CZconservative_c31373.graph_c0_37688** | c72810.graph_c0 | 1650-1669 Slice Site | 1660 |
| **CZconservative_c31373.graph_c0_37688** | c72989.graph_c0 | 2-21 Slice Site | 12 |
| **CZconservative_c31373.graph_c0_37688** | c72995.graph_c0 | 243-264 Slice Site | 255 |
| **CZconservative_c31373.graph_c0_37688** | c73009.graph_c0 | 33-53 Slice Site | 44 |
| **CZconservative_c31373.graph_c0_37688** | c73250.graph_c0 | 2-21 Slice Site | 12 |
| **CZconservative_c31373.graph_c0_37688** | c73250.graph_c0 | 4-23 Slice Site | 14 |
| **CZconservative_c31373.graph_c0_37688** | c73260.graph_c0 | 1745-1765 Slice Site | 1756 |
| **CZconservative_c31373.graph_c0_37688** | c73407.graph_c0 | 2104-2124 Slice Site | 2115 |
| **CZconservative_c31373.graph_c0_37688** | c73456.graph_c1 | 237-256 Slice Site | 247 |
| **CZconservative_c31373.graph_c0_37688** | c73469.graph_c0 | 24-43 Slice Site | 34 |
| **CZconservative_c31373.graph_c0_37688** | c73698.graph_c0 | 145-164 Slice Site | 155 |
| **CZconservative_c31373.graph_c0_37688** | c74024.graph_c0 | 440-458 Slice Site | 449 |
| **CZconservative_c31373.graph_c0_37688** | c74035.graph_c0 | 1-20 Slice Site | 11 |
| **CZconservative_c31373.graph_c0_37688** | c74328.graph_c1 | 4008-4027 Slice Site | 4018 |
| **CZconservative_c31373.graph_c0_37688** | c74506.graph_c0 | 1554-1573 Slice Site | 1564 |
| **CZconservative_c31373.graph_c0_37688** | c74554.graph_c1 | 2260-2279 Slice Site | 2270 |
| **CZconservative_c31373.graph_c0_37688** | c74634.graph_c1 | 569-588 Slice Site | 579 |
| **CZconservative_c31373.graph_c0_37688** | c74751.graph_c0 | 102-121 Slice Site | 112 |
| **CZconservative_c31373.graph_c0_37688** | c74868.graph_c0 | 158-177 Slice Site | 168 |
| **CZconservative_c31373.graph_c0_37688** | c74966.graph_c1 | 4094-4114 Slice Site | 4105 |
| **CZconservative_c31373.graph_c0_37688** | c75248.graph_c0 | 682-700 Slice Site | 692 |
| **CZconservative_c31373.graph_c0_37688** | c75467.graph_c0 | 117-136 Slice Site | 127 |
| **CZconservative_c31373.graph_c0_37688** | c75493.graph_c0 | 1098-1117 Slice Site | 1108 |
| **CZconservative_c31373.graph_c0_37688** | c75548.graph_c3 | 1-20 Slice Site | 11 |
| **CZconservative_c31373.graph_c0_37688** | c75749.graph_c0 | 61-79 Slice Site | 70 |
| **CZconservative_c31373.graph_c0_37688** | c75881.graph_c0 | 1-20 Slice Site | 11 |
| **CZconservative_c31373.graph_c0_37688** | c76027.graph_c0 | 833-852 Slice Site | 843 |
| **CZconservative_c31373.graph_c0_37688** | c76049.graph_c2 | 123-145 Slice Site | 136 |
| **CZconservative_c31373.graph_c0_37688** | c76283.graph_c0 | 1271-1290 Slice Site | 1281 |
| **CZconservative_c31373.graph_c0_37688** | c76386.graph_c0 | 22-41 Slice Site | 32 |
| **CZconservative_c31373.graph_c0_37688** | c76407.graph_c0 | 47-65 Slice Site | 56 |
| **CZconservative_c31373.graph_c0_37688** | c76434.graph_c0 | 76-95 Slice Site | 86 |
| **CZconservative_c31373.graph_c0_37688** | c76608.graph_c0 | 31-50 Slice Site | 41 |
| **CZconservative_c31373.graph_c0_37688** | c76946.graph_c0 | 139-160 Slice Site | 151 |
| **CZconservative_c31373.graph_c0_37688** | c81706.graph_c0 | 160-179 Slice Site | 170 |
| **CZconservative_c31373.graph_c0_37688** | c93027.graph_c0 | 121-140 Slice Site | 131 |
| **CZconservative_c3204.graph_c0_41860** | c69679.graph_c2 | 1384-1403 Slice Site | 1394 |
| **CZconservative_c32677.graph_c0_45729** | c43172.graph_c0 | 245-265 Slice Site | 257 |
| **CZconservative_c32677.graph_c0_45729** | c53375.graph_c0 | 1046-1065 Slice Site | 1057 |
| **CZconservative_c32677.graph_c0_45729** | c60557.graph_c0 | 494-514 Slice Site | 505 |
| **CZconservative_c32677.graph_c0_45729** | c67468.graph_c0 | 1477-1497 Slice Site | 1488 |
| **CZconservative_c32677.graph_c0_45729** | c69827.graph_c0 | 1629-1649 Slice Site | 1640 |
| **CZconservative_c32677.graph_c0_45729** | c73704.graph_c0 | 1528-1547 Slice Site | 1539 |
| **CZconservative_c34554.graph_c0_60739** | c66582.graph_c0 | 2014-2035 Slice Site | 2026 |
| **CZconservative_c39575.graph_c0_84107** | c32535.graph_c0 | 209-229 Slice Site | 220 |
| **CZconservative_c42026.graph_c0_93222** | c65522.graph_c0 | 1352-1371 Slice Site | 1362 |
| **CZconservative_c42026.graph_c0_93222** | c70827.graph_c0 | 119-138 Slice Site | 129 |
| **CZconservative_c42026.graph_c0_93222** | c89900.graph_c0 | 274-294 Slice Site | 285 |
| **CZconservative_c50404.graph_c0_138982** | c65556.graph_c0 | 1581-1602 Slice Site | 1593 |
| **CZconservative_c53910.graph_c0_165739** | c69443.graph_c0 | 160-180 Slice Site | 172 |
| **CZconservative_c53910.graph_c0_165739** | c69828.graph_c0 | 1220-1240 Slice Site | 1232 |
| **CZconservative_c57766.graph_c0_209195** | c76154.graph_c0 | 5327-5349 Slice Site | 5340 |
| **CZconservative_c61140.graph_c0_258415** | c20039.graph_c0 | 607-629 Slice Site | 620 |
| **CZconservative_c61140.graph_c0_258415** | c46270.graph_c1 | 75-94 Slice Site | 85 |
| **CZconservative_c61140.graph_c0_258415** | c57326.graph_c0 | 65-86 Slice Site | 77 |
| **CZconservative_c61140.graph_c0_258415** | c69122.graph_c0 | 1464-1485 Slice Site | 1476 |
| **CZconservative_c61140.graph_c0_258415** | c76511.graph_c0 | 200-222 Slice Site | 213 |
| **CZconservative_c61500.graph_c0_264442** | c18466.graph_c0 | 235-258 Slice Site | 250 |
| **CZconservative_c61500.graph_c0_264442** | c19714.graph_c0 | 2-26 Slice Site | 16 |
| **CZconservative_c61500.graph_c0_264442** | c20373.graph_c0 | 2-26 Slice Site | 16 |
| **CZconservative_c61500.graph_c0_264442** | c20671.graph_c0 | 853-876 Slice Site | 867 |
| **CZconservative_c61500.graph_c0_264442** | c22225.graph_c0 | 191-213 Slice Site | 204 |
| **CZconservative_c61500.graph_c0_264442** | c27614.graph_c0 | 166-187 Slice Site | 178 |
| **CZconservative_c61500.graph_c0_264442** | c28771.graph_c0 | 121-144 Slice Site | 135 |
| **CZconservative_c61500.graph_c0_264442** | c37464.graph_c0 | 64-88 Slice Site | 79 |
| **CZconservative_c61500.graph_c0_264442** | c39805.graph_c0 | 76-101 Slice Site | 92 |
| **CZconservative_c61500.graph_c0_264442** | c40210.graph_c0 | 122-144 Slice Site | 136 |
| **CZconservative_c61500.graph_c0_264442** | c41676.graph_c0 | 2-26 Slice Site | 16 |
| **CZconservative_c61500.graph_c0_264442** | c43395.graph_c0 | 3-26 Slice Site | 17 |
| **CZconservative_c61500.graph_c0_264442** | c43597.graph_c0 | 2-25 Slice Site | 16 |
| **CZconservative_c61500.graph_c0_264442** | c45369.graph_c0 | 6-30 Slice Site | 20 |
| **CZconservative_c61500.graph_c0_264442** | c45628.graph_c0 | 135-158 Slice Site | 149 |
| **CZconservative_c61500.graph_c0_264442** | c46006.graph_c0 | 109-133 Slice Site | 124 |
| **CZconservative_c61500.graph_c0_264442** | c50309.graph_c0 | 1-24 Slice Site | 15 |
| **CZconservative_c61500.graph_c0_264442** | c52589.graph_c0 | 1-24 Slice Site | 15 |
| **CZconservative_c61500.graph_c0_264442** | c54000.graph_c0 | 4-27 Slice Site | 18 |
| **CZconservative_c61500.graph_c0_264442** | c54127.graph_c0 | 660-683 Slice Site | 674 |
| **CZconservative_c61500.graph_c0_264442** | c54310.graph_c0 | 4-27 Slice Site | 18 |
| **CZconservative_c61500.graph_c0_264442** | c54821.graph_c0 | 4-28 Slice Site | 18 |
| **CZconservative_c61500.graph_c0_264442** | c54915.graph_c0 | 1814-1837 Slice Site | 1828 |
| **CZconservative_c61500.graph_c0_264442** | c56017.graph_c0 | 6-30 Slice Site | 20 |
| **CZconservative_c61500.graph_c0_264442** | c56056.graph_c0 | 13-36 Slice Site | 27 |
| **CZconservative_c61500.graph_c0_264442** | c56243.graph_c0 | 1-24 Slice Site | 15 |
| **CZconservative_c61500.graph_c0_264442** | c57223.graph_c0 | 85-110 Slice Site | 101 |
| **CZconservative_c61500.graph_c0_264442** | c57223.graph_c0 | 3-27 Slice Site | 17 |
| **CZconservative_c61500.graph_c0_264442** | c57258.graph_c0 | 1-24 Slice Site | 15 |
| **CZconservative_c61500.graph_c0_264442** | c57472.graph_c0 | 5-28 Slice Site | 19 |
| **CZconservative_c61500.graph_c0_264442** | c57488.graph_c0 | 3-27 Slice Site | 17 |
| **CZconservative_c61500.graph_c0_264442** | c57561.graph_c1 | 2695-2719 Slice Site | 2709 |
| **CZconservative_c61500.graph_c0_264442** | c58782.graph_c0 | 44-67 Slice Site | 58 |
| **CZconservative_c61500.graph_c0_264442** | c59567.graph_c0 | 1-25 Slice Site | 15 |
| **CZconservative_c61500.graph_c0_264442** | c59655.graph_c0 | 4-29 Slice Site | 20 |
| **CZconservative_c61500.graph_c0_264442** | c60321.graph_c0 | 73-95 Slice Site | 87 |
| **CZconservative_c61500.graph_c0_264442** | c60672.graph_c0 | 44-68 Slice Site | 58 |
| **CZconservative_c61500.graph_c0_264442** | c61202.graph_c0 | 663-686 Slice Site | 677 |
| **CZconservative_c61500.graph_c0_264442** | c61307.graph_c0 | 421-444 Slice Site | 435 |
| **CZconservative_c61500.graph_c0_264442** | c61446.graph_c0 | 727-751 Slice Site | 742 |
| **CZconservative_c61500.graph_c0_264442** | c61823.graph_c0 | 6-29 Slice Site | 20 |
| **CZconservative_c61500.graph_c0_264442** | c62353.graph_c0 | 1-24 Slice Site | 15 |
| **CZconservative_c61500.graph_c0_264442** | c62586.graph_c0 | 1-25 Slice Site | 15 |
| **CZconservative_c61500.graph_c0_264442** | c62696.graph_c0 | 1-24 Slice Site | 15 |
| **CZconservative_c61500.graph_c0_264442** | c62720.graph_c0 | 2-25 Slice Site | 16 |
| **CZconservative_c61500.graph_c0_264442** | c62814.graph_c0 | 214-237 Slice Site | 228 |
| **CZconservative_c61500.graph_c0_264442** | c62918.graph_c0 | 7-30 Slice Site | 21 |
| **CZconservative_c61500.graph_c0_264442** | c64016.graph_c0 | 590-612 Slice Site | 603 |
| **CZconservative_c61500.graph_c0_264442** | c64290.graph_c0 | 2-25 Slice Site | 16 |
| **CZconservative_c61500.graph_c0_264442** | c64348.graph_c0 | 2394-2418 Slice Site | 2409 |
| **CZconservative_c61500.graph_c0_264442** | c64451.graph_c0 | 2-25 Slice Site | 16 |
| **CZconservative_c61500.graph_c0_264442** | c64548.graph_c0 | 1-23 Slice Site | 15 |
| **CZconservative_c61500.graph_c0_264442** | c64620.graph_c1 | 362-386 Slice Site | 376 |
| **CZconservative_c61500.graph_c0_264442** | c65354.graph_c0 | 2-26 Slice Site | 16 |
| **CZconservative_c61500.graph_c0_264442** | c66237.graph_c0 | 1184-1207 Slice Site | 1198 |
| **CZconservative_c61500.graph_c0_264442** | c66364.graph_c1 | 1-25 Slice Site | 15 |
| **CZconservative_c61500.graph_c0_264442** | c67041.graph_c0 | 2096-2118 Slice Site | 2110 |
| **CZconservative_c61500.graph_c0_264442** | c67737.graph_c0 | 2-27 Slice Site | 16 |
| **CZconservative_c61500.graph_c0_264442** | c67895.graph_c0 | 1-25 Slice Site | 15 |
| **CZconservative_c61500.graph_c0_264442** | c67909.graph_c0 | 2-25 Slice Site | 16 |
| **CZconservative_c61500.graph_c0_264442** | c68105.graph_c0 | 2-26 Slice Site | 16 |
| **CZconservative_c61500.graph_c0_264442** | c68466.graph_c1 | 179-204 Slice Site | 194 |
| **CZconservative_c61500.graph_c0_264442** | c68715.graph_c0 | 1156-1179 Slice Site | 1170 |
| **CZconservative_c61500.graph_c0_264442** | c68715.graph_c0 | 1195-1218 Slice Site | 1209 |
| **CZconservative_c61500.graph_c0_264442** | c69084.graph_c0 | 1-24 Slice Site | 15 |
| **CZconservative_c61500.graph_c0_264442** | c69116.graph_c1 | 3-26 Slice Site | 17 |
| **CZconservative_c61500.graph_c0_264442** | c69122.graph_c0 | 139-163 Slice Site | 154 |
| **CZconservative_c61500.graph_c0_264442** | c69357.graph_c0 | 1-25 Slice Site | 15 |
| **CZconservative_c61500.graph_c0_264442** | c70150.graph_c0 | 9-32 Slice Site | 23 |
| **CZconservative_c61500.graph_c0_264442** | c70198.graph_c0 | 1-25 Slice Site | 15 |
| **CZconservative_c61500.graph_c0_264442** | c70264.graph_c0 | 3-26 Slice Site | 17 |
| **CZconservative_c61500.graph_c0_264442** | c70489.graph_c0 | 1036-1058 Slice Site | 1049 |
| **CZconservative_c61500.graph_c0_264442** | c70644.graph_c1 | 1-24 Slice Site | 15 |
| **CZconservative_c61500.graph_c0_264442** | c70660.graph_c4 | 90-113 Slice Site | 104 |
| **CZconservative_c61500.graph_c0_264442** | c71063.graph_c0 | 2-26 Slice Site | 16 |
| **CZconservative_c61500.graph_c0_264442** | c71073.graph_c0 | 2-25 Slice Site | 16 |
| **CZconservative_c61500.graph_c0_264442** | c71179.graph_c0 | 4-28 Slice Site | 18 |
| **CZconservative_c61500.graph_c0_264442** | c71472.graph_c0 | 6-30 Slice Site | 20 |
| **CZconservative_c61500.graph_c0_264442** | c71476.graph_c0 | 206-230 Slice Site | 220 |
| **CZconservative_c61500.graph_c0_264442** | c71476.graph_c1 | 2-25 Slice Site | 16 |
| **CZconservative_c61500.graph_c0_264442** | c71517.graph_c1 | 1-24 Slice Site | 15 |
| **CZconservative_c61500.graph_c0_264442** | c71549.graph_c0 | 3-26 Slice Site | 17 |
| **CZconservative_c61500.graph_c0_264442** | c71575.graph_c0 | 136-159 Slice Site | 150 |
| **CZconservative_c61500.graph_c0_264442** | c72322.graph_c0 | 3-26 Slice Site | 17 |
| **CZconservative_c61500.graph_c0_264442** | c72381.graph_c0 | 1631-1655 Slice Site | 1645 |
| **CZconservative_c61500.graph_c0_264442** | c72691.graph_c1 | 2-25 Slice Site | 16 |
| **CZconservative_c61500.graph_c0_264442** | c72840.graph_c0 | 215-239 Slice Site | 229 |
| **CZconservative_c61500.graph_c0_264442** | c73026.graph_c0 | 1099-1122 Slice Site | 1113 |
| **CZconservative_c61500.graph_c0_264442** | c73408.graph_c0 | 44-67 Slice Site | 58 |
| **CZconservative_c61500.graph_c0_264442** | c73408.graph_c0 | 80-103 Slice Site | 94 |
| **CZconservative_c61500.graph_c0_264442** | c73698.graph_c0 | 142-166 Slice Site | 156 |
| **CZconservative_c61500.graph_c0_264442** | c73704.graph_c0 | 125-148 Slice Site | 139 |
| **CZconservative_c61500.graph_c0_264442** | c73730.graph_c2 | 2-26 Slice Site | 16 |
| **CZconservative_c61500.graph_c0_264442** | c73873.graph_c0 | 1-24 Slice Site | 15 |
| **CZconservative_c61500.graph_c0_264442** | c74365.graph_c0 | 2-25 Slice Site | 16 |
| **CZconservative_c61500.graph_c0_264442** | c74630.graph_c0 | 5555-5578 Slice Site | 5569 |
| **CZconservative_c61500.graph_c0_264442** | c74731.graph_c0 | 319-342 Slice Site | 333 |
| **CZconservative_c61500.graph_c0_264442** | c74780.graph_c3 | 40-62 Slice Site | 53 |
| **CZconservative_c61500.graph_c0_264442** | c75031.graph_c1 | 9-32 Slice Site | 23 |
| **CZconservative_c61500.graph_c0_264442** | c75548.graph_c3 | 6-29 Slice Site | 20 |
| **CZconservative_c61500.graph_c0_264442** | c75750.graph_c2 | 9-33 Slice Site | 23 |
| **CZconservative_c61500.graph_c0_264442** | c76161.graph_c1 | 1194-1220 Slice Site | 1210 |
| **CZconservative_c61500.graph_c0_264442** | c76386.graph_c0 | 7-30 Slice Site | 21 |
| **CZconservative_c61500.graph_c0_264442** | c76562.graph_c0 | 87-110 Slice Site | 101 |
| **CZconservative_c61500.graph_c0_264442** | c76608.graph_c0 | 36-59 Slice Site | 50 |
| **CZconservative_c61500.graph_c0_264442** | c76633.graph_c0 | 1-25 Slice Site | 15 |
| **CZconservative_c61500.graph_c0_264442** | c77098.graph_c0 | 4-28 Slice Site | 18 |
| **CZconservative_c61500.graph_c0_264442** | c88640.graph_c0 | 131-154 Slice Site | 145 |
| **CZconservative_c62980.graph_c0_292527** | c65689.graph_c0 | 772-792 Slice Site | 784 |
| **CZconservative_c62980.graph_c0_292527** | c73807.graph_c0 | 2155-2178 Slice Site | 2169 |
| **CZconservative_c64733.graph_c0_331102** | c39503.graph_c0 | 344-363 Slice Site | 354 |
| **CZconservative_c64733.graph_c0_331102** | c64443.graph_c0 | 1783-1802 Slice Site | 1793 |
| **CZconservative_c64733.graph_c0_331102** | c72982.graph_c0 | 255-274 Slice Site | 265 |
| **CZconservative_c64733.graph_c0_331102** | c73980.graph_c0 | 917-935 Slice Site | 926 |
| **CZconservative_c64733.graph_c0_331102** | c74948.graph_c0 | 1620-1638 Slice Site | 1629 |
| **CZconservative_c67358.graph_c0_401366** | c19919.graph_c0 | 3-27 Slice Site | 17 |
| **CZconservative_c67358.graph_c0_401366** | c19935.graph_c0 | 2-25 Slice Site | 16 |
| **CZconservative_c67358.graph_c0_401366** | c19947.graph_c0 | 4-27 Slice Site | 18 |
| **CZconservative_c67358.graph_c0_401366** | c26123.graph_c1 | 1-24 Slice Site | 15 |
| **CZconservative_c67358.graph_c0_401366** | c28889.graph_c0 | 5-28 Slice Site | 19 |
| **CZconservative_c67358.graph_c0_401366** | c32835.graph_c0 | 74-96 Slice Site | 88 |
| **CZconservative_c67358.graph_c0_401366** | c35255.graph_c0 | 28-52 Slice Site | 43 |
| **CZconservative_c67358.graph_c0_401366** | c35908.graph_c0 | 2-25 Slice Site | 16 |
| **CZconservative_c67358.graph_c0_401366** | c41254.graph_c0 | 109-132 Slice Site | 123 |
| **CZconservative_c67358.graph_c0_401366** | c41377.graph_c0 | 1973-1997 Slice Site | 1987 |
| **CZconservative_c67358.graph_c0_401366** | c44797.graph_c0 | 3-26 Slice Site | 17 |
| **CZconservative_c67358.graph_c0_401366** | c45369.graph_c0 | 4-27 Slice Site | 18 |
| **CZconservative_c67358.graph_c0_401366** | c45966.graph_c0 | 4-27 Slice Site | 18 |
| **CZconservative_c67358.graph_c0_401366** | c49455.graph_c0 | 2-24 Slice Site | 16 |
| **CZconservative_c67358.graph_c0_401366** | c49754.graph_c0 | 1-23 Slice Site | 15 |
| **CZconservative_c67358.graph_c0_401366** | c50051.graph_c0 | 2-25 Slice Site | 16 |
| **CZconservative_c67358.graph_c0_401366** | c50136.graph_c0 | 2-24 Slice Site | 16 |
| **CZconservative_c67358.graph_c0_401366** | c51496.graph_c0 | 1-24 Slice Site | 15 |
| **CZconservative_c67358.graph_c0_401366** | c53405.graph_c0 | 4-27 Slice Site | 18 |
| **CZconservative_c67358.graph_c0_401366** | c54245.graph_c0 | 3-27 Slice Site | 17 |
| **CZconservative_c67358.graph_c0_401366** | c54279.graph_c0 | 1-23 Slice Site | 15 |
| **CZconservative_c67358.graph_c0_401366** | c55205.graph_c0 | 1796-1819 Slice Site | 1810 |
| **CZconservative_c67358.graph_c0_401366** | c56201.graph_c0 | 268-291 Slice Site | 283 |
| **CZconservative_c67358.graph_c0_401366** | c57152.graph_c0 | 1852-1874 Slice Site | 1866 |
| **CZconservative_c67358.graph_c0_401366** | c57159.graph_c1 | 4-26 Slice Site | 18 |
| **CZconservative_c67358.graph_c0_401366** | c57196.graph_c0 | 2-25 Slice Site | 16 |
| **CZconservative_c67358.graph_c0_401366** | c57258.graph_c0 | 5-28 Slice Site | 20 |
| **CZconservative_c67358.graph_c0_401366** | c58783.graph_c0 | 1-24 Slice Site | 15 |
| **CZconservative_c67358.graph_c0_401366** | c59112.graph_c0 | 1-24 Slice Site | 15 |
| **CZconservative_c67358.graph_c0_401366** | c59656.graph_c2 | 1-24 Slice Site | 15 |
| **CZconservative_c67358.graph_c0_401366** | c60189.graph_c0 | 1-23 Slice Site | 15 |
| **CZconservative_c67358.graph_c0_401366** | c60607.graph_c0 | 2-26 Slice Site | 16 |
| **CZconservative_c67358.graph_c0_401366** | c61056.graph_c0 | 2-27 Slice Site | 16 |
| **CZconservative_c67358.graph_c0_401366** | c62315.graph_c0 | 2-24 Slice Site | 16 |
| **CZconservative_c67358.graph_c0_401366** | c62696.graph_c0 | 11-32 Slice Site | 24 |
| **CZconservative_c67358.graph_c0_401366** | c63434.graph_c0 | 2-24 Slice Site | 16 |
| **CZconservative_c67358.graph_c0_401366** | c64523.graph_c0 | 2-25 Slice Site | 16 |
| **CZconservative_c67358.graph_c0_401366** | c64663.graph_c0 | 2-24 Slice Site | 16 |
| **CZconservative_c67358.graph_c0_401366** | c65034.graph_c0 | 2-25 Slice Site | 16 |
| **CZconservative_c67358.graph_c0_401366** | c65144.graph_c0 | 1-24 Slice Site | 15 |
| **CZconservative_c67358.graph_c0_401366** | c65220.graph_c0 | 2-27 Slice Site | 16 |
| **CZconservative_c67358.graph_c0_401366** | c65265.graph_c0 | 57-79 Slice Site | 71 |
| **CZconservative_c67358.graph_c0_401366** | c65296.graph_c0 | 1-24 Slice Site | 15 |
| **CZconservative_c67358.graph_c0_401366** | c65449.graph_c0 | 39-62 Slice Site | 54 |
| **CZconservative_c67358.graph_c0_401366** | c65542.graph_c0 | 5-28 Slice Site | 19 |
| **CZconservative_c67358.graph_c0_401366** | c65721.graph_c0 | 2-25 Slice Site | 16 |
| **CZconservative_c67358.graph_c0_401366** | c65994.graph_c0 | 40-63 Slice Site | 54 |
| **CZconservative_c67358.graph_c0_401366** | c66155.graph_c0 | 4-27 Slice Site | 18 |
| **CZconservative_c67358.graph_c0_401366** | c66527.graph_c0 | 54-77 Slice Site | 68 |
| **CZconservative_c67358.graph_c0_401366** | c66619.graph_c1 | 1-24 Slice Site | 15 |
| **CZconservative_c67358.graph_c0_401366** | c66724.graph_c0 | 155-181 Slice Site | 171 |
| **CZconservative_c67358.graph_c0_401366** | c67721.graph_c0 | 207-230 Slice Site | 221 |
| **CZconservative_c67358.graph_c0_401366** | c68001.graph_c0 | 5-29 Slice Site | 19 |
| **CZconservative_c67358.graph_c0_401366** | c68480.graph_c1 | 95-117 Slice Site | 109 |
| **CZconservative_c67358.graph_c0_401366** | c68481.graph_c2 | 101-124 Slice Site | 115 |
| **CZconservative_c67358.graph_c0_401366** | c68518.graph_c0 | 5-30 Slice Site | 19 |
| **CZconservative_c67358.graph_c0_401366** | c69439.graph_c0 | 3-25 Slice Site | 17 |
| **CZconservative_c67358.graph_c0_401366** | c69609.graph_c0 | 4-27 Slice Site | 18 |
| **CZconservative_c67358.graph_c0_401366** | c70187.graph_c0 | 2-25 Slice Site | 16 |
| **CZconservative_c67358.graph_c0_401366** | c70367.graph_c1 | 1-23 Slice Site | 15 |
| **CZconservative_c67358.graph_c0_401366** | c70668.graph_c1 | 2-26 Slice Site | 16 |
| **CZconservative_c67358.graph_c0_401366** | c71290.graph_c0 | 134-156 Slice Site | 148 |
| **CZconservative_c67358.graph_c0_401366** | c71298.graph_c0 | 432-456 Slice Site | 447 |
| **CZconservative_c67358.graph_c0_401366** | c71814.graph_c0 | 115-137 Slice Site | 128 |
| **CZconservative_c67358.graph_c0_401366** | c71853.graph_c2 | 3-27 Slice Site | 17 |
| **CZconservative_c67358.graph_c0_401366** | c73260.graph_c0 | 1749-1772 Slice Site | 1763 |
| **CZconservative_c67358.graph_c0_401366** | c73412.graph_c0 | 2-26 Slice Site | 16 |
| **CZconservative_c67358.graph_c0_401366** | c74422.graph_c1 | 4-26 Slice Site | 18 |
| **CZconservative_c67358.graph_c0_401366** | c75242.graph_c0 | 5-28 Slice Site | 19 |
| **CZconservative_c67358.graph_c0_401366** | c76532.graph_c0 | 3-26 Slice Site | 17 |
| **CZconservative_c67358.graph_c0_401366** | c82372.graph_c0 | 2-24 Slice Site | 16 |
| **CZconservative_c68034.graph_c0_423214** | c45599.graph_c0 | 440-461 Slice Site | 452 |
| **CZconservative_c68034.graph_c0_423214** | c70125.graph_c1 | 621-643 Slice Site | 634 |
| **CZconservative_c69137.graph_c0_464447** | c51883.graph_c0 | 410-427 Slice Site | 418 |
| **CZconservative_c69137.graph_c0_464447** | c68058.graph_c0 | 227-243 Slice Site | 235 |
| **CZconservative_c69137.graph_c0_464447** | c69076.graph_c1 | 413-429 Slice Site | 420 |
| **CZconservative_c69137.graph_c0_464447** | c71576.graph_c0 | 1126-1143 Slice Site | 1134 |
| **CZconservative_c69137.graph_c0_464447** | c72576.graph_c0 | 292-308 Slice Site | 300 |
| **CZconservative_c69137.graph_c0_464447** | c72790.graph_c0 | 196-212 Slice Site | 204 |
| **CZconservative_c69137.graph_c0_464447** | c72804.graph_c0 | 2695-2712 Slice Site | 2703 |
| **CZconservative_c69137.graph_c0_464447** | c75071.graph_c0 | 1086-1104 Slice Site | 1095 |
| **CZconservative_c69137.graph_c0_464447** | c75377.graph_c0 | 3553-3570 Slice Site | 3561 |
| **CZconservative_c69137.graph_c0_464447** | c75775.graph_c0 | 296-313 Slice Site | 304 |
| **CZconservative_c69137.graph_c0_464447** | c76615.graph_c0 | 270-285 Slice Site | 278 |
| **CZconservative_c70108.graph_c0_503430** | c41682.graph_c0 | 164-180 Slice Site | 171 |
| **CZconservative_c70108.graph_c0_503430** | c42930.graph_c0 | 383-401 Slice Site | 392 |
| **CZconservative_c70108.graph_c0_503430** | c70242.graph_c0 | 470-486 Slice Site | 478 |
| **CZconservative_c70108.graph_c0_503430** | c71046.graph_c0 | 2692-2708 Slice Site | 2699 |
| **CZconservative_c70108.graph_c0_503430** | c71878.graph_c0 | 787-805 Slice Site | 796 |
| **CZconservative_c70883.graph_c0_536677** | c34313.graph_c0 | 5-24 Slice Site | 15 |
| **CZconservative_c70883.graph_c0_536677** | c44500.graph_c0 | 1-20 Slice Site | 11 |
| **CZconservative_c70883.graph_c0_536677** | c62873.graph_c0 | 2083-2102 Slice Site | 2093 |
| **CZconservative_c70883.graph_c0_536677** | c72327.graph_c0 | 1878-1897 Slice Site | 1888 |
| **CZconservative_c71433.graph_c0_562517** | c52312.graph_c1 | 244-260 Slice Site | 253 |
| **CZconservative_c71433.graph_c0_562517** | c69053.graph_c0 | 533-552 Slice Site | 543 |
| **CZconservative_c71433.graph_c0_562517** | c74917.graph_c0 | 725-745 Slice Site | 735 |
| **CZconservative_c71510.graph_c2_566150** | c43323.graph_c0 | 247-268 Slice Site | 259 |
| **CZconservative_c71510.graph_c2_566150** | c47108.graph_c0 | 409-430 Slice Site | 421 |
| **CZconservative_c71510.graph_c2_566150** | c57312.graph_c0 | 48-68 Slice Site | 59 |
| **CZconservative_c71510.graph_c2_566150** | c65576.graph_c0 | 193-212 Slice Site | 203 |
| **CZconservative_c71510.graph_c2_566150** | c73534.graph_c0 | 706-726 Slice Site | 717 |
| **CZconservative_c71510.graph_c2_566150** | c74750.graph_c1 | 2287-2306 Slice Site | 2297 |
| **CZconservative_c79373.graph_c0_778990** | c74659.graph_c1 | 56-76 Slice Site | 67 |
| **CZconservative_c82842.graph_c0_793718** | c19916.graph_c0 | 219-234 Slice Site | 225 |
| **CZconservative_c82842.graph_c0_793718** | c40973.graph_c0 | 179-196 Slice Site | 187 |
| **CZconservative_c82842.graph_c0_793718** | c44759.graph_c0 | 203-220 Slice Site | 211 |
| **CZconservative_c82842.graph_c0_793718** | c57266.graph_c0 | 70-87 Slice Site | 78 |
| **CZconservative_c82842.graph_c0_793718** | c62523.graph_c0 | 150-166 Slice Site | 157 |
| **CZconservative_c82842.graph_c0_793718** | c65725.graph_c0 | 217-232 Slice Site | 223 |
| **CZconservative_c82842.graph_c0_793718** | c67047.graph_c0 | 2620-2637 Slice Site | 2628 |
| **CZconservative_c82842.graph_c0_793718** | c67668.graph_c0 | 2296-2313 Slice Site | 2304 |
| **CZconservative_c82842.graph_c0_793718** | c67894.graph_c0 | 4109-4126 Slice Site | 4117 |
| **CZconservative_c82842.graph_c0_793718** | c68204.graph_c0 | 1458-1475 Slice Site | 1466 |
| **CZconservative_c82842.graph_c0_793718** | c68749.graph_c0 | 1331-1348 Slice Site | 1339 |
| **CZconservative_c82842.graph_c0_793718** | c69894.graph_c0 | 852-868 Slice Site | 860 |
| **CZconservative_c82842.graph_c0_793718** | c70067.graph_c0 | 1913-1929 Slice Site | 1921 |
| **CZconservative_c82842.graph_c0_793718** | c70582.graph_c0 | 477-494 Slice Site | 485 |
| **CZconservative_c82842.graph_c0_793718** | c71002.graph_c0 | 2008-2025 Slice Site | 2016 |
| **CZconservative_c82842.graph_c0_793718** | c71061.graph_c0 | 1579-1596 Slice Site | 1587 |
| **CZconservative_c82842.graph_c0_793718** | c71118.graph_c0 | 243-259 Slice Site | 250 |
| **CZconservative_c82842.graph_c0_793718** | c71237.graph_c0 | 504-520 Slice Site | 512 |
| **CZconservative_c82842.graph_c0_793718** | c73216.graph_c0 | 351-368 Slice Site | 360 |
| **CZconservative_c82842.graph_c0_793718** | c73779.graph_c1 | 1536-1553 Slice Site | 1544 |
| **CZconservative_c82842.graph_c0_793718** | c74518.graph_c0 | 1933-1950 Slice Site | 1941 |
| **CZconservative_c82842.graph_c0_793718** | c75547.graph_c0 | 1004-1021 Slice Site | 1012 |
| **CZconservative_c82842.graph_c0_793718** | c75683.graph_c4 | 856-874 Slice Site | 864 |
| **CZconservative_c84589.graph_c0_800317** | c72450.graph_c0 | 2712-2731 Slice Site | 2723 |
| **CZconservative_c84589.graph_c0_800317** | c75592.graph_c0 | 887-907 Slice Site | 898 |
| **CZconservative_c85394.graph_c0_803159** | c103546.graph_c0 | 72-88 Slice Site | 80 |
| **CZconservative_c85394.graph_c0_803159** | c106516.graph_c0 | 182-200 Slice Site | 191 |
| **CZconservative_c85394.graph_c0_803159** | c112338.graph_c0 | 81-100 Slice Site | 91 |
| **CZconservative_c85394.graph_c0_803159** | c114418.graph_c0 | 38-57 Slice Site | 47 |
| **CZconservative_c85394.graph_c0_803159** | c18414.graph_c0 | 338-356 Slice Site | 347 |
| **CZconservative_c85394.graph_c0_803159** | c18576.graph_c0 | 146-164 Slice Site | 155 |
| **CZconservative_c85394.graph_c0_803159** | c19690.graph_c0 | 342-360 Slice Site | 351 |
| **CZconservative_c85394.graph_c0_803159** | c19711.graph_c0 | 1-19 Slice Site | 10 |
| **CZconservative_c85394.graph_c0_803159** | c19825.graph_c0 | 279-298 Slice Site | 289 |
| **CZconservative_c85394.graph_c0_803159** | c19855.graph_c1 | 52-70 Slice Site | 61 |
| **CZconservative_c85394.graph_c0_803159** | c19911.graph_c0 | 176-193 Slice Site | 184 |
| **CZconservative_c85394.graph_c0_803159** | c19972.graph_c0 | 46-64 Slice Site | 55 |
| **CZconservative_c85394.graph_c0_803159** | c20004.graph_c0 | 475-493 Slice Site | 484 |
| **CZconservative_c85394.graph_c0_803159** | c20018.graph_c0 | 166-188 Slice Site | 179 |
| **CZconservative_c85394.graph_c0_803159** | c20859.graph_c0 | 78-96 Slice Site | 87 |
| **CZconservative_c85394.graph_c0_803159** | c24701.graph_c0 | 133-151 Slice Site | 142 |
| **CZconservative_c85394.graph_c0_803159** | c26123.graph_c1 | 1-19 Slice Site | 10 |
| **CZconservative_c85394.graph_c0_803159** | c27847.graph_c0 | 89-108 Slice Site | 98 |
| **CZconservative_c85394.graph_c0_803159** | c28167.graph_c0 | 1-19 Slice Site | 10 |
| **CZconservative_c85394.graph_c0_803159** | c29491.graph_c0 | 1-19 Slice Site | 10 |
| **CZconservative_c85394.graph_c0_803159** | c29616.graph_c0 | 142-162 Slice Site | 153 |
| **CZconservative_c85394.graph_c0_803159** | c29904.graph_c0 | 160-178 Slice Site | 169 |
| **CZconservative_c85394.graph_c0_803159** | c30940.graph_c0 | 282-300 Slice Site | 291 |
| **CZconservative_c85394.graph_c0_803159** | c34679.graph_c0 | 167-184 Slice Site | 175 |
| **CZconservative_c85394.graph_c0_803159** | c36931.graph_c0 | 33-50 Slice Site | 41 |
| **CZconservative_c85394.graph_c0_803159** | c37233.graph_c0 | 12-30 Slice Site | 21 |
| **CZconservative_c85394.graph_c0_803159** | c37736.graph_c0 | 478-496 Slice Site | 487 |
| **CZconservative_c85394.graph_c0_803159** | c40830.graph_c0 | 147-165 Slice Site | 156 |
| **CZconservative_c85394.graph_c0_803159** | c41960.graph_c0 | 17-35 Slice Site | 26 |
| **CZconservative_c85394.graph_c0_803159** | c42606.graph_c0 | 46-64 Slice Site | 55 |
| **CZconservative_c85394.graph_c0_803159** | c43582.graph_c0 | 93-111 Slice Site | 102 |
| **CZconservative_c85394.graph_c0_803159** | c44302.graph_c0 | 40-58 Slice Site | 49 |
| **CZconservative_c85394.graph_c0_803159** | c44601.graph_c0 | 493-510 Slice Site | 502 |
| **CZconservative_c85394.graph_c0_803159** | c44797.graph_c0 | 1-19 Slice Site | 10 |
| **CZconservative_c85394.graph_c0_803159** | c44830.graph_c0 | 1-19 Slice Site | 10 |
| **CZconservative_c85394.graph_c0_803159** | c45136.graph_c0 | 1-19 Slice Site | 10 |
| **CZconservative_c85394.graph_c0_803159** | c46207.graph_c0 | 83-100 Slice Site | 91 |
| **CZconservative_c85394.graph_c0_803159** | c46843.graph_c0 | 6-24 Slice Site | 15 |
| **CZconservative_c85394.graph_c0_803159** | c46856.graph_c0 | 1-19 Slice Site | 10 |
| **CZconservative_c85394.graph_c0_803159** | c47342.graph_c0 | 15-32 Slice Site | 24 |
| **CZconservative_c85394.graph_c0_803159** | c47902.graph_c1 | 131-149 Slice Site | 140 |
| **CZconservative_c85394.graph_c0_803159** | c48656.graph_c0 | 1-19 Slice Site | 10 |
| **CZconservative_c85394.graph_c0_803159** | c49480.graph_c0 | 28-46 Slice Site | 37 |
| **CZconservative_c85394.graph_c0_803159** | c50898.graph_c0 | 39-57 Slice Site | 48 |
| **CZconservative_c85394.graph_c0_803159** | c51546.graph_c0 | 65-83 Slice Site | 74 |
| **CZconservative_c85394.graph_c0_803159** | c52173.graph_c0 | 934-952 Slice Site | 943 |
| **CZconservative_c85394.graph_c0_803159** | c53041.graph_c0 | 1-19 Slice Site | 10 |
| **CZconservative_c85394.graph_c0_803159** | c53255.graph_c0 | 927-946 Slice Site | 937 |
| **CZconservative_c85394.graph_c0_803159** | c53779.graph_c0 | 114-135 Slice Site | 126 |
| **CZconservative_c85394.graph_c0_803159** | c53983.graph_c0 | 188-207 Slice Site | 198 |
| **CZconservative_c85394.graph_c0_803159** | c54243.graph_c0 | 159-176 Slice Site | 168 |
| **CZconservative_c85394.graph_c0_803159** | c54279.graph_c0 | 1-19 Slice Site | 10 |
| **CZconservative_c85394.graph_c0_803159** | c54528.graph_c0 | 466-483 Slice Site | 474 |
| **CZconservative_c85394.graph_c0_803159** | c54661.graph_c0 | 463-480 Slice Site | 471 |
| **CZconservative_c85394.graph_c0_803159** | c55186.graph_c0 | 72-90 Slice Site | 82 |
| **CZconservative_c85394.graph_c0_803159** | c55205.graph_c0 | 1796-1814 Slice Site | 1805 |
| **CZconservative_c85394.graph_c0_803159** | c55551.graph_c0 | 202-218 Slice Site | 210 |
| **CZconservative_c85394.graph_c0_803159** | c55881.graph_c0 | 219-237 Slice Site | 228 |
| **CZconservative_c85394.graph_c0_803159** | c55937.graph_c0 | 155-173 Slice Site | 164 |
| **CZconservative_c85394.graph_c0_803159** | c56125.graph_c0 | 261-279 Slice Site | 270 |
| **CZconservative_c85394.graph_c0_803159** | c56249.graph_c0 | 551-569 Slice Site | 560 |
| **CZconservative_c85394.graph_c0_803159** | c56583.graph_c0 | 151-170 Slice Site | 160 |
| **CZconservative_c85394.graph_c0_803159** | c56909.graph_c0 | 1-19 Slice Site | 10 |
| **CZconservative_c85394.graph_c0_803159** | c57130.graph_c0 | 595-615 Slice Site | 606 |
| **CZconservative_c85394.graph_c0_803159** | c57138.graph_c0 | 189-208 Slice Site | 199 |
| **CZconservative_c85394.graph_c0_803159** | c57150.graph_c0 | 93-111 Slice Site | 102 |
| **CZconservative_c85394.graph_c0_803159** | c57232.graph_c0 | 285-303 Slice Site | 294 |
| **CZconservative_c85394.graph_c0_803159** | c57258.graph_c0 | 1-19 Slice Site | 10 |
| **CZconservative_c85394.graph_c0_803159** | c57488.graph_c0 | 1-19 Slice Site | 10 |
| **CZconservative_c85394.graph_c0_803159** | c59123.graph_c0 | 396-415 Slice Site | 405 |
| **CZconservative_c85394.graph_c0_803159** | c59228.graph_c0 | 2292-2309 Slice Site | 2300 |
| **CZconservative_c85394.graph_c0_803159** | c59569.graph_c0 | 1-19 Slice Site | 10 |
| **CZconservative_c85394.graph_c0_803159** | c59643.graph_c0 | 1-19 Slice Site | 10 |
| **CZconservative_c85394.graph_c0_803159** | c59656.graph_c2 | 1-19 Slice Site | 10 |
| **CZconservative_c85394.graph_c0_803159** | c59964.graph_c0 | 1209-1227 Slice Site | 1218 |
| **CZconservative_c85394.graph_c0_803159** | c60075.graph_c1 | 28-46 Slice Site | 38 |
| **CZconservative_c85394.graph_c0_803159** | c60363.graph_c0 | 56-75 Slice Site | 66 |
| **CZconservative_c85394.graph_c0_803159** | c60515.graph_c0 | 713-731 Slice Site | 722 |
| **CZconservative_c85394.graph_c0_803159** | c60522.graph_c0 | 375-393 Slice Site | 384 |
| **CZconservative_c85394.graph_c0_803159** | c60874.graph_c0 | 798-816 Slice Site | 807 |
| **CZconservative_c85394.graph_c0_803159** | c61194.graph_c0 | 182-200 Slice Site | 191 |
| **CZconservative_c85394.graph_c0_803159** | c61544.graph_c0 | 44-62 Slice Site | 53 |
| **CZconservative_c85394.graph_c0_803159** | c61876.graph_c0 | 447-466 Slice Site | 457 |
| **CZconservative_c85394.graph_c0_803159** | c61945.graph_c0 | 131-151 Slice Site | 142 |
| **CZconservative_c85394.graph_c0_803159** | c61975.graph_c0 | 335-352 Slice Site | 343 |
| **CZconservative_c85394.graph_c0_803159** | c62373.graph_c1 | 109-127 Slice Site | 118 |
| **CZconservative_c85394.graph_c0_803159** | c62395.graph_c0 | 196-214 Slice Site | 205 |
| **CZconservative_c85394.graph_c0_803159** | c62494.graph_c0 | 80-98 Slice Site | 89 |
| **CZconservative_c85394.graph_c0_803159** | c62586.graph_c0 | 1-19 Slice Site | 10 |
| **CZconservative_c85394.graph_c0_803159** | c62661.graph_c0 | 1-19 Slice Site | 10 |
| **CZconservative_c85394.graph_c0_803159** | c63007.graph_c0 | 25-44 Slice Site | 35 |
| **CZconservative_c85394.graph_c0_803159** | c63751.graph_c0 | 33-52 Slice Site | 43 |
| **CZconservative_c85394.graph_c0_803159** | c63787.graph_c0 | 1-19 Slice Site | 10 |
| **CZconservative_c85394.graph_c0_803159** | c63827.graph_c0 | 1-19 Slice Site | 10 |
| **CZconservative_c85394.graph_c0_803159** | c64016.graph_c0 | 1-19 Slice Site | 10 |
| **CZconservative_c85394.graph_c0_803159** | c64161.graph_c0 | 10-29 Slice Site | 19 |
| **CZconservative_c85394.graph_c0_803159** | c64348.graph_c0 | 2406-2424 Slice Site | 2415 |
| **CZconservative_c85394.graph_c0_803159** | c64540.graph_c0 | 1112-1129 Slice Site | 1121 |
| **CZconservative_c85394.graph_c0_803159** | c64908.graph_c1 | 172-190 Slice Site | 181 |
| **CZconservative_c85394.graph_c0_803159** | c65361.graph_c0 | 160-178 Slice Site | 169 |
| **CZconservative_c85394.graph_c0_803159** | c65503.graph_c0 | 381-399 Slice Site | 390 |
| **CZconservative_c85394.graph_c0_803159** | c65982.graph_c0 | 9-28 Slice Site | 19 |
| **CZconservative_c85394.graph_c0_803159** | c66116.graph_c0 | 366-384 Slice Site | 375 |
| **CZconservative_c85394.graph_c0_803159** | c66141.graph_c0 | 414-431 Slice Site | 422 |
| **CZconservative_c85394.graph_c0_803159** | c66222.graph_c0 | 4457-4475 Slice Site | 4466 |
| **CZconservative_c85394.graph_c0_803159** | c66237.graph_c0 | 1178-1196 Slice Site | 1187 |
| **CZconservative_c85394.graph_c0_803159** | c66511.graph_c0 | 1105-1123 Slice Site | 1114 |
| **CZconservative_c85394.graph_c0_803159** | c66546.graph_c0 | 1-19 Slice Site | 10 |
| **CZconservative_c85394.graph_c0_803159** | c66610.graph_c0 | 75-95 Slice Site | 85 |
| **CZconservative_c85394.graph_c0_803159** | c66717.graph_c0 | 4-22 Slice Site | 13 |
| **CZconservative_c85394.graph_c0_803159** | c66724.graph_c0 | 143-163 Slice Site | 152 |
| **CZconservative_c85394.graph_c0_803159** | c66794.graph_c0 | 1-19 Slice Site | 10 |
| **CZconservative_c85394.graph_c0_803159** | c66898.graph_c0 | 1-19 Slice Site | 10 |
| **CZconservative_c85394.graph_c0_803159** | c66985.graph_c0 | 47-65 Slice Site | 56 |
| **CZconservative_c85394.graph_c0_803159** | c67041.graph_c0 | 2090-2108 Slice Site | 2099 |
| **CZconservative_c85394.graph_c0_803159** | c67100.graph_c0 | 40-58 Slice Site | 49 |
| **CZconservative_c85394.graph_c0_803159** | c67226.graph_c0 | 289-306 Slice Site | 297 |
| **CZconservative_c85394.graph_c0_803159** | c67721.graph_c0 | 203-221 Slice Site | 212 |
| **CZconservative_c85394.graph_c0_803159** | c67958.graph_c0 | 552-570 Slice Site | 561 |
| **CZconservative_c85394.graph_c0_803159** | c68024.graph_c0 | 9-26 Slice Site | 18 |
| **CZconservative_c85394.graph_c0_803159** | c68355.graph_c0 | 2-20 Slice Site | 11 |
| **CZconservative_c85394.graph_c0_803159** | c68391.graph_c1 | 60-78 Slice Site | 69 |
| **CZconservative_c85394.graph_c0_803159** | c68480.graph_c1 | 89-107 Slice Site | 98 |
| **CZconservative_c85394.graph_c0_803159** | c68715.graph_c0 | 1144-1162 Slice Site | 1153 |
| **CZconservative_c85394.graph_c0_803159** | c68715.graph_c0 | 1189-1207 Slice Site | 1198 |
| **CZconservative_c85394.graph_c0_803159** | c68725.graph_c0 | 1089-1106 Slice Site | 1098 |
| **CZconservative_c85394.graph_c0_803159** | c68807.graph_c0 | 1939-1956 Slice Site | 1948 |
| **CZconservative_c85394.graph_c0_803159** | c69074.graph_c0 | 1930-1948 Slice Site | 1939 |
| **CZconservative_c85394.graph_c0_803159** | c69357.graph_c0 | 1-19 Slice Site | 10 |
| **CZconservative_c85394.graph_c0_803159** | c69439.graph_c0 | 1-19 Slice Site | 10 |
| **CZconservative_c85394.graph_c0_803159** | c69542.graph_c0 | 2741-2758 Slice Site | 2750 |
| **CZconservative_c85394.graph_c0_803159** | c69586.graph_c0 | 41-59 Slice Site | 50 |
| **CZconservative_c85394.graph_c0_803159** | c69687.graph_c0 | 1-19 Slice Site | 10 |
| **CZconservative_c85394.graph_c0_803159** | c69721.graph_c0 | 1734-1754 Slice Site | 1744 |
| **CZconservative_c85394.graph_c0_803159** | c69763.graph_c0 | 1302-1320 Slice Site | 1311 |
| **CZconservative_c85394.graph_c0_803159** | c69782.graph_c0 | 1-19 Slice Site | 10 |
| **CZconservative_c85394.graph_c0_803159** | c69782.graph_c0 | 2565-2583 Slice Site | 2574 |
| **CZconservative_c85394.graph_c0_803159** | c69918.graph_c0 | 142-161 Slice Site | 152 |
| **CZconservative_c85394.graph_c0_803159** | c70016.graph_c0 | 531-549 Slice Site | 540 |
| **CZconservative_c85394.graph_c0_803159** | c70126.graph_c0 | 87-105 Slice Site | 96 |
| **CZconservative_c85394.graph_c0_803159** | c70264.graph_c0 | 1-19 Slice Site | 10 |
| **CZconservative_c85394.graph_c0_803159** | c70367.graph_c1 | 1-19 Slice Site | 10 |
| **CZconservative_c85394.graph_c0_803159** | c70767.graph_c0 | 1065-1084 Slice Site | 1075 |
| **CZconservative_c85394.graph_c0_803159** | c70780.graph_c0 | 170-189 Slice Site | 179 |
| **CZconservative_c85394.graph_c0_803159** | c70809.graph_c0 | 474-492 Slice Site | 483 |
| **CZconservative_c85394.graph_c0_803159** | c70911.graph_c0 | 3016-3034 Slice Site | 3025 |
| **CZconservative_c85394.graph_c0_803159** | c71112.graph_c0 | 991-1010 Slice Site | 1001 |
| **CZconservative_c85394.graph_c0_803159** | c71549.graph_c0 | 1-19 Slice Site | 10 |
| **CZconservative_c85394.graph_c0_803159** | c71778.graph_c1 | 457-475 Slice Site | 466 |
| **CZconservative_c85394.graph_c0_803159** | c72095.graph_c1 | 36-58 Slice Site | 49 |
| **CZconservative_c85394.graph_c0_803159** | c72105.graph_c0 | 180-199 Slice Site | 190 |
| **CZconservative_c85394.graph_c0_803159** | c72260.graph_c0 | 101-119 Slice Site | 110 |
| **CZconservative_c85394.graph_c0_803159** | c72281.graph_c0 | 2001-2019 Slice Site | 2009 |
| **CZconservative_c85394.graph_c0_803159** | c72281.graph_c0 | 2804-2822 Slice Site | 2813 |
| **CZconservative_c85394.graph_c0_803159** | c72374.graph_c0 | 25-44 Slice Site | 35 |
| **CZconservative_c85394.graph_c0_803159** | c72387.graph_c3 | 57-75 Slice Site | 66 |
| **CZconservative_c85394.graph_c0_803159** | c72537.graph_c0 | 3547-3564 Slice Site | 3555 |
| **CZconservative_c85394.graph_c0_803159** | c72576.graph_c2 | 1-19 Slice Site | 10 |
| **CZconservative_c85394.graph_c0_803159** | c72693.graph_c0 | 1548-1566 Slice Site | 1557 |
| **CZconservative_c85394.graph_c0_803159** | c72721.graph_c0 | 626-644 Slice Site | 635 |
| **CZconservative_c85394.graph_c0_803159** | c72989.graph_c0 | 1-19 Slice Site | 10 |
| **CZconservative_c85394.graph_c0_803159** | c72995.graph_c0 | 242-262 Slice Site | 253 |
| **CZconservative_c85394.graph_c0_803159** | c73009.graph_c0 | 1269-1288 Slice Site | 1279 |
| **CZconservative_c85394.graph_c0_803159** | c73048.graph_c2 | 1437-1455 Slice Site | 1446 |
| **CZconservative_c85394.graph_c0_803159** | c73080.graph_c0 | 155-173 Slice Site | 164 |
| **CZconservative_c85394.graph_c0_803159** | c73514.graph_c0 | 2361-2379 Slice Site | 2370 |
| **CZconservative_c85394.graph_c0_803159** | c73698.graph_c0 | 144-162 Slice Site | 153 |
| **CZconservative_c85394.graph_c0_803159** | c73760.graph_c0 | 1-19 Slice Site | 10 |
| **CZconservative_c85394.graph_c0_803159** | c73769.graph_c1 | 2273-2291 Slice Site | 2282 |
| **CZconservative_c85394.graph_c0_803159** | c74091.graph_c1 | 1-19 Slice Site | 10 |
| **CZconservative_c85394.graph_c0_803159** | c74096.graph_c0 | 874-892 Slice Site | 883 |
| **CZconservative_c85394.graph_c0_803159** | c74142.graph_c0 | 1351-1369 Slice Site | 1359 |
| **CZconservative_c85394.graph_c0_803159** | c74328.graph_c1 | 1608-1626 Slice Site | 1617 |
| **CZconservative_c85394.graph_c0_803159** | c74554.graph_c1 | 2259-2277 Slice Site | 2268 |
| **CZconservative_c85394.graph_c0_803159** | c74568.graph_c0 | 6226-6245 Slice Site | 6236 |
| **CZconservative_c85394.graph_c0_803159** | c74634.graph_c1 | 566-586 Slice Site | 577 |
| **CZconservative_c85394.graph_c0_803159** | c74731.graph_c0 | 79-97 Slice Site | 88 |
| **CZconservative_c85394.graph_c0_803159** | c74868.graph_c0 | 157-175 Slice Site | 166 |
| **CZconservative_c85394.graph_c0_803159** | c74883.graph_c1 | 1304-1321 Slice Site | 1313 |
| **CZconservative_c85394.graph_c0_803159** | c74989.graph_c1 | 36-55 Slice Site | 45 |
| **CZconservative_c85394.graph_c0_803159** | c75231.graph_c0 | 1541-1559 Slice Site | 1550 |
| **CZconservative_c85394.graph_c0_803159** | c75277.graph_c0 | 21-39 Slice Site | 30 |
| **CZconservative_c85394.graph_c0_803159** | c75393.graph_c0 | 241-259 Slice Site | 250 |
| **CZconservative_c85394.graph_c0_803159** | c75586.graph_c0 | 315-332 Slice Site | 324 |
| **CZconservative_c85394.graph_c0_803159** | c75749.graph_c0 | 60-77 Slice Site | 68 |
| **CZconservative_c85394.graph_c0_803159** | c76005.graph_c2 | 2053-2071 Slice Site | 2062 |
| **CZconservative_c85394.graph_c0_803159** | c76027.graph_c0 | 832-850 Slice Site | 841 |
| **CZconservative_c85394.graph_c0_803159** | c76150.graph_c0 | 165-184 Slice Site | 175 |
| **CZconservative_c85394.graph_c0_803159** | c76175.graph_c0 | 2-20 Slice Site | 11 |
| **CZconservative_c85394.graph_c0_803159** | c76532.graph_c0 | 1-19 Slice Site | 10 |
| **CZconservative_c85394.graph_c0_803159** | c76608.graph_c0 | 52-70 Slice Site | 61 |
| **CZconservative_c85394.graph_c0_803159** | c77035.graph_c0 | 1-19 Slice Site | 10 |
| **CZconservative_c85394.graph_c0_803159** | c77320.graph_c0 | 251-268 Slice Site | 260 |
| **CZconservative_c85394.graph_c0_803159** | c79596.graph_c0 | 42-60 Slice Site | 51 |
| **CZconservative_c85394.graph_c0_803159** | c96668.graph_c0 | 139-158 Slice Site | 149 |
| **CZPconservative_c1475.graph_c0_173** | c30890.graph_c0 | 61-81 Slice Site | 73 |
| **CZPconservative_c1475.graph_c0_173** | c37341.graph_c0 | 3-23 Slice Site | 14 |
| **CZPconservative_c1475.graph_c0_173** | c45053.graph_c1 | 112-132 Slice Site | 123 |
| **CZPconservative_c1475.graph_c0_173** | c48068.graph_c0 | 216-235 Slice Site | 227 |
| **CZPconservative_c1475.graph_c0_173** | c49635.graph_c0 | 1-21 Slice Site | 12 |
| **CZPconservative_c1475.graph_c0_173** | c52463.graph_c0 | 88-108 Slice Site | 99 |
| **CZPconservative_c1475.graph_c0_173** | c53828.graph_c0 | 991-1011 Slice Site | 1002 |
| **CZPconservative_c1475.graph_c0_173** | c55235.graph_c0 | 3-23 Slice Site | 14 |
| **CZPconservative_c1475.graph_c0_173** | c62000.graph_c0 | 710-729 Slice Site | 721 |
| **CZPconservative_c1475.graph_c0_173** | c63358.graph_c0 | 1152-1171 Slice Site | 1163 |
| **CZPconservative_c1475.graph_c0_173** | c67990.graph_c0 | 2337-2356 Slice Site | 2348 |
| **CZPconservative_c1475.graph_c0_173** | c71613.graph_c0 | 768-788 Slice Site | 779 |
| **CZPconservative_c1475.graph_c0_173** | c71640.graph_c0 | 1711-1731 Slice Site | 1722 |
| **CZPconservative_c1475.graph_c0_173** | c75813.graph_c1 | 545-565 Slice Site | 556 |
| **CZPconservative_c50750.graph_c0_9083** | c26462.graph_c0 | 193-212 Slice Site | 203 |
| **CZPconservative_c50750.graph_c0_9083** | c53015.graph_c0 | 359-378 Slice Site | 369 |
| **CZPconservative_c50750.graph_c0_9083** | c57599.graph_c0 | 1125-1144 Slice Site | 1135 |
| **CZPconservative_c50750.graph_c0_9083** | c64342.graph_c0 | 169-188 Slice Site | 179 |
| **CZPconservative_c50750.graph_c0_9083** | c67425.graph_c0 | 1042-1059 Slice Site | 1050 |
| **CZPconservative_c50750.graph_c0_9083** | c67928.graph_c0 | 187-206 Slice Site | 197 |
| **CZPconservative_c50750.graph_c0_9083** | c68464.graph_c0 | 212-229 Slice Site | 221 |
| **CZPconservative_c50750.graph_c0_9083** | c70693.graph_c2 | 143-161 Slice Site | 152 |
| **CZPconservative_c50750.graph_c0_9083** | c72782.graph_c0 | 750-768 Slice Site | 759 |
| **CZPconservative_c50750.graph_c0_9083** | c74349.graph_c1 | 5-25 Slice Site | 16 |
| **CZPconservative_c50750.graph_c0_9083** | c75709.graph_c0 | 3167-3187 Slice Site | 3178 |
| **CZPconservative_c52325.graph_c0_9764** | c19631.graph_c0 | 450-470 Slice Site | 461 |
| **CZPconservative_c52325.graph_c0_9764** | c65477.graph_c0 | 674-697 Slice Site | 688 |
| **CZPconservative_c62025.graph_c0_16894** | c22166.graph_c0 | 1564-1582 Slice Site | 1573 |
| **CZPconservative_c62025.graph_c0_16894** | c43479.graph_c1 | 1011-1029 Slice Site | 1020 |
| **CZPconservative_c62025.graph_c0_16894** | c59557.graph_c0 | 296-313 Slice Site | 304 |
| **CZPconservative_c62025.graph_c0_16894** | c63440.graph_c0 | 529-547 Slice Site | 538 |
| **CZPconservative_c62025.graph_c0_16894** | c67011.graph_c0 | 852-872 Slice Site | 863 |
| **CZPconservative_c62025.graph_c0_16894** | c67790.graph_c0 | 236-255 Slice Site | 246 |
| **CZPconservative_c62025.graph_c0_16894** | c71446.graph_c0 | 141-160 Slice Site | 151 |
| **CZPconservative_c62025.graph_c0_16894** | c78105.graph_c0 | 377-395 Slice Site | 386 |
| **CZPconservative_c63965.graph_c0_19028** | c72782.graph_c0 | 2655-2669 Slice Site | 2660 |
| **CZPconservative_c79576.graph_c0_44229** | c20209.graph_c0 | 125-145 Slice Site | 136 |
| **CZPconservative_c79576.graph_c0_44229** | c36109.graph_c0 | 417-437 Slice Site | 428 |
| **CZPconservative_c79576.graph_c0_44229** | c68871.graph_c0 | 2417-2437 Slice Site | 2428 |
| **CZPconservative_c79576.graph_c0_44229** | c69110.graph_c0 | 848-868 Slice Site | 859 |
| **CZPconservative_c79576.graph_c0_44229** | **c71524.graph_c0** | 1535-1555 Slice Site | 1546 |
| **CZPconservative_c79576.graph_c0_44229** | c72823.graph_c0 | 3969-3989 Slice Site | 3980 |
| **CZPconservative_c79576.graph_c0_44229** | c73958.graph_c0 | 804-824 Slice Site | 815 |
| **CZPconservative_c79576.graph_c0_44229** | c74863.graph_c0 | 995-1015 Slice Site | 1006 |
| **CZPconservative_c79576.graph_c0_44229** | c78475.graph_c0 | 263-283 Slice Site | 274 |
| **CZPunconservative_c52117.graph_c0_9677** | c68292.graph_c0 | 1040-1059 Slice Site | 1051 |
| **CZPunconservative_c52117.graph_c0_9677** | c68498.graph_c0 | 1209-1228 Slice Site | 1219 |
| **CZPunconservative_c52117.graph_c0_9677** | c74925.graph_c0 | 2984-3004 Slice Site | 2995 |
| **CZPunconservative_c55465.graph_c2_11696** | c56117.graph_c0 | 425-450 Slice Site | 440 |
| **CZPunconservative_c55465.graph_c2_11696** | c63302.graph_c0 | 26-49 Slice Site | 40 |
| **CZPunconservative_c55465.graph_c2_11696** | c67278.graph_c0 | 457-482 Slice Site | 473 |
| **CZunconservative_c30017.graph_c0_28548** | c73430.graph_c0 | 3021-3044 Slice Site | 3036 |
| **CZunconservative_c30017.graph_c0_28548** | c76040.graph_c0 | 4553-4576 Slice Site | 4567 |
| **CZunconservative_c30704.graph_c0_32842** | c48146.graph_c0 | 881-899 Slice Site | 890 |
| **CZunconservative_c30704.graph_c0_32842** | c55032.graph_c0 | 460-481 Slice Site | 472 |
| **CZunconservative_c30704.graph_c0_32842** | c72248.graph_c0 | 2456-2477 Slice Site | 2468 |
| **CZunconservative_c30704.graph_c0_32842** | c77056.graph_c0 | 32-50 Slice Site | 41 |
| **CZunconservative_c35851.graph_c0_70554** | c45380.graph_c0 | 1169-1192 Slice Site | 1183 |
| **CZunconservative_c35851.graph_c0_70554** | c71436.graph_c0 | 1359-1381 Slice Site | 1372 |
| **CZunconservative_c59645.graph_c0_235593** | c35660.graph_c0 | 338-355 Slice Site | 347 |
| **CZunconservative_c59645.graph_c0_235593** | c63135.graph_c0 | 448-465 Slice Site | 456 |
| **CZunconservative_c59645.graph_c0_235593** | c63935.graph_c0 | 267-285 Slice Site | 275 |
| **CZunconservative_c59645.graph_c0_235593** | c66658.graph_c0 | 409-425 Slice Site | 416 |
| **CZunconservative_c59645.graph_c0_235593** | c69900.graph_c0 | 2715-2733 Slice Site | 2724 |
| **CZunconservative_c59645.graph_c0_235593** | c70435.graph_c0 | 1170-1187 Slice Site | 1179 |
| **CZunconservative_c59645.graph_c0_235593** | c72456.graph_c0 | 2242-2259 Slice Site | 2250 |
| **CZunconservative_c59645.graph_c0_235593** | c72574.graph_c0 | 2712-2729 Slice Site | 2720 |
| **CZunconservative_c59645.graph_c0_235593** | c74946.graph_c1 | 664-683 Slice Site | 674 |
| **CZunconservative_c59645.graph_c0_235593** | c75720.graph_c0 | 613-630 Slice Site | 622 |
| **CZunconservative_c61800.graph_c0_269757** | c36186.graph_c0 | 472-491 Slice Site | 482 |
| **CZunconservative_c61800.graph_c0_269757** | c55783.graph_c0 | 344-364 Slice Site | 355 |
| **CZunconservative_c61800.graph_c0_269757** | c64122.graph_c0 | 274-294 Slice Site | 285 |
| **CZunconservative_c61800.graph_c0_269757** | c70897.graph_c2 | 587-609 Slice Site | 600 |
| **CZunconservative_c61800.graph_c0_269757** | c72317.graph_c4 | 3203-3223 Slice Site | 3214 |
| **CZunconservative_c61800.graph_c0_269757** | c75583.graph_c0 | 989-1009 Slice Site | 1000 |
| **CZunconservative_c65414.graph_c0_347656** | c54301.graph_c0 | 415-437 Slice Site | 430 |
| **CZunconservative_c65414.graph_c0_347656** | c67848.graph_c0 | 1425-1448 Slice Site | 1439 |
| **CZunconservative_c66346.graph_c0_371993** | c75548.graph_c1 | 86-109 Slice Site | 100 |
| **CZunconservative_c66732.graph_c0_382366** | c71427.graph_c0 | 2189-2208 Slice Site | 2199 |
| **CZunconservative_c66732.graph_c0_382366** | c75469.graph_c2 | 479-499 Slice Site | 489 |
| **CZunconservative_c68536.graph_c0_441234** | c27331.graph_c0 | 1416-1437 Slice Site | 1428 |
| **CZunconservative_c68536.graph_c0_441234** | c34600.graph_c0 | 2-22 Slice Site | 13 |
| **CZunconservative_c68536.graph_c0_441234** | c40983.graph_c0 | 97-117 Slice Site | 108 |
| **CZunconservative_c68536.graph_c0_441234** | c67878.graph_c0 | 1047-1067 Slice Site | 1058 |
| **CZunconservative_c68536.graph_c0_441234** | c70330.graph_c0 | 219-240 Slice Site | 231 |
| **CZunconservative_c68536.graph_c0_441234** | c72104.graph_c0 | 2321-2341 Slice Site | 2332 |
| **CZunconservative_c68536.graph_c0_441234** | c73156.graph_c0 | 1675-1695 Slice Site | 1686 |
| **CZunconservative_c68536.graph_c0_441234** | c75390.graph_c0 | 1263-1282 Slice Site | 1274 |
| **CZunconservative_c68536.graph_c0_441234** | c75749.graph_c0 | 1255-1274 Slice Site | 1265 |
| **CZunconservative_c74385.graph_c0_729027** | c67477.graph_c0 | 747-767 Slice Site | 758 |
| **CZunconservative_c74385.graph_c0_729027** | c75782.graph_c1 | 1522-1541 Slice Site | 1532 |
| **CZunconservative_c87430.graph_c0_809815** | c71854.graph_c2 | 15-38 Slice Site | 29 |
| **CZunconservative_c87430.graph_c0_809815** | c75548.graph_c1 | 51-74 Slice Site | 65 |
| **CZunconservative_c95416.graph_c0_833054** | c53423.graph_c0 | 1106-1126 Slice Site | 1117 |
| **CZunconservative_c95416.graph_c0_833054** | c66134.graph_c0 | 73-92 Slice Site | 84 |
| **CZunconservative_c95416.graph_c0_833054** | c70689.graph_c0 | 641-660 Slice Site | 651 |
| **CZunconservative_c95416.graph_c0_833054** | c72407.graph_c0 | 922-941 Slice Site | 933 |

Annotated Targets:

| **Target gene ID** |  | **COG_class_annotation** | **GO_annotation** | **KEGG_annotation** | **KOG_class** | **KOG_class_annotation** | **Pfam_annotation** | **Swissprot_annotation** | **nr_annotation** |
| --- | --- | --- | --- | --- | --- | --- | --- | --- | --- |
| c73514.graph_c0 | -- | -- | -- | -- | [YU] | -- | CRM1 C terminal | -- | PREDICTED: exportin-4 isoform X1 [Vitis vinifera] |
| c72407.graph_c0 | -- | -- | Molecular Function: microtubule motor activity (GO:0003777);; Molecular Function: ATP binding (GO:0005524);; Cellular Component: kinesin complex (GO:0005871);; Cellular Component: microtubule (GO:0005874);; Biological Process: microtubule-based movement (GO:0007018);; Molecular Function: microtubule binding (GO:0008017);; | -- | -- | -- | Kinesin-related;; Kinesin motor | Kinesin-like protein KIN12B GN=MDB19.16 OS=Arabidopsis thaliana (Mouse-ear cress) PE=1 SV=1 | unnamed protein product [Vitis vinifera] |
| c72317.graph_c4 | [LDA] | -- | Molecular Function: exonuclease activity (GO:0004527);; Cellular Component: intracellular (GO:0005622);; Molecular Function: metal ion binding (GO:0046872);; | K12619|0.0|rcu:RCOM_1597380|5'->3' exoribonuclease, putative | [LA] | -- | XRN 5'-3' exonuclease N-terminus;; Zinc knuckle;; ATPase family associated with various cellular activities (AAA);; AAA domain (Cdc48 subfamily);; AAA ATPase domain;; AAA domain (dynein-related subfamily);; Holliday junction DNA helicase ruvB N-terminus | 5&apos;-3&apos; exoribonuclease 3 GN=XRN3 OS=Arabidopsis thaliana (Mouse-ear cress) PE=2 SV=1 | PREDICTED: 5&apos;-3&apos; exoribonuclease 3 [Vitis vinifera] |
| c19690.graph_c0 | -- | -- | -- | K03006|3e-06|vvi:100264310|similar to NRPB1 (RNA POLYMERASE II LARGE SUBUNIT) | [K] | Transcription | -- | DNA-directed RNA polymerase II subunit 1 GN=F4B14.70 OS=Arabidopsis thaliana (Mouse-ear cress) PE=1 SV=3 | hypothetical protein PLEOSDRAFT_1085994 [Pleurotus ostreatus PC15] |
| c69308.graph_c0 | -- | -- | -- | -- | -- | -- | Domain of unknown function (DUF1338);; Domain of unknown function (DUF1338) | -- | PREDICTED: uncharacterized protein LOC100258620 isoform X1 [Vitis vinifera] |
| c36597.graph_c0 | -- | -- | -- | -- | [B] | Chromatin structure and dynamics | -- | -- | PREDICTED: uncharacterized protein LOC100264211 [Vitis vinifera] |
| c71476.graph_c1 | [J] | Translation, ribosomal structure and biogenesis | Molecular Function: endoribonuclease activity (GO:0004521);; Cellular Component: mitochondrion (GO:0005739);; Cellular Component: vacuole (GO:0005773);; Biological Process: gluconeogenesis (GO:0006094);; Biological Process: glycolysis (GO:0006096);; Cellular Component: chloroplast stroma (GO:0009570);; Cellular Component: thylakoid (GO:0009579);; Biological Process: response to salt stress (GO:0009651);; Cellular Component: chloroplast envelope (GO:0009941);; Molecular Function: deaminase activity (GO:0019239);; Biological Process: glucosinolate biosynthetic process (GO:0019761);; | -- | [J] | Translation, ribosomal structure and biogenesis | Endoribonuclease L-PSP | Reactive Intermediate Deaminase A, chloroplastic {ECO:0000303|PubMed:25070638} (Precursor) OS=Arabidopsis thaliana (Mouse-ear cress) PE=1 SV=1 | PREDICTED: reactive Intermediate Deaminase A, chloroplastic [Vitis vinifera] |
| c50309.graph_c0 | -- | -- | Molecular Function: sequence-specific DNA binding transcription factor activity (GO:0003700);; Biological Process: regulation of transcription, DNA-templated (GO:0006355);; Biological Process: cell proliferation (GO:0008283);; Biological Process: response to cytokinin (GO:0009735);; Biological Process: response to abscisic acid (GO:0009737);; Biological Process: response to gibberellin (GO:0009739);; Biological Process: inflorescence development (GO:0010229);; Biological Process: regulation of defense response (GO:0031347);; | -- | -- | -- | TCP family transcription factor | Transcription factor TCP15 GN=TCP15 OS=Arabidopsis thaliana (Mouse-ear cress) PE=2 SV=1 | PREDICTED: transcription factor TCP15 [Vitis vinifera] |
| c69828.graph_c0 | [KL] | -- | Molecular Function: DNA binding (GO:0003677);; Molecular Function: helicase activity (GO:0004386);; Molecular Function: ATP binding (GO:0005524);; Biological Process: DNA replication initiation (GO:0006270);; Biological Process: regulation of DNA replication (GO:0006275);; Biological Process: cell proliferation (GO:0008283);; Biological Process: determination of bilateral symmetry (GO:0009855);; Biological Process: organ morphogenesis (GO:0009887);; Biological Process: xylem and phloem pattern formation (GO:0010051);; Biological Process: flower morphogenesis (GO:0048439);; Biological Process: negative regulation of biological process (GO:0048519);; Biological Process: regulation of cell cycle (GO:0051726);; | -- | [KL] | -- | SNF2 family N-terminal domain;; Type III restriction enzyme, res subunit | Protein CHROMATIN REMODELING 24 {ECO:0000303|PubMed:16547115} OS=Arabidopsis thaliana (Mouse-ear cress) PE=2 SV=1 | PREDICTED: protein CHROMATIN REMODELING 24 [Vitis vinifera] |
| c69433.graph_c0 | [RTKL] | -- | Molecular Function: protein serine/threonine kinase activity (GO:0004674);; Molecular Function: protein tyrosine kinase activity (GO:0004713);; Molecular Function: ATP binding (GO:0005524);; Cellular Component: cytosol (GO:0005829);; Cellular Component: plasma membrane (GO:0005886);; Biological Process: glycolysis (GO:0006096);; Biological Process: water transport (GO:0006833);; Biological Process: Golgi organization (GO:0007030);; Biological Process: response to temperature stimulus (GO:0009266);; Biological Process: detection of brassinosteroid stimulus (GO:0009729);; Biological Process: response to auxin (GO:0009733);; Biological Process: brassinosteroid mediated signaling pathway (GO:0009742);; Biological Process: multidimensional cell growth (GO:0009825);; Biological Process: leaf morphogenesis (GO:0009965);; Biological Process: hyperosmotic salinity response (GO:0042538);; Biological Process: response to cadmium ion (GO:0046686);; Biological Process: protein autophosphorylation (GO:0046777);; Biological Process: positive regulation of protein export from nucleus (GO:0046827);; Biological Process: root hair elongation (GO:0048767);; | -- | [G] | Carbohydrate transport and metabolism | Protein kinase domain;; Protein tyrosine kinase | Shaggy-related protein kinase iota OS=Arabidopsis thaliana (Mouse-ear cress) PE=2 SV=1 | Shaggy-related protein kinase zeta [Morus notabilis] |
| c55977.graph_c0 | [E] | Amino acid transport and metabolism | Cellular Component: nucleus (GO:0005634);; Cellular Component: cytosol (GO:0005829);; Cellular Component: plasma membrane (GO:0005886);; Biological Process: response to abscisic acid (GO:0009737);; | -- | -- | -- | Aluminium induced protein;; Glutamine amidotransferase domain | Stem-specific protein TSJT1 GN=TSJT1 OS=Nicotiana tabacum (Common tobacco) PE=2 SV=1 | hypothetical protein CICLE_v10005714mg [Citrus clementina] |
| c53375.graph_c0 | -- | -- | -- | -- | [B] | Chromatin structure and dynamics | DEK C terminal domain | Histone deacetylase HDT2 GN=MDJ22.7 OS=Arabidopsis thaliana (Mouse-ear cress) PE=1 SV=2 | hypothetical protein CISIN_1g007522mg [Citrus sinensis] |
| c74518.graph_c0 | -- | -- | Cellular Component: mitochondrion (GO:0005739);; Cellular Component: vacuolar membrane (GO:0005774);; Cellular Component: endoplasmic reticulum (GO:0005783);; Cellular Component: Golgi apparatus (GO:0005794);; Cellular Component: plasma membrane (GO:0005886);; Biological Process: N-terminal protein myristoylation (GO:0006499);; Cellular Component: plasmodesma (GO:0009506);; Biological Process: photorespiration (GO:0009853);; Biological Process: regulation of signal transduction (GO:0009966);; Cellular Component: integral component of membrane (GO:0016021);; Biological Process: protein processing (GO:0016485);; | -- | [E] | Amino acid transport and metabolism | Nicastrin;; Peptidase family M28 | -- | PREDICTED: nicalin-1 [Vitis vinifera] |
| c48146.graph_c0 | [CE] | -- | Molecular Function: magnesium ion binding (GO:0000287);; Molecular Function: isocitrate dehydrogenase (NAD+) activity (GO:0004449);; Biological Process: tricarboxylic acid cycle (GO:0006099);; Molecular Function: NAD binding (GO:0051287);; | K00030|0.0|rcu:RCOM_0519460|isocitrate dehydrogenase, putative (EC:1.1.1.41) | [E] | Amino acid transport and metabolism | Isocitrate/isopropylmalate dehydrogenase | Isocitrate dehydrogenase [NAD] regulatory subunit 1, mitochondrial (Precursor) GN=IDH1 OS=Arabidopsis thaliana (Mouse-ear cress) PE=2 SV=2 | isocitrate dehydrogenase, putative [Ricinus communis] |
| c54821.graph_c0 | -- | -- | -- | K03945|3e-32|rcu:RCOM_0126310|hypothetical protein | -- | -- | -- | NADH dehydrogenase [ubiquinone] 1 alpha subcomplex subunit 1 GN=At3g08610 OS=Arabidopsis thaliana (Mouse-ear cress) PE=3 SV=1 | hypothetical protein [Camellia sinensis] |
| c66032.graph_c1 | -- | -- | -- | -- | -- | -- | -- | -- | hypothetical protein MTR_5g051050, partial [Medicago truncatula] |
| c74883.graph_c1 | -- | -- | -- | -- | -- | -- | SPT2 chromatin protein | Nucleolin 1 OS=Oryza sativa subsp. japonica (Rice) PE=2 SV=1 | PREDICTED: protein spt2 isoform X2 [Vitis vinifera] |
| c69772.graph_c0 | -- | -- | -- | -- | -- | -- | -- | -- | PREDICTED: uncharacterized protein LOC104606362 [Nelumbo nucifera] |
| c66629.graph_c0 | -- | -- | -- | -- | -- | -- | Senescence regulator | -- | PREDICTED: uncharacterized protein LOC104604694 isoform X2 [Nelumbo nucifera] |
| c57663.graph_c0 | -- | -- | Molecular Function: sequence-specific DNA binding transcription factor activity (GO:0003700);; Cellular Component: nucleus (GO:0005634);; Biological Process: regulation of transcription, DNA-templated (GO:0006355);; Biological Process: asymmetric cell division (GO:0008356);; Biological Process: response to heat (GO:0009408);; Molecular Function: sequence-specific DNA binding (GO:0043565);; | -- | [K] | Transcription | -- | Heat stress transcription factor B-4 GN=F2G19.8 OS=Arabidopsis thaliana (Mouse-ear cress) PE=2 SV=1 | PREDICTED: heat stress transcription factor B-4 [Vitis vinifera] |
| c47354.graph_c0 | -- | -- | Molecular Function: protein dimerization activity (GO:0046983);; | -- | -- | -- | Helix-loop-helix DNA-binding domain | Transcription factor bHLH93 GN=K21L13.16 OS=Arabidopsis thaliana (Mouse-ear cress) PE=1 SV=1 | PREDICTED: transcription factor bHLH93-like [Nelumbo nucifera] |
| c37464.graph_c0 | [K] | Transcription | -- | -- | [K] | Transcription | -- | -- | unnamed protein product [Coffea canephora] |
| c63759.graph_c0 | [PR] | -- | Molecular Function: chlorophyllide a oxygenase [overall] activity (GO:0010277);; Molecular Function: metal ion binding (GO:0046872);; Molecular Function: 2 iron, 2 sulfur cluster binding (GO:0051537);; Biological Process: oxidation-reduction process (GO:0055114);; | -- | -- | -- | Pheophorbide a oxygenase;; Rieske [2Fe-2S] domain | Protochlorophyllide-dependent translocon component 52, chloroplastic (Precursor) GN=L73G19.30 OS=Arabidopsis thaliana (Mouse-ear cress) PE=1 SV=1 | PREDICTED: protochlorophyllide-dependent translocon component 52, chloroplastic [Vitis vinifera] |
| c57315.graph_c0 | -- | -- | -- | -- | -- | -- | -- | Sulfated surface glycoprotein 185 (Precursor) OS=Volvox carteri (Green alga) PE=1 SV=1 | PREDICTED: glycine-rich cell wall structural protein [Sesamum indicum] |
| c65556.graph_c0 | [RTKL] | -- | Molecular Function: protein serine/threonine kinase activity (GO:0004674);; Molecular Function: ATP binding (GO:0005524);; Biological Process: protein phosphorylation (GO:0006468);; | -- | [D] | Cell cycle control, cell division, chromosome partitioning | Protein kinase domain;; Protein tyrosine kinase | Probable serine/threonine-protein kinase At1g09600 GN=At1g09600 OS=Arabidopsis thaliana (Mouse-ear cress) PE=3 SV=1 | PREDICTED: probable serine/threonine-protein kinase At1g54610 [Nicotiana sylvestris] |
| c72700.graph_c0 | -- | -- | -- | -- | [GMW] | -- | Exostosin family | Probable glycosyltransferase At5g03795 GN=At5g03795 OS=Arabidopsis thaliana (Mouse-ear cress) PE=3 SV=2 | PREDICTED: probable glycosyltransferase At5g03795 [Vitis vinifera] |
| c72450.graph_c0 | [UD] | -- | -- | -- | [UD] | -- | SAC3/GANP/Nin1/mts3/eIF-3 p25 family;; Flavin containing amine oxidoreductase;; NAD(P)-binding Rossmann-like domain;; FAD dependent oxidoreductase;; Thi4 family;; FAD binding domain | Polyamine oxidase 1 GN=MSH12.17 OS=Arabidopsis thaliana (Mouse-ear cress) PE=1 SV=1 | PREDICTED: uncharacterized protein LOC100266559 [Vitis vinifera] |
| c20671.graph_c0 | [C] | Energy production and conversion | Cellular Component: chloroplast thylakoid membrane (GO:0009535);; Cellular Component: chloroplast envelope (GO:0009941);; Molecular Function: hydrogen ion transmembrane transporter activity (GO:0015078);; Biological Process: ATP synthesis coupled proton transport (GO:0015986);; Cellular Component: integral component of membrane (GO:0016021);; Biological Process: defense response to bacterium (GO:0042742);; Cellular Component: proton-transporting ATP synthase complex, coupling factor F(o) (GO:0045263);; | K02109|2e-90|vvi:100250997|hypothetical protein LOC100250997 | -- | -- | ATP synthase B/B' CF(0) | ATP synthase subunit b&apos;, chloroplastic (Precursor) GN=ATPG OS=Spinacia oleracea (Spinach) PE=1 SV=2 | PREDICTED: ATP synthase subunit b&apos;, chloroplastic-like [Solanum tuberosum] |
| c57196.graph_c0 | [J] | Translation, ribosomal structure and biogenesis | -- | -- | [J] | Translation, ribosomal structure and biogenesis | Ribosomal protein L20 | 50S ribosomal protein L20, chloroplastic GN=rpl20 OS=Chlorella vulgaris (Green alga) PE=3 SV=2 | PREDICTED: 50S ribosomal protein L20, chloroplastic [Sesamum indicum] |
| c69782.graph_c0 | [R] | General function prediction only | -- | -- | [A] | RNA processing and modification | RNA recognition motif. (a.k.a. RRM, RBD, or RNP domain);; RNA recognition motif (a.k.a. RRM, RBD, or RNP domain);; RNA recognition motif. (a.k.a. RRM, RBD, or RNP domain) | Polyadenylate-binding protein 2 GN=PAB2 OS=Arabidopsis thaliana (Mouse-ear cress) PE=1 SV=1 | PREDICTED: nucleolin-like isoform X2 [Nelumbo nucifera] |
| c57472.graph_c0 | -- | -- | Cellular Component: nucleus (GO:0005634);; Biological Process: nucleotide-excision repair (GO:0006289);; | -- | [K] | Transcription | Ubiquitin family | -- | PREDICTED: polyubiquitin 9 [Nelumbo nucifera] |
| c77035.graph_c0 | [R] | General function prediction only | Cellular Component: Golgi membrane (GO:0000139);; Molecular Function: GTPase activity (GO:0003924);; Molecular Function: GTP binding (GO:0005525);; Cellular Component: plasma membrane (GO:0005886);; Biological Process: GTP catabolic process (GO:0006184);; Biological Process: intracellular protein transport (GO:0006886);; Biological Process: ER to Golgi vesicle-mediated transport (GO:0006888);; Biological Process: nucleocytoplasmic transport (GO:0006913);; Biological Process: small GTPase mediated signal transduction (GO:0007264);; Molecular Function: GTP-dependent protein binding (GO:0030742);; Cellular Component: trans-Golgi network membrane (GO:0032588);; Molecular Function: myosin XI tail binding (GO:0080115);; | -- | [TU] | -- | Ras family | Ras-related protein RABD1 GN=F26K24.2 OS=Arabidopsis thaliana (Mouse-ear cress) PE=1 SV=1 | PREDICTED: ras-related protein RABD1 [Vitis vinifera] |
| c66546.graph_c0 | -- | -- | Cellular Component: endoplasmic reticulum (GO:0005783);; Cellular Component: plasmodesma (GO:0009506);; Cellular Component: integral component of membrane (GO:0016021);; | -- | -- | -- | Uncharacterised protein family (UPF0121) | -- | Bifunctional dihydroflavonol 4-reductase/flavanone 4-reductase isoform 1 [Theobroma cacao] |
| c69542.graph_c0 | -- | -- | -- | -- | -- | -- | Zn-finger in Ran binding protein and others | Transcription initiation factor TFIID subunit 15b GN=TAF15B OS=Arabidopsis thaliana (Mouse-ear cress) PE=1 SV=1 | RNA-binding protein-related [Theobroma cacao] |
| c70947.graph_c1 | [M] | Cell wall/membrane/envelope biogenesis | Cellular Component: cytosol (GO:0005829);; Molecular Function: GDP-mannose 4,6-dehydratase activity (GO:0008446);; Biological Process: unidimensional cell growth (GO:0009826);; Biological Process: GDP-mannose metabolic process (GO:0019673);; Biological Process: 'de novo' GDP-L-fucose biosynthetic process (GO:0042351);; Molecular Function: coenzyme binding (GO:0050662);; | K01711|1e-169|pop:POPTR_1083784|GDP-mannose 4,6-dehydratase (EC:4.2.1.47) | [G] | Carbohydrate transport and metabolism | NAD dependent epimerase/dehydratase family | GDP-mannose 4,6 dehydratase 1 GN=GMD1 OS=Arabidopsis thaliana (Mouse-ear cress) PE=1 SV=1 | PREDICTED: GDP-mannose 4,6 dehydratase 1 [Eucalyptus grandis] |
| c62494.graph_c0 | -- | -- | Cellular Component: mitochondrial respiratory chain complex I (GO:0005747);; Biological Process: photorespiration (GO:0009853);; | -- | -- | -- | -- | -- | hypothetical protein EUTSA_v10017500mg [Eutrema salsugineum] |
| c63358.graph_c0 | -- | -- | Biological Process: metabolic process (GO:0008152);; Cellular Component: membrane (GO:0016020);; Molecular Function: transferase activity, transferring glycosyl groups (GO:0016757);; | -- | [GMW] | -- | Exostosin family | Xyloglucan galactosyltransferase KATAMARI1 GN=F11A3.8 OS=Arabidopsis thaliana (Mouse-ear cress) PE=1 SV=1 | PREDICTED: xyloglucan galactosyltransferase KATAMARI1 [Nicotiana tomentosiformis] |
| c71476.graph_c0 | [OC] | -- | Biological Process: glycerol ether metabolic process (GO:0006662);; Molecular Function: electron carrier activity (GO:0009055);; Molecular Function: protein disulfide oxidoreductase activity (GO:0015035);; Molecular Function: isomerase activity (GO:0016853);; Biological Process: cell redox homeostasis (GO:0045454);; | K09584|0.0|vvi:100243120|hypothetical protein LOC100243120 | [O] | Posttranslational modification, protein turnover, chaperones | Thioredoxin;; Thioredoxin-like domain;; Thioredoxin-like domain;; Thioredoxin-like;; Thioredoxin-like;; Thioredoxin domain;; Thioredoxin | Protein disulfide isomerase-like 2-3 (Precursor) OS=Oryza sativa subsp. japonica (Rice) PE=2 SV=1 | PREDICTED: protein disulfide isomerase-like 2-3 [Pyrus x bretschneideri] |
| c74150.graph_c0 | [T] | Signal transduction mechanisms | Molecular Function: phosphorelay response regulator activity (GO:0000156);; Biological Process: phosphorelay signal transduction system (GO:0000160);; Molecular Function: DNA binding (GO:0003677);; Molecular Function: chromatin binding (GO:0003682);; Biological Process: regulation of transcription, DNA-templated (GO:0006355);; Molecular Function: kinase activity (GO:0016301);; Biological Process: phosphorylation (GO:0016310);; Biological Process: intracellular signal transduction (GO:0035556);; | K14491|0.0|rcu:RCOM_0999330|sensor histidine kinase, putative | [K] | Transcription | Response regulator receiver domain;; Myb-like DNA-binding domain | Two-component response regulator ARR2 OS=Arabidopsis thaliana (Mouse-ear cress) PE=1 SV=1 | PREDICTED: two-component response regulator ARR1 isoform X2 [Vitis vinifera] |
| c72570.graph_c0 | [O] | Posttranslational modification, protein turnover, chaperones | Molecular Function: peptide receptor activity (GO:0001653);; Cellular Component: nucleus (GO:0005634);; Cellular Component: cytosol (GO:0005829);; Biological Process: gluconeogenesis (GO:0006094);; Biological Process: glycolysis (GO:0006096);; Biological Process: DNA repair (GO:0006281);; Biological Process: regulation of transcription, DNA-templated (GO:0006355);; Biological Process: protein targeting to vacuole (GO:0006623);; Biological Process: fatty acid beta-oxidation (GO:0006635);; Biological Process: water transport (GO:0006833);; Biological Process: hyperosmotic response (GO:0006972);; Biological Process: cytoskeleton organization (GO:0007010);; Biological Process: Golgi organization (GO:0007030);; Molecular Function: zinc ion binding (GO:0008270);; Cellular Component: proteasome regulatory particle, base subcomplex (GO:0008540);; Biological Process: toxin catabolic process (GO:0009407);; Biological Process: response to heat (GO:0009408);; Biological Process: pollen development (GO:0009555);; Biological Process: response to salt stress (GO:0009651);; Biological Process: response to auxin (GO:0009733);; Biological Process: response to abscisic acid (GO:0009737);; Biological Process: response to sucrose (GO:0009744);; Biological Process: photorespiration (GO:0009853);; Biological Process: regulation of seed germination (GO:0010029);; Biological Process: leaf senescence (GO:0010150);; Cellular Component: membrane (GO:0016020);; Biological Process: proteasome-mediated ubiquitin-dependent protein catabolic process (GO:0043161);; Biological Process: response to cadmium ion (GO:0046686);; Biological Process: stamen formation (GO:0048455);; Biological Process: post-embryonic root development (GO:0048528);; Biological Process: root hair elongation (GO:0048767);; Biological Process: response to misfolded protein (GO:0051788);; Biological Process: proteasome core complex assembly (GO:0080129);; | K03029|0.0|vvi:100257366|hypothetical protein LOC100257366 | [O] | Posttranslational modification, protein turnover, chaperones | Ubiquitin interaction motif;; von Willebrand factor type A domain | 26S proteasome non-ATPase regulatory subunit 4 homolog OS=Arabidopsis thaliana (Mouse-ear cress) PE=1 SV=1 | PREDICTED: 26S proteasome non-ATPase regulatory subunit 4 homolog [Vitis vinifera] |
| c66768.graph_c0 | -- | -- | -- | -- | -- | -- | -- | -- | PREDICTED: suppressor protein SRP40-like [Citrus sinensis] |
| c59112.graph_c0 | -- | -- | -- | -- | [MO] | -- | Phosphatidylinositol-glycan biosynthesis class S protein | -- | PREDICTED: GPI transamidase component PIG-S isoform X3 [Nicotiana tomentosiformis] |
| c114418.graph_c0 | -- | -- | -- | -- | -- | -- | Probable lipid transfer | -- | PREDICTED: uncharacterized protein LOC100252722 [Vitis vinifera] |
| c72526.graph_c0 | [G] | Carbohydrate transport and metabolism | Molecular Function: transketolase activity (GO:0004802);; Biological Process: metabolic process (GO:0008152);; | K00615|0.0|rcu:RCOM_1611360|transketolase, putative (EC:2.2.1.1) | [G] | Carbohydrate transport and metabolism | Transketolase, thiamine diphosphate binding domain;; Transketolase, pyrimidine binding domain;; Transketolase, C-terminal domain | Transketolase, chloroplastic (Precursor) OS=Solanum tuberosum (Potato) PE=2 SV=1 | transketolase [Camellia sinensis] |
| c75277.graph_c0 | [J] | Translation, ribosomal structure and biogenesis | Molecular Function: RNA 7-methylguanosine cap binding (GO:0000340);; Molecular Function: translation initiation factor activity (GO:0003743);; Cellular Component: nucleus (GO:0005634);; Cellular Component: cytosol (GO:0005829);; Biological Process: translational initiation (GO:0006413);; Biological Process: negative regulation of defense response to virus (GO:0050687);; | K03259|1e-125|vvi:100259631|hypothetical protein LOC100259631 | [J] | Translation, ribosomal structure and biogenesis | Eukaryotic initiation factor 4E | Eukaryotic translation initiation factor isoform 4E-2 OS=Triticum aestivum (Wheat) PE=1 SV=1 | PREDICTED: eukaryotic translation initiation factor isoform 4E-2 [Sesamum indicum] |
| c41377.graph_c0 | [T] | Signal transduction mechanisms | Molecular Function: phosphoprotein phosphatase activity (GO:0004721);; | K06269|0.0|rcu:RCOM_0608890|serine/threonine protein phosphatase, putative (EC:3.1.3.16) | [TR] | -- | Calcineurin-like phosphoesterase | Serine/threonine-protein phosphatase PP1 OS=Oryza sativa subsp. japonica (Rice) PE=2 SV=2 | serine/threonine protein phosphatase, putative [Ricinus communis] |
| c69847.graph_c0 | -- | -- | -- | -- | -- | -- | -- | -- | unnamed protein product [Coffea canephora] |
| c74758.graph_c0 | [RTKL] | -- | Molecular Function: protein serine/threonine kinase activity (GO:0004674);; Molecular Function: ATP binding (GO:0005524);; Biological Process: protein phosphorylation (GO:0006468);; Cellular Component: integral component of membrane (GO:0016021);; Molecular Function: 2-alkenal reductase [NAD(P)] activity (GO:0032440);; Biological Process: oxidation-reduction process (GO:0055114);; | -- | -- | -- | Protein kinase domain;; Protein tyrosine kinase;; Leucine Rich repeats (2 copies);; Leucine rich repeat N-terminal domain;; Leucine rich repeat;; Leucine Rich Repeat;; Leucine Rich repeat;; Leucine rich repeat | Protein NSP-INTERACTING KINASE 1 (Precursor) GN=NIK1 OS=Arabidopsis thaliana (Mouse-ear cress) PE=1 SV=1 | PREDICTED: protein NSP-INTERACTING KINASE 1 [Vitis vinifera] |
| c70780.graph_c0 | -- | -- | -- | -- | -- | -- | Ricin-type beta-trefoil lectin domain-like | Leucine-rich repeat extensin-like protein 3 (Precursor) GN=LRX3 OS=Arabidopsis thaliana (Mouse-ear cress) PE=1 SV=1 | PREDICTED: formin-1 isoform X2 [Sesamum indicum] |
| c71237.graph_c0 | [C] | Energy production and conversion | Biological Process: transition metal ion transport (GO:0000041);; Cellular Component: nucleus (GO:0005634);; Cellular Component: vacuole (GO:0005773);; Cellular Component: peroxisome (GO:0005777);; Molecular Function: glycolate oxidase activity (GO:0008891);; Cellular Component: chloroplast stroma (GO:0009570);; Molecular Function: FMN binding (GO:0010181);; Biological Process: defense response signaling pathway, resistance gene-independent (GO:0010204);; Cellular Component: membrane (GO:0016020);; Cellular Component: cytosolic ribosome (GO:0022626);; Biological Process: defense response to bacterium (GO:0042742);; Cellular Component: apoplast (GO:0048046);; Biological Process: hydrogen peroxide biosynthetic process (GO:0050665);; Biological Process: oxidation-reduction process (GO:0055114);; | K11517|8e-59|vvi:100244701|hypothetical protein LOC100244701 | [C] | Energy production and conversion | FMN-dependent dehydrogenase;; IMP dehydrogenase / GMP reductase domain;; Conserved region in glutamate synthase;; Nitronate monooxygenase | Peroxisomal (S)-2-hydroxy-acid oxidase GLO2 GN=MLN21.20 OS=Arabidopsis thaliana (Mouse-ear cress) PE=1 SV=1 | glycolate oxidase [Pachysandra terminalis] |
| c71937.graph_c0 | [E] | Amino acid transport and metabolism | Molecular Function: 3-deoxy-7-phosphoheptulonate synthase activity (GO:0003849);; Biological Process: aromatic amino acid family biosynthetic process (GO:0009073);; Biological Process: chorismate biosynthetic process (GO:0009423);; Cellular Component: chloroplast (GO:0009507);; Biological Process: response to wounding (GO:0009611);; Biological Process: systemic acquired resistance (GO:0009627);; Biological Process: coumarin biosynthetic process (GO:0009805);; Cellular Component: membrane (GO:0016020);; Molecular Function: lyase activity (GO:0016829);; Biological Process: response to endoplasmic reticulum stress (GO:0034976);; | K01626|0.0|vvi:100233115|3-deoxy-D-arabino-heptulosonate 7-phosphate synthase 02 | -- | -- | Class-II DAHP synthetase family | Phospho-2-dehydro-3-deoxyheptonate aldolase 1, chloroplastic (Precursor) GN=DHAPS-1 OS=Nicotiana tabacum (Common tobacco) PE=1 SV=1 | PREDICTED: phospho-2-dehydro-3-deoxyheptonate aldolase 1, chloroplastic-like [Pyrus x bretschneideri] |
| c72095.graph_c1 | [C] | Energy production and conversion | Molecular Function: L-lactate dehydrogenase activity (GO:0004459);; Cellular Component: cytoplasm (GO:0005737);; Biological Process: glycolysis (GO:0006096);; Biological Process: cellular carbohydrate metabolic process (GO:0044262);; Biological Process: oxidation-reduction process (GO:0055114);; | K00016|0.0|rcu:RCOM_0540790|l-lactate dehydrogenase, putative (EC:1.1.1.27) | [C] | Energy production and conversion | lactate/malate dehydrogenase, NAD binding domain;; lactate/malate dehydrogenase, alpha/beta C-terminal domain | L-lactate dehydrogenase A OS=Hordeum vulgare (Barley) PE=1 SV=1 | lactate dehydrogenase [Camellia sinensis] |
| c73469.graph_c0 | [P] | Inorganic ion transport and metabolism | Molecular Function: potassium ion transmembrane transporter activity (GO:0015079);; Cellular Component: membrane (GO:0016020);; Biological Process: potassium ion transmembrane transport (GO:0071805);; | -- | -- | -- | K+ potassium transporter | Probable potassium transporter 17 OS=Oryza sativa subsp. japonica (Rice) PE=2 SV=2 | hypothetical protein POPTR_0010s11100g [Populus trichocarpa] |
| c74365.graph_c0 | -- | -- | Cellular Component: plasma membrane (GO:0005886);; Cellular Component: chloroplast (GO:0009507);; Biological Process: response to salt stress (GO:0009651);; | -- | -- | -- | AIG2-like family | AIG2-like protein OS=Arabidopsis thaliana (Mouse-ear cress) PE=1 SV=1 | PREDICTED: AIG2-like protein isoform X4 [Nelumbo nucifera] |
| c65316.graph_c0 | -- | -- | Cellular Component: chloroplast thylakoid membrane (GO:0009535);; Biological Process: response to blue light (GO:0009637);; Biological Process: photosynthesis, light harvesting (GO:0009765);; Cellular Component: chloroplast envelope (GO:0009941);; Biological Process: response to red light (GO:0010114);; Biological Process: response to far red light (GO:0010218);; Cellular Component: plastoglobule (GO:0010287);; Molecular Function: metal ion binding (GO:0046872);; | K08915|0.0|rcu:RCOM_0644360|chlorophyll A/B binding protein, putative | -- | -- | Chlorophyll A-B binding protein | Chlorophyll a-b binding protein CP29.1, chloroplastic (Precursor) GN=LHCB4.1 OS=Arabidopsis thaliana (Mouse-ear cress) PE=1 SV=1 | chlorophyll A/B binding protein, putative [Ricinus communis] |
| c46006.graph_c0 | [K] | Transcription | Molecular Function: DNA binding (GO:0003677);; Molecular Function: DNA-directed RNA polymerase activity (GO:0003899);; Cellular Component: nucleus (GO:0005634);; Biological Process: transcription, DNA-templated (GO:0006351);; Molecular Function: zinc ion binding (GO:0008270);; | K03017|4e-80|vvi:100253286|hypothetical protein LOC100253286 | [K] | Transcription | Transcription factor S-II (TFIIS);; RNA polymerases M/15 Kd subunit | DNA-directed RNA polymerases II, IV and V subunit 9A GN=K14A17 OS=Arabidopsis thaliana (Mouse-ear cress) PE=1 SV=1 | PREDICTED: DNA-directed RNA polymerases II, IV and V subunit 9A [Sesamum indicum] |
| c19855.graph_c1 | [K] | Transcription | Cellular Component: nucleus (GO:0005634);; Molecular Function: zinc ion binding (GO:0008270);; Biological Process: positive regulation of DNA-templated transcription, elongation (GO:0032786);; | -- | [K] | Transcription | Spt4/RpoE2 zinc finger | Transcription elongation factor SPT4 homolog 2 GN=At5g63670 OS=Arabidopsis thaliana (Mouse-ear cress) PE=2 SV=1 | hypothetical protein POPTR_0010s26160g [Populus trichocarpa] |
| c55032.graph_c0 | -- | -- | -- | -- | -- | -- | Plant transposase (Ptta/En/Spm family) | -- | PREDICTED: uncharacterized protein LOC103718272 [Phoenix dactylifera] |
| c75261.graph_c0 | -- | -- | Molecular Function: translation initiation factor activity (GO:0003743);; Cellular Component: cytosol (GO:0005829);; Cellular Component: plasma membrane (GO:0005886);; Biological Process: gluconeogenesis (GO:0006094);; Biological Process: translational initiation (GO:0006413);; Biological Process: cytoskeleton organization (GO:0007010);; Cellular Component: chloroplast (GO:0009507);; Biological Process: proteasomal protein catabolic process (GO:0010498);; Biological Process: intracellular distribution of mitochondria (GO:0048312);; | -- | [R] | General function prediction only | Clustered mitochondria;; Translation initiation factor eIF3 subunit 135;; Tetratricopeptide repeat;; Mitochondrial function, CLU-N-term;; Tetratricopeptide repeat;; TPR repeat;; Protein of unknown function (DUF727) | Clustered mitochondria protein GN=F4F15.250 OS=Arabidopsis thaliana (Mouse-ear cress) PE=2 SV=1 | PREDICTED: clustered mitochondria protein [Vitis vinifera] |
| c66008.graph_c0 | -- | -- | -- | -- | -- | -- | Protein of unknown function (DUF4057) | Alpha-ketoglutarate-dependent dioxygenase alkB homolog 2 GN=ALKBH2 OS=Arabidopsis thaliana (Mouse-ear cress) PE=2 SV=2 | hypothetical protein JCGZ_15776 [Jatropha curcas] |
| c75927.graph_c1 | -- | -- | -- | -- | -- | -- | -- | -- | PREDICTED: uncharacterized protein LOC104878107 [Vitis vinifera] |
| c71875.graph_c0 | -- | -- | -- | -- | [KTDL] | -- | BRCA1 C Terminus (BRCT) domain | -- | PREDICTED: uncharacterized protein LOC100266667 [Vitis vinifera] |
| c66351.graph_c0 | [O] | Posttranslational modification, protein turnover, chaperones | Biological Process: protein peptidyl-prolyl isomerization (GO:0000413);; Molecular Function: peptidyl-prolyl cis-trans isomerase activity (GO:0003755);; Biological Process: protein folding (GO:0006457);; Cellular Component: chloroplast stromal thylakoid (GO:0009533);; Cellular Component: chloroplast thylakoid membrane (GO:0009535);; Cellular Component: chloroplast thylakoid lumen (GO:0009543);; Biological Process: NAD(P)H dehydrogenase complex assembly (GO:0010275);; Biological Process: cysteine biosynthetic process (GO:0019344);; Molecular Function: protein histidine kinase binding (GO:0043424);; | K03768|1e-130|rcu:RCOM_1342590|peptidyl-prolyl cis-trans isomerase, putative (EC:5.2.1.8) | [O] | Posttranslational modification, protein turnover, chaperones | Cyclophilin type peptidyl-prolyl cis-trans isomerase/CLD | Peptidyl-prolyl cis-trans isomerase CYP20-3, chloroplastic (Precursor) GN=F21F14.200 OS=Arabidopsis thaliana (Mouse-ear cress) PE=1 SV=1 | PREDICTED: peptidyl-prolyl cis-trans isomerase [Vitis vinifera] |
| c64290.graph_c0 | -- | -- | Molecular Function: hydrolase activity, hydrolyzing O-glycosyl compounds (GO:0004553);; Biological Process: carbohydrate metabolic process (GO:0005975);; Cellular Component: anchored component of membrane (GO:0031225);; Molecular Function: cation binding (GO:0043169);; | -- | -- | -- | Glycosyl hydrolases family 17 | Glucan endo-1,3-beta-glucosidase 11 (Precursor) GN=At1g32860 OS=Arabidopsis thaliana (Mouse-ear cress) PE=1 SV=1 | PREDICTED: glucan endo-1,3-beta-glucosidase 14-like [Pyrus x bretschneideri] |
| c67909.graph_c0 | -- | -- | -- | -- | -- | -- | -- | -- | PREDICTED: uncharacterized protein LOC100244084 [Vitis vinifera] |
| c57967.graph_c0 | [E] | Amino acid transport and metabolism | Molecular Function: L-aspartate:2-oxoglutarate aminotransferase activity (GO:0004069);; Molecular Function: aromatic-amino-acid:2-oxoglutarate aminotransferase activity (GO:0008793);; Biological Process: ethylene biosynthetic process (GO:0009693);; Molecular Function: 1-aminocyclopropane-1-carboxylate synthase activity (GO:0016847);; Molecular Function: pyridoxal phosphate binding (GO:0030170);; Biological Process: 1-aminocyclopropane-1-carboxylate biosynthetic process (GO:0042218);; | K14270|0.0|vvi:100249614|hypothetical protein LOC100249614 | [T] | Signal transduction mechanisms | Aminotransferase class I and II | Probable aminotransferase ACS10 GN=ACS10 OS=Arabidopsis thaliana (Mouse-ear cress) PE=1 SV=1 | unnamed protein product [Coffea canephora] |
| c55205.graph_c0 | [F] | Nucleotide transport and metabolism | Molecular Function: amidophosphoribosyltransferase activity (GO:0004044);; Cellular Component: cell wall (GO:0005618);; Biological Process: 'de novo' IMP biosynthetic process (GO:0006189);; Biological Process: purine nucleobase biosynthetic process (GO:0009113);; Biological Process: nucleoside metabolic process (GO:0009116);; Cellular Component: chloroplast (GO:0009507);; Cellular Component: plastid stroma (GO:0009532);; Biological Process: leaf morphogenesis (GO:0009965);; Molecular Function: metal ion binding (GO:0046872);; Molecular Function: iron-sulfur cluster binding (GO:0051536);; | K00764|0.0|pop:POPTR_1076823|amidophosphoribosyltransferase (EC:2.4.2.14) | [F] | Nucleotide transport and metabolism | Glutamine amidotransferases class-II;; Glutamine amidotransferase domain;; Glutamine amidotransferase domain;; Phosphoribosyl transferase domain;; Glutamine amidotransferases class-II | Amidophosphoribosyltransferase 1, chloroplastic (Precursor) GN=F1P15 OS=Arabidopsis thaliana (Mouse-ear cress) PE=2 SV=1 | hypothetical protein CICLE_v10028076mg [Citrus clementina] |
| c74659.graph_c1 | -- | -- | Molecular Function: ATP binding (GO:0005524);; Cellular Component: plasma membrane (GO:0005886);; Biological Process: ATP catabolic process (GO:0006200);; Biological Process: ATP biosynthetic process (GO:0006754);; Biological Process: cation transport (GO:0006812);; Biological Process: response to salt stress (GO:0009651);; Cellular Component: integral component of membrane (GO:0016021);; Molecular Function: cation-transporting ATPase activity (GO:0019829);; Molecular Function: metal ion binding (GO:0046872);; | K01535|0.0|vvi:100241576|similar to plasma membrane H+ ATPase | [P] | Inorganic ion transport and metabolism | haloacid dehalogenase-like hydrolase;; haloacid dehalogenase-like hydrolase | Plasma membrane ATPase 3 GN=PMA3 OS=Nicotiana plumbaginifolia (Leadwort-leaved tobacco) PE=1 SV=1 | plasma membrane H+-ATPase [Sesbania rostrata] |
| c67721.graph_c0 | -- | -- | Cellular Component: mitochondrial proton-transporting ATP synthase complex, catalytic core F(1) (GO:0000275);; Biological Process: photorespiration (GO:0009853);; Biological Process: ATP synthesis coupled proton transport (GO:0015986);; Molecular Function: proton-transporting ATP synthase activity, rotational mechanism (GO:0046933);; Molecular Function: proton-transporting ATPase activity, rotational mechanism (GO:0046961);; | K02135|1e-39|rcu:RCOM_1460050|ATP synthase epsilon chain, mitochondrial, putative (EC:3.6.3.14) | [C] | Energy production and conversion | Mitochondrial ATP synthase epsilon chain | ATP synthase subunit epsilon, mitochondrial OS=Ipomoea batatas (Sweet potato) PE=1 SV=2 | PREDICTED: ATP synthase subunit epsilon, mitochondrial-like [Nicotiana tomentosiformis] |
| c71396.graph_c0 | -- | -- | -- | -- | [O] | Posttranslational modification, protein turnover, chaperones | Ring finger domain;; RING-H2 zinc finger;; Zinc finger, C3HC4 type (RING finger);; Zinc finger, C3HC4 type (RING finger);; Zinc finger, C3HC4 type (RING finger) | E3 ubiquitin-protein ligase SDIR1 GN=SDIR1 OS=Arabidopsis thaliana (Mouse-ear cress) PE=1 SV=1 | PREDICTED: uncharacterized protein LOC103324317 isoform X2 [Prunus mume] |
| c75709.graph_c0 | [V] | Defense mechanisms | Molecular Function: ATP binding (GO:0005524);; Biological Process: ATP catabolic process (GO:0006200);; Cellular Component: integral component of membrane (GO:0016021);; Molecular Function: ATPase activity, coupled to transmembrane movement of substances (GO:0042626);; Biological Process: transmembrane transport (GO:0055085);; | -- | [Q] | Secondary metabolites biosynthesis, transport and catabolism | ABC transporter;; ABC transporter transmembrane region;; Miro-like protein;; AAA domain;; Domain of unknown function DUF87;; 50S ribosome-binding GTPase;; Dynamin family;; FtsK/SpoIIIE family | ABC transporter C family member 2 GN=T29F13.13 OS=Arabidopsis thaliana (Mouse-ear cress) PE=1 SV=2 | PREDICTED: ABC transporter C family member 12-like [Vitis vinifera] |
| c75779.graph_c2 | -- | -- | -- | -- | -- | -- | WRC;; QLQ | Growth-regulating factor 3 GN=GRF3 OS=Arabidopsis thaliana (Mouse-ear cress) PE=2 SV=1 | Growth-regulating factor 5 isoform 3 [Theobroma cacao] |
| c64161.graph_c0 | [T] | Signal transduction mechanisms | Molecular Function: phosphoprotein phosphatase activity (GO:0004721);; Cellular Component: nucleus (GO:0005634);; Biological Process: metabolic process (GO:0008152);; | -- | [T] | Signal transduction mechanisms | Protein phosphatase 2C | Probable protein phosphatase 2C 33 GN=PPC6-1 OS=Arabidopsis thaliana (Mouse-ear cress) PE=1 SV=1 | phosphatase 2C family protein [Populus trichocarpa] |
| c60342.graph_c3 | -- | -- | Molecular Function: catalytic activity (GO:0003824);; | -- | [WV] | -- | -- | -- | hypothetical protein S7711_07062, partial [Stachybotrys chartarum IBT 7711] |
| c54310.graph_c0 | -- | -- | Molecular Function: mRNA binding (GO:0003729);; Cellular Component: mRNA cleavage factor complex (GO:0005849);; Biological Process: mRNA polyadenylation (GO:0006378);; Molecular Function: hydrolase activity (GO:0016787);; | K14397|1e-140|rcu:RCOM_1496400|pre-mRNA cleavage factor im, 25kD subunit, putative | [A] | RNA processing and modification | Nucleotide hydrolase | Pre-mRNA cleavage factor Im 25 kDa subunit 2 {ECO:0000303|PubMed:18479511} OS=Arabidopsis thaliana (Mouse-ear cress) PE=1 SV=1 | hypothetical protein CICLE_v10005929mg [Citrus clementina] |
| c69439.graph_c0 | [O] | Posttranslational modification, protein turnover, chaperones | Molecular Function: glutathione transferase activity (GO:0004364);; | K00799|1e-130|rcu:RCOM_0324140|glutathione-s-transferase theta, gst, putative (EC:2.5.1.18) | [O] | Posttranslational modification, protein turnover, chaperones | Glutathione S-transferase, C-terminal domain;; Glutathione S-transferase, N-terminal domain;; Glutathione S-transferase, N-terminal domain;; Glutathione S-transferase, N-terminal domain;; Glutathione S-transferase, C-terminal domain | Glutathione S-transferase F9 GN=F7F1.7 OS=Arabidopsis thaliana (Mouse-ear cress) PE=1 SV=1 | hypothetical protein JCGZ_04912 [Jatropha curcas] |
| c71254.graph_c0 | -- | -- | Cellular Component: intracellular (GO:0005622);; Molecular Function: zinc ion binding (GO:0008270);; Biological Process: gravitropism (GO:0009630);; | -- | [R] | General function prediction only | Ankyrin repeats (3 copies);; Ankyrin repeats (many copies);; Ankyrin repeats (many copies) | -- | unnamed protein product [Vitis vinifera] |
| c72804.graph_c0 | [F] | Nucleotide transport and metabolism | Molecular Function: dihydrofolate reductase activity (GO:0004146);; Molecular Function: thymidylate synthase activity (GO:0004799);; Biological Process: dTMP biosynthetic process (GO:0006231);; Biological Process: glycine biosynthetic process (GO:0006545);; Biological Process: one-carbon metabolic process (GO:0006730);; Biological Process: methylation (GO:0032259);; Biological Process: tetrahydrofolate biosynthetic process (GO:0046654);; Biological Process: oxidation-reduction process (GO:0055114);; | K13998|1e-174|vvi:100267529|similar to Bifunctional dihydrofolate reductase-thymidylate synthase | [F] | Nucleotide transport and metabolism | Dihydrofolate reductase;; Thymidylate synthase;; Thymidylate synthase | Thymidylate synthase OS=Daucus carota (Wild carrot) PE=2 SV=1 | PREDICTED: bifunctional dihydrofolate reductase-thymidylate synthase-like isoform X1 [Pyrus x bretschneideri] |
| c61514.graph_c0 | -- | -- | Molecular Function: RNA binding (GO:0003723);; | -- | [AR] | -- | KH domain;; KH domain;; KH domain;; KH domain;; KH domain | KH domain-containing protein At4g18375 GN=At4g18375 OS=Arabidopsis thaliana (Mouse-ear cress) PE=2 SV=1 | PREDICTED: KH domain-containing protein At4g18375 [Nicotiana sylvestris] |
| c76175.graph_c2 | [V] | Defense mechanisms | Molecular Function: ATP binding (GO:0005524);; Biological Process: ATP catabolic process (GO:0006200);; Cellular Component: integral component of membrane (GO:0016021);; Molecular Function: ATPase activity, coupled to transmembrane movement of substances (GO:0042626);; Biological Process: transmembrane transport (GO:0055085);; | -- | [Q] | Secondary metabolites biosynthesis, transport and catabolism | ABC transporter transmembrane region;; ABC transporter;; AAA domain;; Miro-like protein;; 50S ribosome-binding GTPase;; Domain of unknown function DUF87;; Dynamin family;; Helix-loop-helix DNA-binding domain | ABC transporter C family member 2 GN=T29F13.13 OS=Arabidopsis thaliana (Mouse-ear cress) PE=1 SV=2 | PREDICTED: ABC transporter C family member 2 [Vitis vinifera] |
| c55937.graph_c0 | [Z] | Cytoskeleton | Molecular Function: GTPase activity (GO:0003924);; Molecular Function: structural constituent of cytoskeleton (GO:0005200);; Molecular Function: GTP binding (GO:0005525);; Cellular Component: cytoplasm (GO:0005737);; Cellular Component: microtubule (GO:0005874);; Biological Process: GTP catabolic process (GO:0006184);; Biological Process: microtubule-based process (GO:0007017);; Biological Process: protein polymerization (GO:0051258);; | -- | [Z] | Cytoskeleton | -- | Tubulin beta-7 chain GN=F16P2.7 OS=Arabidopsis thaliana (Mouse-ear cress) PE=2 SV=1 | beta-tubulin 3 [Populus tremuloides] |
| c62586.graph_c0 | [R] | General function prediction only | Biological Process: protein targeting to vacuole (GO:0006623);; Biological Process: membrane fusion (GO:0006944);; Cellular Component: integral component of membrane (GO:0016021);; Biological Process: endosomal transport (GO:0016197);; Biological Process: Golgi vesicle transport (GO:0048193);; | K06890|1e-135|vvi:100266616|hypothetical protein LOC100266616 | [T] | Signal transduction mechanisms | Inhibitor of apoptosis-promoting Bax1 | BI1-like protein GN=At4g15470 OS=Arabidopsis thaliana (Mouse-ear cress) PE=2 SV=1 | PREDICTED: BI1-like protein [Nicotiana tomentosiformis] |
| c75467.graph_c0 | -- | -- | Cellular Component: plasma membrane (GO:0005886);; Biological Process: carbohydrate metabolic process (GO:0005975);; Cellular Component: plant-type cell wall (GO:0009505);; Biological Process: response to abscisic acid (GO:0009737);; Molecular Function: isomerase activity (GO:0016853);; Molecular Function: carbohydrate binding (GO:0030246);; | K01792|3e-24|vvi:100259893|hypothetical protein LOC100259893 | [G] | Carbohydrate transport and metabolism | Aldose 1-epimerase | Putative glucose-6-phosphate 1-epimerase OS=Cenchrus ciliaris (Buffelgrass) PE=2 SV=1 | PREDICTED: putative glucose-6-phosphate 1-epimerase [Vitis vinifera] |
| c70005.graph_c0 | -- | -- | -- | -- | -- | -- | -- | Uncharacterized protein At1g10890 GN=At1g10890 OS=Arabidopsis thaliana (Mouse-ear cress) PE=4 SV=1 | PREDICTED: uncharacterized protein LOC100257222 [Vitis vinifera] |
| c44069.graph_c1 | -- | -- | -- | -- | -- | -- | -- | -- | TCP family transcription factor, putative [Theobroma cacao] |
| c70721.graph_c0 | -- | -- | -- | -- | -- | -- | BURP domain | Polygalacturonase-1 non-catalytic subunit beta (Precursor) GN=GP1 OS=Solanum lycopersicum (Tomato) PE=1 SV=1 | PREDICTED: probable polygalacturonase non-catalytic subunit JP650 [Nelumbo nucifera] |
| c68807.graph_c0 | -- | -- | -- | -- | -- | -- | DnaJ domain | -- | PREDICTED: caldesmon [Prunus mume] |
| c62523.graph_c0 | [O] | Posttranslational modification, protein turnover, chaperones | Biological Process: protein folding (GO:0006457);; Molecular Function: unfolded protein binding (GO:0051082);; | K09510|1e-112|vvi:100246709|hypothetical protein LOC100246709 | [O] | Posttranslational modification, protein turnover, chaperones | DnaJ domain | DnaJ protein homolog (Precursor) GN=DNAJ1 OS=Cucumis sativus (Cucumber) PE=2 SV=1 | PREDICTED: dnaJ homolog subfamily B member 1-like isoform X2 [Vitis vinifera] |
| c32835.graph_c0 | [J] | Translation, ribosomal structure and biogenesis | Biological Process: RNA methylation (GO:0001510);; Molecular Function: structural constituent of ribosome (GO:0003735);; Biological Process: translation (GO:0006412);; Cellular Component: cytosolic large ribosomal subunit (GO:0022625);; | K02901|2e-81|vvi:100261052|hypothetical protein LOC100261052 | [J] | Translation, ribosomal structure and biogenesis | Ribosomal L27e protein family | 60S ribosomal protein L27-3 GN=RPL27C OS=Arabidopsis thaliana (Mouse-ear cress) PE=2 SV=2 | PREDICTED: 60S ribosomal protein L27-3 [Musa acuminata subsp. malaccensis] |
| c45599.graph_c0 | -- | -- | -- | -- | [R] | General function prediction only | PPR repeat family;; Pentatricopeptide repeat domain;; PPR repeat;; PPR repeat | Pentatricopeptide repeat-containing protein At1g26460, mitochondrial (Precursor) GN=At1g26460 OS=Arabidopsis thaliana (Mouse-ear cress) PE=2 SV=1 | PREDICTED: pentatricopeptide repeat-containing protein At1g26460, mitochondrial [Vitis vinifera] |
| c74349.graph_c1 | [O] | Posttranslational modification, protein turnover, chaperones | Molecular Function: catalytic activity (GO:0003824);; Biological Process: metabolic process (GO:0008152);; | -- | [R] | General function prediction only | Protein of unknown function, DUF255 | -- | hypothetical protein PRUPE_ppa001352mg [Prunus persica] |
| c57223.graph_c0 | -- | -- | Molecular Function: DNA binding (GO:0003677);; Molecular Function: sequence-specific DNA binding transcription factor activity (GO:0003700);; Cellular Component: nucleus (GO:0005634);; Cellular Component: cytoplasm (GO:0005737);; Biological Process: regulation of transcription, DNA-templated (GO:0006355);; Molecular Function: protein dimerization activity (GO:0046983);; Biological Process: ovule development (GO:0048481);; | K09264|1e-111|vvi:100232978|MADS9, PI | [K] | Transcription | SRF-type transcription factor (DNA-binding and dimerisation domain);; K-box region | Floral homeotic protein PMADS 2 GN=PMADS2 OS=Petunia hybrida (Petunia) PE=2 SV=1 | MADS domain transcription factor [Camellia japonica] |
| c26123.graph_c1 | [O] | Posttranslational modification, protein turnover, chaperones | Cellular Component: nucleus (GO:0005634);; Biological Process: response to heat (GO:0009408);; Biological Process: protein sumoylation (GO:0016925);; Molecular Function: protein tag (GO:0031386);; | K12160|3e-30|rcu:RCOM_0753100|hypothetical protein | [O] | Posttranslational modification, protein turnover, chaperones | Ubiquitin-2 like Rad60 SUMO-like | Small ubiquitin-related modifier 1 GN=F10M23.180 OS=Arabidopsis thaliana (Mouse-ear cress) PE=1 SV=2 | ubiquitin-like family protein [Camellia sinensis var. sinensis] |
| c72111.graph_c0 | [R] | General function prediction only | Molecular Function: nucleotide binding (GO:0000166);; Molecular Function: RNA binding (GO:0003723);; Cellular Component: nucleolus (GO:0005730);; Cellular Component: cytoplasm (GO:0005737);; Biological Process: RNA processing (GO:0006396);; Cellular Component: nuclear body (GO:0016604);; Cellular Component: exon-exon junction complex (GO:0035145);; | K12876|1e-115|vvi:100260882|hypothetical protein LOC100260882 | [R] | General function prediction only | RNA recognition motif. (a.k.a. RRM, RBD, or RNP domain);; RNA recognition motif (a.k.a. RRM, RBD, or RNP domain);; RNA recognition motif. (a.k.a. RRM, RBD, or RNP domain);; Plant mobile domain | Serine/arginine-rich splicing factor SC35 GN=SC35 OS=Arabidopsis thaliana (Mouse-ear cress) PE=1 SV=1 | PREDICTED: RNA-binding protein 8A [Vitis vinifera] |
| c69687.graph_c0 | -- | -- | Molecular Function: zinc ion binding (GO:0008270);; Biological Process: chlorophyll catabolic process (GO:0015996);; | -- | [O] | Posttranslational modification, protein turnover, chaperones | Zinc finger, C3HC4 type (RING finger);; Ring finger domain;; Zinc finger, C3HC4 type (RING finger);; Zinc finger, C3HC4 type (RING finger) | -- | RING/U-box superfamily protein [Theobroma cacao] |
| c42478.graph_c0 | -- | -- | Cellular Component: chloroplast thylakoid membrane (GO:0009535);; Cellular Component: chloroplast envelope (GO:0009941);; Biological Process: protein targeting to chloroplast (GO:0045036);; | -- | -- | -- | -- | -- | PREDICTED: uncharacterized protein LOC105175213 [Sesamum indicum] |
| c40469.graph_c0 | -- | -- | Cellular Component: cell part (GO:0044464);; | K14499|1e-119|vvi:100243702|hypothetical protein LOC100243702 | -- | -- | -- | BRI1 kinase inhibitor 1 GN=BKI1 OS=Arabidopsis thaliana (Mouse-ear cress) PE=1 SV=1 | PREDICTED: BRI1 kinase inhibitor 1 [Vitis vinifera] |
| c74969.graph_c2 | -- | -- | Molecular Function: GTPase activator activity (GO:0005096);; Molecular Function: phospholipid binding (GO:0005543);; Cellular Component: intracellular (GO:0005622);; Biological Process: signal transduction (GO:0007165);; Biological Process: positive regulation of GTPase activity (GO:0043547);; | -- | [T] | Signal transduction mechanisms | Leucine-zipper of ternary complex factor MIP1 | Rho GTPase-activating protein 7 GN=ROPGAP7 OS=Arabidopsis thaliana (Mouse-ear cress) PE=2 SV=1 | PREDICTED: rho GTPase-activating protein 7 isoform X2 [Vitis vinifera] |
| c43479.graph_c1 | -- | -- | -- | -- | [K] | Transcription | CCT motif;; B-box zinc finger | Zinc finger protein CONSTANS-LIKE 16 GN=COL16 OS=Arabidopsis thaliana (Mouse-ear cress) PE=2 SV=2 | PREDICTED: zinc finger protein CONSTANS-LIKE 16-like [Nelumbo nucifera] |
| c76314.graph_c0 | [L] | Replication, recombination and repair | Biological Process: DNA repair (GO:0006281);; Biological Process: cellular macromolecule biosynthetic process (GO:0034645);; | K03509|0.0|vvi:100266192|hypothetical protein LOC100266192 | [L] | Replication, recombination and repair | impB/mucB/samB family;; impB/mucB/samB family C-terminal domain | DNA polymerase eta OS=Arabidopsis thaliana (Mouse-ear cress) PE=1 SV=1 | PREDICTED: DNA polymerase eta-like [Fragaria vesca subsp. vesca] |
| c68749.graph_c0 | [R] | General function prediction only | Cellular Component: Golgi apparatus (GO:0005794);; Biological Process: protein secretion (GO:0009306);; | -- | [R] | General function prediction only | C2 domain | Synaptotagmin-1 OS=Arabidopsis thaliana (Mouse-ear cress) PE=1 SV=2 | PREDICTED: synaptotagmin-2-like [Nelumbo nucifera] |
| c45987.graph_c0 | [T] | Signal transduction mechanisms | Biological Process: transition metal ion transport (GO:0000041);; Biological Process: phosphorelay signal transduction system (GO:0000160);; Cellular Component: nucleus (GO:0005634);; Cellular Component: cytoplasm (GO:0005737);; Biological Process: embryo sac development (GO:0009553);; Molecular Function: histidine phosphotransfer kinase activity (GO:0009927);; Biological Process: regulation of seed germination (GO:0010029);; Biological Process: inorganic anion transport (GO:0015698);; Biological Process: cell growth (GO:0016049);; Biological Process: regulation of anthocyanin metabolic process (GO:0031537);; Molecular Function: 2-alkenal reductase [NAD(P)] activity (GO:0032440);; Molecular Function: protein histidine kinase binding (GO:0043424);; Biological Process: regulation of shoot system development (GO:0048831);; Biological Process: cell division (GO:0051301);; Biological Process: oxidation-reduction process (GO:0055114);; Biological Process: regulation of cytokinin-activated signaling pathway (GO:0080036);; | K14490|3e-55|vvi:100250410|hypothetical protein LOC100250410 | [T] | Signal transduction mechanisms | Hpt domain | Histidine-containing phosphotransfer protein 1 GN=MIL23.8 OS=Arabidopsis thaliana (Mouse-ear cress) PE=1 SV=1 | Histidine-containing phosphotransfer protein 1 isoform 1 [Theobroma cacao] |
| c72782.graph_c0 | -- | -- | Molecular Function: nucleotide binding (GO:0000166);; Biological Process: glucose metabolic process (GO:0006006);; Molecular Function: oxidoreductase activity (GO:0016491);; Biological Process: oxidation-reduction process (GO:0055114);; | K00036|0.0|vvi:100254628|hypothetical protein LOC100254628 | [G] | Carbohydrate transport and metabolism | Glucose-6-phosphate dehydrogenase, C-terminal domain;; Glucose-6-phosphate dehydrogenase, NAD binding domain | Glucose-6-phosphate 1-dehydrogenase 4, chloroplastic (Precursor) GN=At1g09420 OS=Arabidopsis thaliana (Mouse-ear cress) PE=2 SV=1 | PREDICTED: glucose-6-phosphate 1-dehydrogenase 4, chloroplastic isoform X1 [Vitis vinifera] |
| c60424.graph_c0 | -- | -- | -- | -- | -- | -- | -- | -- | PREDICTED: uncharacterized protein LOC100247652 [Vitis vinifera] |
| c66291.graph_c0 | -- | -- | Molecular Function: RNA binding (GO:0003723);; | -- | -- | -- | CRS1 / YhbY (CRM) domain | CRS2-associated factor 2, chloroplastic (Precursor) GN=At1g23400 OS=Arabidopsis thaliana (Mouse-ear cress) PE=2 SV=1 | PREDICTED: CRS2-associated factor 2, chloroplastic [Vitis vinifera] |
| c73698.graph_c0 | -- | -- | -- | -- | -- | -- | ZF-HD protein dimerisation region | Zinc-finger homeodomain protein 1 OS=Oryza sativa subsp. japonica (Rice) PE=2 SV=1 | unnamed protein product [Coffea canephora] |
| c19972.graph_c0 | -- | -- | Molecular Function: protein binding (GO:0005515);; Biological Process: phosphatidylinositol biosynthetic process (GO:0006661);; Biological Process: response to auxin (GO:0009733);; Biological Process: regulation of gene expression, epigenetic (GO:0040029);; | -- | -- | -- | -- | Small acidic protein 1 GN=SMAP1 OS=Arabidopsis thaliana (Mouse-ear cress) PE=1 SV=1 | PREDICTED: small acidic protein 1 [Vitis vinifera] |
| c39287.graph_c0 | [KAD] | -- | Molecular Function: binding (GO:0005488);; Biological Process: cellular process (GO:0009987);; | K09422|4e-96|vvi:100256500|hypothetical protein LOC100256500 | [K] | Transcription | Myb-like DNA-binding domain;; Myb-like DNA-binding domain | Transcription factor MYB59 GN=MYB59 OS=Arabidopsis thaliana (Mouse-ear cress) PE=2 SV=2 | PREDICTED: transcription factor MYB48 [Vitis vinifera] |
| c27614.graph_c0 | -- | -- | Cellular Component: nucleosome (GO:0000786);; Molecular Function: DNA binding (GO:0003677);; Cellular Component: nucleolus (GO:0005730);; Biological Process: nucleosome assembly (GO:0006334);; Biological Process: calcium ion transport (GO:0006816);; Biological Process: Golgi organization (GO:0007030);; Biological Process: response to salt stress (GO:0009651);; Molecular Function: protein heterodimerization activity (GO:0046982);; | K11251|5e-38|vvi:100256203|hypothetical protein LOC100256203 | [B] | Chromatin structure and dynamics | Core histone H2A/H2B/H3/H4 | Histone H2A.1 OS=Solanum lycopersicum (Tomato) PE=2 SV=1 | unnamed protein product [Coffea canephora] |
| c71517.graph_c1 | [CE] | -- | Molecular Function: magnesium ion binding (GO:0000287);; Molecular Function: 3-isopropylmalate dehydrogenase activity (GO:0003862);; Molecular Function: isocitrate dehydrogenase (NAD+) activity (GO:0004449);; Biological Process: tricarboxylic acid cycle (GO:0006099);; Biological Process: leucine biosynthetic process (GO:0009098);; Cellular Component: chloroplast (GO:0009507);; Molecular Function: NAD binding (GO:0051287);; | K00030|7e-97|rcu:RCOM_1355470|isocitrate dehydrogenase, putative (EC:1.1.1.41) | [E] | Amino acid transport and metabolism | Isocitrate/isopropylmalate dehydrogenase | 3-isopropylmalate dehydrogenase, chloroplastic (Precursor) OS=Solanum tuberosum (Potato) PE=1 SV=1 | hypothetical protein CISIN_1g017413mg [Citrus sinensis] |
| c64044.graph_c0 | -- | -- | -- | -- | [R] | General function prediction only | PPR repeat;; PPR repeat family;; PPR repeat;; Pentatricopeptide repeat domain | Pentatricopeptide repeat-containing protein At2g17525, mitochondrial (Precursor) GN=At2g17525 OS=Arabidopsis thaliana (Mouse-ear cress) PE=2 SV=2 | PREDICTED: pentatricopeptide repeat-containing protein At2g17525, mitochondrial [Vitis vinifera] |
| c45369.graph_c0 | [KAD] | -- | -- | K09422|1e-105|vvi:100244605|hypothetical protein LOC100244605 | [K] | Transcription | Myb-like DNA-binding domain;; Myb-like DNA-binding domain | Anthocyanin regulatory C1 protein GN=C1 OS=Zea mays (Maize) PE=2 SV=1 | Duplicated homeodomain-like superfamily protein, putative [Theobroma cacao] |
| c48068.graph_c0 | [G] | Carbohydrate transport and metabolism | -- | -- | -- | -- | -- | Glycine-rich cell wall structural protein (Precursor) GN=At3g17050 OS=Arabidopsis thaliana (Mouse-ear cress) PE=3 SV=2 | PREDICTED: glycine-rich cell wall structural protein [Malus domestica] |
| c75749.graph_c0 | [J] | Translation, ribosomal structure and biogenesis | -- | K01873|0.0|rcu:RCOM_0771810|valyl-tRNA synthetase, putative (EC:6.1.1.9) | [J] | Translation, ribosomal structure and biogenesis | tRNA synthetases class I (I, L, M and V);; Anticodon-binding domain of tRNA;; tRNA synthetases class I (M);; Leucyl-tRNA synthetase, Domain 2;; Valyl tRNA synthetase tRNA binding arm;; tRNA synthetases class I (C) catalytic domain | Valine--tRNA ligase GN=VALRS OS=Arabidopsis thaliana (Mouse-ear cress) PE=2 SV=2 | PREDICTED: valine--tRNA ligase isoform X2 [Vitis vinifera] |
| c75132.graph_c0 | -- | -- | Cellular Component: mitochondrial inner membrane (GO:0005743);; Biological Process: protein targeting to membrane (GO:0006612);; Biological Process: mitochondrial transport (GO:0006839);; Biological Process: nucleotide transport (GO:0006862);; Biological Process: ER to Golgi vesicle-mediated transport (GO:0006888);; Biological Process: membrane fusion (GO:0006944);; Biological Process: nucleotide biosynthetic process (GO:0009165);; Biological Process: embryo development ending in seed dormancy (GO:0009793);; Cellular Component: chloroplast envelope (GO:0009941);; Biological Process: regulation of plant-type hypersensitive response (GO:0010363);; Molecular Function: nucleotide transmembrane transporter activity (GO:0015215);; Biological Process: ammonium transport (GO:0015696);; Biological Process: basic amino acid transport (GO:0015802);; Cellular Component: integral component of membrane (GO:0016021);; Biological Process: negative regulation of programmed cell death (GO:0043069);; Biological Process: amino acid import (GO:0043090);; Biological Process: regulation of ion transport (GO:0043269);; Biological Process: transmembrane transport (GO:0055085);; | -- | [C] | Energy production and conversion | Mitochondrial carrier protein | Adenine nucleotide transporter BT1, chloroplastic/mitochondrial (Precursor) GN=F8B4.100 OS=Arabidopsis thaliana (Mouse-ear cress) PE=1 SV=1 | hypothetical protein VITISV_005135 [Vitis vinifera] |
| c74406.graph_c0 | [DK] | -- | -- | -- | [K] | Transcription | -- | -- | unnamed protein product [Vitis vinifera] |
| c71082.graph_c1 | -- | -- | Cellular Component: integral component of membrane (GO:0016021);; | -- | [GE] | -- | -- | UDP-galactose transporter 2 GN=UDP-GALT2 OS=Arabidopsis thaliana (Mouse-ear cress) PE=2 SV=1 | PREDICTED: UDP-galactose transporter 2-like [Nelumbo nucifera] |
| c67024.graph_c0 | [HE] | -- | Molecular Function: O-phospho-L-serine:2-oxoglutarate aminotransferase activity (GO:0004648);; Biological Process: L-serine biosynthetic process (GO:0006564);; Molecular Function: pyridoxal phosphate binding (GO:0030170);; | K00831|0.0|vvi:100243722|hypothetical protein LOC100243722 | [HE] | -- | Aminotransferase class-V | Phosphoserine aminotransferase 1, chloroplastic (Precursor) GN=PSAT1 OS=Arabidopsis thaliana (Mouse-ear cress) PE=1 SV=1 | PREDICTED: phosphoserine aminotransferase 2, chloroplastic-like [Vitis vinifera] |
| c19714.graph_c0 | -- | -- | Biological Process: calcium ion transport (GO:0006816);; Biological Process: Golgi organization (GO:0007030);; Cellular Component: chloroplast thylakoid membrane (GO:0009535);; Biological Process: response to salt stress (GO:0009651);; Biological Process: photosynthesis, light harvesting (GO:0009765);; Cellular Component: photosystem II antenna complex (GO:0009783);; Cellular Component: chloroplast envelope (GO:0009941);; Biological Process: nonphotochemical quenching (GO:0010196);; Cellular Component: plastoglobule (GO:0010287);; Molecular Function: chlorophyll binding (GO:0016168);; Biological Process: cysteine biosynthetic process (GO:0019344);; Biological Process: regulation of protein dephosphorylation (GO:0035304);; Molecular Function: metal ion binding (GO:0046872);; | K08917|3e-82|vvi:100241887|hypothetical protein LOC100241887 | -- | -- | Chlorophyll A-B binding protein | Chlorophyll a-b binding protein CP24 10A, chloroplastic (Precursor) GN=CAP10A OS=Solanum lycopersicum (Tomato) PE=3 SV=1 | PREDICTED: chlorophyll a-b binding protein CP24 10A, chloroplastic [Nelumbo nucifera] |
| c61446.graph_c0 | [Q] | Secondary metabolites biosynthesis, transport and catabolism | Molecular Function: monooxygenase activity (GO:0004497);; Molecular Function: iron ion binding (GO:0005506);; Molecular Function: electron carrier activity (GO:0009055);; Molecular Function: oxidoreductase activity, acting on paired donors, with incorporation or reduction of molecular oxygen (GO:0016705);; Molecular Function: heme binding (GO:0020037);; Biological Process: oxidation-reduction process (GO:0055114);; | K09588|0.0|pop:POPTR_656234|CYP90A6v1 | [QI] | -- | Cytochrome P450 | Cytochrome P450 90A1 GN=MJJ3.9 OS=Arabidopsis thaliana (Mouse-ear cress) PE=2 SV=1 | cytochrome P450 90A2 [Camellia japonica] |
| c66116.graph_c0 | -- | -- | -- | -- | -- | -- | -- | LOB domain-containing protein 36 GN=MUD21.13 OS=Arabidopsis thaliana (Mouse-ear cress) PE=2 SV=1 | ASYMMETRIC LEAVES 2-like 1 [Theobroma cacao] |
| c57150.graph_c0 | [U] | Intracellular trafficking, secretion, and vesicular transport | Cellular Component: integral component of membrane (GO:0016021);; Biological Process: vesicle-mediated transport (GO:0016192);; | K08516|1e-132|rcu:RCOM_0700610|snare protein ykt6, putative | [U] | Intracellular trafficking, secretion, and vesicular transport | Regulated-SNARE-like domain;; Synaptobrevin | VAMP-like protein YKT61 (Precursor) GN=YKT61 OS=Arabidopsis thaliana (Mouse-ear cress) PE=2 SV=1 | snare protein ykt6, putative [Ricinus communis] |
| c63497.graph_c0 | -- | -- | Molecular Function: cytochrome-c oxidase activity (GO:0004129);; Cellular Component: mitochondrial envelope (GO:0005740);; | K02265|1e-79|rcu:RCOM_0897910|cytochrome C oxidase, putative | [C] | Energy production and conversion | Cytochrome c oxidase subunit Vb | Cytochrome c oxidase subunit 5b-2, mitochondrial (Precursor) GN=COX5B-2 OS=Arabidopsis thaliana (Mouse-ear cress) PE=2 SV=1 | PREDICTED: putative cytochrome c oxidase subunit 5b-like [Nicotiana tomentosiformis] |
| c59228.graph_c0 | -- | -- | Molecular Function: nucleotide binding (GO:0000166);; Molecular Function: nucleic acid binding (GO:0003676);; Biological Process: mRNA processing (GO:0006397);; | K12822|0.0|vvi:100263874|hypothetical protein LOC100263874 | [A] | RNA processing and modification | PWI domain;; RNA recognition motif. (a.k.a. RRM, RBD, or RNP domain);; RNA recognition motif (a.k.a. RRM, RBD, or RNP domain) | 60S ribosomal protein L18a-like protein GN=At1g29970 OS=Arabidopsis thaliana (Mouse-ear cress) PE=2 SV=2 | PREDICTED: RNA-binding protein 25 isoform X3 [Nelumbo nucifera] |
| c74506.graph_c0 | -- | -- | -- | K10727|0.0|vvi:100263560|hypothetical protein LOC100263560 | [L] | Replication, recombination and repair | DNA replication factor CDT1 like | CDT1-like protein a, chloroplastic (Precursor) GN=CDT1A OS=Arabidopsis thaliana (Mouse-ear cress) PE=1 SV=1 | PREDICTED: CDT1-like protein a, chloroplastic [Vitis vinifera] |
| c61544.graph_c0 | -- | -- | Molecular Function: mRNA binding (GO:0003729);; Cellular Component: mRNA cleavage factor complex (GO:0005849);; Biological Process: mRNA polyadenylation (GO:0006378);; Molecular Function: hydrolase activity (GO:0016787);; | K14397|1e-139|vvi:100261950|hypothetical protein LOC100261950 | [A] | RNA processing and modification | Nucleotide hydrolase | Pre-mRNA cleavage factor Im 25 kDa subunit 2 {ECO:0000303|PubMed:18479511} OS=Arabidopsis thaliana (Mouse-ear cress) PE=1 SV=1 | PREDICTED: pre-mRNA cleavage factor Im 25 kDa subunit 2-like [Sesamum indicum] |
| c68887.graph_c0 | -- | -- | Molecular Function: organic anion transmembrane transporter activity (GO:0008514);; Biological Process: plant-type cell wall biogenesis (GO:0009832);; Biological Process: organic anion transport (GO:0015711);; Cellular Component: integral component of membrane (GO:0016021);; Biological Process: cell growth (GO:0016049);; Biological Process: cellulose metabolic process (GO:0030243);; | -- | [GE] | -- | Triose-phosphate Transporter family;; UAA transporter family;; EamA-like transporter family | Probable sugar phosphate/phosphate translocator At1g12500 GN=At1g12500 OS=Arabidopsis thaliana (Mouse-ear cress) PE=1 SV=1 | PREDICTED: probable sugar phosphate/phosphate translocator At1g12500 [Vitis vinifera] |
| c70067.graph_c0 | -- | -- | -- | -- | -- | -- | -- | Nuclear-pore anchor {ECO:0000303|PubMed:17513499, ECO:0000303|PubMed:21189294} OS=Arabidopsis thaliana (Mouse-ear cress) PE=1 SV=1 | PREDICTED: ELKS/Rab6-interacting/CAST family member 1 [Vitis vinifera] |
| c64548.graph_c0 | -- | -- | -- | -- | -- | -- | MYND finger | F-box protein At1g67340 GN=At1g67340 OS=Arabidopsis thaliana (Mouse-ear cress) PE=1 SV=1 | PREDICTED: F-box protein At1g67340 [Nelumbo nucifera] |
| c60422.graph_c1 | -- | -- | -- | -- | [U] | Intracellular trafficking, secretion, and vesicular transport | PRA1 family protein | PRA1 family protein F3 GN=PRA1F3 OS=Arabidopsis thaliana (Mouse-ear cress) PE=1 SV=1 | PREDICTED: PRA1 family protein F2-like [Vitis vinifera] |
| c67468.graph_c0 | -- | -- | -- | -- | -- | -- | SBP domain | Squamosa promoter-binding-like protein 12 GN=SPL12 OS=Oryza sativa subsp. indica (Rice) PE=2 SV=1 | PREDICTED: squamosa promoter-binding-like protein 12 [Sesamum indicum] |
| c73078.graph_c0 | [GEPR] | -- | -- | -- | [R] | General function prediction only | Sugar (and other) transporter;; Major Facilitator Superfamily;; CCT motif;; B-box zinc finger | Sugar carrier protein A GN=STA OS=Ricinus communis (Castor bean) PE=2 SV=1 | hexose transport protein [Actinidia chinensis] |
| c56056.graph_c0 | [R] | General function prediction only | -- | -- | -- | -- | Histone chaperone domain CHZ;; DEK C terminal domain | Nucleolin 2 GN=OSJNBa0058K23.21 OS=Oryza sativa subsp. japonica (Rice) PE=2 SV=2 | PREDICTED: glutamic acid-rich protein [Vitis vinifera] |
| c70652.graph_c0 | [K] | Transcription | -- | -- | [K] | Transcription | Early transcription elongation factor of RNA pol II, NGN section;; KOW motif | Putative transcription elongation factor SPT5 homolog 1 GN=At4g08350 OS=Arabidopsis thaliana (Mouse-ear cress) PE=1 SV=2 | PREDICTED: hornerin isoform X2 [Vitis vinifera] |
| c71061.graph_c0 | [L] | Replication, recombination and repair | Molecular Function: binding (GO:0005488);; | -- | [R] | General function prediction only | Reverse transcriptase (RNA-dependent DNA polymerase) | RNA-directed DNA polymerase homolog OS=Oenothera berteroana (Bertero&apos;s evening primrose) PE=4 SV=1 | hypothetical protein PRUPE_ppa026856mg [Prunus persica] |
| c76562.graph_c0 | -- | -- | -- | -- | [J] | Translation, ribosomal structure and biogenesis | -- | 40S ribosomal protein S9-2 GN=RPS9C OS=Arabidopsis thaliana (Mouse-ear cress) PE=2 SV=1 | Os07g0628300 [Oryza sativa Japonica Group] |
| c65449.graph_c0 | -- | -- | Molecular Function: RNA binding (GO:0003723);; | -- | [AR] | -- | KH domain;; KH domain;; KH domain;; KH domain | KH domain-containing protein At4g18375 GN=At4g18375 OS=Arabidopsis thaliana (Mouse-ear cress) PE=2 SV=1 | PREDICTED: KH domain-containing protein At4g18375 [Vitis vinifera] |
| c76150.graph_c0 | [J] | Translation, ribosomal structure and biogenesis | Molecular Function: tRNA binding (GO:0000049);; Molecular Function: alanine-tRNA ligase activity (GO:0004813);; Molecular Function: ATP binding (GO:0005524);; Cellular Component: mitochondrion (GO:0005739);; Biological Process: alanyl-tRNA aminoacylation (GO:0006419);; Molecular Function: zinc ion binding (GO:0008270);; Cellular Component: chloroplast stroma (GO:0009570);; Biological Process: embryo development ending in seed dormancy (GO:0009793);; Cellular Component: membrane (GO:0016020);; | K01872|0.0|vvi:100255425|hypothetical protein LOC100255425 | [J] | Translation, ribosomal structure and biogenesis | tRNA synthetases class II (A);; Threonyl and Alanyl tRNA synthetase second additional domain;; DHHA1 domain;; tRNA synthetases class II (A);; tRNA synthetases class II (A) | Probable alanine--tRNA ligase, chloroplastic {ECO:0000255|HAMAP-Rule:MF_03134} GN=POPTRDRAFT_821063 OS=Populus trichocarpa (Western balsam poplar) PE=3 SV=1 | hypothetical protein JCGZ_03949 [Jatropha curcas] |
| c73408.graph_c0 | [RTKL] | -- | Molecular Function: protein kinase activity (GO:0004672);; | K08827|0.0|vvi:100247382|hypothetical protein LOC100247382 | [A] | RNA processing and modification | Protein kinase domain;; Protein tyrosine kinase | Serine/threonine-protein kinase AFC3 GN=F4D11.140 OS=Arabidopsis thaliana (Mouse-ear cress) PE=2 SV=2 | PREDICTED: serine/threonine-protein kinase prpf4B-like [Vitis vinifera] |
| c52529.graph_c0 | -- | -- | Biological Process: anatomical structure morphogenesis (GO:0009653);; Biological Process: cellular process (GO:0009987);; Biological Process: single-organism developmental process (GO:0044767);; Biological Process: organ development (GO:0048513);; Biological Process: regulation of biological process (GO:0050789);; | -- | -- | -- | -- | -- | PREDICTED: transcription factor LHW-like isoform X2 [Vitis vinifera] |
| c67737.graph_c0 | -- | -- | Molecular Function: calmodulin binding (GO:0005516);; Biological Process: defense response (GO:0006952);; Biological Process: response to biotic stimulus (GO:0009607);; Cellular Component: integral component of membrane (GO:0016021);; | -- | -- | -- | Mlo family | MLO-like protein 12 GN=MLO12 OS=Arabidopsis thaliana (Mouse-ear cress) PE=2 SV=2 | unnamed protein product [Vitis vinifera] |
| c30890.graph_c0 | -- | -- | -- | -- | [K] | Transcription | -- | -- | hypothetical protein [Oryza sativa Japonica Group] |
| c65725.graph_c0 | -- | -- | Cellular Component: chloroplast (GO:0009507);; Biological Process: photosynthesis, light harvesting (GO:0009765);; Cellular Component: membrane (GO:0016020);; Molecular Function: metal ion binding (GO:0046872);; | K08912|0.0|vvi:100241745|hypothetical protein LOC100241745 | -- | -- | Chlorophyll A-B binding protein | Chlorophyll a-b binding protein 40, chloroplastic (Precursor) GN=CAB40 OS=Nicotiana tabacum (Common tobacco) PE=2 SV=1 | PREDICTED: chlorophyll a-b binding protein of LHCII type 1 [Vitis vinifera] |
| c72248.graph_c0 | [R] | General function prediction only | Molecular Function: nucleotide binding (GO:0000166);; Molecular Function: nucleic acid binding (GO:0003676);; Biological Process: regulation of transcription, DNA-templated (GO:0006355);; Cellular Component: ribonucleoprotein complex (GO:0030529);; Biological Process: posttranscriptional gene silencing by RNA (GO:0035194);; Biological Process: production of small RNA involved in gene silencing by RNA (GO:0070918);; | K11093|1e-170|vvi:100244445|hypothetical protein LOC100244445 | [A] | RNA processing and modification | U1 small nuclear ribonucleoprotein of 70kDa MW N terminal;; RNA recognition motif. (a.k.a. RRM, RBD, or RNP domain) | U1 small nuclear ribonucleoprotein 70 kDa GN=RNU1 OS=Arabidopsis thaliana (Mouse-ear cress) PE=1 SV=1 | PREDICTED: U1 small nuclear ribonucleoprotein 70 kDa [Vitis vinifera] |
| c69122.graph_c0 | -- | -- | -- | -- | -- | -- | Cupin;; Cupin domain | Antimicrobial peptide 2d (Precursor) OS=Macadamia integrifolia (Macadamia nut) PE=2 SV=1 | PREDICTED: vicilin-like antimicrobial peptides 2-2 [Sesamum indicum] |
| c65296.graph_c0 | [S] | Function unknown | Biological Process: aromatic amino acid family biosynthetic process (GO:0009073);; Biological Process: iron-sulfur cluster assembly (GO:0016226);; Biological Process: vitamin B6 biosynthetic process (GO:0042819);; | -- | [S] | Function unknown | Transcriptional regulator | Probable transcriptional regulatory protein At2g25830 GN=At2g25830 OS=Arabidopsis thaliana (Mouse-ear cress) PE=2 SV=2 | PREDICTED: probable transcriptional regulatory protein At2g25830 isoform X1 [Vitis vinifera] |
| c75568.graph_c1 | -- | -- | -- | -- | [TZ] | -- | Formin Homology 2 Domain | Formin-like protein 1 (Precursor) OS=Oryza sativa subsp. japonica (Rice) PE=2 SV=1 | PREDICTED: formin-like protein 1 [Vitis vinifera] |
| c81706.graph_c0 | -- | -- | -- | K13091|1e-06|vvi:100243992|hypothetical protein LOC100243992 | [TV] | -- | -- | -- | predicted protein [Botrytis cinerea B05.10] |
| c54000.graph_c0 | -- | -- | Biological Process: calcium ion transport (GO:0006816);; Biological Process: Golgi organization (GO:0007030);; Cellular Component: chloroplast thylakoid membrane (GO:0009535);; Biological Process: response to salt stress (GO:0009651);; Biological Process: photosynthesis, light harvesting (GO:0009765);; Cellular Component: photosystem II antenna complex (GO:0009783);; Cellular Component: chloroplast envelope (GO:0009941);; Biological Process: nonphotochemical quenching (GO:0010196);; Cellular Component: plastoglobule (GO:0010287);; Cellular Component: integral component of membrane (GO:0016021);; Molecular Function: chlorophyll binding (GO:0016168);; Biological Process: protein-chromophore linkage (GO:0018298);; Biological Process: cysteine biosynthetic process (GO:0019344);; Biological Process: regulation of protein dephosphorylation (GO:0035304);; Molecular Function: metal ion binding (GO:0046872);; | K08917|1e-171|vvi:100241887|hypothetical protein LOC100241887 | -- | -- | Chlorophyll A-B binding protein | Chlorophyll a-b binding protein CP24 10A, chloroplastic (Precursor) GN=CAP10A OS=Solanum lycopersicum (Tomato) PE=3 SV=1 | PREDICTED: chlorophyll a-b binding protein CP24 10A, chloroplastic [Vitis vinifera] |
| c65982.graph_c0 | -- | -- | -- | -- | -- | -- | Protein of unknown function (DUF640) | Protein LIGHT-DEPENDENT SHORT HYPOCOTYLS 6 GN=F10K1.20 OS=Arabidopsis thaliana (Mouse-ear cress) PE=1 SV=1 | hypothetical protein VITISV_019556 [Vitis vinifera] |
| c69827.graph_c0 | -- | -- | -- | -- | -- | -- | SBP domain | Squamosa promoter-binding-like protein 6 GN=SPL6 OS=Arabidopsis thaliana (Mouse-ear cress) PE=2 SV=2 | PREDICTED: squamosa promoter-binding-like protein 6 isoform X2 [Vitis vinifera] |
| c75586.graph_c0 | -- | -- | -- | -- | [K] | Transcription | CCT motif | Zinc finger protein CONSTANS-LIKE 4 GN=COL4 OS=Arabidopsis thaliana (Mouse-ear cress) PE=2 SV=2 | PREDICTED: zinc finger protein CONSTANS-LIKE 5-like [Sesamum indicum] |
| c72104.graph_c0 | [RTKL] | -- | Molecular Function: ATP binding (GO:0005524);; Cellular Component: nucleolus (GO:0005730);; Cellular Component: spindle (GO:0005819);; Molecular Function: histone kinase activity (H3-S10 specific) (GO:0035175);; Biological Process: histone H3-S10 phosphorylation (GO:0043987);; | K08850|1e-148|vvi:100268070|hypothetical protein LOC100268070 | [D] | Cell cycle control, cell division, chromosome partitioning | Protein kinase domain;; Protein tyrosine kinase | Serine/threonine-protein kinase Aurora-1 GN=AUR1 OS=Arabidopsis thaliana (Mouse-ear cress) PE=1 SV=1 | PREDICTED: serine/threonine-protein kinase Aurora-1 [Vitis vinifera] |
| c68001.graph_c0 | -- | -- | Molecular Function: 1-phosphatidylinositol binding (GO:0005545);; Cellular Component: clathrin coat (GO:0030118);; Molecular Function: clathrin binding (GO:0030276);; Molecular Function: 2-alkenal reductase [NAD(P)] activity (GO:0032440);; Biological Process: clathrin coat assembly (GO:0048268);; Biological Process: oxidation-reduction process (GO:0055114);; | -- | [TU] | -- | ANTH domain;; ENTH domain | Putative clathrin assembly protein At2g01600 GN=At2g01600 OS=Arabidopsis thaliana (Mouse-ear cress) PE=2 SV=2 | PREDICTED: putative clathrin assembly protein At2g01600 [Sesamum indicum] |
| c69076.graph_c1 | -- | -- | Molecular Function: methyltransferase activity (GO:0008168);; Biological Process: methylation (GO:0032259);; | -- | -- | -- | Methyltransferase domain;; Methyltransferase domain | -- | PREDICTED: uncharacterized protein LOC104097182 [Nicotiana tomentosiformis] |
| c76161.graph_c1 | -- | -- | Molecular Function: nucleotide binding (GO:0000166);; Molecular Function: nucleic acid binding (GO:0003676);; | -- | [E] | Amino acid transport and metabolism | RNA recognition motif 2;; RNA recognition motif. (a.k.a. RRM, RBD, or RNP domain);; RNA recognition motif. (a.k.a. RRM, RBD, or RNP domain);; RNA recognition motif (a.k.a. RRM, RBD, or RNP domain);; Transmembrane amino acid transporter protein;; Tryptophan/tyrosine permease family;; TCP family transcription factor;; Embryo-specific protein 3, (ATS3) | Protein MEI2-like 4 OS=Oryza sativa subsp. japonica (Rice) PE=2 SV=1 | PREDICTED: protein MEI2-like 4 isoform X3 [Vitis vinifera] |
| c60415.graph_c0 | -- | -- | -- | K03128|0.0|vvi:100266355|hypothetical protein LOC100266355 | [K] | Transcription | Peptidase family M1 | Transcription initiation factor TFIID subunit 2 GN=TAF2 OS=Arabidopsis thaliana (Mouse-ear cress) PE=2 SV=1 | unnamed protein product [Coffea canephora] |
| c64122.graph_c0 | [T] | Signal transduction mechanisms | Molecular Function: protein domain specific binding (GO:0019904);; | K06630|1e-164|rcu:RCOM_1035790|14-3-3 protein, putative | [O] | Posttranslational modification, protein turnover, chaperones | 14-3-3 protein | 14-3-3 protein 7 GN=TFT7 OS=Solanum lycopersicum (Tomato) PE=2 SV=2 | hypothetical protein JCGZ_15794 [Jatropha curcas] |
| c62954.graph_c0 | [O] | Posttranslational modification, protein turnover, chaperones | Molecular Function: threonine-type endopeptidase activity (GO:0004298);; Cellular Component: proteasome core complex (GO:0005839);; Biological Process: proteolysis involved in cellular protein catabolic process (GO:0051603);; | K02734|1e-123|vvi:100240752|hypothetical protein LOC100240752 | [O] | Posttranslational modification, protein turnover, chaperones | Proteasome subunit | Proteasome subunit beta type-2-A GN=F16J14.18 OS=Arabidopsis thaliana (Mouse-ear cress) PE=1 SV=1 | PREDICTED: proteasome subunit beta type-2-A [Nicotiana tomentosiformis] |
| c62873.graph_c0 | -- | -- | Molecular Function: DNA binding (GO:0003677);; Molecular Function: sequence-specific DNA binding transcription factor activity (GO:0003700);; Cellular Component: nucleus (GO:0005634);; Biological Process: regulation of transcription, DNA-templated (GO:0006355);; Biological Process: pattern specification process (GO:0007389);; Biological Process: auxin-activated signaling pathway (GO:0009734);; Biological Process: abscisic acid-activated signaling pathway (GO:0009738);; Biological Process: response to carbohydrate (GO:0009743);; Biological Process: fruit development (GO:0010154);; Biological Process: regulation of anthocyanin biosynthetic process (GO:0031540);; Molecular Function: miRNA binding (GO:0035198);; Molecular Function: protein dimerization activity (GO:0046983);; Biological Process: leaf development (GO:0048366);; Biological Process: petal development (GO:0048441);; Biological Process: sepal development (GO:0048442);; Biological Process: developmental growth (GO:0048589);; Biological Process: root cap development (GO:0048829);; Biological Process: cell division (GO:0051301);; | -- | -- | -- | Auxin response factor;; B3 DNA binding domain;; AUX/IAA family | Auxin response factor 18 OS=Oryza sativa subsp. japonica (Rice) PE=2 SV=1 | PREDICTED: auxin response factor 18 [Vitis vinifera] |
| c71850.graph_c0 | [RTKL] | -- | Biological Process: cytokinesis by cell plate formation (GO:0000911);; Molecular Function: protein serine/threonine kinase activity (GO:0004674);; Molecular Function: ATP binding (GO:0005524);; Biological Process: protein phosphorylation (GO:0006468);; Biological Process: transmembrane receptor protein tyrosine kinase signaling pathway (GO:0007169);; | -- | -- | -- | Protein kinase domain;; Protein tyrosine kinase;; Leucine rich repeat;; Leucine Rich repeats (2 copies);; Leucine Rich Repeat;; Leucine rich repeat | Probable inactive receptor kinase At5g67200 (Precursor) GN=At5g67200 OS=Arabidopsis thaliana (Mouse-ear cress) PE=1 SV=1 | PREDICTED: probable inactive receptor kinase At5g67200 [Vitis vinifera] |
| c103546.graph_c0 | -- | -- | -- | -- | -- | -- | -- | -- | aminotransferase [Populus trichocarpa] |
| c25572.graph_c0 | -- | -- | -- | -- | -- | -- | -- | Formin-like protein 3 OS=Oryza sativa subsp. japonica (Rice) PE=2 SV=2 | PREDICTED: uncharacterized protein LOC100260477 [Vitis vinifera] |
| c59123.graph_c0 | [P] | Inorganic ion transport and metabolism | Molecular Function: heme binding (GO:0020037);; Molecular Function: ferredoxin-nitrite reductase activity (GO:0048307);; Molecular Function: iron-sulfur cluster binding (GO:0051536);; Biological Process: oxidation-reduction process (GO:0055114);; | K00366|0.0|vvi:100253671|hypothetical protein LOC100253671 | [P] | Inorganic ion transport and metabolism | Nitrite and sulphite reductase 4Fe-4S domain;; Nitrite/Sulfite reductase ferredoxin-like half domain | Ferredoxin--nitrite reductase, chloroplastic (Precursor) GN=NIR1 OS=Betula pendula (European white birch) PE=2 SV=1 | PREDICTED: ferredoxin--nitrite reductase, chloroplastic [Nelumbo nucifera] |
| c70689.graph_c0 | [R] | General function prediction only | Molecular Function: methyltransferase activity (GO:0008168);; Biological Process: methylation (GO:0032259);; Cellular Component: intracellular organelle (GO:0043229);; | -- | [BK] | -- | SET domain;; Pre-SET motif | Histone-lysine N-methyltransferase SUVR3 GN=F20H23.22 OS=Arabidopsis thaliana (Mouse-ear cress) PE=2 SV=3 | PREDICTED: histone-lysine N-methyltransferase SUVR3 [Vitis vinifera] |
| c74422.graph_c1 | -- | -- | Biological Process: protein targeting to vacuole (GO:0006623);; Biological Process: vesicle-mediated transport (GO:0016192);; | -- | [R] | General function prediction only | Domain of unknown function (DUF814) | -- | PREDICTED: coiled-coil domain-containing protein 25 [Sesamum indicum] |
| c70767.graph_c0 | -- | -- | -- | -- | -- | -- | -- | -- | PREDICTED: uncharacterized protein LOC102604157 isoform X1 [Solanum tuberosum] |
| c74917.graph_c0 | -- | -- | Biological Process: vegetative to reproductive phase transition of meristem (GO:0010228);; Biological Process: protein desumoylation (GO:0016926);; Biological Process: hydrogen peroxide biosynthetic process (GO:0050665);; | -- | -- | -- | Plant protein of unknown function (DUF639);; Plant protein of unknown function (DUF639);; Plant protein of unknown function (DUF639) | -- | PREDICTED: uncharacterized protein LOC103321118 [Prunus mume] |
| c71575.graph_c0 | [R] | General function prediction only | Biological Process: flavonoid biosynthetic process (GO:0009813);; Molecular Function: oxidoreductase activity, acting on single donors with incorporation of molecular oxygen, incorporation of two atoms of oxygen (GO:0016702);; Molecular Function: L-ascorbic acid binding (GO:0031418);; Molecular Function: naringenin 3-dioxygenase activity (GO:0045486);; Molecular Function: metal ion binding (GO:0046872);; Biological Process: oxidation-reduction process (GO:0055114);; | K00475|0.0|vvi:100233079|F3H, FHT | [QR] | -- | non-haem dioxygenase in morphine synthesis N-terminal;; 2OG-Fe(II) oxygenase superfamily | Flavanone 3-dioxygenase GN=FHT OS=Petroselinum crispum (Parsley) PE=1 SV=1 | flavanone 3-hydroxylase [Camellia nitidissima] |
| c63573.graph_c0 | [C] | Energy production and conversion | Cellular Component: chloroplast thylakoid membrane (GO:0009535);; Cellular Component: chloroplast envelope (GO:0009941);; Biological Process: vitamin E biosynthetic process (GO:0010189);; Biological Process: chlorophyll biosynthetic process (GO:0015995);; Molecular Function: oxidoreductase activity, acting on the CH-CH group of donors, NAD or NADP as acceptor (GO:0016628);; Biological Process: cysteine biosynthetic process (GO:0019344);; Biological Process: photosynthesis, light reaction (GO:0019684);; Biological Process: geranylgeranyl diphosphate metabolic process (GO:0033385);; Biological Process: phytyl diphosphate biosynthetic process (GO:0033521);; Biological Process: regulation of protein dephosphorylation (GO:0035304);; Molecular Function: geranylgeranyl reductase activity (GO:0045550);; Biological Process: oxidation-reduction process (GO:0055114);; | K10960|3e-76|vvi:100243277|hypothetical protein LOC100243277 | -- | -- | Pyridine nucleotide-disulphide oxidoreductase;; Glucose inhibited division protein A;; FAD binding domain | Geranylgeranyl diphosphate reductase, chloroplastic (Precursor) GN=CHLP OS=Nicotiana tabacum (Common tobacco) PE=2 SV=1 | geranylgeranyl diphosphate reductase [Camellia sinensis] |
| c67041.graph_c0 | -- | -- | -- | -- | -- | -- | Protein of unknown function (DUF707);; Senescence regulator | -- | PREDICTED: uncharacterized protein LOC104591513 [Nelumbo nucifera] |
| c67758.graph_c0 | [O] | Posttranslational modification, protein turnover, chaperones | Molecular Function: ATP binding (GO:0005524);; Cellular Component: cell wall (GO:0005618);; Cellular Component: nucleus (GO:0005634);; Cellular Component: cytosol (GO:0005829);; Biological Process: gluconeogenesis (GO:0006094);; Biological Process: ATP catabolic process (GO:0006200);; Biological Process: fatty acid beta-oxidation (GO:0006635);; Biological Process: ER to Golgi vesicle-mediated transport (GO:0006888);; Biological Process: cytoskeleton organization (GO:0007010);; Molecular Function: peptidase activity (GO:0008233);; Cellular Component: proteasome regulatory particle, base subcomplex (GO:0008540);; Molecular Function: microtubule-severing ATPase activity (GO:0008568);; Biological Process: toxin catabolic process (GO:0009407);; Cellular Component: plasmodesma (GO:0009506);; Biological Process: response to cytokinin (GO:0009735);; Biological Process: photorespiration (GO:0009853);; Cellular Component: membrane (GO:0016020);; Biological Process: amino acid import (GO:0043090);; Biological Process: proteasome-mediated ubiquitin-dependent protein catabolic process (GO:0043161);; Biological Process: root hair elongation (GO:0048767);; Biological Process: response to misfolded protein (GO:0051788);; Biological Process: proteasome core complex assembly (GO:0080129);; | -- | [O] | Posttranslational modification, protein turnover, chaperones | ATPase family associated with various cellular activities (AAA);; AAA domain (dynein-related subfamily);; AAA domain (Cdc48 subfamily);; AAA ATPase domain;; Protein of unknown function (DUF815);; Holliday junction DNA helicase ruvB N-terminus | 26S protease regulatory subunit 6B homolog {ECO:0000250|UniProtKB:P54778} OS=Helianthus annuus (Common sunflower) PE=1 SV=1 | PREDICTED: 26S protease regulatory subunit 6B homolog [Nelumbo nucifera] |
| c29491.graph_c0 | [T] | Signal transduction mechanisms | Biological Process: response to molecule of fungal origin (GO:0002238);; Cellular Component: plasma membrane (GO:0005886);; Biological Process: protein targeting to vacuole (GO:0006623);; Biological Process: response to cold (GO:0009409);; | -- | -- | -- | Universal stress protein family | Universal stress protein A-like protein GN=At3g01520 OS=Arabidopsis thaliana (Mouse-ear cress) PE=1 SV=2 | conserved hypothetical protein [Ricinus communis] |
| c73456.graph_c1 | [G] | Carbohydrate transport and metabolism | Molecular Function: fructose-bisphosphate aldolase activity (GO:0004332);; Biological Process: glycolysis (GO:0006096);; | K01623|0.0|rcu:RCOM_0577600|fructose-bisphosphate aldolase, putative (EC:4.1.2.13) | [G] | Carbohydrate transport and metabolism | Fructose-bisphosphate aldolase class-I | Probable fructose-bisphosphate aldolase 3, chloroplastic (Precursor) GN=FBA3 OS=Arabidopsis thaliana (Mouse-ear cress) PE=1 SV=1 | fructose-bisphosphate aldolase [Camellia oleifera] |
| c71046.graph_c0 | [J] | Translation, ribosomal structure and biogenesis | Molecular Function: translation initiation factor activity (GO:0003743);; Biological Process: translational initiation (GO:0006413);; | K03238|1e-130|vvi:100263785|hypothetical protein LOC100263785 | [J] | Translation, ribosomal structure and biogenesis | Domain found in IF2B/IF5;; Rer1 family | Eukaryotic translation initiation factor 2 subunit beta OS=Malus domestica (Apple) PE=2 SV=2 | unnamed protein product [Vitis vinifera] |
| c67278.graph_c0 | -- | -- | -- | -- | -- | -- | -- | -- | PREDICTED: uncharacterized protein YGR130C [Nicotiana tomentosiformis] |
| c62600.graph_c0 | -- | -- | -- | -- | [G] | Carbohydrate transport and metabolism | -- | -- | PREDICTED: uncharacterized protein LOC100255271 [Vitis vinifera] |
| c46843.graph_c0 | -- | -- | Molecular Function: transferase activity, transferring phosphorus-containing groups (GO:0016772);; Biological Process: primary metabolic process (GO:0044238);; Biological Process: cellular macromolecule metabolic process (GO:0044260);; Molecular Function: organic cyclic compound binding (GO:0097159);; Molecular Function: heterocyclic compound binding (GO:1901363);; | -- | -- | -- | -- | Protein Ycf2 {ECO:0000255|HAMAP-Rule:MF_01330} OS=Oenothera parviflora (Small-flowered evening primrose) PE=3 SV=1 | hypothetical protein MTR_5g032980 [Medicago truncatula] |
| c74751.graph_c0 | [PR] | -- | Molecular Function: iron ion binding (GO:0005506);; Molecular Function: electron carrier activity (GO:0009055);; Molecular Function: oxidoreductase activity, acting on paired donors, with incorporation or reduction of molecular oxygen, NAD(P)H as one donor, and incorporation of two atoms of oxygen into one donor (GO:0016708);; Biological Process: aromatic compound catabolic process (GO:0019439);; Molecular Function: 2 iron, 2 sulfur cluster binding (GO:0051537);; Biological Process: oxidation-reduction process (GO:0055114);; | -- | -- | -- | Ring hydroxylating alpha subunit (catalytic domain);; Rieske [2Fe-2S] domain | Choline monooxygenase, chloroplastic (Precursor) GN=At4g29890 OS=Arabidopsis thaliana (Mouse-ear cress) PE=2 SV=2 | choline monooxygenase [Camellia sinensis] |
| c68715.graph_c0 | [R] | General function prediction only | Molecular Function: nucleotide binding (GO:0000166);; Biological Process: double-strand break repair via homologous recombination (GO:0000724);; Molecular Function: nucleic acid binding (GO:0003676);; Biological Process: mitotic recombination (GO:0006312);; Cellular Component: chloroplast (GO:0009507);; Biological Process: embryo sac egg cell differentiation (GO:0009560);; Biological Process: response to ionizing radiation (GO:0010212);; Biological Process: post-translational protein modification (GO:0043687);; Biological Process: positive regulation of transcription, DNA-templated (GO:0045893);; | K12840|1e-145|vvi:100265303|hypothetical protein LOC100265303 | [A] | RNA processing and modification | WWE domain;; G-patch domain | DNA-damage-repair/toleration protein DRT111, chloroplastic (Precursor) GN=DRT111 OS=Arabidopsis thaliana (Mouse-ear cress) PE=1 SV=2 | DNA-damage-repair/toleration protein [Morus notabilis] |
| c57152.graph_c0 | [OC] | -- | Molecular Function: protein disulfide isomerase activity (GO:0003756);; Molecular Function: dolichyl-diphosphooligosaccharide-protein glycotransferase activity (GO:0004579);; Cellular Component: mitochondrion (GO:0005739);; Cellular Component: vacuolar membrane (GO:0005774);; Cellular Component: endoplasmic reticulum (GO:0005783);; Cellular Component: plasma membrane (GO:0005886);; Biological Process: protein folding (GO:0006457);; Biological Process: N-terminal protein myristoylation (GO:0006499);; Biological Process: glycerol ether metabolic process (GO:0006662);; Biological Process: response to oxidative stress (GO:0006979);; Molecular Function: electron carrier activity (GO:0009055);; Cellular Component: chloroplast (GO:0009507);; Molecular Function: protein disulfide oxidoreductase activity (GO:0015035);; Biological Process: cellulose biosynthetic process (GO:0030244);; Biological Process: cell redox homeostasis (GO:0045454);; Biological Process: Golgi vesicle transport (GO:0048193);; | K09580|0.0|vvi:100246809|hypothetical protein LOC100246809 | [O] | Posttranslational modification, protein turnover, chaperones | Thioredoxin;; Thioredoxin-like domain;; Thioredoxin-like;; Thioredoxin-like;; Thioredoxin-like domain;; AhpC/TSA family;; ERp29, N-terminal domain | Protein disulfide isomerase-like 1-4 (Precursor) GN=MUP24.6 OS=Arabidopsis thaliana (Mouse-ear cress) PE=1 SV=1 | unnamed protein product [Coffea canephora] |
| c70152.graph_c0 | [E] | Amino acid transport and metabolism | Molecular Function: N2-acetyl-L-ornithine:2-oxoglutarate 5-aminotransferase activity (GO:0003992);; Molecular Function: copper ion binding (GO:0005507);; Cellular Component: mitochondrion (GO:0005739);; Biological Process: arginine biosynthetic process (GO:0006526);; Cellular Component: chloroplast stroma (GO:0009570);; Molecular Function: pyridoxal phosphate binding (GO:0030170);; Biological Process: defense response to bacterium (GO:0042742);; Biological Process: interspecies interaction between organisms (GO:0044419);; Biological Process: ovule development (GO:0048481);; Biological Process: primary root development (GO:0080022);; | K00818|0.0|vvi:100264460|hypothetical protein LOC100264460 | [E] | Amino acid transport and metabolism | Aminotransferase class-III | Acetylornithine aminotransferase, mitochondrial (Precursor) GN=AG118 OS=Alnus glutinosa (European alder) PE=2 SV=1 | PREDICTED: acetylornithine aminotransferase, mitochondrial [Vitis vinifera] |
| c69172.graph_c1 | -- | -- | -- | -- | -- | -- | F-box domain;; Cold acclimation protein WCOR413 | F-box protein At5g39450 GN=At5g39450 OS=Arabidopsis thaliana (Mouse-ear cress) PE=2 SV=1 | PREDICTED: F-box protein At5g39450 isoform X2 [Vitis vinifera] |
| c73161.graph_c0 | -- | -- | Biological Process: single-organism process (GO:0044699);; | -- | [S] | Function unknown | Trafficking protein Mon1;; Trafficking protein Mon1 | -- | PREDICTED: protein SAND isoform X3 [Nelumbo nucifera] |
| c62395.graph_c0 | [R] | General function prediction only | -- | K09553|0.0|pop:POPTR_837084|hypothetical protein | [O] | Posttranslational modification, protein turnover, chaperones | TPR repeat;; Tetratricopeptide repeat;; Tetratricopeptide repeat;; Tetratricopeptide repeat;; Tetratricopeptide repeat;; Tetratricopeptide repeat;; Tetratricopeptide repeat;; Tetratricopeptide repeat;; Tetratricopeptide repeat;; Tetratricopeptide repeat;; Tetratricopeptide repeat;; Tetratricopeptide repeat;; Anaphase-promoting complex, cyclosome, subunit 3;; MIT (microtubule interacting and transport) domain;; Fis1 C-terminal tetratricopeptide repeat | Hsp70-Hsp90 organizing protein 1 GN=HOP1 OS=Arabidopsis thaliana (Mouse-ear cress) PE=2 SV=1 | hypothetical protein JCGZ_20777 [Jatropha curcas] |
| c63135.graph_c0 | [M] | Cell wall/membrane/envelope biogenesis | Molecular Function: UDP-N-acetylglucosamine 1-carboxyvinyltransferase activity (GO:0008760);; Biological Process: UDP-N-acetylgalactosamine biosynthetic process (GO:0019277);; | -- | -- | -- | EPSP synthase (3-phosphoshikimate 1-carboxyvinyltransferase);; EPSP synthase (3-phosphoshikimate 1-carboxyvinyltransferase) | -- | PREDICTED: uncharacterized protein LOC100262376 isoform X1 [Vitis vinifera] |
| c36931.graph_c0 | -- | -- | Biological Process: cell communication (GO:0007154);; Biological Process: cellular metabolic process (GO:0044237);; Biological Process: single organism signaling (GO:0044700);; Biological Process: single-organism metabolic process (GO:0044710);; Biological Process: organonitrogen compound metabolic process (GO:1901564);; | -- | -- | -- | Rapid ALkalinization Factor (RALF) | Protein RALF-like 33 (Precursor) OS=Arabidopsis thaliana (Mouse-ear cress) PE=2 SV=1 | RALF-LIKE 23 family protein [Populus trichocarpa] |
| c71613.graph_c0 | -- | -- | -- | -- | -- | -- | Late embryogenesis abundant protein | Protein YLS9 GN=F11F19.11 OS=Arabidopsis thaliana (Mouse-ear cress) PE=2 SV=1 | PREDICTED: protein YLS9 [Nicotiana tomentosiformis] |
| c67408.graph_c0 | -- | -- | -- | -- | [R] | General function prediction only | -- | -- | PREDICTED: uncharacterized protein LOC100257639 [Vitis vinifera] |
| c75798.graph_c0 | [Q] | Secondary metabolites biosynthesis, transport and catabolism | Molecular Function: copper ion binding (GO:0005507);; Molecular Function: primary amine oxidase activity (GO:0008131);; Biological Process: amine metabolic process (GO:0009308);; Molecular Function: quinone binding (GO:0048038);; Biological Process: oxidation-reduction process (GO:0055114);; | -- | [Q] | Secondary metabolites biosynthesis, transport and catabolism | Copper amine oxidase, enzyme domain;; Copper amine oxidase, N3 domain;; Copper amine oxidase, N2 domain | Primary amine oxidase (Precursor) GN=At1g62810 OS=Arabidopsis thaliana (Mouse-ear cress) PE=2 SV=1 | PREDICTED: primary amine oxidase-like [Sesamum indicum] |
| c31689.graph_c0 | -- | -- | -- | -- | -- | -- | Zinc-binding;; 4F5 protein family | Uncharacterized protein At2g23090 GN=At2g23090 OS=Arabidopsis thaliana (Mouse-ear cress) PE=1 SV=1 | PREDICTED: uncharacterized protein At2g23090-like [Populus euphratica] |
| c68352.graph_c0 | [J] | Translation, ribosomal structure and biogenesis | Cellular Component: cytoplasm (GO:0005737);; Biological Process: peptidyl-diphthamide biosynthetic process from peptidyl-histidine (GO:0017183);; | -- | [J] | Translation, ribosomal structure and biogenesis | Putative diphthamide synthesis protein | Proline-rich receptor-like protein kinase PERK2 GN=PERK2 OS=Arabidopsis thaliana (Mouse-ear cress) PE=2 SV=3 | PREDICTED: diphthamide biosynthesis protein 1 [Nelumbo nucifera] |
| c76005.graph_c2 | -- | -- | Molecular Function: binding (GO:0005488);; | -- | -- | -- | Protein of unknown function (DUF 659);; hAT family C-terminal dimerisation region | -- | PREDICTED: uncharacterized protein LOC104901267 [Beta vulgaris subsp. vulgaris] |
| c19971.graph_c0 | -- | -- | -- | -- | -- | -- | Domain of unknown function (DUF4408) | -- | PREDICTED: LOW QUALITY PROTEIN: uncharacterized protein LOC105173623 [Sesamum indicum] |
| c22570.graph_c0 | -- | -- | Molecular Function: N-acetyltransferase activity (GO:0008080);; Cellular Component: chloroplast (GO:0009507);; Molecular Function: tyramine N-feruloyltransferase activity (GO:0050366);; | -- | [E] | Amino acid transport and metabolism | -- | Probable acetyltransferase NATA1-like GN=At2g39020 OS=Arabidopsis thaliana (Mouse-ear cress) PE=2 SV=1 | PREDICTED: probable acetyltransferase NATA1-like [Glycine max] |
| c66163.graph_c0 | -- | -- | -- | -- | -- | -- | Hydrophobic seed protein;; Protease inhibitor/seed storage/LTP family;; Probable lipid transfer | 36.4 kDa proline-rich protein GN=TPRP-F1 OS=Solanum lycopersicum (Tomato) PE=2 SV=1 | putative proline-rich cell wall protein [Prunus dulcis] |
| c55881.graph_c0 | [J] | Translation, ribosomal structure and biogenesis | Molecular Function: RNA binding (GO:0003723);; Molecular Function: structural constituent of ribosome (GO:0003735);; Cellular Component: ribosome (GO:0005840);; Biological Process: pentose-phosphate shunt (GO:0006098);; Biological Process: rRNA processing (GO:0006364);; Biological Process: translation (GO:0006412);; Cellular Component: chloroplast stroma (GO:0009570);; Cellular Component: thylakoid (GO:0009579);; Biological Process: photosynthetic electron transport in photosystem I (GO:0009773);; Biological Process: chloroplast relocation (GO:0009902);; Biological Process: thylakoid membrane organization (GO:0010027);; Biological Process: chlorophyll biosynthetic process (GO:0015995);; Cellular Component: membrane (GO:0016020);; Biological Process: isopentenyl diphosphate biosynthetic process, methylerythritol 4-phosphate pathway (GO:0019288);; | K02945|0.0|vvi:100265325|hypothetical protein LOC100265325 | -- | -- | S1 RNA binding domain | 30S ribosomal protein S1, chloroplastic (Precursor) GN=RPS1 OS=Spinacia oleracea (Spinach) PE=1 SV=1 | PREDICTED: 30S ribosomal protein S1, chloroplastic [Nelumbo nucifera] |
| c71911.graph_c1 | [I] | Lipid transport and metabolism | Molecular Function: iron ion binding (GO:0005506);; Biological Process: fatty acid biosynthetic process (GO:0006633);; Cellular Component: integral component of membrane (GO:0016021);; Molecular Function: oxidoreductase activity (GO:0016491);; Biological Process: oxidation-reduction process (GO:0055114);; | K14424|1e-105|vvi:100260328|hypothetical protein LOC100260328 | [I] | Lipid transport and metabolism | hTAFII28-like protein conserved region | Methylsterol monooxygenase 2-2 GN=F22G5.23 OS=Arabidopsis thaliana (Mouse-ear cress) PE=2 SV=1 | unnamed protein product [Coffea canephora] |
| c73260.graph_c0 | -- | -- | -- | -- | -- | -- | -- | -- | hypothetical protein L484_027744 [Morus notabilis] |
| c75775.graph_c0 | -- | -- | Cellular Component: integral component of membrane (GO:0016021);; | -- | [R] | General function prediction only | TMPIT-like protein;; Ring finger domain;; RING-H2 zinc finger;; Zinc finger, C3HC4 type (RING finger);; Zinc finger, C3HC4 type (RING finger);; Zinc finger, C3HC4 type (RING finger) | E3 ubiquitin-protein ligase ATL23 GN=ATL23 OS=Arabidopsis thaliana (Mouse-ear cress) PE=1 SV=2 | unnamed protein product [Coffea canephora] |
| c70437.graph_c0 | [R] | General function prediction only | Cellular Component: plasma membrane (GO:0005886);; Biological Process: nitrogen compound metabolic process (GO:0006807);; Molecular Function: cyanoalanine nitrilase activity (GO:0047427);; | K13035|0.0|vvi:100250098|similar to Bifunctional nitrilase/nitrile hydratase NIT4B | [E] | Amino acid transport and metabolism | Carbon-nitrogen hydrolase | Bifunctional nitrilase/nitrile hydratase NIT4A GN=NIT4A OS=Nicotiana tabacum (Common tobacco) PE=2 SV=1 | nitrilase [Camellia sinensis] |
| c72874.graph_c3 | [O] | Posttranslational modification, protein turnover, chaperones | Molecular Function: transferase activity (GO:0016740);; | -- | [O] | Posttranslational modification, protein turnover, chaperones | Glutathione S-transferase, N-terminal domain;; Glutathione S-transferase, N-terminal domain;; Glutathione S-transferase, C-terminal domain;; Glutathione S-transferase, N-terminal domain;; Glutathione S-transferase, C-terminal domain;; Glutathione S-transferase, C-terminal domain | Probable glutathione S-transferase OS=Nicotiana tabacum (Common tobacco) PE=2 SV=1 | PREDICTED: probable glutathione S-transferase parC [Prunus mume] |
| c72718.graph_c0 | -- | -- | -- | -- | -- | -- | PWWP domain | Uncharacterized protein At1g51745 GN=At1g51745 OS=Arabidopsis thaliana (Mouse-ear cress) PE=2 SV=2 | PREDICTED: uncharacterized protein At1g51745 isoform X1 [Vitis vinifera] |
| c71179.graph_c0 | -- | -- | Molecular Function: phosphatidylinositol phospholipase C activity (GO:0004435);; Molecular Function: signal transducer activity (GO:0004871);; Molecular Function: calcium ion binding (GO:0005509);; Biological Process: lipid metabolic process (GO:0006629);; Biological Process: intracellular signal transduction (GO:0035556);; | K05857|0.0|pop:POPTR_803315|phosphoinositide phospholipase C (EC:3.1.4.11) | [T] | Signal transduction mechanisms | Phosphatidylinositol-specific phospholipase C, X domain;; Phosphoinositide-specific phospholipase C, efhand-like;; Phosphatidylinositol-specific phospholipase C, Y domain;; C2 domain | Phosphoinositide phospholipase C 6 GN=PLC6 OS=Arabidopsis thaliana (Mouse-ear cress) PE=2 SV=2 | unnamed protein product [Coffea canephora] |
| c65503.graph_c0 | -- | -- | Biological Process: microtubule cytoskeleton organization (GO:0000226);; Biological Process: cytokinesis (GO:0000910);; Molecular Function: microtubule binding (GO:0008017);; | -- | [DZ] | -- | Microtubule associated protein (MAP65/ASE1 family) | 65-kDa microtubule-associated protein 6 GN=MAP65-6 OS=Arabidopsis thaliana (Mouse-ear cress) PE=1 SV=1 | PLE, putative [Ricinus communis] |
| c73092.graph_c0 | -- | -- | -- | -- | -- | -- | FBD | F-box/LRR-repeat protein At4g14103 GN=At4g14103 OS=Arabidopsis thaliana (Mouse-ear cress) PE=2 SV=1 | PREDICTED: F-box/LRR-repeat protein At4g14103-like isoform X2 [Vitis vinifera] |
| c63577.graph_c0 | -- | -- | -- | -- | -- | -- | Protein of unknown function (DUF581) | -- | hypothetical protein L484_008024 [Morus notabilis] |
| c65994.graph_c0 | [RTKL] | -- | Molecular Function: calmodulin-dependent protein kinase activity (GO:0004683);; Molecular Function: calcium ion binding (GO:0005509);; Molecular Function: ATP binding (GO:0005524);; Biological Process: protein phosphorylation (GO:0006468);; Biological Process: abscisic acid-activated signaling pathway (GO:0009738);; | K13412|0.0|pop:POPTR_826066|CPK12 | [T] | Signal transduction mechanisms | Protein kinase domain;; EF hand;; EF-hand domain pair;; Protein tyrosine kinase;; EF-hand domain;; EF hand;; EF-hand domain pair;; Cytoskeletal-regulatory complex EF hand | Calcium-dependent protein kinase SK5 OS=Glycine max (Soybean) PE=1 SV=1 | PREDICTED: calcium-dependent protein kinase SK5-like [Solanum tuberosum] |
| c61241.graph_c0 | -- | -- | -- | -- | -- | -- | -- | F-box protein At2g27310 GN=At2g27310 OS=Arabidopsis thaliana (Mouse-ear cress) PE=2 SV=1 | PREDICTED: F-box protein At2g27310 [Vitis vinifera] |
| c66985.graph_c0 | [R] | General function prediction only | Biological Process: cytokinesis (GO:0000910);; Molecular Function: GTP binding (GO:0005525);; Cellular Component: endosome (GO:0005768);; Cellular Component: cytosol (GO:0005829);; Cellular Component: plasma membrane (GO:0005886);; Biological Process: small GTPase mediated signal transduction (GO:0007264);; Cellular Component: cell plate (GO:0009504);; Biological Process: protein transport (GO:0015031);; Biological Process: vesicle-mediated transport (GO:0016192);; Molecular Function: hydrolase activity (GO:0016787);; Biological Process: second-messenger-mediated signaling (GO:0019932);; | -- | [U] | Intracellular trafficking, secretion, and vesicular transport | Ras family;; Miro-like protein;; ADP-ribosylation factor family;; 50S ribosome-binding GTPase;; Elongation factor Tu GTP binding domain | Ras-related protein RABA2a (Precursor) GN=F21M12.2 OS=Arabidopsis thaliana (Mouse-ear cress) PE=2 SV=1 | protein with unknown function [Ricinus communis] |
| c76407.graph_c0 | -- | -- | Cellular Component: plasma membrane (GO:0005886);; Biological Process: response to auxin (GO:0009733);; Biological Process: response to aluminum ion (GO:0010044);; | -- | -- | -- | Aluminium induced protein;; Glutamine amidotransferase domain;; Glutamine amidotransferase domain | Stem-specific protein TSJT1 GN=TSJT1 OS=Nicotiana tabacum (Common tobacco) PE=2 SV=1 | PREDICTED: stem-specific protein TSJT1 [Nelumbo nucifera] |
| c73534.graph_c0 | -- | -- | Biological Process: phenylpropanoid metabolic process (GO:0009698);; | -- | -- | -- | -- | Mediator of RNA polymerase II transcription subunit 33A GN=At3g23590 OS=Arabidopsis thaliana (Mouse-ear cress) PE=1 SV=1 | hypothetical protein glysoja_007263 [Glycine soja] |
| c55551.graph_c0 | -- | -- | Biological Process: defense response to virus (GO:0051607);; | -- | -- | -- | Late embryogenesis abundant protein | Protein YLS9 GN=F11F19.11 OS=Arabidopsis thaliana (Mouse-ear cress) PE=2 SV=1 | PREDICTED: protein YLS9-like [Populus euphratica] |
| c56017.graph_c0 | -- | -- | Cellular Component: mitochondrial respiratory chain complex I (GO:0005747);; | K03966|3e-61|pop:POPTR_823292|hypothetical protein | [C] | Energy production and conversion | NADH-ubiquinone oxidoreductase subunit 10 | NADH dehydrogenase [ubiquinone] 1 beta subcomplex subunit 10-B GN=At3g18410 OS=Arabidopsis thaliana (Mouse-ear cress) PE=2 SV=1 | unnamed protein product [Coffea canephora] |
| c74647.graph_c1 | -- | -- | -- | -- | -- | -- | Leucine Rich repeats (2 copies);; Leucine Rich Repeat;; Leucine Rich repeat;; Leucine rich repeat;; Leucine rich repeat | Polygalacturonase inhibitor {ECO:0000250|UniProtKB:P58822} (Precursor) GN=pgip OS=Vitis vinifera (Grape) PE=1 SV=1 | polygalacturonase inhibitor [Actinidia deliciosa] |
| c72721.graph_c0 | -- | -- | -- | -- | [R] | General function prediction only | Las1-like | -- | PREDICTED: pre-rRNA-processing protein las1 [Vitis vinifera] |
| c71112.graph_c0 | -- | -- | Molecular Function: monooxygenase activity (GO:0004497);; Molecular Function: oxidoreductase activity, acting on paired donors, with incorporation or reduction of molecular oxygen (GO:0016705);; Molecular Function: metal ion binding (GO:0046872);; | -- | [QI] | -- | -- | Beta-amyrin 28-oxidase OS=Panax ginseng (Korean ginseng) PE=2 SV=1 | CYP716A75 [Maesa lanceolata] |
| c72309.graph_c0 | -- | -- | -- | K11984|0.0|vvi:100266959|hypothetical protein LOC100266959 | [A] | RNA processing and modification | SART-1 family | SART-1 family protein DOT2 {ECO:0000305} OS=Arabidopsis thaliana (Mouse-ear cress) PE=1 SV=1 | PREDICTED: SART-1 family protein DOT2 [Vitis vinifera] |
| c62685.graph_c0 | -- | -- | Molecular Function: methyltransferase activity (GO:0008168);; Biological Process: methylation (GO:0032259);; | -- | -- | -- | Methyltransferase domain;; Methyltransferase domain;; ubiE/COQ5 methyltransferase family;; Methyltransferase domain;; Methyltransferase domain;; Methyltransferase domain;; Methyltransferase domain | Uncharacterized methyltransferase At2g41040, chloroplastic (Precursor) GN=At2g41040 OS=Arabidopsis thaliana (Mouse-ear cress) PE=2 SV=1 | PREDICTED: uncharacterized methyltransferase At2g41040, chloroplastic [Sesamum indicum] |
| c60075.graph_c1 | -- | -- | Biological Process: retrograde vesicle-mediated transport, Golgi to ER (GO:0006890);; Cellular Component: integral component of membrane (GO:0016021);; Biological Process: maintenance of protein location (GO:0045185);; | -- | [U] | Intracellular trafficking, secretion, and vesicular transport | Rer1 family | Protein RER1A GN=RER1A OS=Arabidopsis thaliana (Mouse-ear cress) PE=1 SV=1 | PREDICTED: protein RER1A-like [Fragaria vesca subsp. vesca] |
| c62315.graph_c0 | -- | -- | -- | -- | [O] | Posttranslational modification, protein turnover, chaperones | Ring finger domain;; RING-H2 zinc finger | RING-H2 finger protein ATL60 GN=ATL60 OS=Arabidopsis thaliana (Mouse-ear cress) PE=2 SV=1 | PREDICTED: RING-H2 finger protein ATL64-like [Vitis vinifera] |
| c54245.graph_c0 | -- | -- | Cellular Component: endoplasmic reticulum (GO:0005783);; | -- | [S] | Function unknown | Domain of unknown function (DUF4149) | -- | uncharacterized protein LOC100305973 [Glycine max] |
| c70911.graph_c0 | -- | -- | Cellular Component: cytosol (GO:0005829);; Biological Process: protein targeting to vacuole (GO:0006623);; Biological Process: ER to Golgi vesicle-mediated transport (GO:0006888);; Cellular Component: extrinsic component of endoplasmic reticulum membrane (GO:0042406);; | -- | [UD] | -- | RINT-1 / TIP-1 family | RINT1-like protein MAG2 {ECO:0000305} OS=Arabidopsis thaliana (Mouse-ear cress) PE=1 SV=1 | PREDICTED: RINT1-like protein MAG2 [Vitis vinifera] |
| c27331.graph_c0 | [Q] | Secondary metabolites biosynthesis, transport and catabolism | Biological Process: RNA splicing, via endonucleolytic cleavage and ligation (GO:0000394);; Molecular Function: iron ion binding (GO:0005506);; Cellular Component: endoplasmic reticulum (GO:0005783);; Cellular Component: Golgi apparatus (GO:0005794);; Cellular Component: plasma membrane (GO:0005886);; Molecular Function: methyltransferase activity (GO:0008168);; Molecular Function: sterol 14-demethylase activity (GO:0008398);; Molecular Function: electron carrier activity (GO:0009055);; Biological Process: methionine biosynthetic process (GO:0009086);; Biological Process: embryo development ending in seed dormancy (GO:0009793);; Biological Process: sterol biosynthetic process (GO:0016126);; Biological Process: pentacyclic triterpenoid biosynthetic process (GO:0019745);; Molecular Function: oxygen binding (GO:0019825);; Molecular Function: heme binding (GO:0020037);; Biological Process: methylation (GO:0032259);; Biological Process: oxidation-reduction process (GO:0055114);; | K05917|0.0|vvi:100242104|hypothetical protein LOC100242104 | [Q] | Secondary metabolites biosynthesis, transport and catabolism | Cytochrome P450 | Sterol 14-demethylase GN=F25C20.17 OS=Arabidopsis thaliana (Mouse-ear cress) PE=1 SV=1 | PREDICTED: sterol 14-demethylase [Nicotiana tomentosiformis] |
| c72456.graph_c0 | -- | -- | Biological Process: chloroplast organization (GO:0009658);; Cellular Component: Tic complex (GO:0031897);; Biological Process: protein import into chloroplast stroma (GO:0045037);; | -- | -- | -- | -- | Protein TIC110, chloroplastic (Precursor) GN=TIC110 OS=Pisum sativum (Garden pea) PE=1 SV=1 | PREDICTED: protein TIC110, chloroplastic [Vitis vinifera] |
| c75240.graph_c0 | [Q] | Secondary metabolites biosynthesis, transport and catabolism | Molecular Function: monooxygenase activity (GO:0004497);; Molecular Function: iron ion binding (GO:0005506);; Molecular Function: electron carrier activity (GO:0009055);; Molecular Function: oxidoreductase activity, acting on paired donors, with incorporation or reduction of molecular oxygen (GO:0016705);; Molecular Function: heme binding (GO:0020037);; Biological Process: oxidation-reduction process (GO:0055114);; | -- | [QI] | -- | Cytochrome P450 | Cytochrome P450 CYP72A219 OS=Panax ginseng (Korean ginseng) PE=2 SV=1 | cytochrome P450 monooxygenase [Medicago truncatula] |
| c57266.graph_c0 | -- | -- | Molecular Function: nucleotide binding (GO:0000166);; Biological Process: mRNA splicing, via spliceosome (GO:0000398);; Molecular Function: snRNA binding (GO:0017069);; | -- | -- | -- | -- | Protein MATERNALLY EXPRESSED GENE 5 GN=MEG5 OS=Zea mays (Maize) PE=2 SV=1 | PREDICTED: uncharacterized protein LOC100257637 isoform X2 [Vitis vinifera] |
| c22166.graph_c0 | -- | -- | Molecular Function: nucleic acid binding (GO:0003676);; | -- | [R] | General function prediction only | Reverse transcriptase (RNA-dependent DNA polymerase);; Endonuclease/Exonuclease/phosphatase family;; Endonuclease-reverse transcriptase | Uncharacterized mitochondrial protein AtMg01250 GN=AtMg01250 OS=Arabidopsis thaliana (Mouse-ear cress) PE=4 SV=1 | hypothetical protein VITISV_039410 [Vitis vinifera] |
| c66134.graph_c0 | -- | -- | Cellular Component: nucleus (GO:0005634);; Biological Process: regulation of transcription, DNA-templated (GO:0006355);; Biological Process: response to abscisic acid (GO:0009737);; | K11644|1e-57|vvi:100254234|hypothetical protein LOC100254234 | [B] | Chromatin structure and dynamics | -- | Paired amphipathic helix protein Sin3-like 3 GN=F3I6.12 OS=Arabidopsis thaliana (Mouse-ear cress) PE=1 SV=3 | PREDICTED: paired amphipathic helix protein Sin3-like 2 isoform X1 [Vitis vinifera] |
| c63935.graph_c0 | -- | -- | Molecular Function: enzyme inhibitor activity (GO:0004857);; Molecular Function: pectinesterase activity (GO:0030599);; Biological Process: negative regulation of catalytic activity (GO:0043086);; | -- | -- | -- | Plant invertase/pectin methylesterase inhibitor | 21 kDa protein (Precursor) OS=Daucus carota (Wild carrot) PE=2 SV=1 | Plant invertase/pectin methylesterase inhibitor superfamily protein [Theobroma cacao] |
| c70409.graph_c0 | [J] | Translation, ribosomal structure and biogenesis | Molecular Function: structural constituent of ribosome (GO:0003735);; Biological Process: translation (GO:0006412);; Cellular Component: large ribosomal subunit (GO:0015934);; | -- | [J] | Translation, ribosomal structure and biogenesis | Ras-induced vulval development antagonist;; Ribosomal protein L18e/L15 | 50S ribosomal protein L15, chloroplastic (Precursor) OS=Arabidopsis thaliana (Mouse-ear cress) PE=2 SV=2 | Uncharacterized protein TCM_004343 [Theobroma cacao] |
| c73156.graph_c0 | [E] | Amino acid transport and metabolism | -- | -- | [R] | General function prediction only | GMC oxidoreductase;; GMC oxidoreductase | Protein HOTHEAD (Precursor) GN=F3N23.17 OS=Arabidopsis thaliana (Mouse-ear cress) PE=1 SV=1 | PREDICTED: protein HOTHEAD-like isoform X1 [Nicotiana tomentosiformis] |
| c73009.graph_c0 | -- | -- | Molecular Function: ATP transmembrane transporter activity (GO:0005347);; Cellular Component: mitochondrion (GO:0005739);; Cellular Component: plasma membrane (GO:0005886);; Biological Process: aerobic respiration (GO:0009060);; Cellular Component: plastid (GO:0009536);; Molecular Function: ADP transmembrane transporter activity (GO:0015217);; Biological Process: ADP transport (GO:0015866);; Biological Process: ATP transport (GO:0015867);; Cellular Component: integral component of membrane (GO:0016021);; Biological Process: transmembrane transport (GO:0055085);; Biological Process: AMP transport (GO:0080121);; Molecular Function: AMP transmembrane transporter activity (GO:0080122);; | -- | [C] | Energy production and conversion | Mitochondrial carrier protein | Mitochondrial adenine nucleotide transporter ADNT1 OS=Arabidopsis thaliana (Mouse-ear cress) PE=2 SV=1 | mitochondrial substrate carrier family protein [Populus trichocarpa] |
| c69894.graph_c0 | [O] | Posttranslational modification, protein turnover, chaperones | Biological Process: glutathione metabolic process (GO:0006749);; Cellular Component: chloroplast (GO:0009507);; Biological Process: de-etiolation (GO:0009704);; Molecular Function: transferase activity (GO:0016740);; Biological Process: response to cadmium ion (GO:0046686);; Biological Process: lateral root development (GO:0048527);; Biological Process: response to growth hormone (GO:0060416);; Biological Process: response to karrikin (GO:0080167);; | -- | [O] | Posttranslational modification, protein turnover, chaperones | Glutathione S-transferase, N-terminal domain;; Glutathione S-transferase, N-terminal domain;; Glutathione S-transferase, C-terminal domain;; Glutathione S-transferase, C-terminal domain;; Glutathione S-transferase, N-terminal domain;; B-box zinc finger | Glutathione S-transferase U17 GN=At1g10370 OS=Arabidopsis thaliana (Mouse-ear cress) PE=2 SV=1 | PREDICTED: glutathione S-transferase U17-like [Malus domestica] |
| c68480.graph_c1 | [J] | Translation, ribosomal structure and biogenesis | Molecular Function: structural constituent of ribosome (GO:0003735);; Cellular Component: plasma membrane (GO:0005886);; Biological Process: translation (GO:0006412);; Cellular Component: cytosolic large ribosomal subunit (GO:0022625);; | -- | [J] | Translation, ribosomal structure and biogenesis | Ribosomal L18ae/LX protein domain | 60S ribosomal protein L18a GN=RPL18A OS=Castanea sativa (Sweet chestnut) PE=2 SV=1 | putative 60S ribosomal protein L18a [Gardenia jasminoides] |
| c73430.graph_c0 | [O] | Posttranslational modification, protein turnover, chaperones | Molecular Function: ATP binding (GO:0005524);; Cellular Component: cell wall (GO:0005618);; Cellular Component: nucleolus (GO:0005730);; Cellular Component: cytoplasm (GO:0005737);; Cellular Component: plasma membrane (GO:0005886);; Biological Process: proteolysis (GO:0006508);; Molecular Function: peptidase activity (GO:0008233);; Cellular Component: proteasome regulatory particle, base subcomplex (GO:0008540);; Molecular Function: microtubule-severing ATPase activity (GO:0008568);; Biological Process: protein catabolic process (GO:0030163);; | K03064|0.0|pop:POPTR_643066|hypothetical protein | [O] | Posttranslational modification, protein turnover, chaperones | AFG1-like ATPase;; ATPase family associated with various cellular activities (AAA);; AAA ATPase domain;; AAA domain;; Holliday junction DNA helicase ruvB N-terminus;; AAA domain (Cdc48 subfamily);; AAA domain (dynein-related subfamily) | 26S protease regulatory subunit S10B homolog B GN=RPT4B OS=Arabidopsis thaliana (Mouse-ear cress) PE=1 SV=1 | PREDICTED: 26S protease regulatory subunit S10B homolog B-like [Populus euphratica] |
| c20209.graph_c0 | -- | -- | -- | K09284|2e-11|vvi:100246815|hypothetical protein LOC100246815 | -- | -- | -- | -- | PREDICTED: ethylene-responsive transcription factor RAP2-7-like isoform X2 [Nelumbo nucifera] |
| c71241.graph_c0 | -- | -- | -- | -- | -- | -- | -- | -- | hypothetical protein SORBIDRAFT_06g022210 [Sorghum bicolor] |
| c74328.graph_c1 | [R] | General function prediction only | -- | -- | -- | -- | von Willebrand factor type A domain;; von Willebrand factor type A domain;; von Willebrand factor type A domain;; Ring finger domain;; Zinc finger, C3HC4 type (RING finger);; pfkB family carbohydrate kinase;; pfkB family carbohydrate kinase | -- | PREDICTED: uncharacterized protein LOC100245763 [Vitis vinifera] |
| c46856.graph_c0 | -- | -- | -- | -- | -- | -- | -- | -- | hypothetical protein POPTR_0019s12020g [Populus trichocarpa] |
| c42940.graph_c0 | [O] | Posttranslational modification, protein turnover, chaperones | -- | -- | [O] | Posttranslational modification, protein turnover, chaperones | DnaJ domain | Chaperone protein dnaJ 20, chloroplastic (Precursor) GN=F18A5.220 OS=Arabidopsis thaliana (Mouse-ear cress) PE=1 SV=2 | PREDICTED: chaperone protein dnaJ 20, chloroplastic-like [Nicotiana tomentosiformis] |
| c70668.graph_c1 | -- | -- | -- | -- | -- | -- | -- | Protein IQ-DOMAIN 32 GN=IQD32 OS=Arabidopsis thaliana (Mouse-ear cress) PE=1 SV=3 | PREDICTED: protein IQ-DOMAIN 32 isoform X1 [Vitis vinifera] |
| c66222.graph_c0 | [H] | Coenzyme transport and metabolism | Cellular Component: mitochondrion (GO:0005739);; Cellular Component: chloroplast inner membrane (GO:0009706);; Cellular Component: magnesium chelatase complex (GO:0010007);; Biological Process: chlorophyll biosynthetic process (GO:0015995);; Molecular Function: magnesium chelatase activity (GO:0016851);; Biological Process: photosynthesis, light reaction (GO:0019684);; | K03403|0.0|vvi:100258964|CHLH | -- | -- | CobN/Magnesium Chelatase;; Domain of unknown function (DUF3479) | Magnesium-chelatase subunit ChlH, chloroplastic (Precursor) GN=At5g13630 OS=Arabidopsis thaliana (Mouse-ear cress) PE=1 SV=1 | magnesium chelatase H subunit [Camellia sinensis] |
| c54398.graph_c0 | -- | -- | -- | -- | -- | -- | -- | -- | Uncharacterized protein TCM_009475 [Theobroma cacao] |
| c73251.graph_c0 | -- | -- | -- | -- | -- | -- | Calmodulin binding protein-like | -- | unnamed protein product [Vitis vinifera] |
| c59557.graph_c0 | -- | -- | -- | -- | [R] | General function prediction only | Putative serine esterase (DUF676);; Alpha/beta hydrolase family | -- | PREDICTED: putative lipase YDR444W isoform X1 [Vitis vinifera] |
| c75469.graph_c2 | -- | -- | -- | -- | -- | -- | -- | Formin-like protein 14 (Precursor) GN=P0668H12.11 OS=Oryza sativa subsp. japonica (Rice) PE=2 SV=1 | unnamed protein product [Coffea canephora] |
| c64193.graph_c0 | [J] | Translation, ribosomal structure and biogenesis | Cellular Component: plastid large ribosomal subunit (GO:0000311);; Molecular Function: structural constituent of ribosome (GO:0003735);; Biological Process: translation (GO:0006412);; Cellular Component: chloroplast stroma (GO:0009570);; Cellular Component: chloroplast envelope (GO:0009941);; Biological Process: isopentenyl diphosphate biosynthetic process, methylerythritol 4-phosphate pathway (GO:0019288);; Biological Process: ribosome biogenesis (GO:0042254);; | K02939|1e-103|vvi:100251697|hypothetical protein LOC100251697 | [J] | Translation, ribosomal structure and biogenesis | Ribosomal protein L9, C-terminal domain;; Ribosomal protein L9, N-terminal domain | 50S ribosomal protein L9, chloroplastic (Precursor) GN=RPL9 OS=Arabidopsis thaliana (Mouse-ear cress) PE=2 SV=1 | PREDICTED: 50S ribosomal protein L9, chloroplastic [Eucalyptus grandis] |
| c60672.graph_c0 | -- | -- | -- | -- | -- | -- | Late embryogenesis abundant (LEA) group 1 | Protein LE25 GN=LE25 OS=Solanum lycopersicum (Tomato) PE=2 SV=1 | PREDICTED: 18 kDa seed maturation protein-like [Nicotiana sylvestris] |
| c75683.graph_c4 | -- | -- | Molecular Function: phosphoenolpyruvate carboxykinase (ATP) activity (GO:0004612);; Molecular Function: ATP binding (GO:0005524);; Biological Process: gluconeogenesis (GO:0006094);; Molecular Function: kinase activity (GO:0016301);; Biological Process: phosphorylation (GO:0016310);; | K01610|1e-136|pop:POPTR_816402|phosphoenolpyruvate carboxykinase (ATP) (EC:4.1.1.49) | -- | -- | Phosphoenolpyruvate carboxykinase;; Bacterial PH domain | Phosphoenolpyruvate carboxykinase [ATP] GN=PCKA OS=Arabidopsis thaliana (Mouse-ear cress) PE=1 SV=1 | Phosphoenolpyruvate carboxylase [Morus notabilis] |
| c73080.graph_c0 | [O] | Posttranslational modification, protein turnover, chaperones | Cellular Component: nucleus (GO:0005634);; Biological Process: negative regulation of transcription from RNA polymerase III promoter (GO:0016480);; | -- | [K] | Transcription | Maf1 regulator | -- | PREDICTED: repressor of RNA polymerase III transcription MAF1 homolog isoform X1 [Nicotiana sylvestris] |
| c75689.graph_c0 | [R] | General function prediction only | -- | K14005|0.0|vvi:100263684|hypothetical protein LOC100263684 | [U] | Intracellular trafficking, secretion, and vesicular transport | WD domain, G-beta repeat;; Sec23-binding domain of Sec16;; Steroid receptor RNA activator (SRA1) | Protein transport protein SEC31 homolog B {ECO:0000305} OS=Arabidopsis thaliana (Mouse-ear cress) PE=1 SV=1 | PREDICTED: protein transport protein SEC31 homolog B isoform X2 [Vitis vinifera] |
| c70150.graph_c0 | [S] | Function unknown | Cellular Component: cytosol (GO:0005829);; Biological Process: response to toxic substance (GO:0009636);; Biological Process: response to high light intensity (GO:0009644);; Biological Process: response to hydrogen peroxide (GO:0042542);; Molecular Function: D-aminoacyl-tRNA deacylase activity (GO:0051499);; | -- | -- | -- | D-aminoacyl-tRNA deacylase | D-aminoacyl-tRNA deacylase GN=GEK1 OS=Arabidopsis thaliana (Mouse-ear cress) PE=1 SV=2 | PREDICTED: D-aminoacyl-tRNA deacylase-like isoform X2 [Nelumbo nucifera] |
| c43172.graph_c0 | -- | -- | -- | -- | -- | -- | -- | Protein DEHYDRATION-INDUCED 19 homolog 3 GN=DI19-3 OS=Arabidopsis thaliana (Mouse-ear cress) PE=1 SV=1 | Drought-responsive family protein isoform 2 [Theobroma cacao] |
| c74367.graph_c0 | -- | -- | -- | -- | -- | -- | -- | -- | PREDICTED: uncharacterized protein LOC100243848 isoform X2 [Vitis vinifera] |
| c70693.graph_c2 | [G] | Carbohydrate transport and metabolism | Molecular Function: glyceraldehyde-3-phosphate dehydrogenase (NAD+) (phosphorylating) activity (GO:0004365);; Molecular Function: copper ion binding (GO:0005507);; Biological Process: glucose metabolic process (GO:0006006);; Molecular Function: zinc ion binding (GO:0008270);; Biological Process: pollen development (GO:0009555);; Cellular Component: membrane (GO:0016020);; Biological Process: anther wall tapetum development (GO:0048658);; Molecular Function: NADP binding (GO:0050661);; Molecular Function: NAD binding (GO:0051287);; Biological Process: oxidation-reduction process (GO:0055114);; Biological Process: primary root development (GO:0080022);; Biological Process: amino acid homeostasis (GO:0080144);; | K00134|0.0|rcu:RCOM_1574030|glyceraldehyde 3-phosphate dehydrogenase, putative (EC:1.2.1.12) | [G] | Carbohydrate transport and metabolism | Glyceraldehyde 3-phosphate dehydrogenase, C-terminal domain;; Glyceraldehyde 3-phosphate dehydrogenase, NAD binding domain | Glyceraldehyde-3-phosphate dehydrogenase GAPCP1, chloroplastic (Precursor) GN=GAPCP1 OS=Arabidopsis thaliana (Mouse-ear cress) PE=1 SV=1 | PREDICTED: glyceraldehyde-3-phosphate dehydrogenase GAPCP2, chloroplastic-like [Sesamum indicum] |
| c75377.graph_c0 | [R] | General function prediction only | Molecular Function: GTPase activity (GO:0003924);; Molecular Function: thiamine-phosphate diphosphorylase activity (GO:0004789);; Molecular Function: GTP binding (GO:0005525);; Biological Process: GTP catabolic process (GO:0006184);; Biological Process: rRNA processing (GO:0006364);; Biological Process: thiamine biosynthetic process (GO:0009228);; Biological Process: embryo development ending in seed dormancy (GO:0009793);; Biological Process: chloroplast relocation (GO:0009902);; Biological Process: thylakoid membrane organization (GO:0010027);; Biological Process: photosystem II assembly (GO:0010207);; Biological Process: vegetative to reproductive phase transition of meristem (GO:0010228);; Biological Process: iron-sulfur cluster assembly (GO:0016226);; Cellular Component: chloroplast membrane (GO:0031969);; Biological Process: regulation of protein dephosphorylation (GO:0035304);; Biological Process: transcription from plastid promoter (GO:0042793);; Biological Process: positive regulation of transcription, DNA-templated (GO:0045893);; Biological Process: ovule development (GO:0048481);; | -- | [O] | Posttranslational modification, protein turnover, chaperones | PPR repeat family;; PPR repeat;; Pentatricopeptide repeat domain;; PPR repeat;; Dynamin family;; 50S ribosome-binding GTPase;; Elongation factor Tu GTP binding domain;; Thiamine monophosphate synthase/TENI | Pentatricopeptide repeat-containing protein At5g61370, mitochondrial (Precursor) GN=At5g61370 OS=Arabidopsis thaliana (Mouse-ear cress) PE=2 SV=1 | PREDICTED: uncharacterized protein LOC100256371 [Vitis vinifera] |
| c65689.graph_c0 | [O] | Posttranslational modification, protein turnover, chaperones | Molecular Function: glutathione transferase activity (GO:0004364);; Biological Process: auxin-activated signaling pathway (GO:0009734);; | -- | [O] | Posttranslational modification, protein turnover, chaperones | Glutathione S-transferase, N-terminal domain;; Glutathione S-transferase, N-terminal domain;; Glutathione S-transferase, C-terminal domain;; Glutathione S-transferase, C-terminal domain;; Glutathione S-transferase, N-terminal domain | Probable glutathione S-transferase OS=Nicotiana tabacum (Common tobacco) PE=2 SV=1 | glutathione S-transferase [Camellia sinensis] |
| c69609.graph_c0 | -- | -- | Biological Process: cell proliferation (GO:0008283);; Biological Process: leaf development (GO:0048366);; | -- | [K] | Transcription | SSXT protein (N-terminal region) | GRF1-interacting factor 1 GN=F4I4.20 OS=Arabidopsis thaliana (Mouse-ear cress) PE=1 SV=1 | SSXT family protein isoform 1 [Theobroma cacao] |
| c41676.graph_c0 | -- | -- | -- | -- | -- | -- | -- | Probable LRR receptor-like serine/threonine-protein kinase At1g53440 (Precursor) GN=At1g53440 OS=Arabidopsis thaliana (Mouse-ear cress) PE=2 SV=2 | Contains a weak similarity to disease resistance protein (cf-5) gene from Lycopersicon esculentum gb|AF053993 and contains multiple leucine rich PF|00560 repeats and protein kinase PF|00069 domain. EST gb|T04455 comes from this gene [Arabidopsis thaliana] |
| c66616.graph_c0 | [Q] | Secondary metabolites biosynthesis, transport and catabolism | Molecular Function: calmodulin binding (GO:0005516);; Biological Process: defense response (GO:0006952);; Biological Process: response to biotic stimulus (GO:0009607);; Cellular Component: integral component of membrane (GO:0016021);; | -- | -- | -- | Mlo family;; Cytochrome P450 | MLO-like protein 6 GN=MLO6 OS=Arabidopsis thaliana (Mouse-ear cress) PE=2 SV=2 | MLO-like protein 6 [Morus notabilis] |
| c20373.graph_c0 | -- | -- | Molecular Function: catalytic activity (GO:0003824);; Biological Process: metabolic process (GO:0008152);; | -- | -- | -- | Complex 1 protein (LYR family);; Complex1_LYR-like | -- | PREDICTED: mitochondrial zinc maintenance protein 1, mitochondrial-like [Solanum tuberosum] |
| c41254.graph_c0 | [CI] | -- | Cellular Component: chloroplast outer membrane (GO:0009707);; Cellular Component: stromule (GO:0010319);; Molecular Function: heme binding (GO:0020037);; Molecular Function: metal ion binding (GO:0046872);; | -- | [C] | Energy production and conversion | Cytochrome b5-like Heme/Steroid binding domain | Cytochrome b5 isoform A {ECO:0000303|PubMed:19054355} OS=Arabidopsis thaliana (Mouse-ear cress) PE=1 SV=1 | hypothetical protein F383_27157 [Gossypium arboreum] |
| c57232.graph_c0 | [E] | Amino acid transport and metabolism | Molecular Function: asparagine synthase (glutamine-hydrolyzing) activity (GO:0004066);; Molecular Function: ATP binding (GO:0005524);; Biological Process: L-asparagine biosynthetic process (GO:0070981);; | K01953|0.0|vvi:100240866|hypothetical protein LOC100240866 | [E] | Amino acid transport and metabolism | Asparagine synthase;; Glutamine amidotransferase domain;; Glutamine amidotransferase domain;; Aluminium induced protein;; Glutamine amidotransferases class-II | Asparagine synthetase [glutamine-hydrolyzing] 3 OS=Arabidopsis thaliana (Mouse-ear cress) PE=1 SV=1 | Asparagine synthetase 3 isoform 3 [Theobroma cacao] |
| c61945.graph_c0 | -- | -- | -- | -- | -- | -- | Protein of unknown function (DUF1218) | -- | hypothetical protein CICLE_v10009639mg [Citrus clementina] |
| c58783.graph_c0 | -- | -- | Cellular Component: nucleus (GO:0005634);; Molecular Function: abscisic acid binding (GO:0010427);; | K14496|1e-108|pop:POPTR_664102|hypothetical protein | -- | -- | Polyketide cyclase / dehydrase and lipid transport | Abscisic acid receptor PYL9 GN=F6F3.16 OS=Arabidopsis thaliana (Mouse-ear cress) PE=1 SV=1 | PREDICTED: abscisic acid receptor PYL9-like [Fragaria vesca subsp. vesca] |
| c63440.graph_c0 | -- | -- | Biological Process: polysaccharide biosynthetic process (GO:0000271);; Cellular Component: vacuole (GO:0005773);; Molecular Function: carbon-monoxide oxygenase activity (GO:0008805);; Cellular Component: chloroplast (GO:0009507);; Biological Process: multidimensional cell growth (GO:0009825);; Biological Process: cell tip growth (GO:0009932);; Biological Process: regulation of hormone levels (GO:0010817);; Cellular Component: integral component of membrane (GO:0016021);; Biological Process: cysteine biosynthetic process (GO:0019344);; Biological Process: anthocyanin accumulation in tissues in response to UV light (GO:0043481);; Biological Process: root hair elongation (GO:0048767);; Biological Process: cell wall organization (GO:0071555);; | K08360|1e-130|vvi:100255839|hypothetical protein LOC100255839 | [C] | Energy production and conversion | Eukaryotic cytochrome b561 | Transmembrane ascorbate ferrireductase 1 GN=At4g25570 OS=Arabidopsis thaliana (Mouse-ear cress) PE=1 SV=1 | PREDICTED: transmembrane ascorbate ferrireductase 1 [Sesamum indicum] |
| c45628.graph_c0 | [C] | Energy production and conversion | Biological Process: malate metabolic process (GO:0006108);; Cellular Component: chloroplast (GO:0009507);; Molecular Function: L-malate dehydrogenase activity (GO:0030060);; Biological Process: cellular carbohydrate metabolic process (GO:0044262);; Biological Process: oxidation-reduction process (GO:0055114);; | K00026|0.0|pop:POPTR_583880|malate dehydrogenase (EC:1.1.1.37) | [C] | Energy production and conversion | lactate/malate dehydrogenase, NAD binding domain;; lactate/malate dehydrogenase, alpha/beta C-terminal domain;; 3-beta hydroxysteroid dehydrogenase/isomerase family | Malate dehydrogenase, chloroplastic (Precursor) GN=At3g47520 OS=Arabidopsis thaliana (Mouse-ear cress) PE=1 SV=1 | PREDICTED: malate dehydrogenase, chloroplastic-like [Solanum tuberosum] |
| c61194.graph_c0 | -- | -- | -- | -- | -- | -- | Membrane bound O-acyl transferase family;; MBOAT, membrane-bound O-acyltransferase family | Acyl-CoA--sterol O-acyltransferase 1 GN=ASAT1 OS=Arabidopsis thaliana (Mouse-ear cress) PE=1 SV=1 | PREDICTED: acyl-CoA--sterol O-acyltransferase 1 [Pyrus x bretschneideri] |
| c64443.graph_c0 | [R] | General function prediction only | Molecular Function: nucleotide binding (GO:0000166);; Biological Process: mRNA splicing, via spliceosome (GO:0000398);; Cellular Component: nucleosome (GO:0000786);; Molecular Function: DNA binding (GO:0003677);; Molecular Function: RNA binding (GO:0003723);; Biological Process: sister chromatid cohesion (GO:0007062);; Biological Process: synapsis (GO:0007129);; Biological Process: reciprocal meiotic recombination (GO:0007131);; Biological Process: spermatogenesis (GO:0007283);; Cellular Component: nuclear speck (GO:0016607);; Biological Process: regulation of chromosome organization (GO:0033044);; Biological Process: meiotic DNA double-strand break formation (GO:0042138);; Biological Process: meiotic chromosome segregation (GO:0045132);; | -- | [R] | General function prediction only | -- | Serine/arginine-rich SC35-like splicing factor SCL28 GN=SCL28 OS=Arabidopsis thaliana (Mouse-ear cress) PE=1 SV=1 | unnamed protein product [Coffea canephora] |
| c73026.graph_c0 | [Q] | Secondary metabolites biosynthesis, transport and catabolism | -- | -- | [Q] | Secondary metabolites biosynthesis, transport and catabolism | Cytochrome P450;; Cytochrome P450 | Cytochrome P450 71D10 GN=CYP71D10 OS=Glycine max (Soybean) PE=2 SV=1 | Cytochrome P450 71D10, putative [Theobroma cacao] |
| c41682.graph_c0 | -- | -- | Molecular Function: ATP-dependent peptidase activity (GO:0004176);; Molecular Function: metalloendopeptidase activity (GO:0004222);; Molecular Function: serine-type endopeptidase activity (GO:0004252);; Molecular Function: ATP binding (GO:0005524);; Cellular Component: mitochondrion (GO:0005739);; Biological Process: ATP catabolic process (GO:0006200);; Biological Process: proteolysis (GO:0006508);; Molecular Function: microtubule-severing ATPase activity (GO:0008568);; Cellular Component: plastid (GO:0009536);; Cellular Component: membrane (GO:0016020);; Biological Process: protein catabolic process (GO:0030163);; | -- | [O] | Posttranslational modification, protein turnover, chaperones | -- | ATP-dependent zinc metalloprotease FTSH 4, mitochondrial (Precursor) GN=FTSH4 OS=Arabidopsis thaliana (Mouse-ear cress) PE=1 SV=2 | ATP-dependent zinc metalloprotease FTSH 4 [Morus notabilis] |
| c73807.graph_c0 | [GEPR] | -- | Molecular Function: sugar:hydrogen symporter activity (GO:0005351);; Cellular Component: integral component of membrane (GO:0016021);; Molecular Function: 2-alkenal reductase [NAD(P)] activity (GO:0032440);; Biological Process: carbohydrate transmembrane transport (GO:0034219);; Biological Process: oxidation-reduction process (GO:0055114);; | -- | [R] | General function prediction only | Sugar (and other) transporter;; Major Facilitator Superfamily | Hexose carrier protein HEX6 GN=HEX6 OS=Ricinus communis (Castor bean) PE=2 SV=1 | hypothetical protein POPTR_0016s12820g [Populus trichocarpa] |
| c71640.graph_c0 | [O] | Posttranslational modification, protein turnover, chaperones | Molecular Function: ATP binding (GO:0005524);; Cellular Component: endoplasmic reticulum lumen (GO:0005788);; Biological Process: protein folding (GO:0006457);; Biological Process: response to heat (GO:0009408);; Biological Process: response to high light intensity (GO:0009644);; Biological Process: response to endoplasmic reticulum stress (GO:0034976);; Biological Process: response to hydrogen peroxide (GO:0042542);; | -- | [O] | Posttranslational modification, protein turnover, chaperones | Hsp70 protein;; Hsp70 protein;; Hydantoinase/oxoprolinase | Mediator of RNA polymerase II transcription subunit 37a (Precursor) GN=At5g28540 OS=Arabidopsis thaliana (Mouse-ear cress) PE=1 SV=1 | PREDICTED: luminal-binding protein 5-like [Malus domestica] |
| c42232.graph_c0 | -- | -- | -- | -- | [OT] | -- | -- | FAM10 family protein At4g22670 GN=At4g22670 OS=Arabidopsis thaliana (Mouse-ear cress) PE=1 SV=1 | PREDICTED: FAM10 family protein At4g22670-like [Populus euphratica] |
| c54594.graph_c0 | [G] | Carbohydrate transport and metabolism | Molecular Function: transporter activity (GO:0005215);; Biological Process: transport (GO:0006810);; Cellular Component: integral component of membrane (GO:0016021);; | -- | [G] | Carbohydrate transport and metabolism | Major intrinsic protein | Probable aquaporin PIP1-4 OS=Arabidopsis thaliana (Mouse-ear cress) PE=1 SV=1 | aquaporin [Camellia oleifera] |
| c74634.graph_c1 | [R] | General function prediction only | Molecular Function: Hsp90 protein binding (GO:0051879);; | -- | [R] | General function prediction only | TPR repeat;; Tetratricopeptide repeat;; Tetratricopeptide repeat;; Tetratricopeptide repeat;; Tetratricopeptide repeat;; Tetratricopeptide repeat;; Tetratricopeptide repeat;; Tetratricopeptide repeat;; Tetratricopeptide repeat;; Tetratricopeptide repeat | TPR repeat-containing thioredoxin TTL4 GN=TTL4 OS=Arabidopsis thaliana (Mouse-ear cress) PE=2 SV=1 | Tetratricopeptide repeat (TPR)-like superfamily protein [Theobroma cacao] |
| c71002.graph_c0 | [O] | Posttranslational modification, protein turnover, chaperones | Molecular Function: ATP binding (GO:0005524);; Biological Process: protein folding (GO:0006457);; Molecular Function: unfolded protein binding (GO:0051082);; | K04043|0.0|vvi:100250929|similar to Heat shock 70 kDa protein, mitochondrial | [O] | Posttranslational modification, protein turnover, chaperones | Hsp70 protein;; FGGY family of carbohydrate kinases, C-terminal domain;; NAD-specific glutamate dehydrogenase | Heat shock 70 kDa protein, mitochondrial (Precursor) OS=Phaseolus vulgaris (Kidney bean) PE=1 SV=1 | PREDICTED: heat shock 70 kDa protein, mitochondrial-like [Sesamum indicum] |
| c65550.graph_c1 | [R] | General function prediction only | Molecular Function: naringenin 3-dioxygenase activity (GO:0045486);; Biological Process: oxidation-reduction process (GO:0055114);; | -- | [QR] | -- | 2OG-Fe(II) oxygenase superfamily;; non-haem dioxygenase in morphine synthesis N-terminal | Gibberellin 20 oxidase 1 GN=At4g25420 OS=Arabidopsis thaliana (Mouse-ear cress) PE=2 SV=2 | PREDICTED: gibberellin 20 oxidase 1 [Vitis vinifera] |
| c56909.graph_c0 | -- | -- | -- | -- | [S] | Function unknown | TIP41-like family | -- | PREDICTED: TIP41-like protein [Vitis vinifera] |
| c40973.graph_c0 | -- | -- | -- | -- | -- | -- | Helix-loop-helix DNA-binding domain | Transcription factor bHLH96 GN=T9N14.4 OS=Arabidopsis thaliana (Mouse-ear cress) PE=2 SV=1 | unnamed protein product [Vitis vinifera] |
| c68292.graph_c0 | -- | -- | Molecular Function: transferase activity (GO:0016740);; | K08827|1e-73|vvi:100247382|hypothetical protein LOC100247382 | [A] | RNA processing and modification | -- | DEAD-box ATP-dependent RNA helicase 42 GN=F9H16.10 OS=Arabidopsis thaliana (Mouse-ear cress) PE=1 SV=2 | PREDICTED: serine/threonine-protein kinase prpf4B-like [Vitis vinifera] |
| c69110.graph_c0 | -- | -- | Biological Process: transcription, DNA-templated (GO:0006351);; | K09284|1e-106|pop:POPTR_781029|RAP6 | -- | -- | AP2 domain | Floral homeotic protein APETALA 2 GN=AP2 OS=Arabidopsis thaliana (Mouse-ear cress) PE=1 SV=1 | hypothetical protein POPTR_0006s13460g [Populus trichocarpa] |
| c57312.graph_c0 | [R] | General function prediction only | -- | -- | [S] | Function unknown | -- | -- | Prenylyltransferase superfamily protein [Theobroma cacao] |
| c76283.graph_c0 | -- | -- | -- | K11592|0.0|vvi:100264308|hypothetical protein LOC100264308 | [A] | RNA processing and modification | Dicer dimerisation domain;; Helicase conserved C-terminal domain;; DEAD/DEAH box helicase;; Type III restriction enzyme, res subunit;; SNF2 family N-terminal domain | Endoribonuclease Dicer homolog 1 GN=T25K16.4 OS=Arabidopsis thaliana (Mouse-ear cress) PE=1 SV=2 | hypothetical protein JCGZ_14170 [Jatropha curcas] |
| c65576.graph_c0 | -- | -- | Molecular Function: zinc ion binding (GO:0008270);; | -- | [O] | Posttranslational modification, protein turnover, chaperones | Ring finger domain;; zinc-RING finger domain;; Zinc finger, C3HC4 type (RING finger);; Zinc finger, C3HC4 type (RING finger);; Zinc finger, C3HC4 type (RING finger) | -- | unnamed protein product [Coffea canephora] |
| c67310.graph_c0 | -- | -- | Molecular Function: peptidase activity (GO:0008233);; | -- | [O] | Posttranslational modification, protein turnover, chaperones | Xylanase inhibitor N-terminal;; Xylanase inhibitor C-terminal;; Eukaryotic aspartyl protease | Protein ASPARTIC PROTEASE IN GUARD CELL 2 (Precursor) GN=ASPG2 OS=Arabidopsis thaliana (Mouse-ear cress) PE=2 SV=1 | Eukaryotic aspartyl protease family protein, putative isoform 4, partial [Theobroma cacao] |
| c66582.graph_c0 | [M] | Cell wall/membrane/envelope biogenesis | Cellular Component: integral component of membrane (GO:0016021);; Molecular Function: cellulose synthase (UDP-forming) activity (GO:0016760);; Biological Process: cellulose biosynthetic process (GO:0030244);; | -- | -- | -- | Cellulose synthase;; Glycosyl transferase family group 2;; Glycosyltransferase like family 2;; Cellulose synthase | Cellulose synthase-like protein G2 OS=Arabidopsis thaliana (Mouse-ear cress) PE=2 SV=1 | PREDICTED: cellulose synthase-like protein G3 [Vitis vinifera] |
| c44601.graph_c0 | -- | -- | Molecular Function: transferase activity (GO:0016740);; | -- | -- | -- | -- | Probable xyloglucan glycosyltransferase 5 GN=CSLC5 OS=Arabidopsis thaliana (Mouse-ear cress) PE=1 SV=1 | hypothetical protein VITISV_007479 [Vitis vinifera] |
| c69084.graph_c0 | [G] | Carbohydrate transport and metabolism | Molecular Function: starch synthase activity (GO:0009011);; Cellular Component: amyloplast (GO:0009501);; Cellular Component: chloroplast (GO:0009507);; Biological Process: starch biosynthetic process (GO:0019252);; Molecular Function: starch binding (GO:2001070);; | -- | -- | -- | Carbohydrate binding domain (family 25);; Starch synthase catalytic domain;; Glycosyl transferases group 1;; Glycosyl transferase 4-like domain | Soluble starch synthase 3, chloroplastic/amyloplastic (Precursor) GN=SS3 OS=Solanum tuberosum (Potato) PE=1 SV=1 | PREDICTED: starch synthase 3, chloroplastic/amyloplastic [Vitis vinifera] |
| c62720.graph_c0 | -- | -- | -- | -- | [O] | Posttranslational modification, protein turnover, chaperones | Glutaredoxin | Uncharacterized protein At3g28850 GN=At3g28850 OS=Arabidopsis thaliana (Mouse-ear cress) PE=3 SV=1 | PREDICTED: uncharacterized protein At5g39865 [Vitis vinifera] |
| c74434.graph_c2 | -- | -- | Cellular Component: plasma membrane (GO:0005886);; Cellular Component: plasmodesma (GO:0009506);; Biological Process: photomorphogenesis (GO:0009640);; Biological Process: embryo development ending in seed dormancy (GO:0009793);; Biological Process: seed germination (GO:0009845);; Biological Process: regulation of flower development (GO:0009909);; Biological Process: meristem structural organization (GO:0009933);; Biological Process: seed dormancy process (GO:0010162);; Biological Process: sugar mediated signaling pathway (GO:0010182);; Biological Process: vegetative to reproductive phase transition of meristem (GO:0010228);; Biological Process: protein ubiquitination (GO:0016567);; Biological Process: lipid storage (GO:0019915);; Biological Process: response to freezing (GO:0050826);; | -- | -- | -- | Plant phosphoribosyltransferase C-terminal | -- | hypothetical protein glysoja_015812 [Glycine soja] |
| c74780.graph_c3 | -- | -- | Biological Process: rRNA processing (GO:0006364);; | -- | -- | -- | -- | -- | PREDICTED: uncharacterized protein LOC100253102 [Vitis vinifera] |
| c74525.graph_c3 | [F] | Nucleotide transport and metabolism | Biological Process: MAPK cascade (GO:0000165);; Molecular Function: ATP binding (GO:0005524);; Biological Process: pentose-phosphate shunt (GO:0006098);; Biological Process: rRNA processing (GO:0006364);; Biological Process: protein targeting to membrane (GO:0006612);; Molecular Function: phosphoribulokinase activity (GO:0008974);; Biological Process: response to cold (GO:0009409);; Cellular Component: chloroplast thylakoid membrane (GO:0009535);; Cellular Component: chloroplast stroma (GO:0009570);; Biological Process: detection of biotic stimulus (GO:0009595);; Biological Process: response to blue light (GO:0009637);; Biological Process: salicylic acid biosynthetic process (GO:0009697);; Biological Process: photosynthetic electron transport in photosystem I (GO:0009773);; Biological Process: systemic acquired resistance, salicylic acid mediated signaling pathway (GO:0009862);; Biological Process: jasmonic acid mediated signaling pathway (GO:0009867);; Biological Process: chloroplast relocation (GO:0009902);; Cellular Component: chloroplast envelope (GO:0009941);; Biological Process: stomatal complex morphogenesis (GO:0010103);; Biological Process: response to red light (GO:0010114);; Biological Process: response to chitin (GO:0010200);; Biological Process: photosystem II assembly (GO:0010207);; Biological Process: response to far red light (GO:0010218);; Biological Process: regulation of hydrogen peroxide metabolic process (GO:0010310);; Cellular Component: stromule (GO:0010319);; Biological Process: regulation of plant-type hypersensitive response (GO:0010363);; Biological Process: phosphorylation (GO:0016310);; Biological Process: cysteine biosynthetic process (GO:0019344);; Biological Process: negative regulation of defense response (GO:0031348);; Biological Process: regulation of protein dephosphorylation (GO:0035304);; Biological Process: defense response to bacterium (GO:0042742);; Biological Process: regulation of multi-organism process (GO:0043900);; Cellular Component: apoplast (GO:0048046);; Biological Process: defense response to fungus (GO:0050832);; | K00855|1e-170|pop:POPTR_712554|phosphoribulokinase (EC:2.7.1.19) | [TZ] | -- | Phosphoribulokinase / Uridine kinase family;; GAGA binding protein-like family | Phosphoribulokinase, chloroplastic (Precursor) OS=Triticum aestivum (Wheat) PE=2 SV=1 | unknown [Populus trichocarpa] |
| c78105.graph_c0 | -- | -- | -- | -- | -- | -- | -- | -- | DNA-directed RNA polymerase subunit alpha [Triticum urartu] |
| c65265.graph_c0 | -- | -- | Molecular Function: ubiquitin-protein ligase activity (GO:0004842);; Cellular Component: nucleus (GO:0005634);; Biological Process: phosphate ion transport (GO:0006817);; Molecular Function: zinc ion binding (GO:0008270);; Biological Process: plant-type hypersensitive response (GO:0009626);; Biological Process: systemic acquired resistance (GO:0009627);; Biological Process: salicylic acid biosynthetic process (GO:0009697);; Biological Process: response to salicylic acid (GO:0009751);; Biological Process: response to nitrate (GO:0010167);; Biological Process: regulation of salicylic acid metabolic process (GO:0010337);; Biological Process: cellular response to phosphate starvation (GO:0016036);; Biological Process: protein ubiquitination (GO:0016567);; Biological Process: defense response to bacterium (GO:0042742);; Biological Process: response to benzoic acid (GO:0080021);; | -- | -- | -- | SPX domain;; Zinc finger, C3HC4 type (RING finger);; Zinc finger, C3HC4 type (RING finger);; Ring finger domain;; Zinc finger, C3HC4 type (RING finger) | Probable E3 ubiquitin-protein ligase BAH1-like 1 GN=OsI_27296 OS=Oryza sativa subsp. indica (Rice) PE=3 SV=1 | hypothetical protein CICLE_v10021143mg [Citrus clementina] |
| c57488.graph_c0 | [K] | Transcription | Biological Process: RNA splicing, via endonucleolytic cleavage and ligation (GO:0000394);; Cellular Component: DNA-directed RNA polymerase IV complex (GO:0000418);; Cellular Component: DNA-directed RNA polymerase V complex (GO:0000419);; Molecular Function: DNA binding (GO:0003677);; Molecular Function: DNA-directed RNA polymerase activity (GO:0003899);; Cellular Component: DNA-directed RNA polymerase II, core complex (GO:0005665);; Biological Process: transcription from RNA polymerase II promoter (GO:0006366);; Biological Process: ubiquitin-dependent protein catabolic process (GO:0006511);; Biological Process: methionine biosynthetic process (GO:0009086);; Biological Process: virus induced gene silencing (GO:0009616);; Biological Process: photorespiration (GO:0009853);; Biological Process: vegetative phase change (GO:0010050);; Molecular Function: protein dimerization activity (GO:0046983);; Biological Process: response to misfolded protein (GO:0051788);; Biological Process: proteasome core complex assembly (GO:0080129);; | K03008|3e-78|vvi:100259467|hypothetical protein LOC100259467 | [K] | Transcription | RNA polymerase Rpb3/Rpb11 dimerisation domain;; RNA polymerase Rpb3/Rpb11 dimerisation domain | DNA-directed RNA polymerases II, IV and V subunit 11 GN=At3g52090 OS=Arabidopsis thaliana (Mouse-ear cress) PE=1 SV=1 | PREDICTED: DNA-directed RNA polymerases II, IV and V subunit 11 [Vitis vinifera] |
| c67878.graph_c0 | -- | -- | -- | -- | -- | -- | -- | -- | PREDICTED: uncharacterized protein LOC104426511 [Eucalyptus grandis] |
| c70016.graph_c0 | -- | -- | Cellular Component: THO complex part of transcription export complex (GO:0000445);; Biological Process: mRNA processing (GO:0006397);; | K13176|1e-133|vvi:100256436|hypothetical protein LOC100256436 | [S] | Function unknown | Tho complex subunit 7 | THO complex subunit 7A GN=F5E19.130 OS=Arabidopsis thaliana (Mouse-ear cress) PE=1 SV=1 | PREDICTED: THO complex subunit 7A [Sesamum indicum] |
| c70156.graph_c0 | [R] | General function prediction only | Molecular Function: nucleotide binding (GO:0000166);; Biological Process: endonucleolytic cleavage involved in rRNA processing (GO:0000478);; Biological Process: RNA methylation (GO:0001510);; Cellular Component: nucleolus (GO:0005730);; Cellular Component: heterotrimeric G-protein complex (GO:0005834);; Biological Process: protein import into nucleus (GO:0006606);; Biological Process: protein targeting to mitochondrion (GO:0006626);; Cellular Component: plasmodesma (GO:0009506);; Biological Process: protein maturation (GO:0051604);; Cellular Component: Cul4-RING ubiquitin ligase complex (GO:0080008);; | -- | [Z] | Cytoskeleton | WD domain, G-beta repeat;; NLE (NUC135) domain | Ribosome biogenesis protein WDR12 homolog {ECO:0000255|HAMAP-Rule:MF_03029} GN=CHLREDRAFT_128420 OS=Chlamydomonas reinhardtii (Chlamydomonas smithii) PE=1 SV=2 | PREDICTED: ribosome biogenesis protein WDR12 homolog [Vitis vinifera] |
| c46270.graph_c1 | -- | -- | -- | -- | -- | -- | -- | -- | hypothetical protein MIMGU_mgv1a014092mg [Erythranthe guttata] |
| c70896.graph_c1 | -- | -- | Molecular Function: zinc ion binding (GO:0008270);; | -- | [O] | Posttranslational modification, protein turnover, chaperones | Zinc finger, C3HC4 type (RING finger);; Zinc finger, C3HC4 type (RING finger) | Probable E3 ubiquitin-protein ligase LOG2 OS=Arabidopsis thaliana (Mouse-ear cress) PE=1 SV=1 | PREDICTED: probable E3 ubiquitin-protein ligase LOG2 [Vitis vinifera] |
| c75390.graph_c0 | -- | -- | -- | -- | -- | -- | Lecithin retinol acyltransferase | -- | PREDICTED: uncharacterized protein LOC104604719 isoform X2 [Nelumbo nucifera] |
| c66138.graph_c0 | -- | -- | -- | K12669|1e-160|rcu:RCOM_0905660|Tumor suppressor candidate, putative | [O] | Posttranslational modification, protein turnover, chaperones | OST3 / OST6 family | Probable dolichyl-diphosphooligosaccharide--protein glycosyltransferase subunit 3B (Precursor) GN=OST3B OS=Arabidopsis thaliana (Mouse-ear cress) PE=2 SV=1 | PREDICTED: polyubiquitin-like isoform 3 [Cucumis sativus] |
| c76364.graph_c0 | [S] | Function unknown | Molecular Function: DNA binding (GO:0003677);; Molecular Function: RNA binding (GO:0003723);; Molecular Function: RNA-directed DNA polymerase activity (GO:0003964);; Biological Process: RNA-dependent DNA replication (GO:0006278);; | -- | [R] | General function prediction only | Reverse transcriptase (RNA-dependent DNA polymerase);; Endonuclease-reverse transcriptase;; Endonuclease/Exonuclease/phosphatase family | Uncharacterized protein ycf19 GN=ycf19 OS=Guillardia theta (Cryptomonas phi) PE=3 SV=1 | PREDICTED: uncharacterized protein LOC103417687 [Malus domestica] |
| c71472.graph_c0 | -- | -- | -- | -- | -- | -- | Transcriptional activator | WEB family protein At3g02930, chloroplastic (Precursor) GN=At3g02930 OS=Arabidopsis thaliana (Mouse-ear cress) PE=2 SV=1 | PREDICTED: structural maintenance of chromosomes protein 1A [Sesamum indicum] |
| c75905.graph_c0 | -- | -- | -- | -- | [T] | Signal transduction mechanisms | NB-ARC domain;; Leucine Rich repeats (2 copies);; Leucine rich repeat;; Leucine Rich Repeat;; Leucine rich repeat | Putative disease resistance protein RGA3 GN=RGA3 OS=Solanum bulbocastanum (Wild potato) PE=2 SV=2 | hypothetical protein JCGZ_13002 [Jatropha curcas] |
| c35908.graph_c0 | -- | -- | Molecular Function: anthranilate N-benzoyltransferase activity (GO:0047672);; | -- | -- | -- | Transferase family | Uncharacterized acetyltransferase At3g50280 GN=At3g50280 OS=Arabidopsis thaliana (Mouse-ear cress) PE=3 SV=1 | hypothetical protein MIMGU_mgv1a005405mg [Erythranthe guttata] |
| c71276.graph_c0 | [R] | General function prediction only | Biological Process: metabolic process (GO:0008152);; Molecular Function: transferase activity, transferring acyl groups other than amino-acyl groups (GO:0016747);; | -- | -- | -- | Alpha/beta hydrolase family;; alpha/beta hydrolase fold;; Diacylglycerol acyltransferase | Acyltransferase-like protein At1g54570, chloroplastic (Precursor) GN=At1g54570 OS=Arabidopsis thaliana (Mouse-ear cress) PE=2 SV=1 | PREDICTED: acyltransferase-like protein At3g26840, chloroplastic [Vitis vinifera] |
| c66610.graph_c0 | [J] | Translation, ribosomal structure and biogenesis | Molecular Function: sequence-specific DNA binding transcription factor activity (GO:0003700);; Biological Process: regulation of transcription, DNA-templated (GO:0006355);; Biological Process: ribosome biogenesis (GO:0042254);; | K14574|0.0|rcu:RCOM_0559400|Shwachman-Bodian-Diamond syndrome protein, putative | [J] | Translation, ribosomal structure and biogenesis | SBDS protein C-terminal domain;; Shwachman-Bodian-Diamond syndrome (SBDS) protein | -- | PREDICTED: ribosome maturation protein SBDS [Nelumbo nucifera] |
| c42930.graph_c0 | -- | -- | Biological Process: regulation of transcription, DNA-templated (GO:0006355);; | -- | -- | -- | GRAS domain family | Scarecrow-like protein 6 GN=SCL6 OS=Arabidopsis thaliana (Mouse-ear cress) PE=1 SV=1 | GRAS family transcription factor [Theobroma cacao] |
| c69397.graph_c0 | [TK] | -- | Molecular Function: phosphorelay response regulator activity (GO:0000156);; Biological Process: phosphorelay signal transduction system (GO:0000160);; Molecular Function: DNA binding (GO:0003677);; Molecular Function: chromatin binding (GO:0003682);; Biological Process: regulation of transcription, DNA-templated (GO:0006355);; Molecular Function: kinase activity (GO:0016301);; Biological Process: phosphorylation (GO:0016310);; Biological Process: intracellular signal transduction (GO:0035556);; | K14491|0.0|vvi:100250832|hypothetical protein LOC100250832 | [GC] | -- | UDP-glucoronosyl and UDP-glucosyl transferase;; Response regulator receiver domain;; Myb-like DNA-binding domain | Abscisate beta-glucosyltransferase GN=AOG OS=Phaseolus angularis (Azuki bean) PE=1 SV=1 | PREDICTED: two-component response regulator ARR1 isoform X1 [Vitis vinifera] |
| c65620.graph_c0 | [C] | Energy production and conversion | Cellular Component: mitochondrial respiratory chain complex III (GO:0005750);; Molecular Function: ubiquinol-cytochrome-c reductase activity (GO:0008121);; Cellular Component: integral component of membrane (GO:0016021);; Biological Process: electron transport chain (GO:0022900);; Molecular Function: metal ion binding (GO:0046872);; Molecular Function: 2 iron, 2 sulfur cluster binding (GO:0051537);; | K00411|1e-157|vvi:100257494|hypothetical protein LOC100257494 | [C] | Energy production and conversion | Rieske [2Fe-2S] domain;; Ubiquinol cytochrome reductase transmembrane region | Cytochrome b-c1 complex subunit Rieske-3, mitochondrial (Precursor) OS=Nicotiana tabacum (Common tobacco) PE=2 SV=1 | PREDICTED: cytochrome b-c1 complex subunit Rieske-4, mitochondrial-like [Sesamum indicum] |
| c73250.graph_c0 | [MG] | -- | Molecular Function: catalytic activity (GO:0003824);; Biological Process: cellular metabolic process (GO:0044237);; Molecular Function: coenzyme binding (GO:0050662);; | -- | [V] | Defense mechanisms | NAD dependent epimerase/dehydratase family;; 3-beta hydroxysteroid dehydrogenase/isomerase family;; NADH(P)-binding;; Male sterility protein;; NmrA-like family | Cinnamoyl-CoA reductase 1 GN=T24D18.5 OS=Arabidopsis thaliana (Mouse-ear cress) PE=1 SV=1 | cinnamyl alcohol dehydrogenase [Camellia sinensis] |
| c73777.graph_c0 | -- | -- | -- | -- | -- | -- | F-box domain;; F-box-like | Putative F-box protein At4g38870 GN=At4g38870 OS=Arabidopsis thaliana (Mouse-ear cress) PE=3 SV=1 | PREDICTED: F-box protein At5g49610-like isoform X7 [Vitis vinifera] |
| c62353.graph_c0 | [F] | Nucleotide transport and metabolism | Molecular Function: adenylate kinase activity (GO:0004017);; Molecular Function: copper ion binding (GO:0005507);; Molecular Function: ATP binding (GO:0005524);; Cellular Component: mitochondrion (GO:0005739);; Cellular Component: plasma membrane (GO:0005886);; Biological Process: purine nucleotide metabolic process (GO:0006163);; Biological Process: response to cadmium ion (GO:0046686);; Biological Process: nucleotide phosphorylation (GO:0046939);; | K00939|1e-159|pop:POPTR_823409|adenylate kinase (EC:2.7.4.3) | [F] | Nucleotide transport and metabolism | Adenylate kinase;; Adenylate kinase, active site lid | Adenylate kinase 4 GN=ADK-B OS=Oryza sativa subsp. japonica (Rice) PE=2 SV=1 | hypothetical protein POPTR_0012s09720g [Populus trichocarpa] |
| c70644.graph_c1 | -- | -- | Molecular Function: binding (GO:0005488);; | -- | -- | -- | Helix-loop-helix DNA-binding domain | Transcription factor bHLH51 GN=T7M7.8 OS=Arabidopsis thaliana (Mouse-ear cress) PE=2 SV=1 | PREDICTED: transcription factor bHLH51-like [Malus domestica] |
| c61975.graph_c0 | -- | -- | Molecular Function: calcium ion binding (GO:0005509);; Cellular Component: cytosol (GO:0005829);; Biological Process: vegetative to reproductive phase transition of meristem (GO:0010228);; Biological Process: protein desumoylation (GO:0016926);; Biological Process: hydrogen peroxide biosynthetic process (GO:0050665);; | -- | [S] | Function unknown | Domain of unknown function (DUF4205) | Classical arabinogalactan protein 9 (Precursor) GN=AGP9 OS=Arabidopsis thaliana (Mouse-ear cress) PE=1 SV=2 | Ubiquitin interaction motif-containing protein [Theobroma cacao] |
| c19964.graph_c0 | [R] | General function prediction only | Molecular Function: nucleotide binding (GO:0000166);; Molecular Function: nucleic acid binding (GO:0003676);; | -- | [R] | General function prediction only | RNA recognition motif. (a.k.a. RRM, RBD, or RNP domain);; RNA recognition motif (a.k.a. RRM, RBD, or RNP domain);; RNA recognition motif. (a.k.a. RRM, RBD, or RNP domain) | Oligouridylate-binding protein 1B GN=UBP1B OS=Arabidopsis thaliana (Mouse-ear cress) PE=1 SV=1 | nucleolysin tia-1, putative [Ricinus communis] |
| c65361.graph_c0 | [LR] | -- | Molecular Function: hydrolase activity (GO:0016787);; | -- | [T] | Signal transduction mechanisms | NUDIX domain | Nudix hydrolase 16, mitochondrial (Precursor) GN=T2E22.9 OS=Arabidopsis thaliana (Mouse-ear cress) PE=2 SV=1 | PREDICTED: nudix hydrolase 16, mitochondrial-like [Pyrus x bretschneideri] |
| c75881.graph_c0 | -- | -- | -- | -- | [BK] | -- | -- | Chromatin structure-remodeling complex protein SYD GN=SYD OS=Arabidopsis thaliana (Mouse-ear cress) PE=1 SV=1 | PREDICTED: chromatin structure-remodeling complex protein SYD [Prunus mume] |
| c64348.graph_c0 | [Q] | Secondary metabolites biosynthesis, transport and catabolism | Molecular Function: oxidoreductase activity, acting on single donors with incorporation of molecular oxygen, incorporation of two atoms of oxygen (GO:0016702);; | K09840|0.0|vvi:100251100|hypothetical protein LOC100251100 | [Q] | Secondary metabolites biosynthesis, transport and catabolism | Retinal pigment epithelial membrane protein | Probable carotenoid cleavage dioxygenase 4, chloroplastic (Precursor) GN=T18B16.140 OS=Arabidopsis thaliana (Mouse-ear cress) PE=1 SV=1 | carotenoid cleavage dioxygenase 4 [Lycium barbarum] |
| c70660.graph_c4 | [C] | Energy production and conversion | Biological Process: malate metabolic process (GO:0006108);; Molecular Function: malate dehydrogenase (decarboxylating) (NAD+) activity (GO:0016619);; Molecular Function: metal ion binding (GO:0046872);; Molecular Function: NAD binding (GO:0051287);; Biological Process: oxidation-reduction process (GO:0055114);; | -- | [C] | Energy production and conversion | Malic enzyme, N-terminal domain | NADP-dependent malic enzyme OS=Populus trichocarpa (Western balsam poplar) PE=2 SV=3 | PREDICTED: NADP-dependent malic enzyme-like isoform X1 [Solanum lycopersicum] |
| c40974.graph_c0 | -- | -- | -- | -- | -- | -- | Protein of unknown function (DUF3223) | DNA-directed RNA polymerase V subunit 1 GN=At2g40030 OS=Arabidopsis thaliana (Mouse-ear cress) PE=1 SV=1 | PREDICTED: DNA-directed RNA polymerase V subunit 1 [Vitis vinifera] |
| c67100.graph_c0 | -- | -- | Biological Process: lipid transport (GO:0006869);; Molecular Function: lipid binding (GO:0008289);; | -- | -- | -- | Protease inhibitor/seed storage/LTP family;; Probable lipid transfer | Non-specific lipid-transfer protein (Precursor) OS=Helianthus annuus (Common sunflower) PE=3 SV=1 | non-specific lipid-transfer protein [Helianthus annuus] |
| c60372.graph_c0 | -- | -- | -- | -- | [M] | Cell wall/membrane/envelope biogenesis | Ankyrin repeats (3 copies);; Ankyrin repeat;; Ankyrin repeat | -- | unnamed protein product [Vitis vinifera] |
| c70435.graph_c0 | [EH] | -- | Molecular Function: magnesium ion binding (GO:0000287);; Molecular Function: acetolactate synthase activity (GO:0003984);; Biological Process: isoleucine biosynthetic process (GO:0009097);; Biological Process: valine biosynthetic process (GO:0009099);; Molecular Function: thiamine pyrophosphate binding (GO:0030976);; Molecular Function: flavin adenine dinucleotide binding (GO:0050660);; | K01652|0.0|rcu:RCOM_1506000|acetolactate synthase, putative (EC:2.2.1.6) | [EH] | -- | Thiamine pyrophosphate enzyme, N-terminal TPP binding domain;; Thiamine pyrophosphate enzyme, C-terminal TPP binding domain;; Thiamine pyrophosphate enzyme, central domain | Acetolactate synthase 1, chloroplastic (Precursor) OS=Nicotiana tabacum (Common tobacco) PE=1 SV=1 | PREDICTED: acetolactate synthase 3, chloroplastic-like [Prunus mume] |
| c68481.graph_c2 | [E] | Amino acid transport and metabolism | Molecular Function: glutamate-ammonia ligase activity (GO:0004356);; Molecular Function: ATP binding (GO:0005524);; Cellular Component: cytoplasm (GO:0005737);; Biological Process: glutamine biosynthetic process (GO:0006542);; Biological Process: nitrogen fixation (GO:0009399);; | K01915|4e-50|osa:4330649|Os02g0735200 | [E] | Amino acid transport and metabolism | Glutamine synthetase, beta-Grasp domain | Glutamine synthetase cytosolic isozyme 1-1 OS=Oryza sativa subsp. japonica (Rice) PE=1 SV=1 | glutamine synthetase [Camellia sinensis] |
| c76040.graph_c0 | [V] | Defense mechanisms | Molecular Function: ATP binding (GO:0005524);; Biological Process: ATP catabolic process (GO:0006200);; Cellular Component: integral component of membrane (GO:0016021);; Molecular Function: 2-alkenal reductase [NAD(P)] activity (GO:0032440);; Molecular Function: ATPase activity, coupled to transmembrane movement of substances (GO:0042626);; Biological Process: transmembrane transport (GO:0055085);; Biological Process: oxidation-reduction process (GO:0055114);; | -- | [Q] | Secondary metabolites biosynthesis, transport and catabolism | ABC transporter transmembrane region;; ABC transporter;; ABC transporter;; AAA domain | ABC transporter C family member 8 (Precursor) GN=MXL8.11 OS=Arabidopsis thaliana (Mouse-ear cress) PE=2 SV=3 | PREDICTED: ABC transporter C family member 8-like isoform X1 [Vitis vinifera] |
| c74858.graph_c0 | [R] | General function prediction only | Molecular Function: protein serine/threonine kinase activity (GO:0004674);; Molecular Function: protein kinase activator activity (GO:0030295);; Molecular Function: adenyl nucleotide binding (GO:0030554);; Biological Process: cellular response to glucose starvation (GO:0042149);; Biological Process: positive regulation of protein kinase activity (GO:0045860);; Biological Process: protein autophosphorylation (GO:0046777);; | -- | [C] | Energy production and conversion | CBS domain | Sucrose nonfermenting 4-like protein GN=F7G19.11 OS=Arabidopsis thaliana (Mouse-ear cress) PE=1 SV=1 | PREDICTED: sucrose nonfermenting 4-like protein isoform X1 [Vitis vinifera] |
| c63818.graph_c0 | -- | -- | -- | -- | -- | -- | Protein of unknown function (DUF581) | -- | hypothetical protein VITISV_039443 [Vitis vinifera] |
| c56701.graph_c0 | [FJ] | -- | Cellular Component: cytosol (GO:0005829);; Biological Process: metabolic process (GO:0008152);; Molecular Function: zinc ion binding (GO:0008270);; Molecular Function: hydrolase activity (GO:0016787);; | -- | [F] | Nucleotide transport and metabolism | Cytidine and deoxycytidylate deaminase zinc-binding region | tRNA(adenine(34)) deaminase, chloroplastic (Precursor) GN=TADA OS=Arabidopsis thaliana (Mouse-ear cress) PE=1 SV=1 | PREDICTED: tRNA(adenine(34)) deaminase, chloroplastic [Nelumbo nucifera] |
| c72790.graph_c0 | [EH] | -- | Molecular Function: sucrose alpha-glucosidase activity (GO:0004575);; Biological Process: carbohydrate metabolic process (GO:0005975);; | K01193|0.0|rcu:RCOM_0293270|Beta-fructofuranosidase, insoluble isoenzyme 3 precursor, putative (EC:3.2.1.26) | [G] | Carbohydrate transport and metabolism | Glycosyl hydrolases family 32 N-terminal domain;; Glycosyl hydrolases family 32 C terminal;; Phosphoadenosine phosphosulfate reductase family;; Thioredoxin | Beta-fructofuranosidase, insoluble isoenzyme 1 (Precursor) GN=INV1 OS=Daucus carota (Wild carrot) PE=1 SV=1 | PREDICTED: beta-fructofuranosidase, insoluble isoenzyme 1-like [Citrus sinensis] |
| c19950.graph_c0 | -- | -- | Cellular Component: mitochondrial outer membrane translocase complex (GO:0005742);; Biological Process: protein import into mitochondrial matrix (GO:0030150);; | -- | -- | -- | TOM7 family | Mitochondrial import receptor subunit TOM7-1 GN=TOM7-1 OS=Arabidopsis thaliana (Mouse-ear cress) PE=1 SV=1 | PREDICTED: mitochondrial import receptor subunit TOM7-1-like isoform 1 [Glycine max] |
| c61484.graph_c0 | -- | -- | Molecular Function: nucleotide binding (GO:0000166);; Cellular Component: intracellular (GO:0005622);; | -- | [DT] | -- | -- | GTP-binding protein ERG GN=ERG OS=Arabidopsis thaliana (Mouse-ear cress) PE=1 SV=2 | PREDICTED: GTP-binding protein ERG [Vitis vinifera] |
| c61055.graph_c0 | -- | -- | Cellular Component: Golgi apparatus (GO:0005794);; Biological Process: protein glycosylation (GO:0006486);; Biological Process: secondary cell wall biogenesis (GO:0009834);; Biological Process: glucuronoxylan biosynthetic process (GO:0010417);; Biological Process: pollen exine formation (GO:0010584);; Molecular Function: galactosylgalactosylxylosylprotein 3-beta-glucuronosyltransferase activity (GO:0015018);; Cellular Component: membrane (GO:0016020);; Molecular Function: xylosyltransferase activity (GO:0042285);; | -- | [O] | Posttranslational modification, protein turnover, chaperones | Glycosyltransferase family 43 | Probable beta-1,4-xylosyltransferase IRX9H GN=IRX9H OS=Arabidopsis thaliana (Mouse-ear cress) PE=2 SV=2 | PREDICTED: probable beta-1,4-xylosyltransferase IRX9H [Vitis vinifera] |
| c67575.graph_c0 | -- | -- | Cellular Component: plasma membrane (GO:0005886);; Molecular Function: kinase activity (GO:0016301);; Biological Process: phosphorylation (GO:0016310);; | -- | [R] | General function prediction only | Leucine Rich repeats (2 copies);; Leucine rich repeat;; Leucine Rich Repeat;; Leucine rich repeat | Probable inactive receptor kinase At1g27190 (Precursor) GN=At1g27190 OS=Arabidopsis thaliana (Mouse-ear cress) PE=1 SV=1 | Receptor like protein 44 isoform 1 [Theobroma cacao] |
| c64540.graph_c0 | -- | -- | -- | -- | -- | -- | -- | Putative nuclear matrix constituent protein 1-like protein GN=At5g65770 OS=Arabidopsis thaliana (Mouse-ear cress) PE=2 SV=1 | PREDICTED: putative nuclear matrix constituent protein 1-like protein isoform X2 [Vitis vinifera] |
| c74630.graph_c0 | -- | -- | -- | K12879|0.0|vvi:100254201|hypothetical protein LOC100254201 | [K] | Transcription | Transcription factor/nuclear export subunit protein 2;; Transcription- and export-related complex subunit | THO complex subunit 2 GN=F5A9.21/F5A9.22 OS=Arabidopsis thaliana (Mouse-ear cress) PE=1 SV=1 | PREDICTED: THO complex subunit 2 [Vitis vinifera] |
| c53634.graph_c1 | -- | -- | -- | -- | -- | -- | Domain of unknown function (DUF4408) | -- | PREDICTED: uncharacterized protein LOC105176754 [Sesamum indicum] |
| c56631.graph_c0 | [RTKL] | -- | Molecular Function: protein kinase activity (GO:0004672);; Biological Process: phosphorylation (GO:0016310);; | -- | [T] | Signal transduction mechanisms | Protein tyrosine kinase;; Protein kinase domain | Putative receptor-like protein kinase At5g39000 (Precursor) GN=At5g39000 OS=Arabidopsis thaliana (Mouse-ear cress) PE=3 SV=1 | FERONIA receptor-like kinase [Glycine max] |
| c78425.graph_c0 | -- | -- | Biological Process: lipid transport (GO:0006869);; Molecular Function: lipid binding (GO:0008289);; | -- | -- | -- | Probable lipid transfer;; Protease inhibitor/seed storage/LTP family | Non-specific lipid-transfer protein-like protein At2g13820 (Precursor) GN=At2g13820 OS=Arabidopsis thaliana (Mouse-ear cress) PE=1 SV=1 | hypothetical protein JCGZ_02198 [Jatropha curcas] |
[truncated: 251,315 more chars]
